# Discovery of XL01126: A Potent, Fast, Cooperative, Selective, Orally Bioavailable, and Blood–Brain Barrier Penetrant PROTAC Degrader of Leucine-Rich Repeat Kinase 2

**DOI:** 10.1021/jacs.2c05499

**Published:** 2022-08-25

**Authors:** Xingui Liu, Alexia F. Kalogeropulou, Sofia Domingos, Nikolai Makukhin, Raja S. Nirujogi, Francois Singh, Natalia Shpiro, Anton Saalfrank, Esther Sammler, Ian G. Ganley, Rui Moreira, Dario R. Alessi, Alessio Ciulli

**Affiliations:** † Centre for Targeted Protein Degradation, Division of Biological Chemistry and Drug Discovery, School of Life Sciences, University of Dundee, Dow Street, Dundee DD1 5EH, United Kingdom; ‡ Medical Research Council (MRC) Protein Phosphorylation and Ubiquitylation Unit, School of Life Sciences, University of Dundee, Dow Street, Dundee DD1 5EH, United Kingdom; § Research Institute for Medicines (iMed.ULisboa), Faculty of Pharmacy, Universidade de Lisboa, Av. Prof. Gama Pinto, 1649-003 Lisboa, Portugal

## Abstract

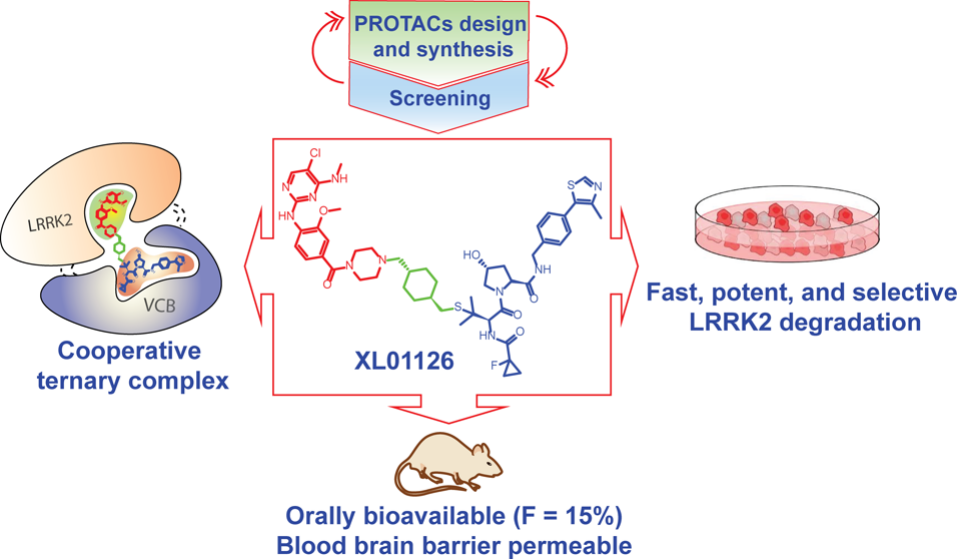

Leucine-rich repeat kinase 2 (LRRK2) is one of the most promising targets for Parkinson’s disease. LRRK2-targeting strategies have primarily focused on type 1 kinase inhibitors, which, however, have limitations as the inhibited protein can interfere with natural mechanisms, which could lead to undesirable side effects. Herein, we report the development of LRRK2 proteolysis targeting chimeras (PROTACs), culminating in the discovery of degrader XL01126, as an alternative LRRK2-targeting strategy. Initial designs and screens of PROTACs based on ligands for E3 ligases von Hippel–Lindau (VHL), Cereblon (CRBN), and cellular inhibitor of apoptosis (cIAP) identified the best degraders containing thioether-conjugated VHL ligand VH101. A second round of medicinal chemistry exploration led to qualifying XL01126 as a fast and potent degrader of LRRK2 in multiple cell lines, with DC_50_ values within 15–72 nM, *D*_max_ values ranging from 82 to 90%, and degradation half-lives spanning from 0.6 to 2.4 h. XL01126 exhibits high cell permeability and forms a positively cooperative ternary complex with VHL and LRRK2 (α = 5.7), which compensates for a substantial loss of binary binding affinities to VHL and LRRK2, underscoring its strong degradation performance in cells. Remarkably, XL01126 is orally bioavailable (*F* = 15%) and can penetrate the blood–brain barrier after either oral or parenteral dosing in mice. Taken together, these experiments qualify XL01126 as a suitable degrader probe to study the noncatalytic and scaffolding functions of LRRK2 *in vitro* and *in vivo* and offer an attractive starting point for future drug development.

## Introduction

Around 10 million people worldwide are living with Parkinson’s disease (PD),^1^ a progressive neurodegenerative disorder characterized by both motor (*e.g.*, bradykinesia, resting tremor, postural instability, rigidity) and nonmotor (*e.g.*, memory loss, hyposmia) disabilities. Current PD treatment is limited to motor symptom management with dopamine replacement or by enhancing the activity of the remaining dopaminergic neurons. No known therapy is available that can slow down the progress or prevent the onset of the disease. Furthermore, PD cases are growing at a fast ever speed and are projected to increase to over 17.5 million by 2040 due to the fast-growing aging population.^2^ While aging remains to be the major risk factor of PD, >20 genes have been identified to be associated with the onset and progress of PD,^3^ suggesting the potential of discovering disease-modifying PD treatments.

Leucine-rich repeat kinase 2 (LRRK2), encoded by *LRRK2* gene, is a large (286 kDa), multidomain protein that, in addition to its kinase domain, possesses a second enzymatic guanosine triphosphatase (GTPase) domain and several other domains and motifs that are involved in protein–protein interactions.^4^ Pathological mutations in the kinase domain and GTPase domain of LRRK2, such as G2019S and R1441C/G/H mutations, can increase the kinase activity of LRRK2 and eventually lead to pathogenic hallmarks associated with PD, such as ciliogenesis inhibition,^5,6^ defective mitophagy and autophagy,^7−9^ and mitochondrial dysfunction.^10^ Increased LRRK2 kinase activity, independent of LRRK2 mutations, has also been reported in idiopathic PD patients.^11^ Conversely, LRRK2 knockout or pharmacological inhibition of LRRK2 kinase activity is neuroprotective in cellular and animal models.^12−15^ These observations provide a strong rationale for targeting LRRK2 to treat PD.

Over the past years, several LRRK2 kinase inhibitors have been developed, including LRRK2-IN-1,^16^ HG-10-102-01,^17^ MLi-2,^18^ PF-06447475,^19^ and DNL201 and DNL151, which are the first two LRRK2 kinase inhibitors in clinical trials.^20,21^ However, all of these inhibitors are ATP-competitive type 1 kinase inhibitors, which preferably bind to the closed active conformation of LRRK2, leading to dephosphorylation of Ser935 and other biomarker sites, LRRK2 aggregation, and mislocalization to microtubules.^22,23^ These unintended effects may interfere with vesicle trafficking and could underlie undesirable on-target side effects observed on lungs and kidneys.^24,25^ Alternative LRRK2-targeting strategies, such as G2019S LRRK2 selective inhibitors,^26,27^ LRRK2 dimerization inhibitors,^28^ GTPase inhibitors, antisense oligonucleotide,^29^ type 2 LRRK2 kinase inhibitors,^30^ and LRRK2 proteolysis targeting chimeras (PROTACs),^31−34^ have therefore been proposed and are under active exploration.

As one of the most promising disease-modifying targets, LRRK2 lies at the nexus of an emerging signaling network of high relevance for understanding and developing treatments for PD.^35^ Although three LRRK2-targeting therapies^29,36,37^ are already in clinical trials, the exact mechanism by which LRRK2 mutations and their kinase activity contribute to the development of PD is still under investigation. Rab GTPases implicated in vesicular trafficking have been identified as *bona fide* physiological substrates of LRRK2,^37^ but many components involved in the upstream and downstream wiring of LRRK2 signaling pathways are yet to be discovered, and the question remains as to whether LRRK2 kinase inhibitors will have beneficial disease-modifying effects in PD patients. More in-depth LRRK2 target validation is therefore warranted.

Induced target protein degradation is a paradigm-shifting drug discovery approach. Heterobifunctional degraders (also known as PROTACs) can induce target protein degradation by recruiting an E3 ubiquitin ligase in proximity to the target protein, resulting in the polyubiquitination and subsequent degradation of the target protein by the proteasome.^38−40^ More than 15 PROTAC degraders are in or approaching the clinic currently,^41−43^ against a variety of targets, including hormone receptors (*e.g*. AR and ER), transcription factor (*e.g*. STAT3), antiapoptotic protein (*e.g*. BCL-XL), kinases (*e.g*. BTK and IRAK4), and epigenetic proteins (*e.g.*, BRD9). PROTAC is not only an emerging drug discovery modality but also offers new chemical tools for target identification and validation and for deciphering target biology.^44,45^ For example, PROTAC-mediated degradation can reveal the noncatalytic activity of protein kinases.^46,47^ Herein, we report the discovery and characterization of XL01126, a von Hippel–Lindau (VHL)-based, fast, potent, cooperative, and selective LRRK2 PROTAC degrader that is also orally bioavailable and blood–brain barrier (BBB)-permeable. XL01126 qualifies as a chemical probe to study LRRK2 biology, further validate the target as a therapeutic concept in PD, and usher in future drug development.

## Results

### Identification of Initial VH101 Thioether-Linked PROTACs as Moderate LRRK2 Degraders

**Figure 1:**
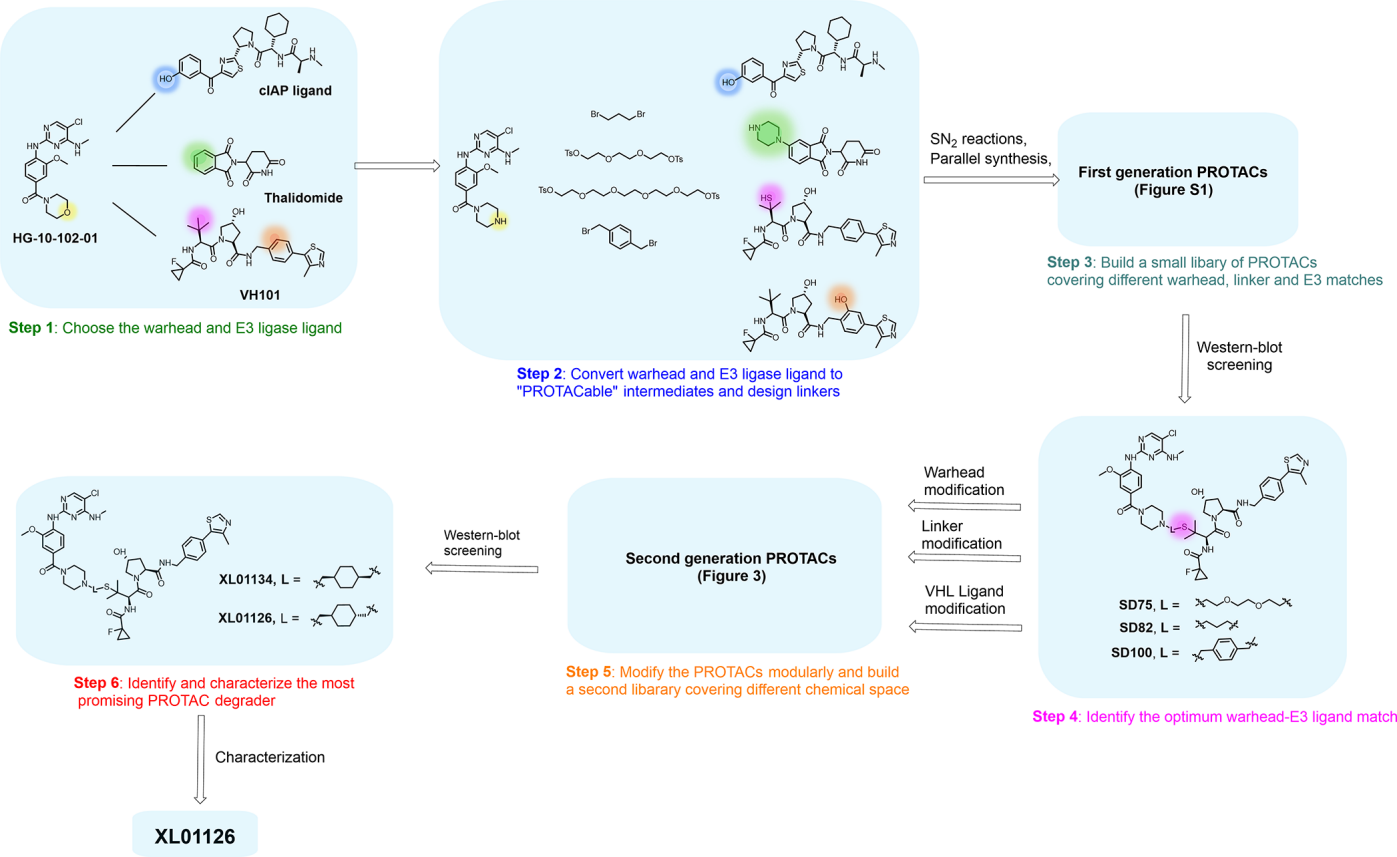
Our step-by-step PROTAC discovery strategies.

We began our efforts by designing and synthesizing a small set of PROTACs aiming to maximize the sampling of chemical space and target–PROTAC–E3 ternary complex pairing. HG-10-102-01 (Figure 1), a BBB penetrant type 1 LRRK2 inhibitor, was chosen as the LRRK2 ligand on the basis of its small molecular size and favorable physicochemical properties.^17^ According to the homology modeling of HG-10-102-01 with LRRK2, the morpholine ring is pointing toward the solvent,^17^ suggesting a suitable exiting vector for the PROTAC linkage. We converted the morpholine ring to piperazine to facilitate linker attachment. For the E3 ubiquitin ligases, we decided to recruit Cereblon (CRBN), a cellular inhibitor of apoptosis (cIAP), and VHL, which have readily available ligands with known “PROTACable” sites^48^ (Figure 1). After converting both the warhead and E3 ligase ligands into “PROTACable” intermediates, they were tethered together through linkers and a small library of first-generation compounds containing 12 LRRK2 PROTACs (Figure S1) was generated (Schemes S3–S5).

**Figure 2:**
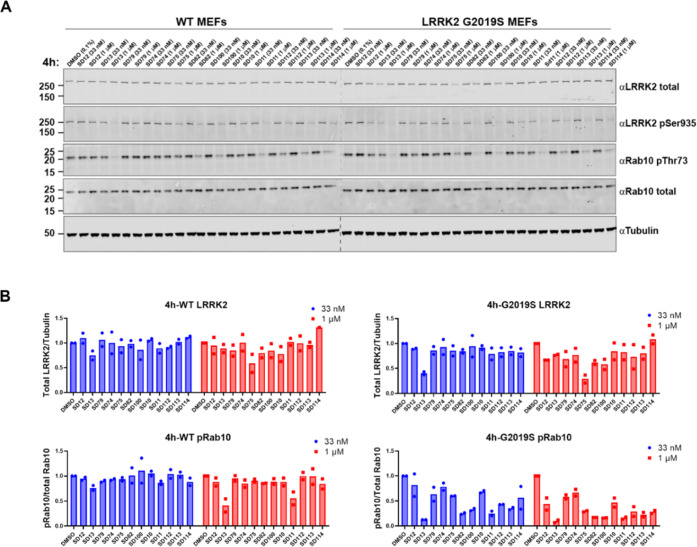
Screening of the first-generation PROTACs in wild-type (WT) and G2019S LRRK2 MEFs. (A) Representative Western blots monitoring the total LRRK2, LRRK2-pSer935, Rab10-pThr73, total Rab10, and tubulin levels following the treatment of WT and G2019S MEFs with the indicated compounds at 33 nM, 1 μM, or dimethyl sulfoxide (DMSO) for 4 h. (B) Quantitative analysis of the relative LRRK2 protein and Rab10-pThr73 levels, which are presented as ratios of total LRRK2/tubulin or Rab10-pThr73/total Rab10, normalized to the DMSO-treated sample. Data were obtained from two biological independent experiments.

These PROTACs were then biologically evaluated in mouse embryonic fibroblasts (MEFs) by Western blotting. Briefly, MEFs were treated with compounds at 33 nM and 1 μM for 4 h (Figure 2) and 24 h (Figure S2) separately, and the intracellular level of LRRK2, phosphorylated LRRK2 at Ser935, and phosphorylated Rab10 (pRab10) at Thr73 was determined. Rab10^5,49^ is one of the *bona fide* substrates of LRRK2, whose phosphorylation status is directly affected by the LRRK2 kinase activity and protein level. Phosphorylation of LRRK2 at Ser935 is a well-studied biomarker site used to assess the efficacy of type 1 LRRK2 inhibitors.^30^ HG-10-102-01-based PROTACs can potentially dephosphorylate LRRK2 at Ser935 through both LRRK2 degradation and inhibition. Among the first-generation PROTACs, compounds SD75, SD82, and SD100 (Figure 1) degraded 30–70% of G2019S LRRK2 at 1 μM/4 h (Figure 2) and achieved 70–85% G2019S LRRK2 degradation after 1 μM/24 h treatment (Figure S2). These three compounds also showed substantial dephosphorylation of LRRK2 and Rab10, with 75–90% pRab10 dephosphorylated after 1 μM/24 h treatment in G2019S LRRK2 MEFs (Figure S2). A fourth compound, SD13, also looked promising as it degraded 60% G2019S LRRK2 at 33 nM/4 h treatment (Figure 2) and 68% G2019S LRRK2 at 33 nM/24 h (Figure S2). However, less G2019S LRRK2 was degraded upon 1 μM treatment by SD13, compared to the 33 nM treatments, suggestive of the “hook effect”.^50^ Although SD75, SD82, and SD100 showed only moderate LRRK2 degradation, they did not show any sign of the “hook effect” at a 1 μM concentration. Notably, all three compounds share the same E3 ligase and ligand (VHL, VH101) and exit vector out of the *tert*-leucine group, suggesting a potential hot-spot of the ternary complex formation between VHL and LRRK2. We therefore decided to focus further medicinal chemistry optimization on this chemical series with the goal of further improving the compounds’ fitness as LRRK2 degraders.

### Design, Synthesis, and Screening of Second-Generation LRRK2 PROTAC Degraders

**Figure 3:**
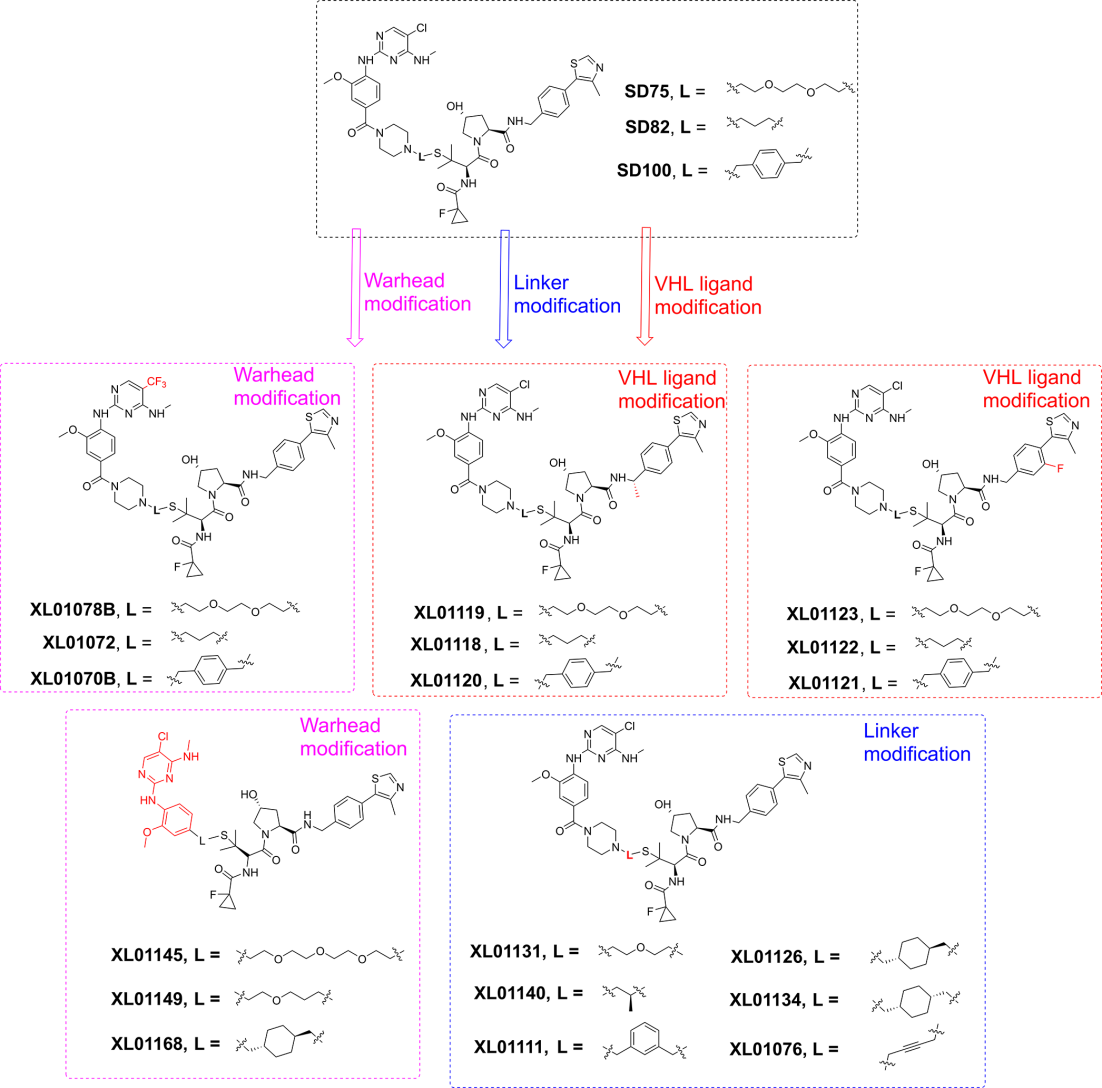
Second generation of LRRK2 PROTAC degraders derived from SD75, SD82, and SD100.

**Figure 4:**
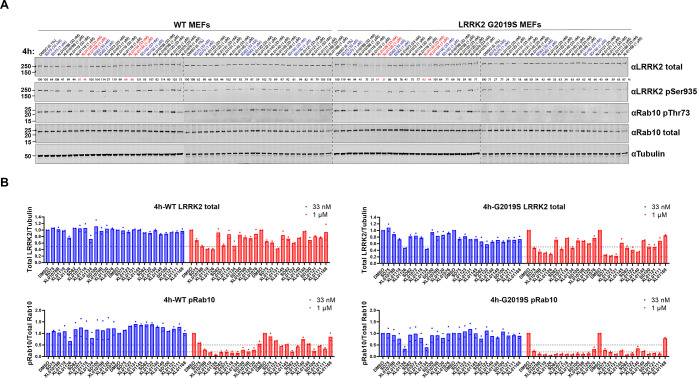
Screening of the second-generation PROTACs in WT and G2019S LRRK2 MEFs. (A) Representative Western blots monitoring the total LRRK2, LRRK2-pSer935, Rab10-pThr73, total Rab10, and tubulin levels after treating WT and G2019S MEFs with the indicated compounds at 33 nM, 1 μM, or DMSO for 4 h. (B) Quantitative analysis of the relative LRRK2 and Rab10-pThr73 levels, which are presented as ratios of total LRRK2/tubulin or Rab10-pThr73/total Rab10, normalized to the DMSO-treated sample. Data were obtained from two biological independent experiments.

**Scheme 1:**
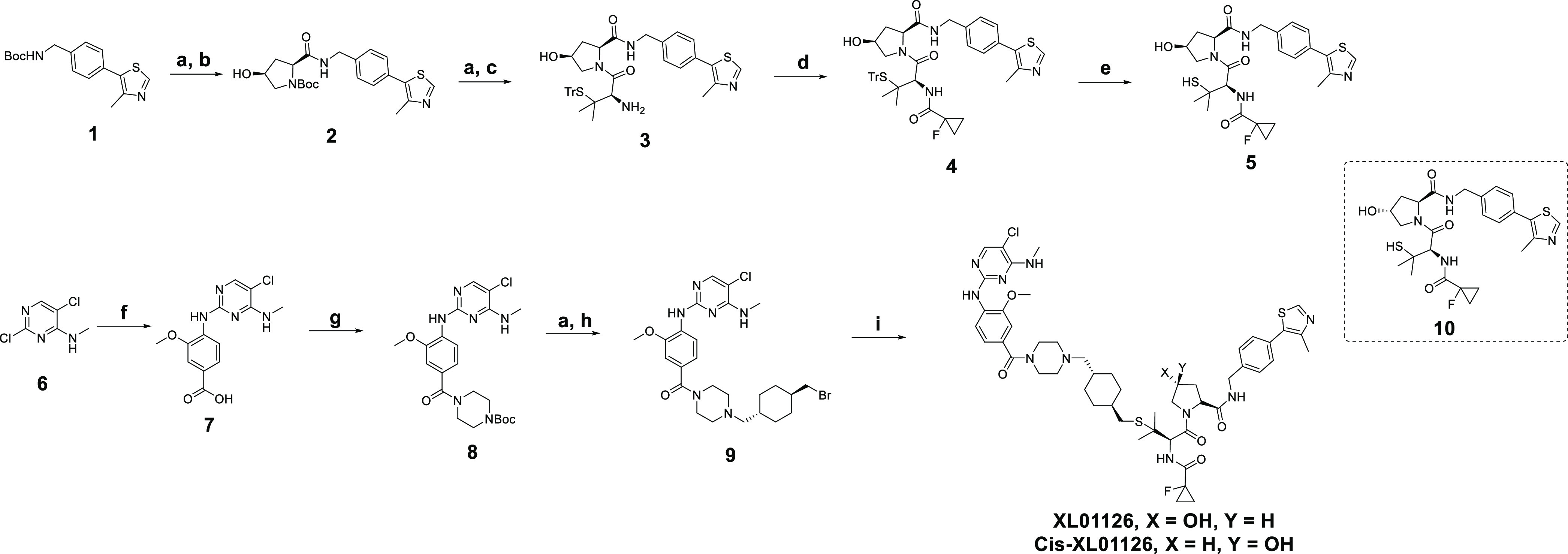
Synthesis of XL01126 and *cis*-XL01126 Reagents and conditions: (a) 2 N HCl in dioxane and dichloromethane (DCM) or DCM/methanol mix; (b) (2*S*,4*S*)-1-(*tert*-butoxycarbonyl)-4-hydroxypyrrolidine-2-carboxylic acid, 1-[bis(dimethylamino)methylene]-1*H*-1,2,3-triazolo[4,5-*b*]pyridinium 3-oxide hexafluorophosphate (HATU), triethylamine (TEA), DCM; (c) Fmoc-*S*-trityl-l-penicillamine, HATU, TEA, dimethylformamide (DMF); (d) 1-fluorocyclopropane-1-carboxylic acid, HATU, TEA, DCM; (e) trifluoroacetic acid (TFA), triisopropylsilane, DCM, 0 °C; (f) 4 N HCl in 1,4-dioxane, 4-amino-3-methoxybenzoic acid, water, 100 °C; (g) hydroxybenzotriazole (HOBt), 1-ethyl-3-(3-dimethylaminopropyl)carbodiimide (EDCI), *N*,*N*-diisopropylethylamine (DIPEA), 1-Boc-piperizine, DMF; (h) *trans*-1,4-bis(bromomethyl)cyclohexane, K_2_CO_3_, acetone, 50 °C; and (i) **5** or **10**, 1,8-diazabicyclo[5.4.0]undec-7-ene (DBU), tetrahydrofuran (THF).

Given the modular nature of PROTAC molecules, the structural modification of the second generation of LRRK2 PROTAC degraders focused on modifying the LRRK2 ligand, the linker, and the VHL ligand (Figures 1 and 3), separately. To best assess which structural modification would confer the most significant activity improvement, we designed molecular match pairs of SD75, SD82, and SD100 by changing one structural moiety at a time. XL01078B, XL01072, and XL01070B were designed (Figure 3) and synthesized (Scheme S3), where the 5-chlorine substitution on the aminopyrimidine ring of HG-10-102-01 was replaced with the −CF_3_ substitution, which was reported to improve the binding affinity to LRRK2.^51^ XL01119, XL01118, and XL01120 (Figure 3) were molecular match pairs of SD75, SD82, and SD100, respectively, by harboring an extra methyl group on the benzylic position of VHL ligand, which was introduced to increase the binding affinity to VHL E3 ligase.^52^ Fluorine substitution was introduced on the phenyl group of the VHL ligand of XL01123, XL01122, and XL01121 (Figure 3), attempting to fine-tune the physicochemical properties at a permissible site.^53,54^ The linker length, composition, and rigidity, which can significantly affect the physicochemical and pharmacokinetic (PK) properties of PROTACs, as well as their ternary complex formation and activity,^52,55,56^ were explored as represented by compounds XL01131, XL01140, XL01111, XL01126, XL01134, and XL01076 (Figure 3). In an attempt to improve the druglike properties and reduce the molecular size, we designed XL01145, XL01149, and XL01168 (Figure 3). These compounds are derived from truncated HG-10-102-01 with the morpholinoamide moiety removed as its absence retains binary binding affinity to LRRK2.^51^ These 18 new compounds were synthesized, as outlined in Schemes 1, S1–S3, and S6–S9, and were also screened *via* Western blotting (Figures 4 and S3). Quantitative analysis of the Western blots (Figures 4B and S3B) revealed that at 33 nM/4 h treatment, XL01126 and XL01134 were the most effective optimized compounds that degraded 20–30% of WT LRRK2 and 50–60% of G2019S LRRK2 (Figure 4B). Accordingly, these two compounds were also the most potent in decreasing pRab10 in both WT and G2019S LRRK2 MEFs, with >60% pRab10 inhibited in G2019S LRRK2 MEFs at 33 nM/4 h (Figure 4B). In contrast, the first-generation degraders SD75, SD82, and SD100 induced little to no degradation of LRRK2 at 33 nM/4 h treatment (Figure 4) and showed weak (<40%) degradation at 33 nM/24 h treatment (Figure S3), at which XL01126 and XL01134 degraded 50–60% of WT LRRK2 and 70–80% of LRRK2 G2019S (Figure S3). Most of the compounds exhibited substantial WT LRRK2 and G2019S LRRK2 degradation (30–80%) at 1 μM/4 h or 1 μM/24 h treatment (Figures 4 and S3), leading to potent and almost complete pRab10 inhibition in WT MEFs and G2019S LRRK2 MEFs, respectively. Multiple new compounds, including XL01078B, XL01119, XL01123, XL01131, XL01126, and XL01134, surpassed SD75, SD82, and SD100 in degrading WT LRRK2 and G2019S LRRK2 at 1 μM/4 h and 1 μM/24 h treatments, suggesting that modifications at the warhead (XL01078B), the E3 ligase ligand (XL01119 and XL01123), and the linkers (XL01131, XL01126, and XL01134) can all improve the degraders’ fitness to some extent. Nonetheless, the significant improvement exhibited by XL01126 and XL01134, which are isomers of each other, encouraged us to characterize them further.

### Identification of XL01126 as a Potent and Fast LRRK2 Degrader

**Figure 5:**
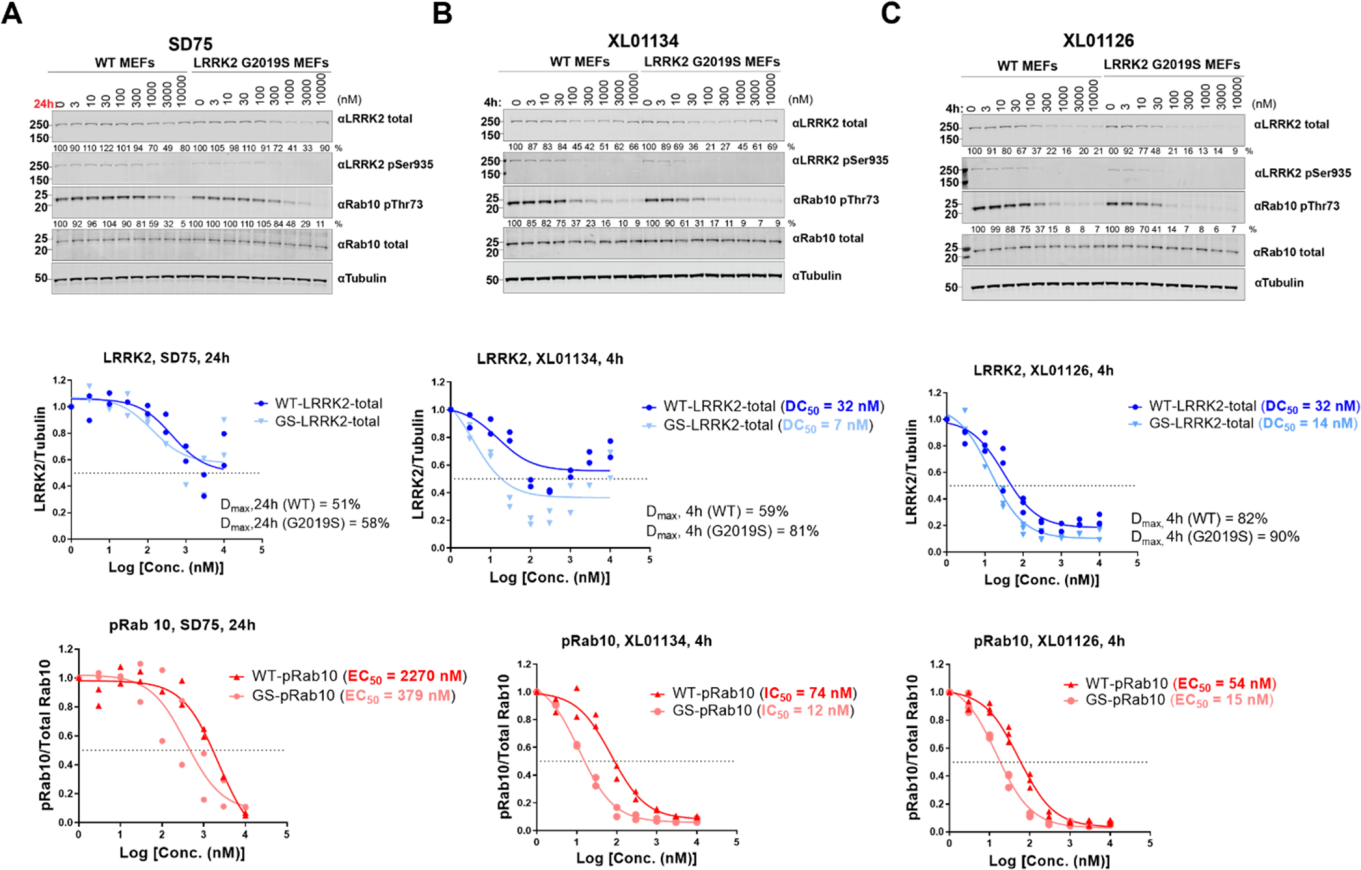
Dose-dependent LRRK2 degradation, LRRK2 dephosphorylation, and Rab10 dephosphorylation by SD75, XL01134, and XL01126 in WT and G2019S LRRK2 MEFs. Representative Western blots of total LRRK2, LRRK2-pSer935, Rab10-pThr73, total Rab10, and tubulin levels after treating WT and G2019S LRRK2 MEFs with SD75 (A), XL01134 (B), or XL01126 (C) at the indicated concentrations for the indicated time period. The relative LRRK2 protein and pRab10 levels were obtained by quantifying the ratios of total LRRK2/tubulin or Rab10-pThr73/total Rab10, respectively, and the ratios were normalized to the DMSO-treated samples. The relative total LRRK2 and pRab10 levels were plotted against the compound concentration and fitted against “nonlinear regression, one site-fit Log IC_50_” in GraphPad to obtain the DC_50_ and EC_50_ values. Data were obtained from two to three biological independent experiments.

To characterize XL01126 and XL01134, the top LRRK2 degraders from the second-generation compounds, and compare them with the top first-generation degrader SD75, a dose-dependent degradation assay was carried out in WT and G2019S LRRK2 MEFs (Figure 5). SD75 dose-dependently degraded LRRK2 following 24 h treatment in WT and G2019S LRRK2 MEFs (Figure 5A). However, the degradation of LRRK2 was only partial with *D*_max_ achieved at 3 μM (*D*_max,24h_ = 51 and 58% for WT and G2019S LRRK2, respectively). Dose-dependent LRRK2-pSer935 and pRab10 dephosphorylation, which account for both LRRK2 inhibition and degradation, were also observed after SD75 treatment, with EC_50_ = 2270 and 379 nM for the dephosphorylation of Rab10 in WT and G2019S LRRK2 MEFs, respectively. XL01134 and XL01126 showed more extensive LRRK2 degradation after a significantly shorter treatment time (4 h) when compared to SD75 (Figure 5B,C). XL01134 degraded G2019S LRRK2 (DC_50,4h_ = 7 nM) more potently than WT LRRK2 (DC_50,4h_ = 32 nM), with the maximum LRRK2 degradation achieved at 300 nM and *D*_max_ values against WT LRRK2 and G2019S LRRK2 are 59 and 81%, respectively. However, at concentrations above 300 nM, a strong “hook effect” was observed (Figure 5B). XL01126 also degraded G2019S LRRK2 (DC_50,4h_ = 14 nM) and WT LRRK2 (DC_50,4h_ = 32 nM) at nanomolar concentrations but achieved more complete degradation than XL01134, with *D*_max,4h_ = 82% in WT MEFs and *D*_max,4h_ = 90% in G2019S LRRK2 MEFs, achieved at around 1 μM. Moreover, no “hook effect” was observed with XL01126 at higher concentrations (Figure 5C). Due to the potent LRRK2 degradation capabilities, XL01134 and XL01126 resulted in more pronounced pRab10 dephosphorylation (Figure 5B,C) than SD75. XL01134, at 4 h, showed 30-fold more potent pRab10 inhibition than SD75 (at 24 h) in both WT MEFs and G2019S LRRK2 MEFs. XL01126 (at 4 h) is 40-fold more potent than SD75 (at 24 h) in inhibiting Rab10 phosphorylation in WT MEFs and 25-fold more potent in G2019S LRRK2 MEFs.

**Figure 6:**
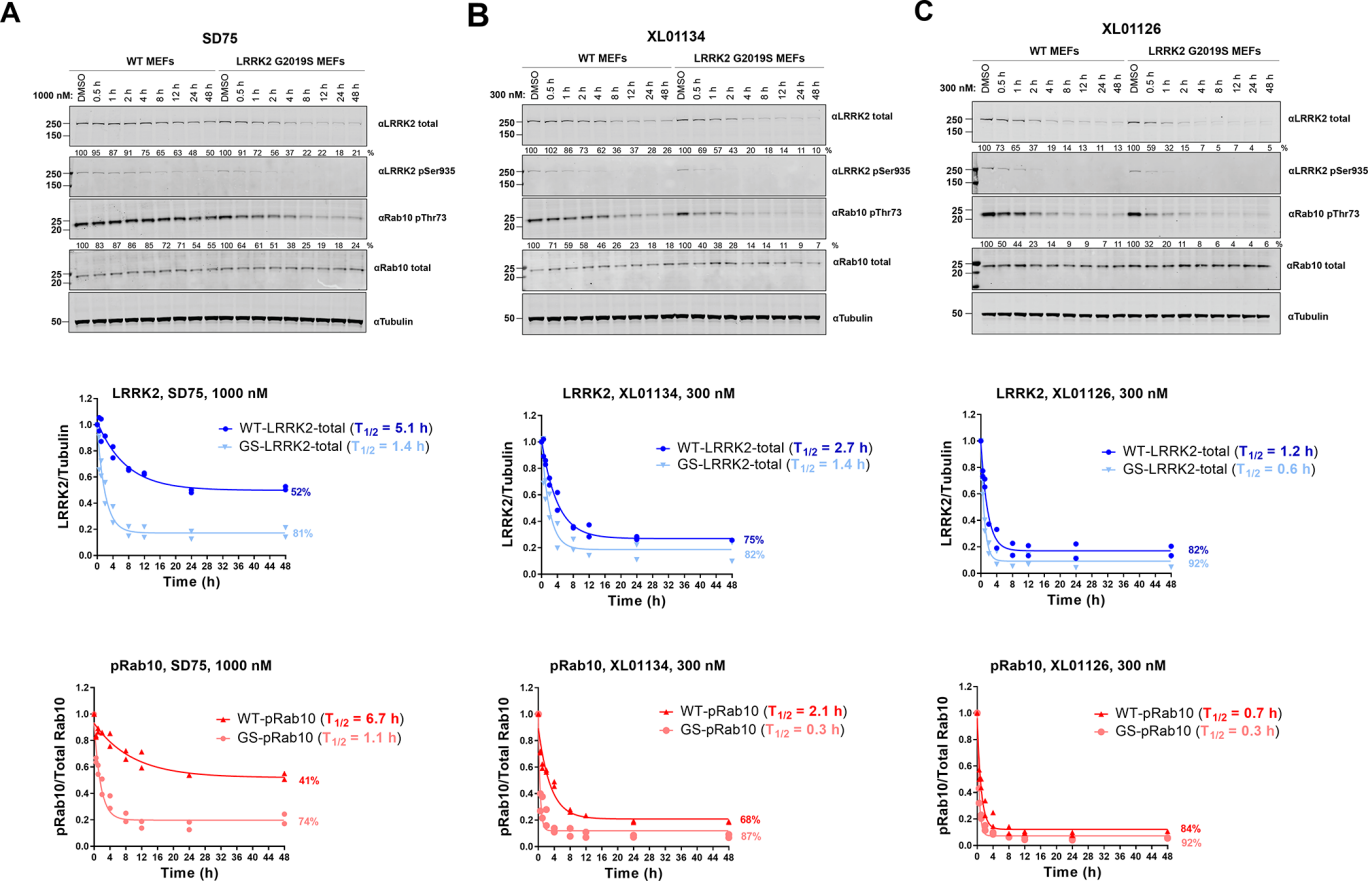
Time-dependent LRRK2 degradation, LRRK2 dephosphorylation, and pRab10 dephosphorylation by SD75, XL01134, and XL01126. Representative Western blots of total LRRK2, LRRK2-pSer935, Rab10-pThr73, Rab10 total, and tubulin levels after treating the WT and G2019S LRRK2 MEFs with SD75 (A), XL01134 (B), or XL01126 (C) at the indicated concentrations for the indicated period of time. The relative LRRK2 protein and pRab10 levels were obtained by quantifying the ratios of total LRRK2/tubulin or Rab10-pThr73/total Rab10, respectively, and the ratios were normalized to the DMSO-treated samples. The relative LRRK2 and pRab10 protein levels were plotted against the treatment time and were fitted against “nonlinear regression, one-phase decay” in GraphPad to obtain the half-life (*T*_1/2_) values. Data were obtained from two independent biological experiments.

To further compare the degradation profiles of XL01134 and XL01126 with that of SD75, a time-dependent degradation assay was performed in MEFs using Western blotting (Figure 6). SD75 was shown to degrade WT LRRK2 and G2019S LRRK2 at 1 μM in a time-dependent manner with moderate *D*_max_ (52% for WT LRRK2 and 81% for G2019S LRRK2) and half-lives (*T*_1/2_) against WT LRRK2 (5.1 h) and G2019S LRRK2 (1.4 h). In contrast, XL01134 and XL01126 degraded LRRK2 at higher rates and achieved higher *D*_max_ values at only 300 nM, a concentration at which SD75 barely degraded LRRK2. Remarkably, XL01126 presented an improved profile (*D*_max,WT_ = 82%, *D*_max,G2019S_ = 92%, *T*_1/2,WT_ = 1.2 h, *T*_1/2,G2019S_ = 0.6 h) when compared to XL01134 (*D*_max,WT_ = 75%, *D*_max,G2019S_ = 82%, *T*_1/2,WT_ = 2.7 h, *T*_1/2,G2019S_ = 1.4 h). With the shortest degradation half-lives and highest degradation percentage, XL01126 emerged as the most efficient and fastest degrader among the three. The time-dependent pRab10 dephosphorylation correlates well with the LRRK2 degradation (Figure 6A–C). XL01126 dephosphorylated pRab10 the fastest with *T*_1/2,pRab10_ at 0.7 and 0.3 h in WT and G2019S LRRK2 MEFs, respectively. This was followed by XL01134, which induced a 50% reduction in Rab10 phosphorylation after 2.1 and 0.3 h in WT and G2019S LRRK2 MEFs, respectively. In contrast, SD75 exhibited the slowest inhibition of pRab10 (*T*_1/2,pRab10_ = 6.7 h on WT MEFs and 1.1 h on G2019S LRRK2 MEFs).

**Figure 7:**
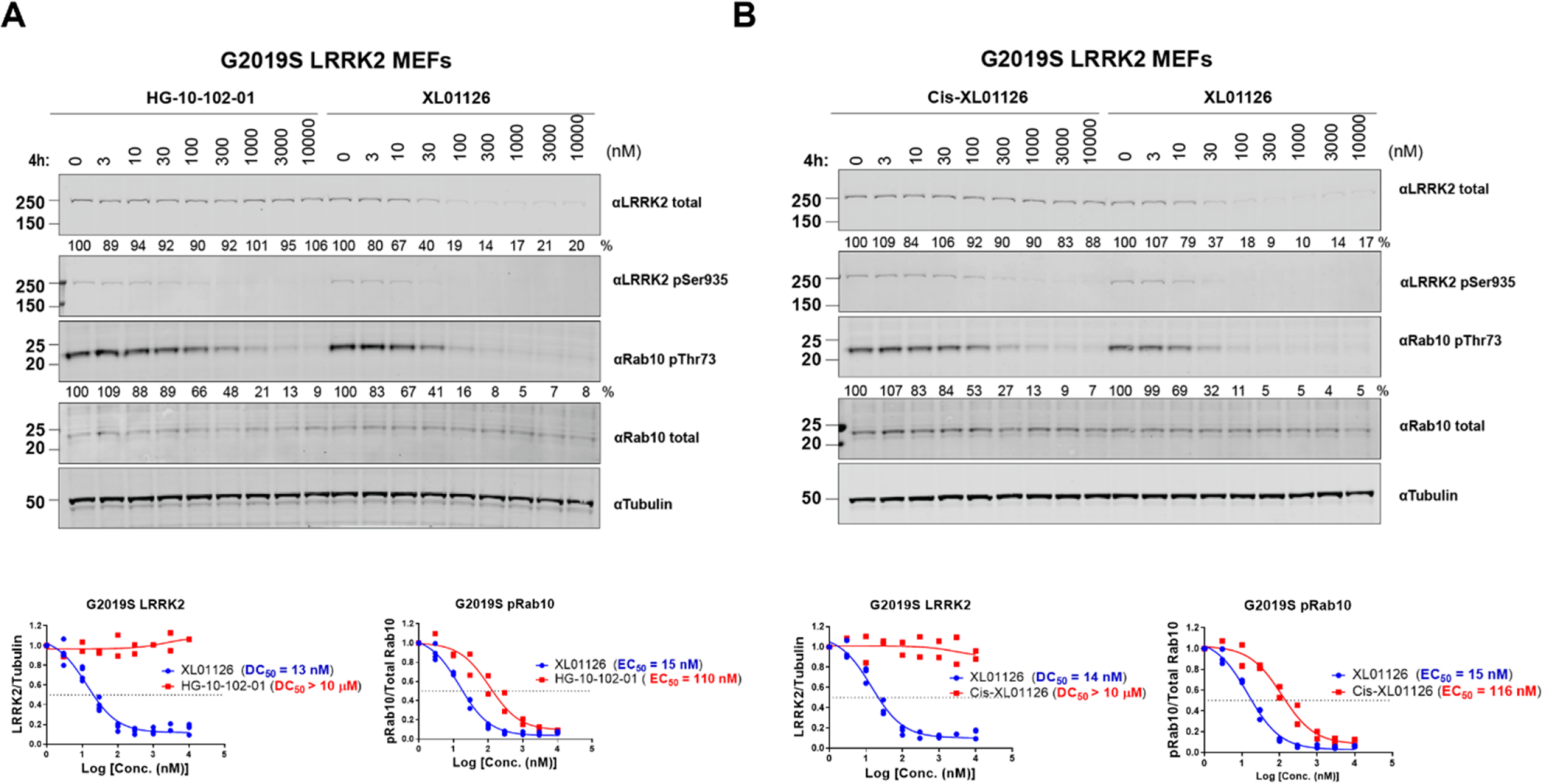
XL01126 surpassed its warhead and negative PROTAC *cis*-XL01126 in inhibiting downstream signaling in G2019S LRRK2 MEFs. Representative Western blots of total LRRK2, LRRK2-pSer935, pRab10, Rab10 total, and tubulin levels following the treatment of G2019S LRRK2 MEFs with HG-10-102-01 (A), XL01126 (A, B), and *cis*-XL01126 (B) at the indicated concentrations for 4 h. The relative LRRK2 protein and pRab10 levels were obtained by quantifying the ratios of total LRRK2/tubulin or Rab10-pThr73/total Rab10, respectively, and the ratios were normalized to the DMSO-treated samples. The relative LRRK2 and pRab10 protein levels were plotted against the compound concentration and fitted against “nonlinear regression, one site-fit log IC_50_” in GraphPad to obtain the DC_50_ and EC_50_ values. Data were obtained from two independent biological experiments.

The potent and fast degradation of LRRK2 and inhibition of the Rab10 substrate phosphorylation by XL01126 prompted us to test if our PROTAC could surpass its warhead (HG-10-102-01) in dephosphorylating the substrate of LRRK2. This would allow us to ask how much of the substrate dephosphorylation activity of XL01126 is due to the degradation of LRRK2, and how much could be due to enzyme inhibition. This question is of particular relevance for this project because the warhead ligand itself is a strong LRRK2 inhibitor with nanomolar kinase inhibition activities (Figure S4)^17^ and is a general challenge with PROTACs against protein kinases. As expected, the warhead HG-10-102-01 did not degrade LRRK2 but potently inhibited LRRK2 phosphorylation and Rab10 phosphorylation (EC_50_ = 110 nM on G2019S LRRK2 MEFs, EC_50_ = 214 nM on WT MEFs) (Figures 7 and S5). In contrast, XL01126 dose-dependently degraded both WT LRRK2 (Figure S5) and G2019S LRRK2 (Figure 7A). Crucially, XL01126 showed around 3-fold more potent inhibition of Rab10 phosphorylation in WT MEFs than HG-10-102-01 (Figure S5A) and 6-fold in G2019S LRRK2 MEFs (Figure 7A). These observations suggest that converting HG-10-102-01 to a PROTAC degrader not only improves downstream signaling inhibition but also increases the selectivity for G2019S LRRK2 over WT. *Cis*-XL01126 (Scheme 1), a non-degrading distomer control of XL01126 where the stereochemistry at the hydroxyl group of hydroxyproline is inverted to abrogate VHL binding,^57^ showed no degradation of WT LRRK2 (Figure S5B) and G2019S LRRK2 (Figure 7B) but inhibited Rab10 phosphorylation at a similar potency as HG-10-102-01 in both WT MEFs (Figure S5) and G2019S LRRK2 MEFs (Figure 7). However, due to the lack of LRRK2 degradation, *cis*-XL01126 was around 7-fold less potent than XL01126 in inhibiting Rab10 phosphorylation (116 *vs* 15 nM), further demonstrating the potency boost in downstream functionality achieved from LRRK2 degradation over and above the kinase inhibition.

**Figure 8:**
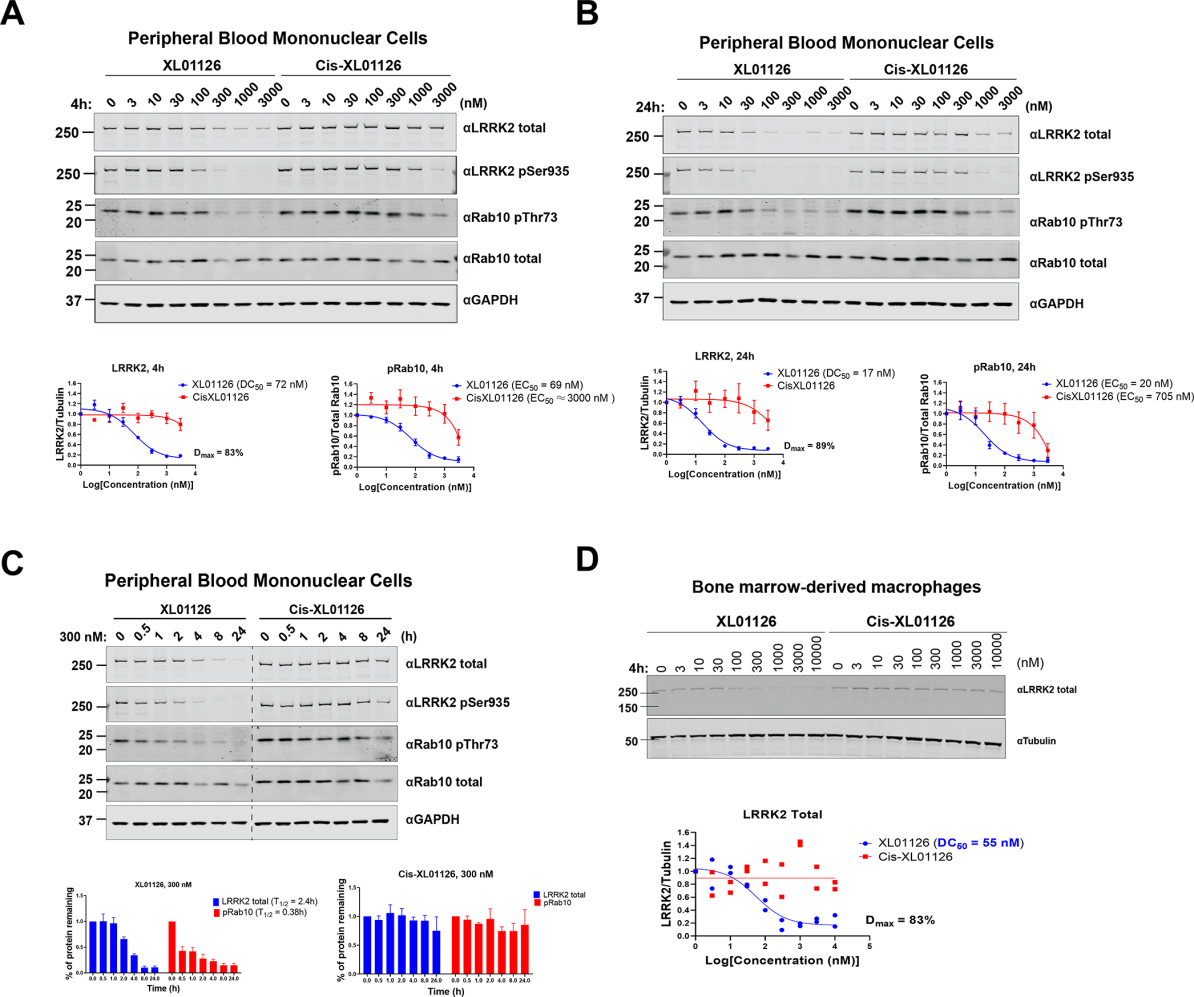
XL01126 degrades LRRK2 in human peripheral blood mononuclear cells (PBMCs) derived from healthy donors and mouse bone marrow-derived macrophages (BMDMs). Representative Western blotting of total LRRK2, LRRK2-pSer935, pRab10, Rab10 total, and GAPDH levels following treating the PBMCs with XL01126 and *cis*-XL01126 at the indicated concentrations for 4 h (A) and 24 h (B). The relative LRRK2 protein and pRab10 levels were obtained by quantifying the ratios of total LRRK2/GAPDH or Rab10-pThr73/total Rab10, respectively, and the ratios were normalized to the DMSO-treated samples. The relative LRRK2 and pRab10 protein levels were plotted against the compound concentration and fitted against “nonlinear regression, one site-fit log IC_50_” in GraphPad to obtain the DC_50_ and EC_50_ values. Data points are presented as mean ± standard error of the mean (SEM) from three biological independent replicates. (C) Representative Western blotting of total LRRK2, LRRK2-pSer935, pRab10, Rab10 total, and GAPDH levels following treating the PBMCs with 300 nM of XL01126 and *cis*-XL01126 for the indicated time periods. The relative LRRK2 protein and pRab10 levels were obtained by quantifying the ratios of total LRRK2/ GAPDH or Rab10-pThr73/total Rab10, respectively, and the ratios were normalized to the DMSO-treated samples. The relative LRRK2 and pRab10 protein levels were plotted against the treatment time and were fitted against “nonlinear regression, one-phase decay” in GraphPad to obtain the half-life (*T*_1/2_) values. Data points are presented as mean ± SEM from three biological independent replicates. (D) Representative Western blotting of LRRK2 total and tubulin levels after treating BMDMs with XL01126 and *cis*-XL01126 for 4 h. The relative LRRK2 levels were obtained by quantifying the ratios of total LRRK2/tubulin and normalized to the DMSO-treated samples. The relative LRRK2 levels were plotted against the compound concentration and fitted against “nonlinear regression, one site-fit log IC_50_” in GraphPad to obtain the DC_50_ values.

To scope and assess the degradation activity of XL01126 on other LRRK2 mutants and cell lines, dose-dependent degradation assays of XL01126 were carried out in R1441C LRRK2 MEFs (Figure S6), bone marrow-derived macrophages (BMDMs), and human peripheral blood mononuclear cells (PBMCs) (Figure 8). XL01126 exhibited potent LRRK2 degradation in all of these cell types, with a significant differentiation observed between XL01126 and *cis*-XL01126 in terms of Rab10 dephosphorylation (Table 1 and Figures S6 and 8). The fast (*T*_1/2,300nM_ = 2.4 h) and potent (DC_50,4h_ = 72 nM, DC_50,24h_ = 17 nM) degradation of human LRRK2 in PBMCs suggest the potential of applying XL01126 to additional human cell lines. Testing of XL01126 and *cis*-XL01126 on SH-SY5Y, a human neuroblastoma cell line widely used as PD cell model,^58^ revealed that XL01126 induced 50% or more degradation of LRRK2 after 6 h/300 nM or 24 h/300 nM treatment (Figure S7).

**Table 1:** Summary of the Degradation Activities of XL01126 and *cis*-XL01126 on R1441C LRRK2 MEFs, BMDMs, and PBMCs

	R1441C LRRK2 MEFs^a^		BMDMs^b^		PBMCs^c^	
	XL01126	*cis*-XL01126	XL01126	*cis*-XL01126	XL01126	*cis*-XL01126
DC_50_s (LRRK2)	15 nM (4 h)	NDO	55 nM (4 h)	NDO	72 nM (4 h)	NDO
					17 nM (24 h)	
*D*_max_ (LRRK2)	89% (4 h)	NDO	83% (4 h)	NDO	83% (4 h)	NDO
					89% (24 h)	
EC_50_s (pRab10)	30 nM (4 h)	158 nM (4 h)			69 nM (4 h)	3000 nM (4 h)
					20 nM (24 h)	705 nM (24 h)
*T*_1/2_^d^		NDO^e^			2.4 h	

a R1441C LRRK2 mutant mouse embryonic fibroblasts (MEFs).

b Bone marrow-derived macrophages (BMDMs).

c Peripheral blood mononuclear cells (PBMCs).

d Degradation half-life of LRRK2.

e NDO, no significant degradation of LRRK2 observed.

### XL01126 Induces Cooperative Ternary Complex Formation

**Figure 9:**
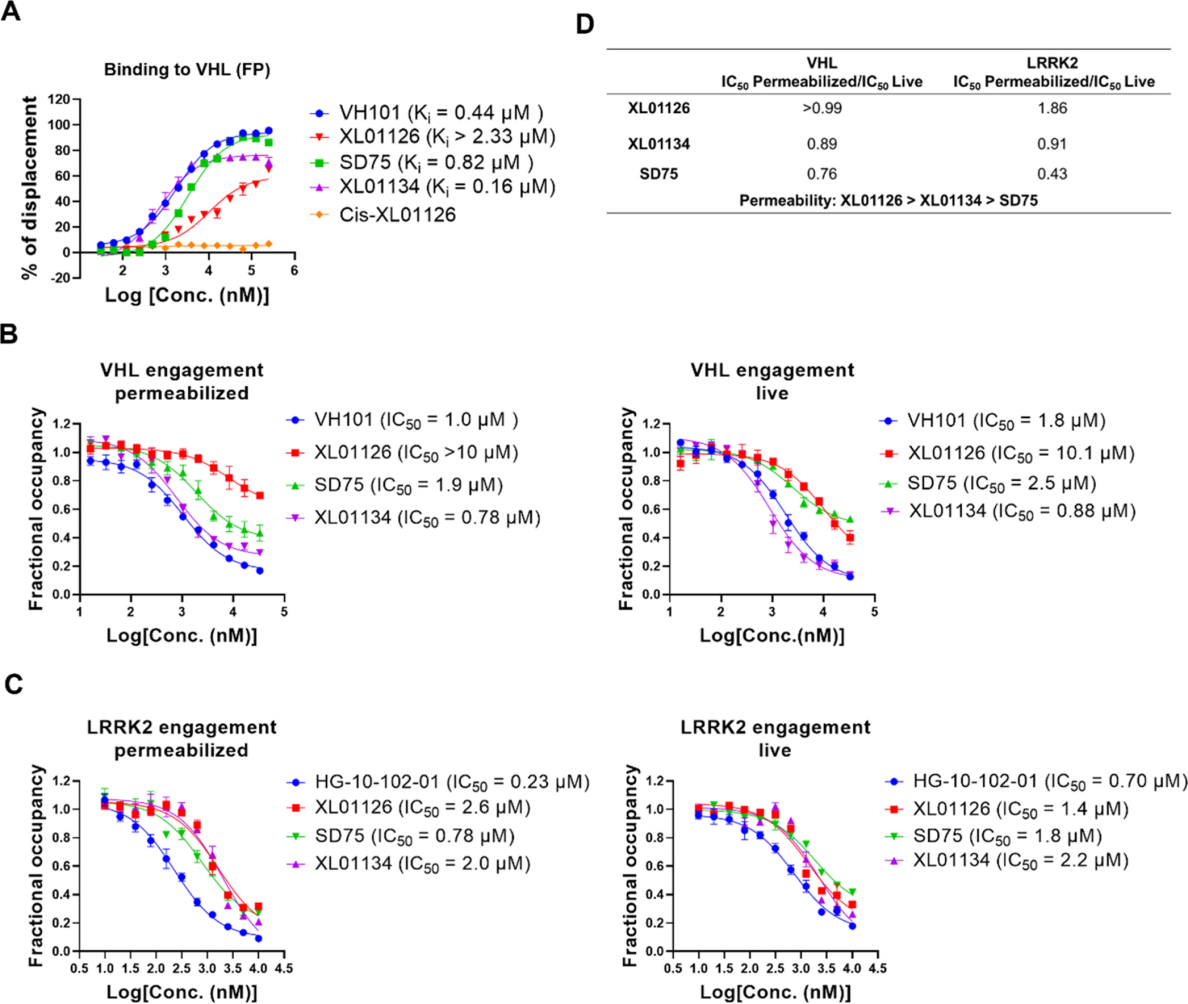
Binding affinities to VHL and LRRK2. (A) Binding affinity of the tested compounds to VHL using the FP assay. The indicated compounds were titrated to a solution of VCB protein (10 nM) and JC9 (5 nM) (a FAM-labeled probe that binds to VCB) to displace JC9, and the percentage of displacement was plotted against the compounds’ concentration and fitted into the “nonlinear regression, one site-log IC_50_” to obtain the IC_50_ values, which were used to back-calculate the *K*_i_ values. NanoBRET target engagement assays of tested compounds to VHL (B) and LRRK2 (C) in permeabilized and live-cell modes. The indicated compounds were titrated into HEK293 cells transfected with VHL-NanoLuc (B) or LRRK2-NanoLuc fusion (C) in the presence of VHL tracer (B) or LRRK2 tracer (C). 0.25 and 0.5 μM VHL tracers were used for the permeabilized and live mode VHL engagement assays separately. 0.125 and 0.5 μM of LRRK2 tracer were used for the permeabilized and live mode LRRK2 engagement assays separately. The fractional occupancy of the tracers is plotted against the tested compounds’ concentrations and fitted into “nonlinear regression, one site-log IC_50_” to obtain the IC_50_ values of each compound against both permeabilized and live cells, separately. Data points are presented as mean ± SEM from three independent experiments. (D) The IC_50_ ratios between permeabilized and live mode target engagements of each compound were used to compare their permeabilities.

**Figure 10:**
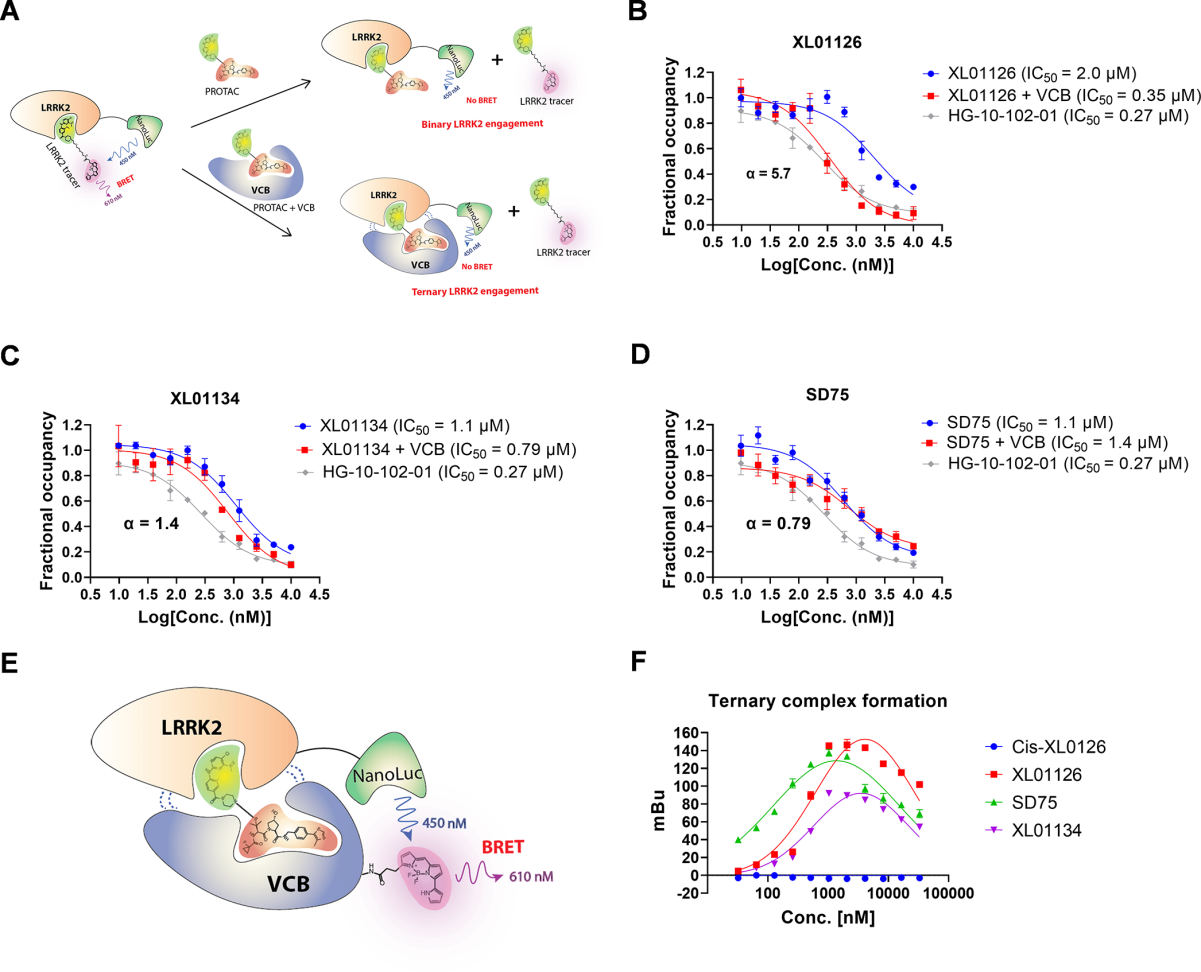
Binary/ternary binding affinity, cooperativity, and ternary complex formation of XL01126, XL01134, and SD75. (A). Schematic illustration of the binary and ternary LRRK2 engagement assays. HG-10-102-01, XL01126 (B), XL01134 (C), and SD75 (D) were titrated into the lysate of HEK293 cells (transfected LRRK2-NanoLuc) alone (blue line) or preincubated with VCB (red line) in the presence of LRRK2 tracer. The fractional occupancy of the tracer is plotted against the concentrations of the compounds and fitted into the “nonlinear regression, one site-log IC_50_” model in GraphPad to obtain the IC_50_ values. The IC_50_ ratio between the blue curve and red cure is calculated as cooperativity (α). (E) Schematic illustration of the ternary complex formation assay (F). *cis*-XL01126, XL01126, SD75, and XL01134 were titrated into the lysate of HEK293 cells (transfected with LRRK2-NanoLuc) and 0.5 μM VCB protein labeled with Bodipy^576/589^. The NanoBRET signal was plotted against the compounds’ concentrations and fitted into “nonlinear regression, Gaussian” model in GraphPad. Error bars are mean ± SEM from three biological independent experiments.

As the top two degraders from the second generation, XL01126 and XL01134 are epimers of each other, the only difference being swapped chirality at one of the two tertiary carbons of the cyclohexyl ring in their linkers. This small difference in the chemical structure gives rise to very different degradation profiles for the two compounds (Figures 5 and 6). These two epimeric PROTACs also exhibited strikingly different binding affinities to VHL, as revealed by a fluorescence polarization (FP) displacement binding assay (Figure 9A)^50,59^ and a VHL target engagement assay (Figure 9B).^60^ XL01126 has >10-fold weaker binary binding to VHL than XL01134 and also was found to be the weakest LRRK2 binder among the compounds tested (Figure 9C). PROTACs have previously been shown to tolerate weakened binary binding affinities to either their E3 ligase^61,62^ or target protein^63,64^ such that, despite the weak binding, they are able to induce potent protein degradation at a concentration well below the *K*_d_. Conversely, PROTACs made of more potent target ligands do not necessarily guarantee for more potent degraders.^56,63^ These studies together illustrated a now well-established feature with PROTACs, that is, the extent of target degradation does not necessarily correlate with the PROTAC’s binary binding affinity to E3 ligase or target protein. The ternary binding affinity, cooperativity, and stability of the ternary complex can instead play critically important roles in PROTAC-induced protein degradation.^65−68^ To test whether our PROTACs can induce a cooperative ternary complex formation and illuminate the relationship between the degradation potency and ternary complex formation, a ternary binding affinity assay and a ternary complex formation assay are warranted. However, we could not implement the most commonly used biophysical techniques such as fluorescence polarization^59^ and surface plasma resonance^69^ for these assays due to the lack of sufficient recombinant expressed LRRK2 in hand. We therefore turned to endogenously expressed LRRK2 and developed a NanoBRET-based ternary binding affinity assay and ternary complex formation assay in HEK293 cells (Figure 10).

In the NanoBRET-based ternary binding affinity assay, a LRRK2-NanoLuc fusion was transiently expressed in HEK293 cells as the BRET donor, while a LRRK2 tracer prepared by conjugating HG-10-102-01 with a fluorophore (BODIPY^576/589^) (Figure 10A and Scheme S11) was introduced as the acceptor. Titration of PROTACs to the lysed cells and LRRK2 tracer in the presence or absence of recombinant VCB protein (VHL complexed with elongin B–elongin C) gives ternary and binary binding affinities of PROTACs for LRRK2, respectively. Similarly, the ternary complex formation assay also used LRRK2-NanoLuc transiently expressed in HEK293 as the BRET donor, but the acceptor was recombinant VCB protein labeled with BODIPY^576/589^*via* the NHS ester-activated cross-linking reaction. PROTACs that can bridge LRRK2 and VCB together will produce a BRET signal (Figure 10E).

In line with the degradation potency, XL01126 induced the most cooperative ternary complex as indicated by its positive cooperativity (α = 5.7) (Figure 10B) and the highest maximal level of ternary complex formation (Figure 10F). In contrast, XL01134 induced significantly lower cooperativity (α = 1.4) and SD75 has a negative cooperativity with VHL and LRRK2 (Figure 10).

In the NanoBRET-based ternary complex formation assay, SD75, although a less potent degrader than XL01134, induced a greater level of ternary complex than XL01134. However, it should be noted that this assay was carried out in the permeabilized HEK293 cells, and SD75 is likely to induce less intracellular ternary complex formation given its relatively lower permeability compared to XL01134 (Figure 9D). The relative permeability (intracellular availability) of each compound was obtained by querying VHL engagement or LRRK2 engagement under live-cell and permeabilized-cell conditions^60,70^ (Figure 9).

### XL01126-Induced LRRK2 Degradation Is Selective and Dependent on the Ubiquitin–Proteasome System

**Figure 11:**
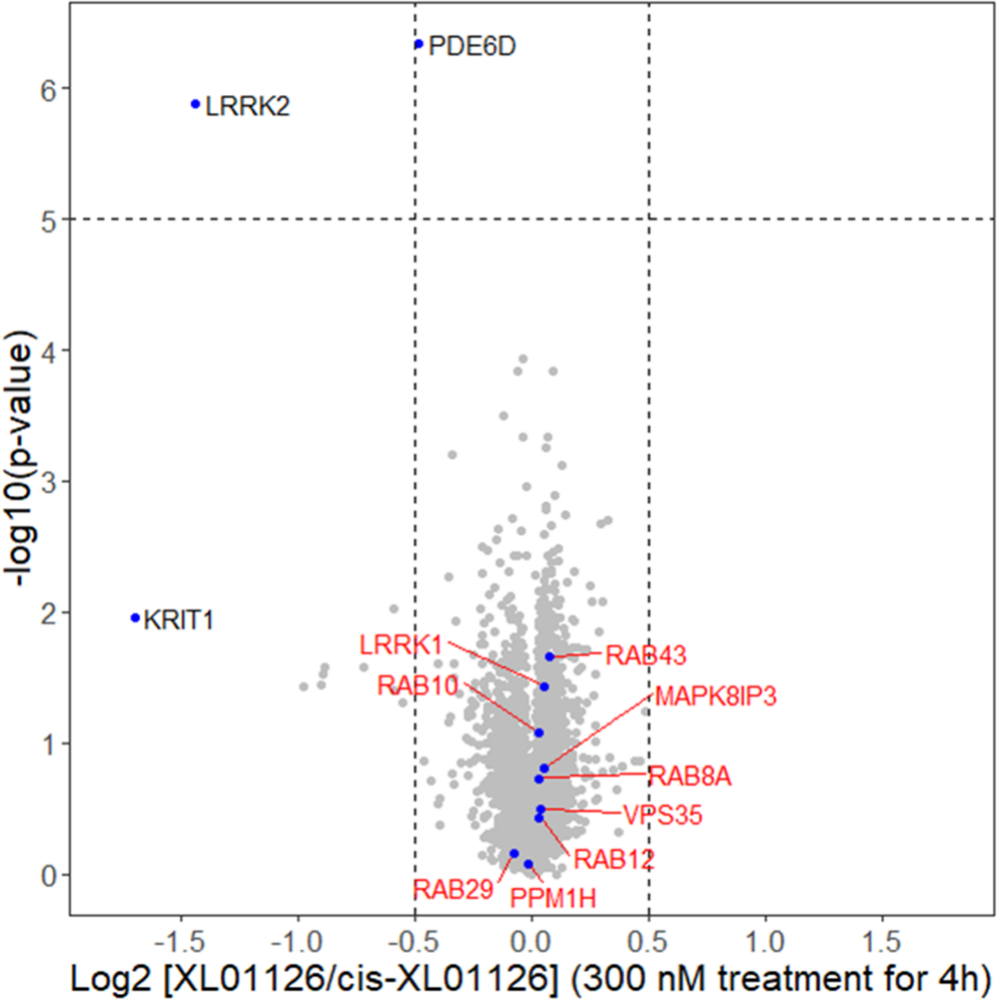
XL01126 induced selective LRRK2 degradation. WT MEFs were treated with 300 nM XL01126, *cis*-XL01126, or DMSO for 4 h and lysed (three to four replicates per condition). The lysate samples were analyzed with quantitative proteomics. Data plotted log 2 of the fold change *vscis*-XL01126 control against −log10 of the *P* value per protein. Both XL01126- and *cis*-XL01126-treated samples were normalized to DMSO samples before taking the ratio.

To assess the degradation selectivity of XL01126 and identify potential off-targets at the proteome level, we performed unbiased quantitative tandem mass tag (TMT)-based global proteomic profiling in WT MEFs. Over 8000 proteins were quantified in the cell lysate samples from WT MEFs that were treated with 300 nM XL01126, *cis*-XL01126, or DMSO for 4 h (Figure 11). The data corroborate a significant chemical knockdown of LRRK2, as validated by Western blotting (Figure S8). LRRK1, the closest homologue of LRRK2, and other LRRK2-related proteins such as VPS35 and Rab-specific phosphatase PPM1H remained unaffected. The proteomic data also revealed a small (∼30%) depletion in protein levels of phosphodiesterase 6δ (PDE6D) (Figure 11). PDE6D has a deep hydrophobic ligand-binding pocket and has been shown to be degradable *via* PROTACs.^44,71^ Curiously, PDE6D was also found as the adventitious off-target degradation of PTK2 PROTACs previously.^72^ Inspection of chemical structures highlighted that the PTK2 PROTAC and XL01126 share a similar aminopyrimidine warhead at the target ligand end, a moiety known to be critical to the high binding affinity in PDE6D inhibitor deltasonamide,^71^ suggesting a potential off-target degradation due to adventitious PROTAC binding to PDE6D. Dose-dependent degradation of PDE6D in both WT MEFs and LRRK2 KO MEFs as shown *via* Western blotting (Figure S8) indicated that XL01126-induced PDE6D degradation is LRRK2-independent and excluded it being a downstream consequence of LRRK2 degradation.

**Figure 12:**
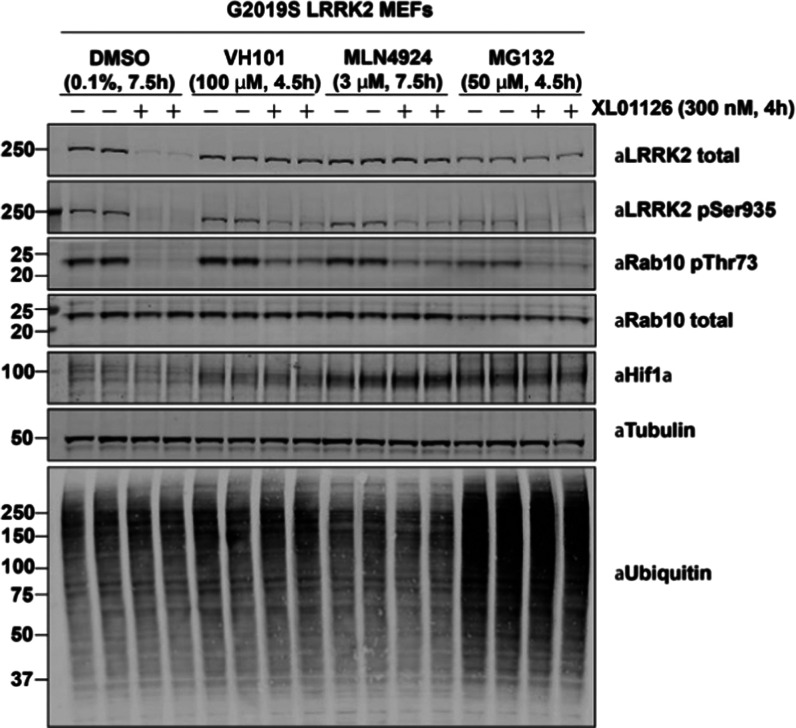
XL01126-induced LRRK2 degradation is rescued by VH101, MLN4924, and MG132 pretreatments. Representative Western blots of total LRRK2, LRRK2-pSer935, pRab10, total Rab10, HIF-1α, tubulin, and ubiquitinated protein after treating the G2019S LRRK2 MEFs with 300 nM XL01126 for 4 h with or without VH101, MLN4924, and MG132 pretreatments.

A study examining the mechanism of LRRK2 degradation demonstrated that degradation by XL01126 is mediated by the ubiquitin–proteasome system as XL01126-induced degradation can be blocked by VHL ligand (VH101), neddylation inhibitor (MLN4924), and proteasome inhibitor (MG132) pretreatments in both WT MEFs (Figure S9) and G2019S LRRK2 MEFs (Figure 12). However, the LRRK2 dephosphorylation and Rab10 dephosphorylation are not completely rescued by VH101, MLN4924, and MG132 pretreatments owing to the kinase inhibition effect of XL01126, as also evidenced in our kinase inhibition assay (Figure S4).

### XL01126 Increases Mitophagy in Immortalized Mouse Embryonic Fibroblast Cells

**Figure 13:**
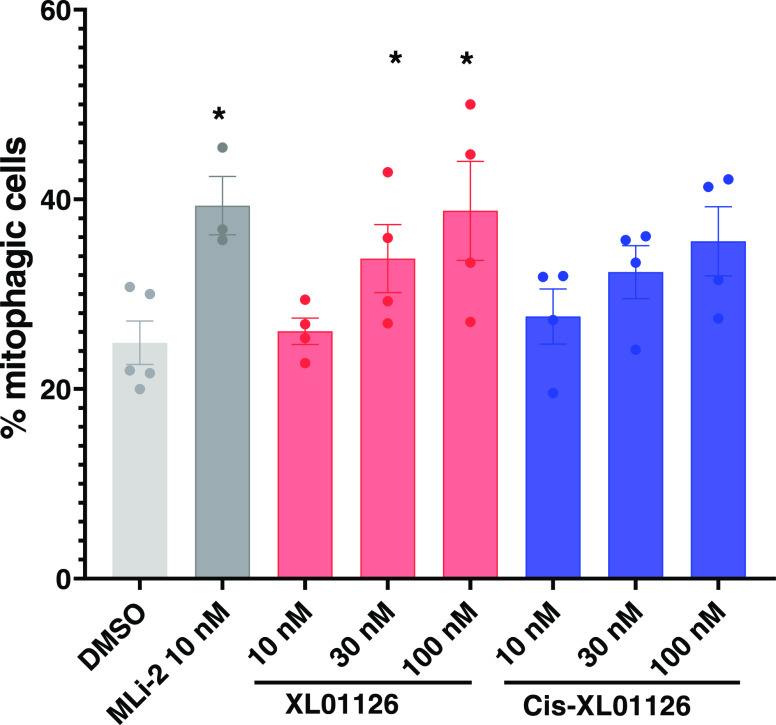
Effects of XL01126 and *cis*-XL01126 on mitophagy in Mito-QC MEFs. Quantitation of the percentage of mitophagic cells in mito-QC MEFs after 24 h treatment with DMSO, MLi-2, XL01126, or *cis*-XL01126 at the indicated concentrations. Data are represented as mean ± SEM from three to five independent experiments. Statistical significance is displayed as *p < 0.05 compared with the DMSO-treated sample.

With a potent, fast, and selective LRRK2 degrader in hand, we next established the XL01126 cellular functionality in bioassays that report on LRRK2 activity. Mitochondrial dysfunction is one of the pathophysiological hallmarks of PD^73^ and can be rescued by mitophagy, a quality control mechanism whereby damaged or unnecessary mitochondria are delivered to lysosomes for degradation through membrane trafficking.^8^ It has been shown that increasing mitophagy with inducer agents has the potential as a PD therapy.^74^ Previous studies have shown that the LRRK2 kinase activity impairs basal mitophagy and that LRRK2 knockout or pharmacological inhibition of LRRK2 with kinase inhibitors was able to rescue the mitophagy level.^8^ Utilizing XL01126 as a chemical degrader tool and using *cis*-XL01126 as a nondegrader, kinase inhibitor control, we found that both XL01126 and *cis*-XL01126 induced the mitophagy level dose-dependently (Figure 13) in mito-QC MEFs, an mCherry–GFP–mitochondria reporter cell model developed previously.^75^ Although XL01126 and *cis*-XL01126 act on LRRK2 through different mechanisms, they shared similar potency in inducing mitophagy at 10–100 nM, indicating that the mitophagy level is indeed LRRK2 kinase-dependent and that other domains or motifs of LRRK2 are not involved in regulating mitophagy.

### XL01126 Is Orally Bioavailable and Can Penetrate Blood–Brain Barrier

**Figure 14:**
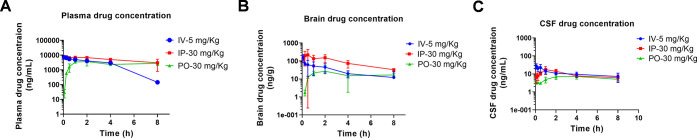
Plasma, brain, and cerebrospinal fluid (CSF) concentrations of XL01126 following a single dose of XL01126 *via* IV, IP, and PO. Male C57BL/6 mice were treated with a single dose of XL01126 by either IV (5 mg/mL), IP (30 mg/kg), or PO (30 mg/kg) injection, and the concentrations of XL01126 in blood plasma (A), brain tissue (B), and CSF (C) were measured at seven time points. Data are mean (±standard deviation (SD)) from three mice at each time point.

To qualify XL01126 as both cellular and *in vivo* suitable degrader probes and to assess its drug development potential, we next evaluated the physicochemical and absorption, distribution, metabolism, and excretion (ADME) properties (Table 2 and Figure S10), as well as the *in vivo* pharmacokinetic profiles of XL01126 (Figure 14). Due to the high molecular weight and lipophilicity, XL01126 has low solubility in phosphate-buffered saline (PBS) and moderate solubility in Fed State Simulated Intestinal Fluid (FeSSIF) (Table 2), which, however, are all well above its DC_50_ values (14–72 nM). The high stability (half-life at 108.29 min) of XL01126 in mouse plasma indicates that XL01126 might be suitable for *in vivo* studies and we reasoned that plasma protein binding may account for its stability as protein binding can decrease the amount of free compound available for enzymatic metabolism. The protein binding also affects the potency of XL01126 in cells, as shown by the significant potency shift of XL01126 in MEFs in the presence and absence of 10% fetal bovine serum (FBS) in the culture media (Figure S11).

**Table 2:**
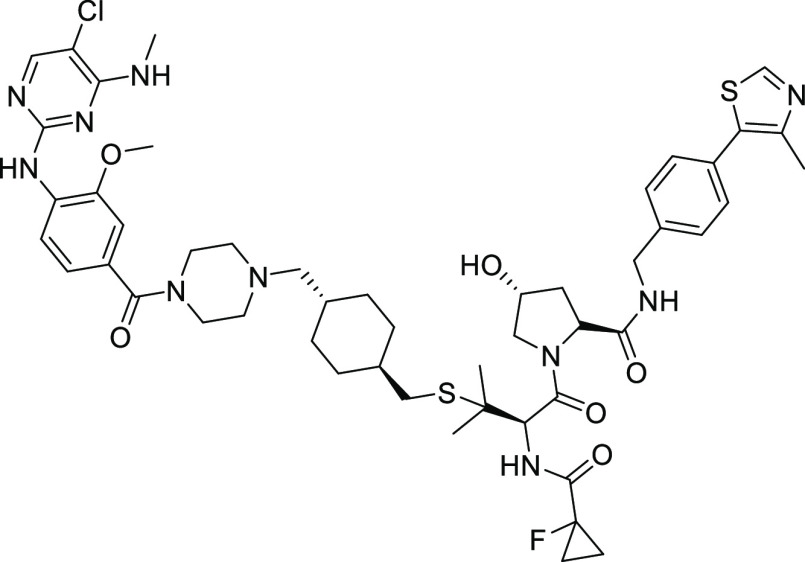
*In Vitro* Physicochemical and ADME Properties of XL01126

XL01126	
molecular weight	1019.7
CLog *D*	4.44^a^
hydrogen bond acceptor	16^a^
hydrogen bond donor	5
total polar surface area	194.3^a^
solubilities in PBS (pH 7.4)	0.55 μM
solubility in FeSSIF (pH 5.8)	26.05 μM
Caco-2 permeability	A–B < 0.74 × 10^–6^ cm/s
	B–A < 1.43 × 10^–6^ cm/s
*T*_1/2_ in mouse plasma	108.29 min
*T*_1/2_ in mouse liver microsome	3.65 min
Cl_int_ in mouse liver microsome	1494.62 mL/min/kg
*T*_1/2_ in mouse hepatocytes	314.33 min
Cl_int_ in mouse hepatocytes	26.04 mL/min/kg

a Values are calculated with Stardrop.

To further qualify XL01126 as appropriate for *in vivo* studies, we assessed its PK profiles in mice (Figure 14 and Table 3). Following a single dose of XL01126 *via* intravenous (IV, 5 mg/kg), intraperitoneal (IP, 30 mg/kg), and oral gavage (PO, 30 mg/kg), the concentrations of XL01126 in plasma, brain tissue, and cerebrospinal fluid (CSF) were determined. XL01126 showed fast absorption in both IP and PO injections with *C*_max_ (7700 and 3620 ng/mL for IP and PO separately) reached at 0.25 min and 2 h for IP and PO dosing, respectively. High plasma concentrations were achieved in all routes of administration and were maintained at levels way above the DC_50_ values for XL01126 in the experimental time period. The metabolism of XL01126 seems slow in all administration routes, probably because of high protein binding. Strikingly, XL01126 was also detected in brain tissues and CSF (Figure 14B,C), suggesting that XL01126 is capable of penetrating the BBB regardless of its unfavorable *in vitro* ADME properties and violation of Ro5 and/or RoCNS.^76^ To the best of our knowledge, this is the first-time report of a VHL-based PROTAC that is both orally bioavailable (*F* = 15%) and BBB-permeable. Further investigation of XL01126 will focus on its in *vivo* pharmacodynamics and PD-related functional studies.

**Table 3:** Pharmacokinetic (PK) Parameters of XL01126 Following a Single Dose of XL01126 *via* IV, IP, and PO

plasma PK properties	CL (L/h/kg)	*V*_ss_ (L/kg)	*T*_max_ (h)	*C*_max_ (ng/mL)	*T*_1/2_ (h)	AUC_last_ (h ng/mL)	AUC_inf_ (h ng/mL)	MRT (h)	*F* (%)
IV^a^ (5 mg/kg)	0.208	0.511			1.52	23 663	23 981	2.45	
IP^b^ (30 mg/kg)			0.25	7700	5.2	41 434	64 068		29.2
PO^c^ (30 mg/kg)			2	3620	21.9	21 337	109 271		15

a Intravenous.

b Intraperitoneal.

c Peroral. CL, clearance; *V*_ss_, volume of distribution; *T*_max_, the time the compound takes to reach the maximum plasma concentration; *C*_max_, the maximum plasma concentration a compound reached after dosing; AUC, area under the curve; MRT, mean resident time; and *F*, bioavailability.

## Conclusions and Discussion

In summary, we discover and characterize a fast, potent, selective, cooperative, orally bioavailable, and BBB-permeable LRRK2 PROTAC degrader, XL01126, through medicinal chemistry exploration and pharmacological evaluation.

Although LRRK2 is a sought-after target for PD, the exact signaling pathways that link LRRK2 with PD pathology are unknown. LRRK2 is a large (286 kDa), multidomain protein that has two enzymatic domains and several other moieties involved in protein–protein interactions. However, LRRK2 kinase inhibitors are the most frequently used, if not the only, pharmacological tools for the study of LRRK2 biology, leaving the GTPase domain and protein–protein interaction domains of LRRK2 underexamined. The LRRK2 degrader that we have developed and characterized in this study offers a new chemical tool for deciphering the biology of LRRK2.

Employing a target protein degradation strategy to treat neurodegenerative diseases can be revolutionary as protein aggregates are among the major pathologies and many attempts to modulate these diseases with conventional small-molecule drugs have not been successful. Significant effort has already been made to target neurodegenerative disease-related proteins with either peptide-based or small molecular PROTAC degraders.^77^ However, achieving favorable PK profiles with oral bioavailability and BBB penetration have been the major obstacles for the central nervous system (CNS)-targeted PROTACs. Among the only successes reported to date, Wang et al. developed a tau-targeting PROTAC (C004019) that can penetrate the BBB after subcutaneous injection and induce tau protein degradation in the brain.^78^ Herein, we disclose the identification of an LRRK2-targeting PROTAC that exhibits remarkable oral bioavailability and BBB penetration. Both CC004019 and XL01126 are VHL-based PROTACs with multiple violations of Ro5 and/or RoCNS. Their capability of penetrating the BBB challenges the Ro5- and RoCNS-based preconceptions and dogma and has expanded the chemical space of CNS targeting drugs. Although BBB permeable, XL01126 showed low concentrations in the brain and in CSF, with a low brain-to-plasma ratio (<0.035). Nevertheless, given the substoichiometric/catalytic mechanism of action, which is different from the occupancy-driven mechanism of inhibitors, PROTACs may achieve target protein degradation in the targeted tissue even with low exposure. Proper selection of administration dosage and routes and further lead optimization will also be important for maximizing compound exposure in the brain. Further *in vivo* LRRK2 degradation studies in various tissues and organs, including the brain, are ongoing, and the results will be reported in due course.

PROTAC is an emerging drug discovery modality, yet the development of an active and efficient degrader is still a laborious and unguided process. Structure-guided PROTAC design^64,68^ is an attractive strategy, but solving the crystal structure of a target protein:PROTAC:E3 ligase ternary complex is a challenging feat. The step-by-step PROTAC development strategy we used here provides an empirical and generalized roadmap for developing PROTACs against LRRK2 and other challenging targets. The ternary binding affinity assay and ternary complex formation assay we developed here successfully circumvented the use of recombinant full-length LRRK2 protein, which is challenging to express and purify. These two assays can potentially be applied to PROTAC or molecular glue development for other challenging targets as well.

Further optimization of XL01126 and related LRRK2 degraders may result in compounds that exhibit improved activity or druglike properties, improved selectivity for a particular LRRK2 mutant, decreased off-target degradation to PDE6D, and improved cooperativity, allowing further enhancement of the degradation *vs* inhibition window to achieve enhanced therapeutic performance.

## Experimental Section

### Chemistry

Chemicals that are commercially available were purchased from Apollo Scientific, Sigma-Aldrich, Fluorochem, and Enamine and were used without further purification. All solvents used for reactions are anhydrous. Liquid chromatography–mass spectrometry (LC-MS) was carried out on a Shimadzu HPLC/MS 2020 equipped with a Hypersil Gold column (1.9 μm, 50 × 2.1 mm^2^), a photodiode array detector, and an electrospray ionization (ESI) detector. The samples were eluted with a 3 min gradient of 5–95% acetonitrile (ACN) in water containing 0.1% formic acid at a flow rate of 0.8 mL/min. Flash column chromatography was performed on a Teledyne ISCO Combiflash Companion installed with disposable normal phase RediSep Rf columns (230–400 mesh, 40–63 mm; SiliCycle). Preparative HPLC purification was performed on a Gilson preparative HPLC system equipped with a Waters X-Bridge C18 column (100 mm × 19 mm and 5 μm particle size) using a gradient from 5 to 95% of acetonitrile in water containing 0.1% formic acid over 10 min at a flow rate of 25 mL/min. Compound characterization using NMR was performed either on a Bruker 500 Ultra shield or on a Bruker Ascend 400 spectrometer. The ^1^H NMR, ^13^C NMR, and ^19^F NMR reference solvents used are CDCl_3_-*d*_1_ (δH = 7.26 ppm/δC = 77.16 ppm), CD_3_OD-*d*_4_ (δH = 3.31 ppm/δC = 49.00 ppm), or DMSO-*d*_6_ (δH = 2.50 ppm/δC = 39.52 ppm). Signal patterns are described as singlet (s), doublet (d), triplet (t), quartet (q), quintet (quint.), multiplet (m), broad (br), or a combination of the listed splitting patterns. The coupling constants (*J*) are measured in hertz (Hz). High-resolution mass spectrometry (HRMS) was performed on a Bruker MicroTOF II focus ESI mass spectrometer connected in parallel to a Dionex Ultimate 3000 RSLC system with a diode array detector and a Waters X-Bridge C18 column (50 mm × 2.1 mm, 3.5 μm particle size). All final compounds are >95% pure by HPLC.

#### *tert*-Butyl (2*S*,4*S*)-4-Hydroxy-2-((4-(4-methylthiazol-5-yl)benzyl)carbamoyl)pyrrolidine-1-carboxylate (**2**)

To a solution of compound **1**(52) (1.2 g, 3.94 mmol) in DCM (7.9 mL) was added 4 N HCl in 1,4-dioxane (7.9 mL). After stirring at room temperature overnight, the mixture was concentrated under reduced pressure, washed with ethyl ether, and dried to give a light yellow solid (902 mg, 95% yield). To a suspension of the solid (500 mg, 2.08 mmol) in DCM (10 mL) were added TEA (0.962 mL), (2*S*,4*S*)-1-(*tert*-butoxycarbonyl)-4-hydroxypyrrolidine-2-carboxylic acid (480 mg, 2.08 mmol), and HATU (830 mg, 2.18 mmol). After stirring at room temperature overnight, the mixture was diluted with DCM, washed with water and brine, dried over sodium sulfate, filtered, and condensed to afford a residue, which was purified *via* flash column chromatography on silica gel (0–10% methanol in DCM) to give compound **2** as a solid (560 mg, 65% yield). ^1^H NMR (400 MHz, CDCl_3_) δ 8.67 (s, 1H), 7.52 (s, 1H), 7.36 (dd, *J* = 22.1, 7.8 Hz, 4H), 5.15 (d, *J* = 9.2 Hz, 1H), 4.58 (dd, *J* = 15.2, 6.3 Hz, 1H), 4.50–4.33 (m, 3H), 3.58–3.42 (m, 2H), 2.52 (s, 3H), 2.38 (d, *J* = 14.1 Hz, 1H), 2.22–2.12 (m, 1H), 1.45 (s, 9H). LC-MS, ESI^+^, *m*/*z* 418.0 [M + H]^+^.

#### (2*S*,4*S*)-1-((*R*)-2-(1-Fluorocyclopropane-1-carboxamido)-3-methyl-3-(tritylthio)butanoyl)-4-hydroxy-*N*-(4-(4-methylthiazol-5-yl)benzyl)pyrrolidine-2-carboxamide (**4**)

To a solution of compound **2** (568 mg, 1.36 mmol) in DCM (6.8 mL) was added 4 N HCl in 1,4-dioxane (6.8 mL). The resulting mixture was stirred at room temperature overnight and condensed to afford a solid (530 mg, 100% yield). To a solution of the obtained solid (200 mg, 0.57 mmol) and TEA (236 μL, 1.70 mmol) in DMF (5 mL) was added dropwise with a mixture of Fmoc-*S*-trityl-l-penicillamine (329 mg, 0.54 mmol), HATU (215 mg, 0.57 mmol), and TEA (79 μL, 0.57 mmol) in DMF (5 mL). After stirring at room temperature overnight, the mixture was diluted with DCM, washed with water and brine, dried over sodium sulfate, filtered, and condensed to afford a residue, which was purified with a flash column (0–10% 0.7 M ammonia-containing methanol in DCM) to afford a residue as an amine compound **3** (120 mg, 32% yield for two steps, LC-MS, ESI^–^, 689.4 [M – H]^−^), which was used in the next step. To a solution of the amine compound **3** (60 mg, 0.087 mmol) in DMF (1.5 mL) was added TEA (24 μL, 0.174 mmol), HATU (35 mg, 0.092 mmol), and 1-fluorocyclopropane-1-carboxylic acid (9 mg, 0.087 mmol) separately. After stirring at room temperature for 4 h, the resulting mixture was diluted with ethyl acetate and washed with water and brine, dried over sodium sulfate, filtered, and condensed to afford a crude product, which was purified *via* flash column chromatography (0–10% methanol in DCM) on silica gel to give **4** (57 mg, 85% yield) as a white solid. ^1^H NMR (400 MHz, CDCl_3_) δ 8.62 (s, 1H), 7.60 (t, *J* = 6.0 Hz, 1H), 7.52–7.45 (m, 6H), 7.25–7.21 (m, 2H), 7.19–7.07 (m, 12H), 5.26 (d, *J* = 9.6 Hz, 1H), 4.54 (d, *J* = 8.7 Hz, 1H), 4.35–4.23 (m, 2H), 4.16 (dd, *J* = 15.1, 5.4 Hz, 1H), 3.40 (d, *J* = 5.1 Hz, 1H), 3.34 (dd, *J* = 11.0, 4.0 Hz, 1H), 3.22 (d, *J* = 10.9 Hz, 1H), 2.43 (s, 3H), 2.12 (d, *J* = 14.0 Hz, 1H), 2.08–1.97 (m, 1H), 1.30–1.12 (m, 4H), 1.07 (s, 3H), 1.01 (s, 3H). ^13^C NMR (101 MHz, CDCl_3_) δ 172.74, 169.95, 169.73 (d, *J* = 20.35 Hz), 150.40, 148.65, 144.50, 137.65, 131.66, 131.14, 129.84, 129.62, 128.06, 127.92, 126.89, 78.30 (d, *J* = 226.86 Hz), 71.12, 68.37, 60.15, 58.94, 56.77, 53.73, 43.38, 35.48, 26.19, 25.71, 16.21, 13.66 (d, *J* = 9.46 Hz), 13.57 (d, *J* = 9.33 Hz). LC-MS, ESI^+^, *m*/*z*, 777.5 [M + H]^+^.

#### (2*S*,4*S*)-1-((*R*)-2-(1-Fluorocyclopropane-1-carboxamido)-3-mercapto-3-methylbutanoyl)-4-hydroxy-*N*-(4-(4-methylthiazol-5-yl)benzyl)pyrrolidine-2-carboxamide (**5**)

To a solution of compound **4** (57 mg, 0.073 mmol) in DCM (1.6 mL) was added triisopropylsilane (0.08 mL) and TFA (0.08 mL) at 0 °C. The resulting mixture was stirred at 0 °C for 30 min and condensed to afford a residue, which was purified through flash column chromatography (0–10% methanol in DCM) on silica gel to yield compound **5** (36 mg, 92% yield). ^1^H NMR (400 MHz, CDCl_3_) δ 8.82 (s, 1H), 7.52 (t, *J* = 5.9 Hz, 1H), 7.41–7.34 (m, 4H), 6.63 (br, s, 2H), 4.74–4.60 (m, 3H), 4.48 (t, *J* = 4.2 Hz, 1H), 4.31 (dd, *J* = 15.0, 5.0 Hz, 1H), 3.96 (dd, *J* = 11.0, 4.0 Hz, 1H), 3.91 (d, *J* = 11.0 Hz, 1H), 2.52 (s, 3H), 2.39–2.29 (m, 2H), 2.27–2.17 (m, 1H), 1.40–1.23 (m, 10H). ^13^C NMR (101 MHz, CDCl_3_) δ 172.53, 170.72, 170.14 (d, *J* = 20.51 Hz), 151.22, 147.71, 137.83, 132.26, 130.84, 129.84, 128.39, 78.21 (d, *J* = 231Hz), 71.15, 60.36, 58.70, 57.19, 46.43, 43.69, 35.65, 30.44, 28.93, 15.64, 13.96, 13.86. LC-MS, ESI^+^, *m*/*z* 535.4 [M + H]^+^.

#### 4-((5-Chloro-4-(methylamino)pyrimidin-2-yl)amino)-3-methoxybenzoic Acid (**7**)

To a solution of 2,5-dichloro-*N*-methylpyrimidin-4-amine^17^ (4.39 g, 24.65 mmol) in a mixture of dioxane and water (70 mL: 70 mL) was added 4-amino-3-methoxybenzoic acid (4.13 g, 24.70 mmol) followed by 4 N solution of HCl in dioxane (6.18 mL, 24.72 mmol) at room temperature. After refluxing the reaction mixture at 100 °C overnight, the mixture was cooled down to precipitate a white solid. The solids were filtered, washed with water, and dried under vacuum to afford compound **7** as a white solid (5.95 g, 19.32 mmol, 78% yield). ^1^H NMR (400 MHz, DMSO-*d*_6_) δ 12.64 (br s, 1H), 8.50 (d, *J* = 8.2 Hz, 1H), 8.02 (s, 1H), 7.93 (s, 1H), 7.59 (d, *J* = 8.2 Hz, 1H), 7.51 (s,1H), 7.47(m, 1H), 3.95 (s, 3H), 2.93 (d, *J* = 4.3 Hz, 3H). LC-MS, ESI^+^, *m*/*z* 309.08 [M + H]^+^.

#### *tert*-Butyl 4-(4-((5-Chloro-4-(methylamino)pyrimidin-2-yl)amino)-3-methoxybenzoyl)piperazine-1-carboxylate (**8**)

To a solution of **7** (2.1 g, 6.08 mmol) in DMF (25 mL) was added HOBt (0.98 g, 7.29 mmol), EDCI (1.39 g, 7.29 mmol), 1-Boc-piperazine (1.19, 6.38 mmol), and DIPEA (4.23 mL, 24.33 mmol) separately at room temperature. The mixture was stirred at room temperature for 16 h, then diluted with water (50 mL), and extracted with EtOAc (200 mL). The organic layer was washed with water and brine, dried over sodium sulfate, filtered, and concentrated to give a residue, which was purified by flash column chromatography on silica gel (0–100% of EtOAc in DCM) to give compound **8** as a white solid (2.52 g, 5.28 mmol, 87%). ^1^H NMR (500 MHz, CDCl_3_) δ 8.58 (d, *J* = 8.05 Hz, 1H), 7.94 (s, 1H), 7.65 (s, 1H), 7.02 (m, 2H), 5.34 (m, 1H), 3.95 (s, 3H), 3.64 (br, s, 4H), 3.48 (br, s, 4H), 3.13 (d, *J* = 4.8 Hz, 3H), 1.49 (s, 9H). ^13^C NMR (126 MHz, CDCl_3_) δ 170.93, 158.65, 157.84, 154.74, 152.73, 147.53, 131.69, 127.42, 120.28, 116.81, 109.61, 105.69, 80.42, 55.96, 44.06, 28.49, 28.21. LC-MS, ESI^+^, *m*/*z* 477.20 [M + H]^+^.

#### (4-(((1*R*,4*R*)-4-(Bromomethyl)cyclohexyl)methyl)piperazin-1-yl)(4-((5-chloro-4-(methylamino)pyrimidin-2-yl)amino)-3-methoxyphenyl)methanone (**9**)

To a solution of **8** (2.52 g, 5.28 mmol) in a mixture of DCM and MeOH 9:1 (30 mL) was added 4 N solution of HCl in dioxane (5.28 mL, 21.12 mmol) at room temperature. After stirring at room temperature overnight, the mixture was diluted with Et_2_O (200 mL) to precipitate a solid, which was filtered, washed with Et_2_O (100 mL), and dried overnight to give a Boc-deprotected product (2.13 g, 5.17 mmol, 98% yield) as a HCl salt. ^1^H NMR (500 MHz, DMSO-*d*_6_) δ 9.65 (s, 2H), 8.72 (s, 1H), 8.28 (s, 1H), 8.13 (d, *J* = 8.20 Hz, 1H), 7.20 (d, *J* = 1.70 Hz, 1H), 7.11 (dd, *J*_1_ = 1.70 Hz, *J*_2_ = 8.20 Hz,1H), 3.91 (s, 3H), 3.75 (br s, 4H), 3.15 (br s, 4H), 2.99 (d, *J* = 4.6 Hz, 3H). ^13^C NMR (126 MHz, DMSO-*d*_6_) δ 168.80, 158.62, 151.41, 149.78, 140.84, 131.47, 127.12, 121.40, 119.54, 110.60, 104.59, 56.20, 42.38, 34.05, 28.62. LC-MS, ESI^+^, *m*/*z* 377.15 [M + H]^+^. To a suspension of the salt (25 mg, 0.06 mmol) in acetone (3 mL) were added K_2_CO_3_ (42 mg, 0.30 mmol) and *trans*-1,4-bis(bromomethyl)cyclohexane (50 mg, 0.185 mmol) (see Scheme S2 for synthesis). After stirring at 50 °C for 2 days, the mixture was diluted with DCM, washed with water and brine, dried over sodium sulfate, filtered, and condensed to afford a residue, which was purified with flash column chromatography (0–10% methanol in DCM) on silica gel to give compound **9** (10 mg, 29% yield). ^1^H NMR (400 MHz, CDCl_3_) δ 8.54 (d, *J* = 8.8 Hz, 1H), 7.92 (s, 1H), 7.62 (s, 1H), 7.03–6.98 (m, 2H), 5.33–5.24 (m, 1H), 3.92 (s, 3H), 3.75–3.55 (s, 4H), 3.29 (d, *J* = 6.3 Hz, 2H), 3.11 (d, *J* = 4.9 Hz, 3H), 2.40 (s, 4H), 2.16 (d, *J* = 7.8 Hz, 2H), 1.95–1.81 (m, 4H), 1.71–1.58 (m, 1H), 1.50–1.38 (m, 1H), 1.07–0.85 (m, 4H). ^13^C NMR (101 MHz, CDCl_3_) δ 170.53, 158.72, 157.99, 152.83, 147.56, 131.43, 128.18, 120.31, 116.92, 109.74, 105.66, 65.33, 56.01, 54.03, 40.61, 40.50, 35.07, 31.49, 31.25, 29.45, 28.22. LC-MS, ESI^+^, *m*/*z* 567.00 [M + H]^+^.

(2*S*,4*S*)-1-((*R*)-3-((((1*R*,4*R*)-4-((4-(4-((5-Chloro-4-(methylamino)pyrimidin-2-yl)amino)-3-methoxybenzoyl)piperazin-1-yl)methyl)cyclohexyl)methyl)thio)-2-(1-fluorocyclopropane-1-carboxamido)-3-methylbutanoyl)-4-hydroxy-*N*-(4-(4-methylthiazol-5-yl)benzyl)pyrrolidine-2-carboxamide (*cis*-XL01126)

To a solution of compound **9** (13 mg, 0.023 mmol) in THF (1.5 mL) were added compound **5** (10 mg, 0.019 mmol) and DBU (0.016 mL, 0.11 mmol). After stirring at room temperature overnight, the mixture was condensed and purified with preparative HPLC under acidic conditions (5–95% CH_3_CN in 0.1% aq HCO_2_H) to give *cis*-XL01126 (11.9 mg, 62% yield) as a white solid. ^1^H NMR (400 MHz, CDCl_3_) δ 8.67 (s, 1H), 8.53 (d, *J* = 8.8 Hz, 1H), 7.92 (s, 1H), 7.62 (s, 1H), 7.45 (t, *J* = 5.9 Hz, 1H), 7.41–7.33 (m, 4H), 7.17 (dd, *J* = 8.1, 3.3 Hz, 1H), 7.03–6.98 (m, 2H), 5.31–5.28 (m, 1H), 4.73 (dd, *J* = 18.0, 8.3 Hz, 2H), 4.58 (dd, *J* = 15.0, 6.6 Hz, 1H), 4.53–4.46 (m, 1H), 4.37 (dd, *J* = 15.0, 5.3 Hz, 1H), 3.97–3.84 (m, 5H), 3.63 (s, 4H), 3.10 (d, *J* = 4.9 Hz, 3H), 2.52 (s, 3H), 2.43–2.32 (m, 7H), 2.21 (ddd, *J* = 14.0, 9.2, 4.8 Hz, 1H), 2.12 (d, *J* = 7.0 Hz, 2H), 1.82 (t, *J* = 13.3 Hz, 4H), 1.44–1.28 (m, 12H), 1.03–0.76 (m, 4H); ^13^C NMR (126 MHz, CDCl_3_) δ 172.39, 170.59, 170.51, 170.02 (d, *J* = 20.6 Hz), 158.67, 157.92, 152.78, 150.49, 148.71, 147.50, 137.38, 131.58, 131.42, 131.36, 129.77, 128.24, 120.26, 116.82, 109.65, 105.60, 79.21, 71.22, 65.42, 60.27, 58.63, 55.98, 55.89, 53.90, 47.56, 43.66, 38.44, 35.48, 34.98, 32.77, 32.66, 31.46, 28.23, 25.74, 25.34, 16.26, 13.92, 13.85; ^19^F NMR (471 MHz, CDCl_3_) δ −197.78. HRMS (ESI^+^) *m*/*z*, calcd for C_50_H_64_ClFN_10_O_6_S_2_: 1019.4197 [M + H]^+^, found 1019.4206.

(2*S*,4*R*)-1-((*R*)-3-((((1*R*,4*R*)-4-((4-(4-((5-Chloro-4-(methylamino)pyrimidin-2-yl)amino)-3-methoxybenzoyl)piperazin-1-yl)methyl)cyclohexyl)methyl)thio)-2-(1-fluorocyclopropane-1-carboxamido)-3-methylbutanoyl)-4-hydroxy-*N*-(4-(4-methylthiazol-5-yl)benzyl)pyrrolidine-2-carboxamide (XL01126)

To a solution of compound **9** (8 mg, 0.014 mmol) in THF (1.5 mL) were added compound **10**(79) (7.6 mg, 0.019 mmol) and DBU (0.012 mL, 0.085 mmol). After stirring at room temperature overnight, the mixture was condensed and purified with preparative HPLC under acidic conditions (5–95% CH_3_CN in 0.1% aq HCO_2_H) to give XL01126 (7.7 mg, 53% yield) as a white solid. ^1^H NMR (400 MHz, CDCl_3_) δ 8.67 (s, 1H), 8.54 (d, *J* = 8.8 Hz, 1H), 7.92 (s, 1H), 7.62 (s, 1H), 7.41–7.30 (m, 5H), 7.22 (dd, *J* = 7.8, 3.3 Hz, 1H), 7.02–6.98 (m, 2H), 5.35–5.20 (m, 1H), 4.79 (t, *J* = 7.9 Hz, 1H), 4.72 (d, *J* = 7.7 Hz, 1H), 4.53 (s, 1H), 4.46 (d, *J* = 5.9 Hz, 2H), 4.06 (d, *J* = 11.3 Hz, 1H), 3.92 (s, 3H), 3.76–3.50 (m, 5H), 3.11 (d, *J* = 4.9 Hz, 3H), 2.72 (s, 1H), 2.52 (s, 3H), 2.52–2.45 (m, 1H), 2.44–2.31 (m, 6H), 2.28–2.19 (m, 1H), 2.11 (d, *J* = 7.1 Hz, 2H), 1.85–1.75 (m, 4H), 1.48–1.21 (m, 12H), 0.99–0.75 (m, 4H). ^13^C NMR (101 MHz, CDCl_3_) δ 170.73, 170.64 (d, *J* = 24.8 Hz), 170.20, 158.73, 157.98, 152.82, 150.36, 148.69, 147.56, 138.18, 131.72, 131.45, 131.18, 129.66, 128.18, 120.30, 116.92, 109.74, 105.67, 78.4 (d, *J* = 261.9 Hz), 70.29, 65.41, 58.99, 56.67, 56.38, 56.02, 53.94, 47.66, 43.26, 38.54, 36.76, 35.41, 35.10, 32.83, 32.76, 31.48, 28.22, 25.79, 25.43, 16.27, 14.07 (d, *J* = 17.5 Hz), 14.0 (d, *J* = 17.4 Hz). ^19^F NMR (471 MHz, CDCl_3_) δ −197.75. HRMS (ESI^+^) *m*/*z*, calcd for C_50_H_64_ClFN_10_O_6_S_2_: 1019.4197 [M + H]^+^, found 1019.4173.

### Generation of Mouse Embryonic Fibroblasts (MEFs)

Primary MEFs were generated as described in a previous study.^80^ Briefly, the uterine horn was collected from adult female mice at day E12.5 and transferred to a 10 cm tissue culture dish containing cold PBS. Two forceps were used to tear the yolk sacs to isolate each embryo. Forceps were cleaned thoroughly with 70% ethanol between each embryo isolation. The embryos were culled, and a tissue piece was collected in a polymerase chain reaction (PCR) tube for genotyping. The red tissue of the embryo was removed, and the remainder was minced with a scalpel blade and incubated with a 7.5 mL trypsin–ethylenediaminetetraacetic acid (EDTA) solution for 10 min in a 37 °C, 5% CO_2_ tissue culture incubator. The dish was removed from the incubator and checked under a light microscope for single cells. Complete media (7.5 mL) was added to the trypsinized cells, and the cell suspension was transferred to a 15 mL Falcon tube and centrifuged at 1200 rpm for 5 min at room temperature. The trypsin was aspirated, the cell pellet was resuspended in a 5 mL fresh complete media, and the cell suspension was plated in a 60 mm tissue culture dish and incubated in a 37 °C, 5% CO_2_ tissue culture incubator. The MEFs at this stage were considered as passage 0 and were passaged and expanded for experimental use once the genotype was confirmed by allelic sequencing and immunoblotting. MEFs were cultured in Dulbecco’s modified Eagle’s medium (DMEM) containing 10% (v/v) fetal bovine serum (FBS), 2 mM l-glutamine, 100 U/mL penicillin, and 100 μg/mL streptomycin supplemented with 1× nonessential amino acids and 1 mM sodium pyruvate.

### Generation of Bone Marrow-Derived Macrophages (BMDMs)

Macrophages were cultured in complete media containing DMEM, 10% (v/v) heat-inactive FBS, 20% (v/v) L929 preconditioned medium, 2.5% (v/v) *N*-(2-hydroxyethyl)piperazine-*N*′-ethanesulfonic acid (HEPES), 2 mM l-glutamine, 100 U/mL penicillin, 100 μg/mL streptomycin, 2% sterile-filtered β-mercaptoethanol, 1× nonessential amino acids, and 1 mM sodium pyruvate. Bone marrow isolation and macrophage differentiation were modified from ref (81), employing an L929 preconditioned medium as the source of M-CSF for differentiation. Briefly, scissors and forceps were used to dissect femurs and tibiae from adult mice, and muscle tissues were carefully removed from bones. Clean femurs and tibiae were placed in a tissue culture dish containing complete media. The ends of each bone were cut with scissors to expose the bone marrow. The bone marrow was flushed with a 25-gauge needle attached to a 10 mL syringe containing complete media. The media containing bone marrow was passed through a 70 μm cell strainer, and precursor cells were plated on nontissue culture-treated 10 cm bacteriological plates containing 10 mL complete media. This was marked as day 0 of isolation. On day 3 post isolation, macrophages were topped up with 5 mL of fresh complete media. On day 7 post isolation, macrophages were rinsed once with PBS and incubated with versene for 5 min in a 37 °C 5% CO_2_ tissue culture incubator. Macrophages were detached with cell scrapers and centrifuged at 1200 rpm for 5 min at room temperature. The versene was aspirated, and the remaining cell pellet was resuspended in complete media. The cell suspension was counted, and cells were seeded for experimental analysis in a six-well format in tissue culture-treated dishes at a final cell density of one million cells per well of a six-well plate.

### PBMC Cell Separation and Treatment

PBMC cells were separated from human blood from healthy volunteer donors following the existing protocol^82^ and pelleted by centrifugation at 1000*g* for 2 min. The supernatant was discarded, and the PBMC pellet was resuspended in PBS containing 2% FBS for washing. The suspension was centrifuged at 1000*g* for 2 min again, and the PBMC pellet was resuspended in Roswell Park Memorial Institute (RPMI)-1640 (Gibco) media supplemented with 10% FBS. The cells were then seeded into six-well plates and treated with testing compounds at indicated concentrations and time periods. After treatment, the cells were collected into a 2 mL Eppendorf tube and centrifuged at 500*g* for 2 min to pellet the cells, the supernatant was discarded, and the pellet was resuspended in 1 mL PBS and centrifuged at 500*g* for 2 min again. The PBMC pellet was lysed with 60 μL of lysis buffer containing 50 mM tris–HCl, pH 7.5, 1% (v/v) Triton X-100, 1 mM ethylene glycol-bis(β-aminoethyl ether)-*N*,*N*,*N*′,*N*′-tetraacetic acid (EGTA), 1 mM sodium orthovanadate, 50 mM NaF, 0.1% (v/v) 2-mercaptoethanol, 10 mM 2-glycerophosphate, 5 mM sodium pyrophosphate, 0.1 μg/mL microcystin-LR (Enzo Life Sciences), 270 mM sucrose, and 0.5 mM diisopropyl fluorophosphate (DIFP) (Sigma-Aldrich, Cat# D0879) in addition to a complete EDTA-free protease inhibitor cocktail (Sigma-Aldrich Cat # 11836170001). DIFP is highly toxic and must be prepared in a fume hood to a stock solution of 0.5 M in isopropanol. The lysed cells were then centrifuged at 1500*g* for 15 min at 0 °C. The supernatants were collected for analysis by quantitative immunoblotting. For long-term storage, the supernatant was flash-frozen and stored at −80 °C. Protein concentrations of cell lysates were determined using the Pierce BCA Protein Assay Kit (Thermo Fisher).

### Cell Culture, Treatment, and Lysis

Culturing and passaging of adherent cell lines were carried out using an aseptic technique in CL1 or CL2 (for PBMC isolation) biological safety cabinets. All cells were incubated in a 37 °C incubator with 5% CO_2_. Cell lines were regularly tested for mycoplasma contamination. For Western blot assay, the cells were seeded in six-well plates. For immunoprecipitation of LRRK2, SH-SY5Y cells (cultured in DMEM-F12, supplemented with 15% (v/v) FBS, 100 U/mL penicillin and 100 μg/mL streptomycin, 1× nonessential amino acids, and 1 mM sodium pyruvate) were seeded in a 10 cm dish. All cells were treated with the indicated compounds such that the final concentration of DMSO was 0.1%. Following the treatment of cells with compounds at indicated concentrations and time periods, the media was removed and the cells were washed with PBS and lysed in a 100 μL ice-cold complete lysis buffer containing 50 mM tris–HCl pH 7.4, 1 mM EGTA, 10 mM 2-glycerophosphate, 50 mM sodium fluoride, 5 mM sodium pyrophosphate, 270 mM sucrose, supplemented with 1 μg/mL microcystin-LR, 1 mM sodium orthovanadate, complete EDTA-free protease inhibitor cocktail (Roche), and 1% (v/v) Triton X-100. The cells were immediately placed on ice and were scraped and collected into 1.5 mL Eppendorf tubes. Cell lysates were incubated on ice for 10 min prior to centrifugation at 15 000*g* at 4 °C for 15 min. The cell pellet was discarded, and the supernatant was collected for analysis by quantitative immunoblotting. For long-term storage, the supernatant was flash-frozen and stored at −80 °C. Protein concentrations of cell lysates were determined using the Bradford assay.

All experiments with human peripheral blood were performed in guidance with local standard operating procedures, in line with the Human Tissue Act^83^ and good clinical practice^84^ for research. Nonclinical local ethical approval was in place, and donors gave written informed consent.

### Quantitative Immunoblotting

Cell lysates containing a quarter of a volume of 4× NuPAGE LDS sample buffer (NP0007) supplemented with 5% β-mercaptoethanol were heated at 95 °C for 5 min. Samples (15–20 μg) were loaded onto precast 4–12% bis–tris midi 20W or 26W gels (Thermo Fisher Scientific, Cat# WG1402BOX or WG1403BOX) and resolved at 130 V for 2 h with a NuPAGE MOPS SDS running buffer (Thermo Fisher Scientific Cat# NP0001-02). Proteins were electrophoretically transferred onto a 0.45 μm nitrocellulose membrane (GE Healthcare, Amersham Protran Supported 0.45 mm NC) at 90 V for 90 min on ice in a transfer buffer (48 mM tris base and 39 mM glycine supplemented with 20% methanol). The transferred membrane was blocked with 5% (w/v) skim milk powder dissolved in tris-buffered saline with Tween (TBS-T) (50 mM tris base, 150 mM sodium chloride (NaCl), 0.1% (v/v) Tween-20) at room temperature for 1 h. Membranes were washed three times with TBS-T and were incubated in primary antibody overnight at 4 °C. Prior to secondary antibody incubation, membranes were washed three times for 15 min with TBS-T. The membranes were incubated with a secondary antibody for 1 h at room temperature and protected from light. Thereafter, the membranes were washed with TBS-T three times with a 15 min incubation for each wash, and protein bands were acquired *via* a near-infrared fluorescent detection using the Odyssey CLx imaging system and quantified using Image Studio software. Graphs were generated using Graphpad Prism version 8 software.

### Antibodies

Monoclonal rabbit LRRK2 Ser935 (Cat# UDD2) was purified by MRC-PPU Reagents and Services at the University of Dundee and was used at a final concentration of 1 μg/mL. Total LRRK2 (C-terminus) was from Antibodies Inc./Neuromab (Cat# 75-253) and was diluted to 1:1000. The MJFF monoclonal rabbit Rab10-pThr73, which was characterized previously,^85^ was purchased from Abcam Inc. (ab230261) and diluted to 1:1000. Mouse monoclonal α-tubulin (#3873) was purchased from Cell Signaling Technology and used at 1:1000. The mouse monoclonal anti-Rab10 total antibody was purchased from Nanotools (#0680-100/Rab10-605B11) and was used at a final concentration of 1 μg/mL. Mouse monoclonal Hif-1α was purchased from R&D Systems (Cat# MAB1536) and was diluted to 1:1000. Mouse monoclonal Ubiquitin was purchased from Biolegend (Cat# 646302) and was diluted to 1:1000. Rabbit polyclonal PDE6D antibody was purchased from Novus Biologicals and was used at a final concentration of 1:500. The mouse GAPDH antibody (6C5) used on detecting PBMC cell protein was purchased from Santa Cruz Biotechnology (SCBT) (Cat. # sc-32233) and used with 1:2000 dilution. The rabbit GAPDH antibody used on detecting LRRK2 KO MEF protein was purchased from Cell Signaling Technology (CST) (Cat. # 2118S) and used with 1:10 000 dilution. All rabbit and mouse primary antibodies were diluted in 5% (w/v) bovine serum albumin (BSA) dissolved in TBS-T (50 mM tris base, 150 mM sodium chloride (NaCl), 0.1% (v/v) Tween-20). Goat antimouse IRDye 800CW (#926-32210), goat antimouse IRDye 680LT (#926-68020), goat antirabbit IRDye 800CW (#926-32211), and goat antirabbit IRDye 680LT (#926-68021) IgG (H + L) secondary antibodies were from LI-COR and were diluted 1:10 000 in 5% (w/v) milk in TBS-T.

### Total Proteome Sample Preparation and MS Analysis

Wild-type MEFs were seeded in 10 cm tissue culture dishes at a density of two million cells per dish. Cells were treated with 0.1% DMSO, 300 nM XL01126, or 300 nM *cis*-XL01126 for 4 h prior to harvest in a 400 μL complete lysis buffer, supplemented with 1 μg/mL microcystin-LR, 1 mM sodium orthovanadate, complete EDTA-free protease inhibitor cocktail (Roche), and 1% (v/v) Triton X-100. Cell lysates were incubated on ice for 10 min and then underwent three rounds of high-energy sonication for 15 cycles (30 s on, 30 s off) using the Diagenode Bioruptor. Cell lysates were centrifuged at 15 000*g* at 4 °C for 15 min. The cell pellet was discarded, and the supernatant was collected for protein quantification using a BCA protein assay kit (Pierce #23225). One hundred micrograms of cell lysate was employed for total proteomic analysis. Proteins in cell lysate were reduced with 0.1 M tris(2-carboxyethyl)phosphine (TCEP) diluted in 300 mM triethylammonium bicarbonate (TEABC) to a final concentration of 10 mM. Samples were incubated on a Thermomixer for 30 min at 60 °C at 800 rpm and then cooled down to room temperature and underwent alkylation with 0.04 M iodoacetamide (IAA) freshly dissolved in water. Samples were then incubated in the dark on a Thermomixer at room temperature for 30 min at 800 rpm. Alkylation was quenched with the addition of 0.1 M TCEP dissolved in 300 mM TEABC at a final concentration of 5 mM. Samples were incubated on a Thermomixer at room temperature for 20 min at 800 rpm. Sodium dodecyl sulfate (SDS) was added to a final concentration of 5% (w/v) from a 20% (w/v) stock. Phosphoric acid (12% (v/v)) was then added to a final concentration of 1.2% (v/v). Samples were diluted six times the sample volume of S-trap wash buffer containing 90% (v/v) methanol diluted in 100 mM (v/v) TEAB pH 7.1.

### S-Trap Cleanup and Digestion

Samples underwent S-trap cleanup to remove detergents and other impurities with S-trap mini columns (PROTIFI Cat# MSPPC02-MINI-80) placed in 2 mL Eppendorfs. The protein mixtures were added to columns and centrifuged briefly (1000*g*/1 min/RT). Columns were washed with 400 μL of S-trap buffer four times, centrifuging after each wash at 1000*g*/1 min/RT. Columns were placed in fresh 2 mL Eppendorfs, and 100 μL of 5 μg trypsin/Lys-C freshly dissolved in 50 mM TEAB, pH 8.5 was added. Columns were centrifuged briefly (200*g*/1 min/RT), and trypsin/Lys-C mixture was pipetted back onto the column. TEAB (100 μL of 50 mM), pH 8.5 was added directly to the 2 mL Eppendorfs to cover any digested peptides remaining in the tube. The S-trap columns in 2 mL Eppendorfs were incubated at 47 °C without shaking for 1.5 h and then at RT overnight. TEAB (80 μL of 50 mM) was added to S-trap columns, which were centrifuged, and eluates were collected in new 1.5 mL Eppendorf tubes. Formic acid (80 μL of 0.2% (v/v)) was added to columns, which were centrifuged, and second eluates were pooled with the first eluates. Acetonitrile (80 μL of 50% (v/v)) diluted in 0.2% (v/v) formic acid was added to columns, which were centrifuged, and third eluates were pooled with previous eluates. Digested peptides (500 ng) were set aside to vacuum dry separately to verify that the digestion efficiency by calculating the zero and single missed cleavages was >98%. The remaining peptides were divided in half (50 μg peptides each tube) and vacuum-dried and stored in −80 °C prior to continuation with the tandem mass tag (TMT) labeling.

### TMT Labeling

Eight hundred micrograms of TMT mass tag reagents were dissolved in 80 μL of 100% (v/v) anhydrous acetonitrile to obtain the final concentrations of 10 μg/μL. Resuspended TMT reagents were incubated at RT for 10 min and then vortexed and centrifuged briefly (2000*g*/2 min/RT). Fifty micrograms of lyophilized peptides were resuspended in 50 μL of a mixture containing 42 μL of 50 mM TEAB and 8 μL of 100% (v/v) anhydrous acetonitrile. Resuspended peptides were sonicated for 10 min and then centrifuged at 17 000*g* for 10 min at RT. Peptides were transferred to fresh protein low-bind 1.5 mL Eppendorf tubes. Twenty microliters of 10 μg/μL TMT reagent was added to solubilized peptides, vortexed, centrifuged briefly (2000*g*/1 min/RT), and incubated on a Thermomixer for 2 h at 800 rpm at RT. Fifty microliters of 50 mM TEAB was added to each reaction, followed by vortex, brief centrifugation (2000*g*/1 min/RT), and incubation on a Thermomixer at 800 rpm at RT for an additional 10 min. Five microliters of each TMT-labeled sample was set aside, vacuum-dried, and injected on MS to confirm that the labeling efficiency was >98%. The remaining reactions were stored at −80 °C until the labeling efficiency was verified. TMT samples were thawed to RT, and labeling reactions were quenched with the addition of 5 μL of 5% (v/v) hydroxylamine (dissolved in water from a 50% (v/v) stock solution). Samples were incubated on a Thermomixer for 20 min at 800 rpm at RT. The quenched TMT-labeled samples were pooled, vacuum-dried, and subjected to high-pH fractionation, as described previously.^86^ Ninety-six fractions were collected and concatenated into 48 fractions. The pooled fractions were vacuum-dried and stored in a −20 freezer until the liquid chromatography with tandem mass spectrometry (LC-MS/MS) analysis.

### LC-MS/MS Analysis

High-pH fractions were solubilized in 60 μL of LC-solution (3% ACN (v/v) and 0.2% formic acid (v/v) in water) by placing them on a Thermomixer at room temperature for 30 min with an agitation at 1800 rpm. Seven microliters of each fraction was transferred into LC-vail inserts for mass spectrometry analysis. LC-MS/MS analysis was carried out on a Thermo Lumos ETD Tribrid mass spectrometer inline with a 3000 ultimate RSLC nano-liquid chromatography system. The sample was injected into precolumn (C18, 5 μm, 100Ao, 100μ, 2 cm Nano-viper column # 164564, Thermo Scientific) at 5 μL/min flow rate and subsequently loaded onto the analytical column (C18, 5 μm, 50 cm, 100Ao Easy nano spray column # ES903, Thermo Scientific) for the separation of peptides using nano pump operated at a 300 nL/min flow rate. An 85 min nonlinear gradient was applied (5% solvent B (80% ACN v/v in 0.1% formic acid v/v) to 22% B for 70 min and increased to 35% B for another 10 min for a total of 100 min run time). The eluted peptides were electrosprayed into the mass spectrometer using the easy nanosource. The data were acquired in a data-dependent acquisition (DDA) mode in SPS MS3 (FT-IT-HCD-FT-HCD) method and was acquired using a top speed for 2 s for each duty cycle. The full MS1 scan was acquired at a 120 000 resolution at *m*/*z* 200 and analyzed using an ultrahigh field Orbitrap mass analyzer in the scan range of 375–1500 *m*/*z*. The precursor ions for MS2 were isolated using a Quadrupole mass filter at a 0.7 Da isolation width and fragmented using a normalized 35% higher-energy collisional dissociation (HCD) of ion routing multipole analyzed using ion trap. The top 10 MS2 fragment ions in a subsequent scan were isolated and fragmented using HCD at a 65% normalized collision energy and analyzed using an Orbitrap mass analyzer at a 50 000 resolution in the scan range of 100–500 *m*/*z*.

### Database Search and Data Analysis

Raw MS data of 48 high-pH fractions were searched using the MaxQuant search algorithm (version 2.0.3.0)^87^ against the Uniprot Mouse database (release version May 20021 containing 25,375 sequences). A 10 plex TMT reporter ion MS3 workflow was loaded and used following the search parameters. Trypsin as a protease was selected by allowing two missed cleavages, deamidation of Asn and Gln; oxidation of Met was used as a variable modification, and carbamidomethylation of Cys was used as a fixed modification. The default mass error tolerance for MS1 and MS2 (4 ppm and 20 ppm) was used. A minimum of two unique + razor peptides were selected for the quantification. The data were filtered for 1% PSM, peptide, and protein level FDR. The output protein group .txt files were further processed using the companion Perseus software suite (version 1.6.15.0).^88^ Decoy hits, contaminants, proteins identified by sites, and single peptide hits were filtered out. The data were then log 2-transformed, and *T*-test was performed between the sample groups and the *p*-values were corrected using a 5% permutation-based FDR to identify the differentially regulated protein groups. The mass spectrometry proteomics data have been deposited to the ProteomeXchange Consortium *via* the PRIDE^89^ partner repository with the data set identifier PXD034055.

### Fluorescence Polarization Assay

FP competitive binding assays were performed following the method described previously.^50,59^ All of the measurements were taken on a PHERAstar (BMG LABTECH) plate reader installed with an FP filter that sets excitation and emission wavelengths at 485 nm and 520 nM separately. Each well of a 384-well plate (Coring 3575) contains 10 nM VCB protein, 5 nM FAM-labeled HIF-1α peptide (FAM-DEALAHypYIPMDDDFQLRSF, “JC9”), and decreasing concentrations of testing compounds (14 concentrations with a 2-fold serial dilution starting from 250 μM) in FP assay buffer (100 mM bis–tris propane, 100 mM NaCl, 1 mM TCEP, pH 7) with a final DMSO concentration of 5%. The control wells containing the VCB and JC9 with no compound are set as the maximum signals (zero displacement). And the control wells containing JC9 in the absence of protein are set as the minimum signals. Control values were used to obtain the percentage of displacement, which was plotted against Log[Compound]. The average IC_50_ values were determined for each titration using nonlinear regression analysis with GraphPad Prism (v.9.3.1). The *K*_i_ values were back-calculated from a *K*_d_ of JC9 (1.5–3.4 nM) and the fitted IC_50_ values, as described previously.^90,91^

### NanoBRET Target Engagement Assay

For VHL and LRRK2 target engagement experiments in live and permeabilized cells, the HEK293 cells were transfected with the VHL-NanoLuc fusion vector (Promega, N275A) or LRRK2-NanoLuc fusion vector (Promega, NV3401) following Promega’s protocol and seeded into a white 384-well plate (Corning3570) at a density of 6000 cells/well. To measure NanoBRET in permeabilized cells, the cells were treated with 50 μg/mL of digitonin (Sigma-Aldrich, D141), 125 nM of VHL tracer/125 nM of LRRK2 tracer, testing compounds at decreasing concentrations (12 concentrations with a 2-fold serial dilution starting from 33 μM), and NanoBRET NanoGlo Substrate (Promega, N157C) at concentrations recommended by the manufacturer’s protocol. In the maximum signal control samples (DMSO control), DMSO was added instead of testing compounds. In the minimum signal control samples (no tracer control), DMSO and tracer dilution buffer were used to replace testing compounds and tracer separately. The filtered luminescence was measured within 10 min following the addition of the substrate on a GloMax Discover microplate reader (Promega) or a PHERAstar (BMG LABTECH) plate reader equipped with a 450 nm bandpass filter (donor) and a 600 nm long-pass filter (acceptor). To measure NanoBRET in live cells, the cells were treated with 250 nM VHL tracer/500 nM LRRK2 tracer, testing compounds at decreasing concentrations (12 concentrations with a 2-fold serial dilution starting from 33 μM) and incubated at 37 °C in an incubator for 2 h. The plates were then cooled down, and a NanoBRET NanoGlo Substrate and an Extracellular NanoLuc Inhibitor (Promega, N2160) were added before performing the same NanoBRET reading as the permeabilized mode on plate readers. The NanoBRET ratio of each well was expressed in milliBRET according to the equation: mBRET = [(signal at 610 nM/signal at 450 nM) – (signal at 610 nM_no tracer control_/signal at 450 nM_no tracer control_)] × 1000. The fractional occupancy was calculated according to the equation: fractional occupancy = (mBRET_testing compound_ – mBRET_no tracer control_)/(mBRET_DMSO control_ – mBRET_no tracer control_).

### NanoBRET-Based Ternary Binding and Cooperativity Assay

The HEK293 cells were transfected with the LRRK2-NanoLuc fusion vector (Promega, NV3401) following Promega’s protocol and seeded into a white 384-well plate (Corning3570) at a density of 6000 cells/well. The cells were then treated with 50 μg/mL of digitonin, 125 nM of LRRK2 tracer, testing compounds at decreasing concentrations (11 concentrations with a 2-fold serial dilution starting from 10 μM) or testing compounds and VCB mix (11 concentrations with a 2-fold serial dilution starting from 10 μM compound). The first six concentrations of VCB start from 32 μM with a 2-fold dilution, while the last five concentrations of VCB were kept fixed at 1 μM. The NanoBRET NanoGlo Substrate (Promega, N157C) was added at concentrations recommended by the manufacturer’s protocol. In the maximum signal control samples (DMSO control), DMSO was added instead of testing compounds. In the minimum signal control samples (no tracer control), DMSO and LRRK2 tracer dilution buffer were used to replace testing compounds and LRRK2 tracer separately. The filtered luminescence was measured within 10 min following the addition of the substrate on a PHERAstar (BMG LABTECH) plate reader equipped with a 450 nm bandpass filter (donor) and a 600 nm long-pass filter (acceptor). The fractional occupancy was calculated according to the equation: fractional occupancy = (mBRET_testing compound_ – mBRET_no tracer control_)/(mBRET_DMSO control_ – mBRET_no tracer control_).

### Bodipy^576/589^ Labeling of VCB

VCB was labeled with Bodipy^576/589^ following a protocol reported previously.^92^ Briefly, the VCB complex was mixed with a Bodipy^576/589^ NHS ester in a 20:1 molar ration and incubated at room temperature (protected from light) for 2 h in reaction buffer (0.1 M sodium phosphate, 75 mM KOAc, 2 mM dithiothreitol (DTT), pH 7.4). The reaction was quenched by diluting 10 times with the reaction buffer, and the unreacted dye was removed with a PD-10 MiniTrap desalting column (GE Healthcare) equilibrated with 100 mM bis–tris pH 7.0, 100 mM NaCl, 1 mM DTT, pH 7. The eluted labeled protein solution was collected and concentrated with a Pierce Concentrator, 3k molecular weight cut-off (MWCO) (Thermo Scientific).

### NanoBRET Ternary Complex Formation Assay

HEK293 cells were transfected with LRRK2-NanoLuc vector (Promega, NV3401) for 24 h, harvested, and resuspended into OptiMEM media without phenol red (Life Technologies). The cells were then seeded into a white 384-well plate (Corning3570) at a density of 6000 cells/well. Digitonin solution (final concentration 50 μg/mL), testing PROTACs at decreasing concentrations (11 concentrations with a 2-fold serial dilution starting from 33 μM) or DMSO, and VCB protein labeled with Bodipy^576/589^ (final concentration 0.5 μM) were added separately. Each well was added with a NanoBRET NanoGlo Substrate (Promega, N157C) before performing the NanoBRET reading on a PHERAstar (BMG LABTECH) plate reader equipped with a 450 nm bandpass filter (donor) and a 600 nm long-pass filter (acceptor). The NanoBRET ratio of each well was expressed in milliBRET according to the equation: mBRET = [(signal at 610 nM/signal at 450 nM) – (signal at 610 nM_no tracer control_/signal at 450 nM_no tracer control_)] × 1000. The background signal as shown in the DMSO control samples was subtracted from each sample.

### Evaluation of Mitophagy in Immortalized Mito-QC MEFs

Immortalized *mito*-QC MEFs^8,93^ were maintained in DMEM (Gibco, 11960–044) supplemented with 10% FBS, 2 mM l-glutamine (Gibco, 2503-081), 1% Na-pyruvate (Gibco, 11360-070), 1% nonessential amino acids (Gibco, 11140–035), and 1% antibiotics (penicillin/streptomycin, 100 U/mL penicillin and 100 μg/mL streptomycin; Gibco) at 37 °C under a humidified 5% CO_2_ atmosphere. To assess mitophagy, MEFs were plated on #1.5 glass coverslips (Epredia, CB00130RAC20MNZ0) and treated for 24 h with XL01126, *cis*-XL01126, MLi-2 (positive control), or DMSO (vehicle).^8,18^ All treatments were in DMEM (Gibco, 11960-044) supplemented with 10% FBS, 2 mM l-glutamine (Gibco, 2503-081), 1% nonessential amino acids (Gibco, 11140-035), and 1% antibiotics (penicillin/streptomycin, 100 U/mL penicillin and 100 μg/mL streptomycin; Gibco) at 37 °C under a humidified 5% CO_2_ atmosphere. MLi-2 was synthesized as previously described.^18^ At the end of the treatment, cells were washed twice with Dulbecco’s phosphate-buffered saline (DPBS) (Gibco, 14190-094) and fixed with 3.7% paraformaldehyde (Sigma-Aldrich, P6148), 200 mM HEPES, pH = 7.00 for 20 min. Cells were washed twice and then incubated for 10 min with DMEM, 10 mM HEPES. After a wash with DPBS, coverslips were mounted on a slide (VWR, Superfrost, 631-0909) with Prolong Glass (Thermo Fisher Scientific, P36984). Images were acquired using a Zeiss LSM880 with an Airyscan laser scanning confocal microscope (Plan-Apochromat 63x/1.4 Oil DIC M27) using the optimal parameters for acquisition (Nyquist). Three to five biological replicates were performed for each experiment with 10 images acquired per condition (124–260 cells per condition). Quantification of red-only dots was semiautomatized using the *mito*-QC counter plugin on FIJI, as previously described,^8,94^ using the following parameters: radius for smoothing images = 2, ratio threshold = 1.5, and red channel threshold = mean + 1 SD. One-way analysis of variance (ANOVA) with Dunnett’s multiple comparisons was performed using GraphPad Prism version 9.3.1. *p*-Values are represented as *p < 0.05. Error bars denote SEM.

### Caco-2 Cell Permeability

Caco-2 cells with transepithelial electrical resistance (TEER) (TEER = (resistance sample – resistance blank) × effective membrane area) = 450 ± 19 Ω cm^2^were used for the experiment. Compounds were dissolved in the appropriate buffer (10 mM DMSO stock solutions were diluted with HBSS buffer to a final concentration of 10 μM testing compound and 0.4% DMSO, Lucifer Yellow was introduced in the apical side buffer to test the intactness of the monocell layer) and was applied to the apical or basolateral donor side for measuring A–B or B–A permeability (two replicates), respectively. The apical and basolateral plates were prewarmed to 37 °C before placing the apical plate onto the basolateral plate. After incubating at 37 °C for 90 min, the apical plate and basolateral plate were separated, and the donor or receiver samples were analyzed with UPLC-MS/MS.

### Plasma Stability Assay

Frozen plasma was thawed at 37 °C and centrifuged at 3000 rpm for 8 min to remove clots, and the supernatant was used in the experiment. The pH of the plasma was recorded, and only the pH range between 7.4 and 8 was used. The plasma and compound solution were prewarmed to 37 °C. Ten microliters of prewarmed testing compound or reference compound (procaine) solution (20 μM in 0.05 mM sodium phosphate buffer (pH 7.4) with 0.5% BSA) was mixed with 90 μL of plasma at different time points to allow for 5, 15, 30, 45, and 60 min of incubation time. For 0 min, the plasma was mixed with vehicle only. Acetonitrile was added to the compound and plasma mixture to quench the reaction, and the resulting mixture was centrifuged (5594*g* for 15 min). The supernatant was taken and diluted before LC-MS analysis.

### Solubility in Phosphate Buffer and Fed State Simulated Intestinal Fluid (FeSSIF)

Eight microliters of reference or test compound stock solution (10 mM in DMSO) was added to 792 μL of 100 mM phosphate buffer (pH 7.4) or FeSSIF (pH 5.8). The resulting mixture was shaken for 1 h (1000 rpm) at room temperature and then centrifuged for 10 min (12 000 rpm) to remove the undissolved particles. The supernatant was collected and diluted 10 times and 100 times separately with 100 mM phosphate buffer or FeSSIF. Five microliters of the supernatant samples (no diluted, 10 times diluted, 100 times diluted) were mixed with 95 μL of acetonitrile (containing internal standard) separately before injecting into LC-MS/MS for analysis.

### Mouse Liver Microsome Stability

Testing compound (1.5 μL) or reference compound (500 μM in 5% DMSO and 95% acetonitrile) was mixed with 18.75 μL of 20 mg/mL liver microsome (Corning) and 479.75 μL of potassium phosphate buffer (0.1 M potassium phosphate buffer, 1 mM EDTA, pH 7.4). The reaction was started by mixing 30 μL of the above mixture (prewarmed to 37 °C) with 15 μL of 6 mM NADPH stock solution (prewarmed to 37 °C). After incubating for 5, 15, 30, or 45 min, 135 μL of acetonitrile containing internal standard was added to stop the reaction. For 0 min, the compound and microsome mixture were mixed with acetonitrile first before adding NADPH. After quenching, the reaction mixture was centrifuged, and the supernatant was taken and diluted for LC-MS analysis.

### Mouse Hepatocyte Stability

Fifty microliters of prewarmed hepatocytes (2 × 10^6^ cells/mL) in suspension media (Krebs–Henseleit buffer (Sigma-Aldrich) containing 5.6 g/L HEPES) was mixed with 50 μL of prewarmed compound dosing solution (2 μM in Krebs–Henseleit buffer with 1% DMSO). After incubating at 37 °C for 15, 30, 60, or 120 min, 100 μL of acetonitrile containing internal standard was added to quench the reaction. For 0 min incubation, acetonitrile was mixed with hepatocytes first before adding the compound solution. After quenching, the mixture was shaken at a vibrator for 10 min (600 rpm/min) and then sonicated for 2 min before centrifugation (5594*g* for 15 min). The supernatant was taken and diluted for LC-MS analysis.

### Pharmacokinetic (PK) Study

PK profiling was outsourced and undertaken by Shanghai ChemPartner Co., Ltd. All animal experiments performed were conducted in compliance with the Institutional Animal Care and Use Committee (IACUC) and the Office of Laboratory Animal Welfare (OLAW) guidelines. Six to eight week old C57BL/6 male mice purchased from Jihui Laboratory Animal Co., Ltd. were used in the study. XL01126 was formulated in 10% HP-β-CD in 50 mM citrate buffer pH = 3.0 at 1 mg/mL for IV injection and at 3 mg/mL for IP and PO injections. For IV injections, 5 mg/kg of XL01126 was administered into the tail vein. For IP and PO injections, 30 mg/kg of XL01126 was administered *via* the intraperitoneal injection or oral gavage, respectively. The animals were restrained manually at the designated time points (0.083, 0.25, 0.5, 1, 2, 4, and 8 h); approximately, 110 μL of blood sample was collected *via* facial vein into K_2_EDTA tubes. Three mice per time point were used, resulting in a total of 21 mice for each administration route. The blood sample was put on ice and centrifuged at 2000*g* for 5 min to obtain the plasma sample within 15 min. The plasma, brain, and CSF samples were stored at approximately −70 °C until analysis. A 30 μL aliquot of plasma was added with 200 μL of internal standard (Glipozode, 40 ng/mL) in MeCN with 5% Citri. The mixture was then vortexed for 1 min and then centrifuged for 10 min at 5800 rpm. The supernatant (100 μL) was transferred to a new plate. The solution (1 μL) was injected into LC-MS/MS. LC-MS/MS instrument used: SCIEX LC-MS/MS-49 (Triple Quad 6500+). Data were analyzed by WinNonLin and Microsoft Excel.

## Supporting Information Available

First-generation compound structures, synthetic procedures for the first- and the second-generation PROTACs, and the LRRK2 and VHL tracers, abbreviations used, figures, and compound characterizations (PDF)

## Notes

Mass spectrometry data: The mass spectrometry raw data and MaxQuant search output tables are deposited in ProteomeXchange, PRIDE database: https://www.ebi.ac.uk/pride/archive/. Unique identifier: PXD034055.
