# Supplementary material for: Discovery of XL01126: A Potent, Fast, Cooperative, Selective, Orally Bioavailable, and Blood–Brain Barrier Penetrant PROTAC Degrader of Leucine-Rich Repeat Kinase 2
Source: J Am Chem Soc. 2022 Aug 25;144(37):16930–52. doi: 10.1021/jacs.2c05499 (PMC9501899; doi:10.1021/jacs.2c05499)
Supplement: Supplementary file 1 — ja2c05499_si_001.pdf [file ja2c05499_si_001.pdf]

Supporting information for

Discovery of XL01126: A Potent, Fast, Cooperative, Selective, Orally Bioavailable and Blood-Brain Barrier Penetrant PROTAC Degrader of Leucine-Rich Repeat Kinase 2

Xingui Liu[†,‡], Alexia F. Kalogeropulou[§,‡], Sofia Domingos[†], Nikolai Makukhin[†,€], Raja S. Nirujogi[§], Francois Singh[§], Natalia Shpiro[§], Anton Saalfrank[§], Esther Sammler[§], Ian G. Ganley[§], Rui Moreira[||], Dario R. Alessi[§,*], Alessio Ciulli[†,*]

[†]Centre for Targeted Protein Degradation, Division of Biological Chemistry and Drug Discovery, School of Life Sciences, University of Dundee, Dow Street, Dundee DD1 5EH, United Kingdom

[§]Medical Research Council (MRC) Protein Phosphorylation and Ubiquitylation Unit, School of Life Sciences, University of Dundee, Dow Street, Dundee DD1 5EH, United Kingdom

[||]Research Institute for Medicines (iMed.ULisboa), Faculty of Pharmacy, Universidade de Lisboa, Av. Prof. Gama Pinto, 1649-003, Lisboa, Portugal

[€]Current address: AstraZeneca, Oncology R&D, Tumour Targeted Delivery, QMB Innovation Centre, 42 New Road, London, E1 2AX, United Kingdom

[‡]Co-first author

*Corresponding authors

Table of Contents

Figure S1. Structures of the first generation LRRK2 PROTAC degraders ... 3

Figure S2. Screening of the first generation PROTACs in WT and G2019S LRRK2 MEFs. ... 4

Figure S3. Screening of the second generation PROTACs in WT MEFs and G2019S LRRK2 MEFs. ... 5

Figure S4. HG-10-102-01, SD75, XL01126, and XL01134 inhibit the kinase activity of WT and G2019S LRRK2. ... 5

Figure S5. XL01126 surpassed its warhead and negative PROTAC cis-XL01126 in inhibiting downstream signaling on WT LRRK2 MEFs. ... 6

Figure S6. Dose-dependent assay of XL01126 and cis-XL01126 on LRRK2 R1441C MEFs. ... 6

Figure S7. XL01126-mediated degradation of LRRK2 in SH-SY5Y human neuroblastoma cell line. ... 7

Figure S8. Validation of XL01126-mediated degradation of LRRK2 in total proteome samples and confirmation of off-target PDE6D degradation by XL01126 in LRRK2 KO MEFs. ... 7

Figure S9. LRRK2 degradation by XL01126 is dependent on the ubiquitin proteasome system. ... 8

Figure S10. Stability of XL01126 in mouse plasma, mouse liver microsome, and mouse hepatocytes in comparison with control drugs. ... 8

Figure S11. Degradation of LRRK2 in the presence and absence of 10% FBS in WT and G2019S LRRK2 MEFs. ... 9

In vitro LRRK2 kinase assay: IC_{50} determination ... 9

Immunoprecipitation of LRRK2 ... 9

Abbreviations used ... 10

Chemistry ... 10

Scheme S1. Synthesis of VHL ligands ... 11

Scheme S2. Synthesis of *cis*- and *trans*-1,4-bis(bromomethyl)cyclohexane ... 17

Scheme S3. Synthesis of SD74, SD75, SD82, SD100, XL01078B, XL01072, XL01078B, XL01119, XL01118, XL01120, XL0123, XL01122, XL01121, XL01131, XL01111, XL01134 ... 18

Scheme S4. Synthesis of SD10 and SD11 ... 34

Scheme S5. Synthesis of SD112, SD113, SD114, SD12, SD13, and SD79 ... 36

Scheme S6. Synthesis of XL01140 ... 39

Scheme S7. Synthesis of XL01145 ... 41

Scheme S8. Synthesis of XL01149 ... 44

Scheme S9. Synthesis of XL01168 ... 47

Scheme S10. Synthesis of VHL NanoBRET tracer ... 49

SCheme S11. Synthesis of LRRK2 NanoBRET tracer ... 53

REFERENCES ... 55

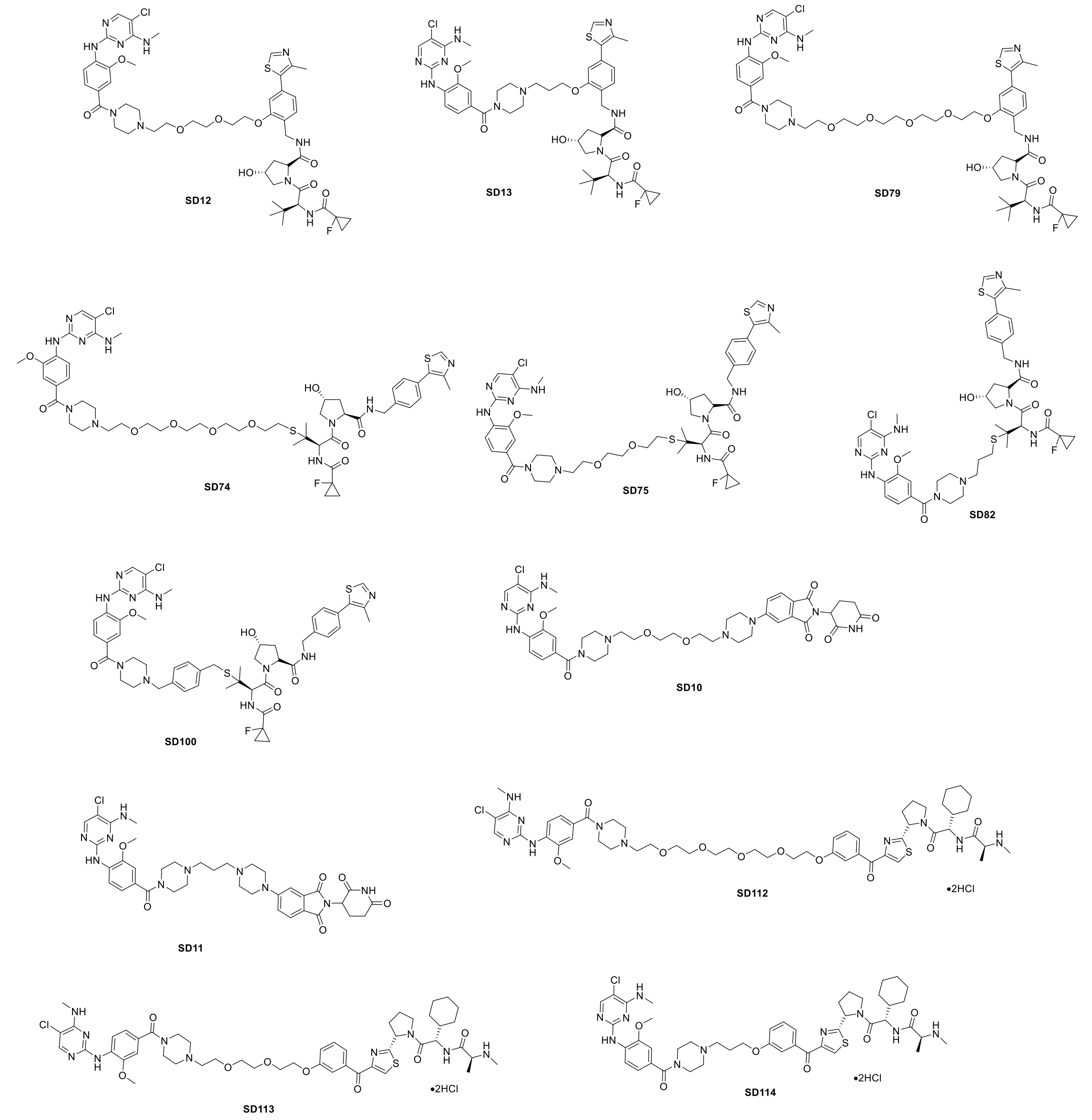

Figure S1. Structures of the first generation LRRK2 PROTAC degraders

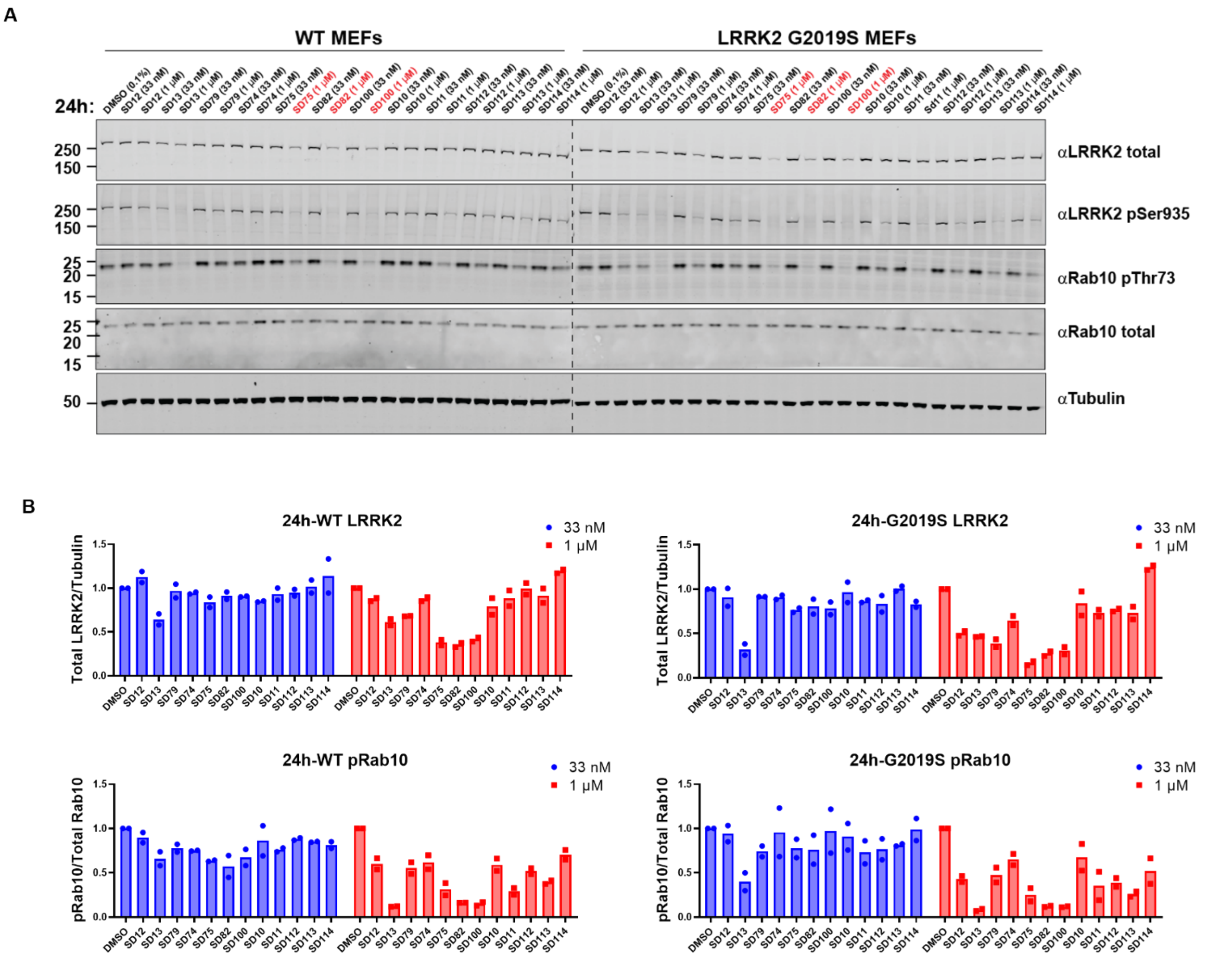

Figure S2. Screening of the first generation PROTACs in WT and G2019S LRRK2 MEFs. (A) Representative Western blots monitoring total LRRK2, LRRK2-pSer935, Rab10-pThr73, total Rab10, and Tubulin levels after treating WT and G2019S MEFs with the indicated compounds at 33 nM, 1 μM, or DMSO for 24h. (B) Quantitative analysis of the relative LRRK2 and Rab10-pThr73 levels, which are presented as ratios of total LRRK2/Tubulin or Rab10-pThr73/total Rab10, normalized to the ratios of DMSO treated sample. Data were obtained from two biological independent experiments.

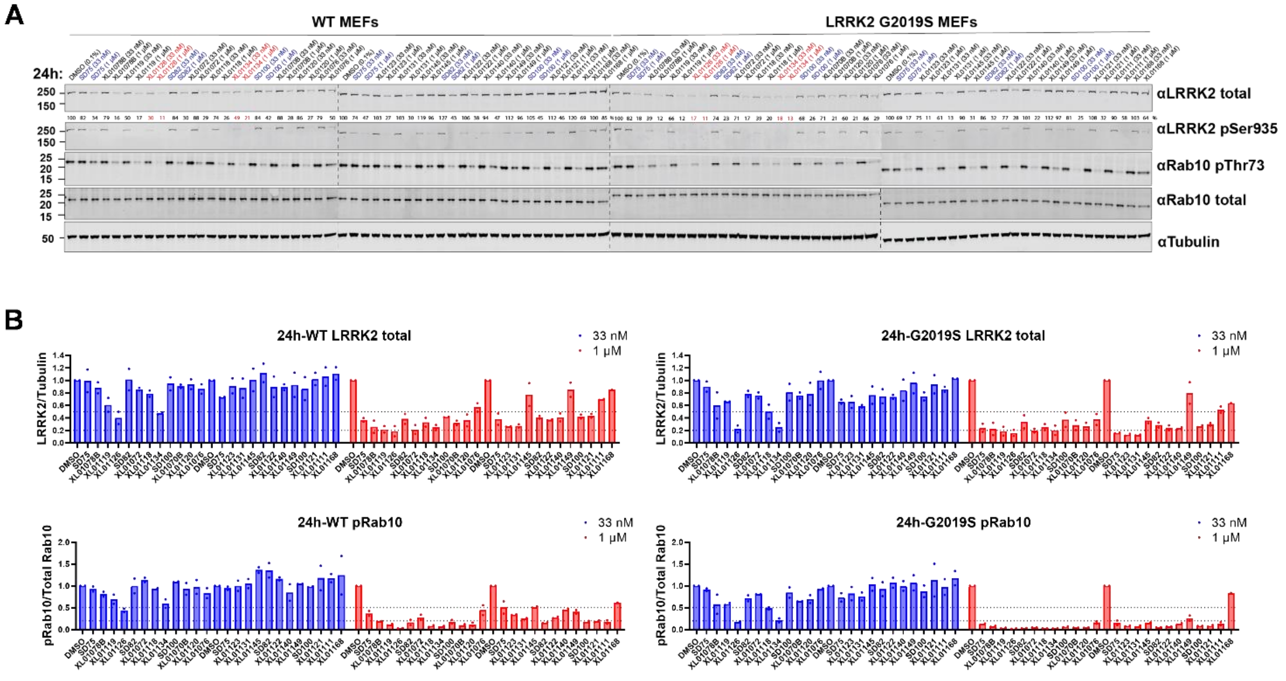

Figure S3. Screening of the second generation PROTACs in WT MEFs and G2019S LRRK2 MEFs. (A) Representative Western blots monitoring total LRRK2, LRRK2-pSer935, Rab10-pThr73, total Rab10, and Tubulin levels after treating WT and G2019S MEFs with the indicated compounds at 33 nM, 1 μM, or DMSO for 24h. (B) Quantitative analysis of the relative LRRK2 and Rab10-pThr73 levels, which are presented as ratios of total LRRK2/Tubulin or Rab10-pThr73/total Rab10, normalized to the ratios of DMSO treated sample. Data were obtained from two biological independent experiments.

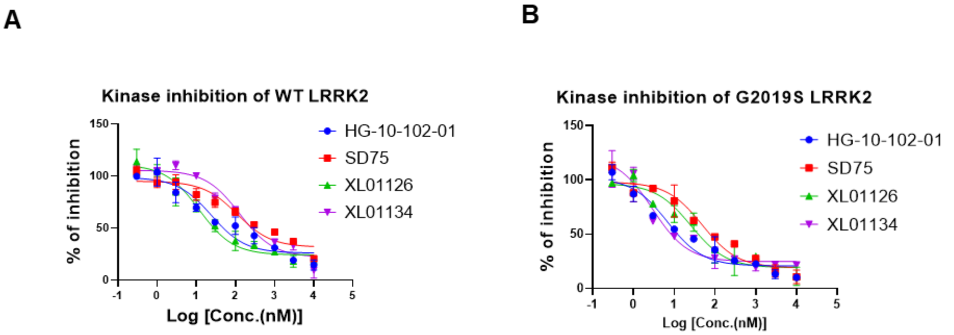

	HG-10-102-01	SD75	XL01126	XL01134
IC_{50} on WT LRRK2 (nM)	24.0 ± 3.1	110.7 ± 7.8	12.4 ± 2.9	139.7 ± 57.8
IC_{50} on G2019S LRRK2 (nM)	7.1 ± 0.9	48.5 ± 1.0	24.3 ± 6.0	3.2 ± 1.9

Figure S4. HG-10-102-01, SD75, XL01126, and XL01134 inhibit the kinase activity of WT and G2019S LRRK2.

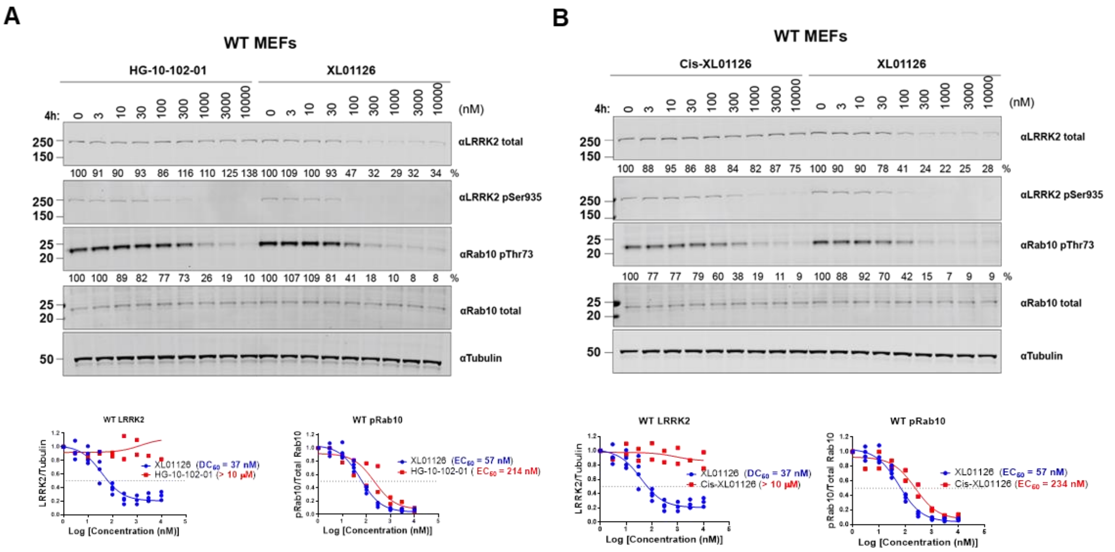

Figure S5. XL01126 surpassed its warhead and negative PROTAC cis-XL01126 in inhibiting downstream signaling on WT LRRK2 MEFs. Representative Western blotting of total LRRK2, LRRK2-pSer935, pRab10, Rab10 total, and Tubulin levels following the treatment of G2019S LRRK2 MEFs with HG-10-102-01 (A), XL01126 (A and B), and *cis*-XL01126 (B) at the indicated concentrations for 4h. The relative LRRK2 protein and pRab10 levels were obtained by quantifying the ratios of total LRRK2/Tubulin or Rab10-pThr73/total Rab10, respectively, and the ratios were normalized to the DMSO treated samples. The relative LRRK2 and pRab10 protein levels were plotted against the compounds concentration and fitted against "non-linear regression, one site-fit LogIC_{50}" in GraphPad to obtain the DC_{50} and EC_{50} values. Data were obtained from two independent biological experiments.

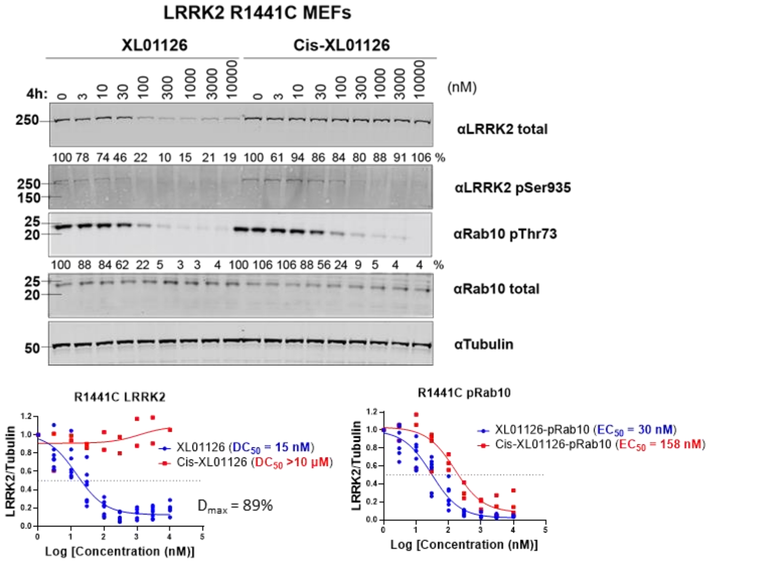

Figure S6. Dose-dependent assay of XL01126 and cis-XL01126 on LRRK2 R1441C MEFs. Representative Western blots of total LRRK2, LRRK2-pSer935, pRab10, Rab10 total, and Tubulin levels after treating the R1441C LRRK2 MEFs with XL01126 and *cis*-XL01126 at the indicated concentrations for 4h. The relative LRRK2 protein and pRab10 levels were obtained by quantifying the ratios of total LRRK2/Tubulin or Rab10-pThr73/total Rab10, respectively, and the ratios were normalized to the DMSO treated samples. The relative total LRRK2 and pRab10 levels were plotted against the compounds concentration and fitted against "non-linear regression, one site-fit LogIC_{50}" in GraphPad to obtain the DC_{50} and EC_{50} values. Data were obtained from two to three biological independent experiments.

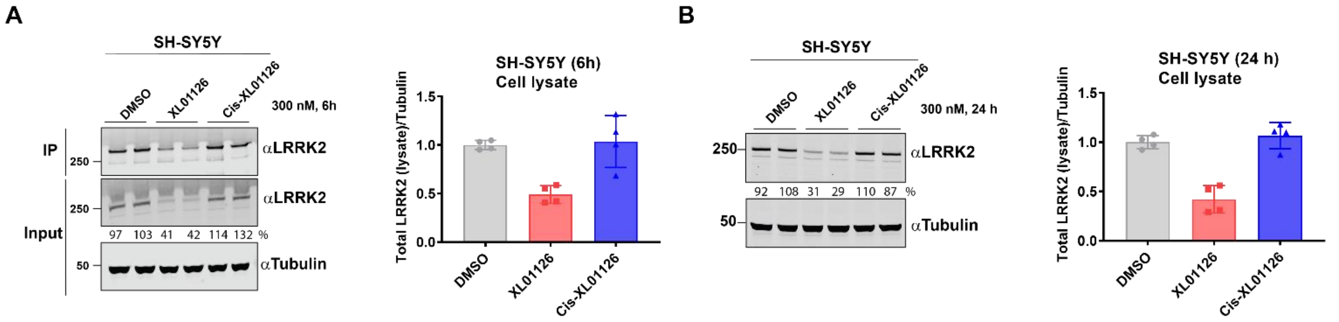

Figure S7. XL01126-mediated degradation of LRRK2 in SH-SY5Y human neuroblastoma cell line. SH-SY5Y cells were treated with vehicle (DMSO), 300 nM XL01126 or cis-XL01126 for 6 hours (A) or 24 hours (B). LRRK2 was immunoprecipitated from 0.5 mg cell lysate (A). LRRK2 immunoprecipitates and cell lysates were subjected to Western blot analysis with antibodies detecting total LRRK2 and Tubulin. Each lane represents a different dish of cells. The relative LRRK2 protein levels in cell lysate were obtained by quantifying the ratios of total LRRK2/Tubulin, and were normalized to the DMSO treated samples. Data are quantified as mean ± SD from two biological replicates.

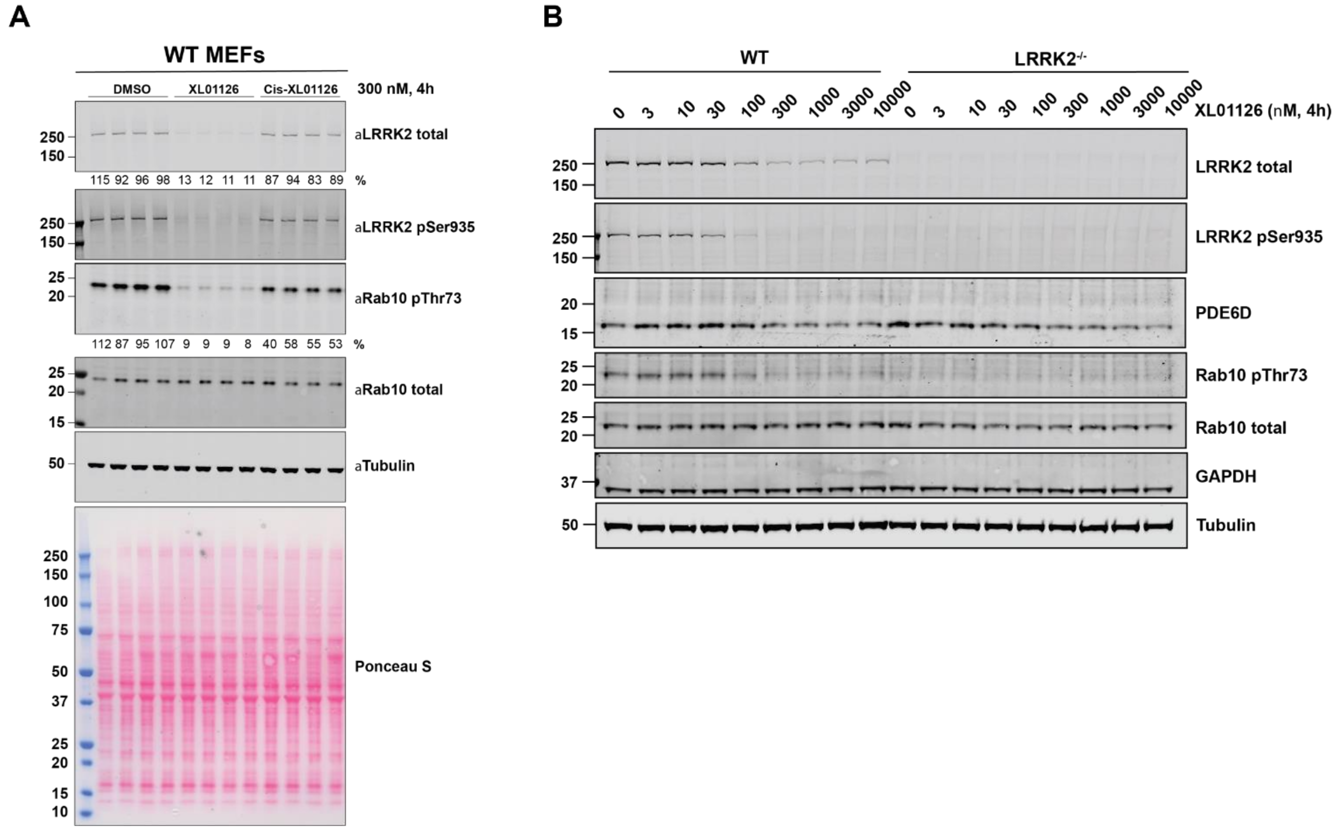

Figure S8. Validation of XL01126-mediated degradation of LRRK2 in total proteome samples and confirmation of off-target PDE6D degradation by XL01126 in LRRK2 KO MEFs. (A) WT MEFs were treated with 300 nM of XL01126, *cis*-XL01126 or DMSO for 4h prior to lysis. The cell lysates were analyzed by quantitative Western blotting with the indicated antibodies. Ponceau S staining shows the intracellular protein levels. The same samples were used for TMT-labeling proteomic studies outlined in Figure. 11. (B). WT and LRRK2 KO MEFs were treated with XL01126 at the indicated concentrations for 4h and the levels of total LRRK2, LRRK2-pSer935, PDE6D, pRab10, total Rab10, Tubulin and GADPH were analyzed by Western blotting.

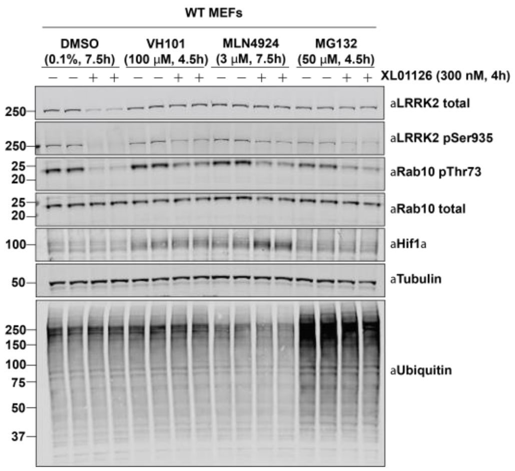

Figure S9. LRRK2 degradation by XL01126 is dependent on the ubiquitin proteasome system. WT MEFs were pre-treated with DMSO, VH101, MLN4924, or MG132 for the indicated periods of time, followed by DMSO or 300 nM XL01126 for 4h. Each replicate represents a different dish of cells. Cell lysate was analyzed by Western blotting for total LRRK2, LRRK2-pSer935, pRab10, total Rab10, HIF1α, Tubulin, and Ubiquitin.

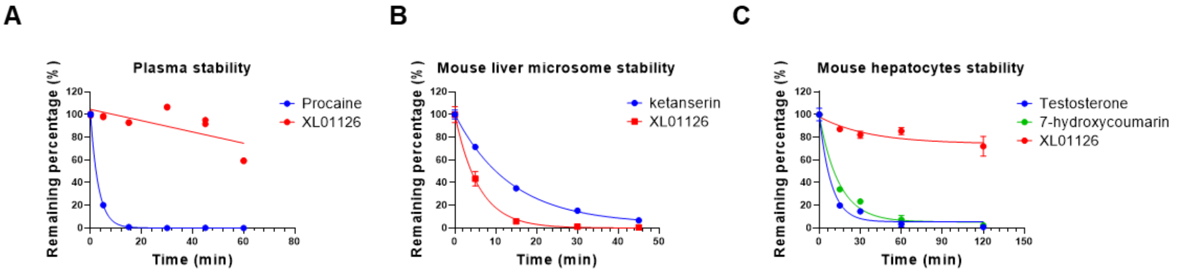

Test Article	$T_{1/2}$ (minute)
Procaine	2.17
XL01126	108.29

Test Article	$T_{1/2}$ (minute)	Cl_{int} (mL/min/Kg)	Comments
ketanserin	11.68	467.05	Fast metabolism (<30 min)
XL01126	3.65	1494.62	Fast metabolism (<30 min)

Test Article	$T_{1/2}$ (minute)	Cl_{int} (mL/min/kg)
Testosterone	6.44	1270.33
7-hydroxycoumarin	9.67	846.47
XL01126	314.33	26.04

Figure S10. Stability of XL01126 in mouse plasma, mouse liver microsome, and mouse hepatocytes in comparison with control drugs. (A) Percentage of XL01126 and procaine (control) remaining after 0, 5, 15, 30, 45, and 60 min in mouse plasma at 37 °C, normalized to 0 min time point, with two independent replicates per time point. $T_{1/2}$ was calculated from K ($T_{1/2}$=0.693/K) which is the rate constant from a plot of ln [concentration] vs. incubation time. (B) Percentage of XL01126 and ketanserin (control) remaining after 0, 5, 15, 30, and 45 min in mouse liver microsome at 37 °C, normalized to 0 min time point, with two independent replicates per time point. $T_{1/2}$ = 0.693/K (K is the rate constant from a plot of ln [concentration] vs. incubation time). Cl_{int} = (0.693/$T_{1/2}$) × (1/(microsomal protein concentration (0.5 mg/mL))) × Scaling Factors, where Scaling Factor = (microsomal protein per gram of liver) × (liver weight per kilogram of body weight). (C) Percentage of XL01126, Testosterone (control), and 7-hydroxycoumarin (control) remaining after 0, 15, 30, 60, and 120 min in mouse hepatocytes at 37 °C, normalized to 0 min time point, with two independent replicates per time point. $T_{1/2}$ = 0.693/K (K is the rate constant from a plot of ln [concentration] vs. incubation time). Clint = (0.693/$T_{1/2}$) × (1/hepatocyte density) × Scaling Factors, where Scaling Factor = (Hepatocellularity) × (Liver weight).

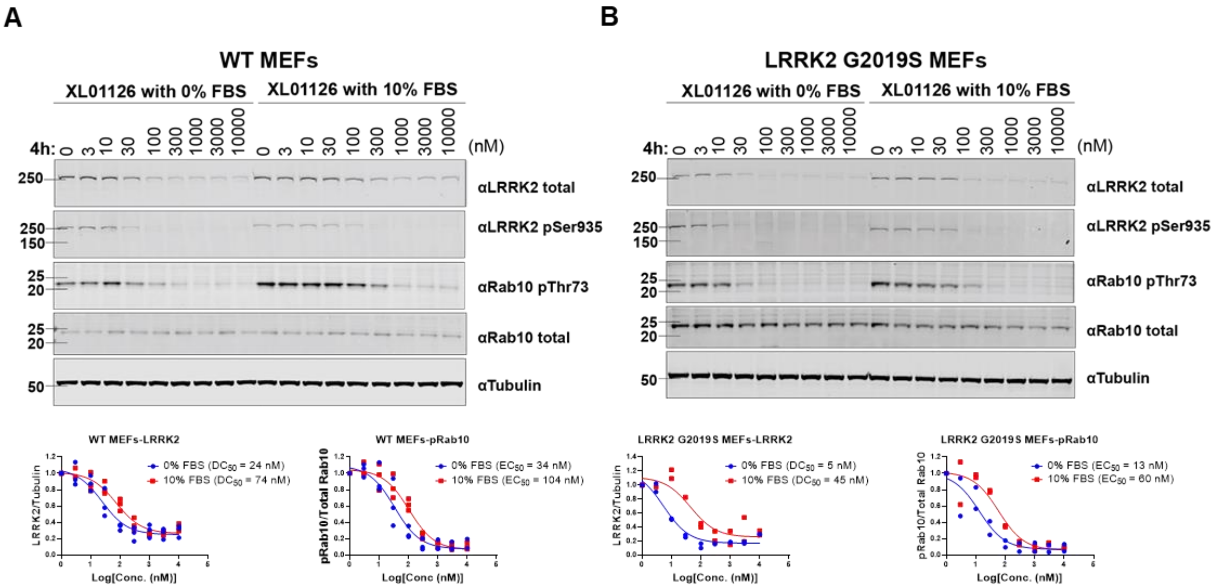

Figure S11. Degradation of LRRK2 in the presence and absence of 10% FBS in WT and G2019S LRRK2 MEFs. Representative western blotting of total LRRK2, LRRK2-pSer935, pRab10-Thr73, Rab10 total, and Tubulin in WT MEFs (A) and G2019S LRRK2 MEFs (B) following treatment with XL01126 (with FBS removed (0%) or added (10%) in the culture media) at the indicated concentrations for 4h. The relativeLRRK2 protein and pRab10 levels were obtained by quantifying the ratios of total LRRK2/Tubulin or Rab10-pThr73/total Rab10, respectively, and the ratios were normalized to the ratios of DMSO treated samples. The relative LRRK2 and pRab10 were plotted against compounds' concentration and fitted into "non-linear progression, one site-LogIC50" to obtain the DC_{50} values against LRRK2 and EC_{50} values against pRab10. Data were obtained from two biological independent experiments.

In vitro LRRK2 kinase assay: IC50 determination

The *in vitro* kinase activity of LRRK2 was assessed by MRC PPU Reagents and Services [https://www.kinase-screen.mrc.ac.uk/services/ic50] using a method that has been described previously[1].To determine the IC_{50} of the tested compounds, peptide kinase assays were carried out with GST-LRRK2 WT or GST-LRRK2 [G2019S] at a linear range concentration and 0.2 mM Nictide peptide substrate [RLGWWRFYTLRRARQGNTKQR] in a 25.5 µl reaction volume containing 50 mM Tris-HCl pH 7.5, 0.1 mM EGTA, 10 mM $MgCl_2$, 2 mM DTT, 0.1 mM [γ- ^{33}P]ATP (~300-600 cpm/pmol), and the indicated concentrations of inhibitor or PROTAC dissolved in DMSO. The kinase reactions were incubated for 30 minutes at room temperature, then were terminated by applying the reaction mixture to P81 phosphocellulose filter papers followed by immersion in 50 mM phosphoric acid. After extensive washing, reaction products were quantified by Cerenkov counting. IC_{50} values were calculated using GraphPad Prism software (non-linear regression analysis).

Immunoprecipitation of LRRK2

To assess LRRK2 degradation in SH-SY5Y cells, LRRK2 was immunoprecipitated using anti-LRRK2 (UDD3) coupled to protein A/G Sepharose beads at a ratio of 1 µg of antibody per 1 µl beads. LRRK2 was immunoprecipitated from 0.5 mg cell lysate using 10 µl antibody coupled beads for one hour at 4°C. Immunoprecipitated LRRK2 was washed three times with ice-cold buffer (50 mM Tris-HCl, pH 7.5, 270 mM sucrose, 1 mM EGTA, 50 mM NaF, 10 mM 2-glycerophosphate, 5 mM sodium pyrophosphate) supplemented with 300 mM NaCl, and twice with 50 mM Tris-HCl pH 7.5. The beads were resuspended in 2X NuPAGE LDS sample buffer and heated at 70°C for 15 minutes. The bead slurry was then centrifuged through a 0.22 µm Spinex filter and eluate was supplemented with β-mercaptoethanol. The eluate was heated at 95°C for 5 minutes, and subjected to western blot analysis with a LRRK2 antibody (NeuroMab, clone N241A/34).

Abbreviations used

LRRK2, Leucine Rich Repeat Kinase 2; PROTACs, Proteolysis Targeting Chimeras; MEFs, mouse embryonic fibroblasts; WT, wildtype; PD, Parkinson's disease; GTPase, guanosine triphosphatase; VHL, von Hippel-Lindau; CRBN, Cereblon, cIAP, Cellular Inhibitor of Apoptosis; PBMC peripheral blood mononuclear cells; BMDM, bone marrow derived macrophages; VCB, VHL complexed with elongin B and elongin C; FeSSIF, Fed State Simulated Intestinal Fluid; IV, intravenous; IP, intraperitoneal; PO, Per oral; CSF, cerebrospinal fluid; CNS, central nervous system; PK, Pharmacokinetic; PBS, Phosphate-Buffered Saline; EGTA, Ethylene glycol tetraacetic acid; EDTA, Ethylenediaminetetraacetic acid; LDS, Lithium dodecyl sulfate; TBS-T, tris-buffered saline with tween 20; TCEP, Tris(2-carboxyethyl)phosphine; TEABC, triethylammonium bicarbonate; IAA, iodoacetamide; SDS, Sodium dodecyl sulfate; TMT, Tandem Mass Tag; TEAB, Tetraethylammonium bromide; FP, Fluorescence polarization; HATU, (1-[Bis(dimethylamino)methylene]-1H-1,2,3-triazolo[4,5-b]pyridinium 3-oxide hexafluorophosphate; TEA, triethyl amine; TFA, trifluoroacetic acid; DMF, Dimethylformamide; DCM, methylene chloride; EDCI, 1-Ethyl-3-(3-dimethylaminopropyl)carbodiimide; DIPEA, N,N-Diisopropylethylamine; DBU, 1,8-Diazabicyclo(5.4.0)undec-7-ene; THF, Tetrahydrofuran; EtOAc, ethyl acetate; DMSO, dimethyl sulfoxide, $CDCl_3$, deuterated chloroform; PE, Petroleum ether; DMA, Dimethylacetamide; HOBt, Hydroxybenzotriazole; PPh_3, Triphenylphosphine; CBr_4, Carbon tetrabromide; PyOxim, [Ethyl cyano(hydroxyimino)acetato-O2]tri-1-pyrrolidinylphosphonium hexafluorophosphate; MW, microwave; DIAD, Diisopropyl azodicarboxylate; NHS, N-Hydroxysuccinimide; TIPS, Triisopropyl silane.

Chemistry

Chemicals that are commercially available were purchased from Apollo Scientific, Sigma-Aldrich, Fluorochem, and Enamine and were used without further purification. All solvents use for reactions are anhydrous. LC-MS was carried out on Shimadzu HPLC/MS 2020 equipped with a Hypersil Gold column (1.9 μm 50 × 2.1 mm), photodiode array detector and ESI detector. The samples were eluted with a 3 min gradient of 5-95% acetonitrile in water containing 0.1% formic acid at a flow rate of 0.8 mL/min. Flash column chromatography was performed on Teledyne ISCO Combiflash Companion installed with disposable normal phase RediSep Rf columns (230-400 mesh, 40-63 mm; SiliCycle). Preparative HPLC purification was performed on Gilson Preparative HPLC system equipped with a Waters X-Bridge C18 column (100 mm × 19 mm and 5 μm particle size) using a gradient from 5 to 95% of acetonitrile in water containing 0.1% formic acid over 10 min at a flow rate of 25 mL/min. Compound characterization using NMR was performed either on a Bruker 500 Ultra shield or on a Bruker Ascend 400 spectrometer. The ^{1}H NMR, ^{13}C NMR and ^{19}F NMR reference solvents used are $CDCl_3$ -d_1 (δH = 7.26 ppm/δC = 77.16 ppm), CD_3OD-d_4 (δH = 3.31 ppm/δC = 49.00 ppm) or DMSO-d_6 (δH = 2.50 ppm/δC = 39.52 ppm). Signal patterns are described as singlet (s), doublet (d), triplet (t), quartet (q), quintet (quint.), multiplet (m), broad (br), or a combination of the listed splitting patterns. The coupling constants (J) are measured in hertz (Hz). HRMS was performed on a Bruker MicroTOF II focus ESI Mass Spectrometer connected in parallel to a Dionex Ultimate 3000 RSLC system with diode array detector and a Waters XBridge C18 column (50 mm × 2.1, 3.5 μm particle size). All final compounds are >95% pure by HPLC.

***General procedure** 1 (Boc protection)* To a solution of benzyl amine compound (1 eq) and TEA (2 eq) in DCM was added Boc_2O (1.1 eq) at 0 °C, the resulting mixture was stirred at room temperature for 4h or overnight. DCM was added and the mixture was washed with 1N HCl aqueous, saturated $NaHCO_3$ aqueous, water and brine separately. The collected organic phase was then dried over sodium sulfate, filtered, and condensed under reduced pressure to afford the Boc protected compounds.

***General procedure 2** ($Pd(OAc)_2$ mediated coupling)* Under nitrogen protection, $Pd(OAc)_2$ (0.1 eq) was added to a mixture of bromide compound (1 eq), KOAc (2 eq), and 4-methylthiazole (2 eq) in DMA. The resulting mixture was heated to 110 °C and stirred at this temperature for overnight. The reaction mixture was then diluted with EtOAc and filtered through a pad of celite. The collected filtrate was washed with

water and brine, dried over sodium sulfate, filtered, and condensed under reduced pressure to afford a residue which was purified by flash column chromatography on silica (0% -100% EtOAc in heptane) to afford the desired compounds.

General procedure 3 *(Boc deprotection and condensation with Boc protected hydroxyl proline)* To a solution of Boc protected (1 eq) compound in DCM (same volume as 4N HCl in dioxane) was added 4N HCl in dioxane (8 eq). The resulting mixture was stirred at room temperature for 1h and then condensed to give a solid which was washed with diethyl ether and dried under reduced pressure to afford the amine intermediate as a HCl salt. The salt was suspended in DCM, followed by the addition of TEA (4 eq), (2*S*,4*R*)-1-(*tert*-Butoxycarbonyl)-4-hydroxypyrrolidine-2-carboxylic acid (1 eq), and HATU (1.05 eq). After stirring at room temperature for 2h, the resulting mixture was diluted with DCM, washed with water and brine, dried over sodium sulfate, filtered, and condensed to give a residue which was purified by flash column chromatography on silica (0% -100% EtOAc in heptane) to afford the desired compounds.

General procedure 4 *(Boc deprotection and condensation with Fmoc-S-trityl-L-penicillamine)* To a stirring solution of Boc protected substrate (1 eq) in DCM was added 4N HCl in dioxane (same volume as DCM, 10 eq). After stirring at room temperature for 1h, the mixture was condensed under reduced pressure to afford a solid which was washed with diethyl ethyl to give light yellow solid as HCl salt quantitatively. The solid (1 eq) was dissolved in DMF, TEA (1.5 eq) was added to neutralize the HCl, followed by the addition of a mixed solution of Fmoc-*S*-trityl-L-penicillamine (1 eq), HATU (1.1 eq), and TEA (2 eq) in DMF dropwise at 0 °C. The resulting mixture was stirred at 0 °C for 2h then added with EtOAc and water. The water phase was extracted with EtOAc (3×) and the combined organic phase was washed with water, brine, dried over sodium sulfate, filtered and condensed to afford a residue which was purified by flash column chromatography on silica (0%-100% EtOAc in heptane) to afford the desired compound.

General procedure 5 *(Fmoc deprotection)* To a solution of Fmoc protected compound (1 eq) in DCM was added piperidine (5 eq) dropwise. The resulting mixture was stirred at room temperature for 2h, and then condensed under reduced pressure to give a residue which was purified by flash column chromatography on silica gel (0%-10% methanol in DCM with 0.7 M ammonia) to give the desired product.

General procedure 6 *(condensation with 1-fluorocyclopropane-1-carboxylic acid)* To a solution of amine compound (1 eq), TEA (2 eq), and HATU (1.05 eq) in DMF was added 1-fluorocyclopropane-1-carboxylic acid (1 eq). After stirring at room temperature overnight, the mixture was added with water, extracted with EtOAc (3×), and the combined organic phase was washed with water and brine separately, dried over sodium sulfate, filtered and condensed to give a residue which was purified by flash column chromatography on silica gel (0%-100% EtOAc in PE) to give the desired product.

General procedure 7 *(Trityl group deprotection)* To a solution of trityl protected VHL ligand (1 eq) in DCM was added Triisopropyl silane (TIPS, 10 eq) and trifluoroacetic acid (10 eq) at 0 °C. After stirring at 0 °C for 10 min, the mixture was concentrated under vacuum to give a residue which was purified by flash column chromatography on silica gel (0%-10% methanol in DCM) to give desired thiol compound.

General procedure 8 (*Linker attachment*) To a suspension of compound **3-5a** or **3-5b** in acetone was added K_2CO_3 (5 eq) and dibromide or di-tosylate compound (3-5 eq). After stirring at room temperature or 50 °C overnight, the mixture was diluted with DCM, washed with water and brine, dried over sodium sulfate, filtered and condensed to give a residue which was purified by flash column chromatography on silica gel to give desired product.

General procedure 9 (*Substitution reaction with thiol compound*) To a solution of tosylated or bromide compound (1 eq) in THF was added thiol compound (1 eq) and DBU (6 eq). After stirring at room temperature overnight, the reaction mixture was condensed to afford a residue which was purified on preparative HPLC under acidic condition (5-95 % CH_3CN in 0.1 % aq. HCO_2H) to give desired compound.

Scheme S1. Synthesis of VHL ligands

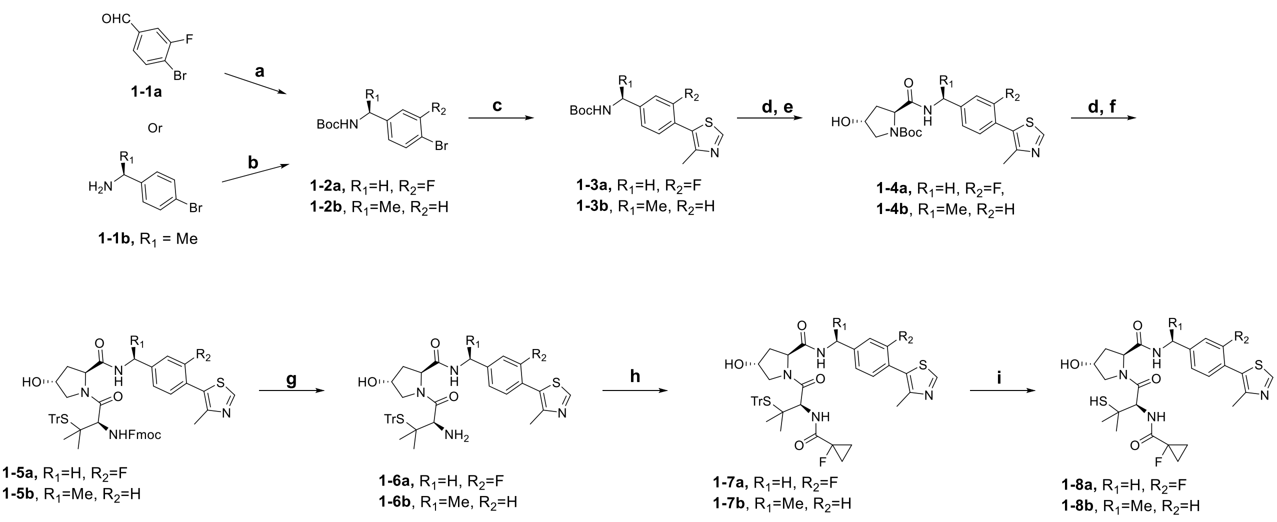

Reagents and conditions: a. *tert*-butyl carbamate, Et_3SiH, TFA, DCM/acetonitrile; b. Boc_2O, TEA, DCM, 4h; c. 4-methyl thiazole, $Pd(OAc)_2$, KOAc, DMA, 110 °C, overnight; d. 2N HCl in Dioxane and DCM, e. (2*S*,4*R*)-1-(tert-Butoxycarbonyl)-4-hydroxypyrrolidine-2-carboxylic acid, HATU, TEA, DCM; f. Fmoc-*S*-trityl-L-penicillamine, HATU, TEA, DMF, 0 °C; g. Piperidine, DCM; h. 1-fluorocyclopropane-1-carboxylic acid, HATU, TEA, DCM; i. TFA, triisopropylsilane, DCM.

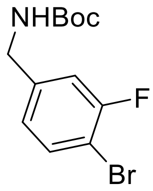

tert-butyl (4-bromo-3-fluorobenzyl)carbamate (**1-2a**)

Trifluoroacetic acid (0.754 ml, 9.9 mmol) was added to a solution of 4-bromo-3-fluorobenzaldehyde (1g, 4.9 mmol), tert-butyl carbamate (1.73 g, 14.8 mmol) and triethylsilane (1.72g, 14.8 mmol) in DCM and acetonitrile (10 ml DCM mixed with 30 ml acetonitrile). After stirring at room temperature overnight, the mixture was diluted with DCM, washed with water and brine separately, dried over sodium sulfate, filtered, and condensed to give a residue which was purified via flash column chromatography on silica gel (0%-10% ethyl acetate in heptane) to give the title compound (710 mg, 48% yield) as white solid. ^{1}H NMR (400 MHz, $CDCl_3$) δ 7.48 (dd, *J* = 8.1, 7.2 Hz, 1H), 7.05 (dd, *J* = 9.3, 1.9 Hz, 1H), 6.94 (dd, *J* = 8.2, 1.4 Hz, 1H), 4.91 (s, 1H), 4.26 (d, *J* = 5.9 Hz, 2H), 1.45 (s, 9H). ^{13}C NMR (101 MHz, $CDCl_3$) δ 159.31 (d, *J* = 246.4 Hz), 155.93, 141.26 (d, *J* = 6.2 Hz), 133.70, 124.14, 115.47 (d, *J* = 22.7Hz), 107.65 (d, *J* = 20.8 Hz), 80.12, 43.89, 28.51. LC-MS, ESI$^+$, m/z 247.7 [M-56+H]$^+$, 249.7 [M+2-56+H]$^+$.

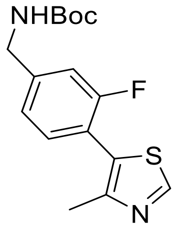

tert-butyl (3-fluoro-4-(4-methylthiazol-5-yl)benzyl)carbamate (**1-3a**)

550 mg **1-3a** was prepared from 865 mg **1-2a** following general procedure 2 (60% yield).^{1}H NMR (400 MHz, $CDCl_3$) δ 8.73 (s, 1H), 7.33 – 7.27 (m, 1H), 7.14 – 7.05 (m, 2H), 5.14 (s, 1H), 4.33 (d, *J* = 5.8 Hz, 2H), 2.40 (d, *J* = 1.2 Hz, 3H), 1.45 (s, 9H). ^{13}C NMR (100 MHz, $CDCl_3$) δ 159.79 (d, *J* = 248.2 Hz), 155.93, 151.66, 150.91, 142.40 (d, *J* = 7.12 Hz), 132.21 (d, *J* = 2.66 Hz), 124.36, 122.89 (d, *J* = 2.81 Hz), 118.30 (d, *J* = 15.67 Hz), 114.80 (d, *J* = 22.81 Hz), 79.85, 43.89, 28.38, 15.92 (d, *J* = 2.59 Hz). LC-MS, m/z 323.3 [M+H]$^+$.

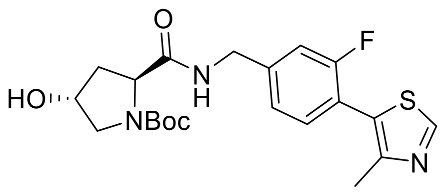

tert-butyl (2*S*,4*R*)-2-((3-fluoro-4-(4-methylthiazol-5-yl)benzyl)carbamoyl)-4-hydroxypyrrolidine-1-carboxylate (**1-4a**)

550 mg **1-4a** was prepared from 502 mg **1-3a** following general procedure 3 (81% yield). ^{1}H NMR (400 MHz, DMSO-d_6) δ 9.09 (s, 1H), 8.51 (t, *J* = 6.0 Hz, 1H), 7.47 – 7.37 (m, 1H), 7.34 – 7.18 (m, 2H), 5.02 – 4.94 (m, 1H), 4.40 – 4.14 (m, 4H), 3.49 – 3.37 (m, 1H), 3.31 (d, *J* = 10.8 Hz, 1H), 2.32 (s, 3H), 2.16 – 2.00 (m, 1H), 1.91 – 1.82 (m, 1H), 1.41 (s, 3H), 1.26 (s, 6H). LC-MS, ESI$^+$, m/z 436.5 [M+H]$^+$.

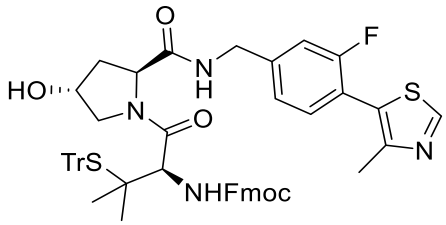

(9H-fluoren-9-yl)methyl ((*R*)-1-((2*S*,4*R*)-2-((3-fluoro-4-(4-methylthiazol-5-yl)benzyl)carbamoyl)-4-hydroxypyrrolidin-1-yl)-3-methyl-1-oxo-3-(tritylthio)butan-2-yl)carbamate (**1-5a**)

104 mg **1-5a** was prepared from 118.6 mg **1-4a** following general procedure 4 (41% yield). ^{1}H NMR (400 MHz, $CDCl_3$) δ 8.79 (s, 1H), 7.77 – 7.72 (m, 2H), 7.61 – 7.47 (m, 8H), 7.40 – 7.33 (m, 3H), 7.26 – 7.15 (m, 12H), 7.07 – 6.96 (m, 2H), 5.64 (d, *J* = 5.8 Hz, 1H), 4.67 (t, *J* = 8.0 Hz, 1H), 4.45 – 4.24 (m, 4H), 4.23 – 4.13 (m, *J* = 18.5, 6.6 Hz, 2H), 3.68 (d, *J* = 5.9 Hz, 1H), 3.52 (d, *J* = 11.4 Hz, 1H), 3.31 (dd, *J* = 11.4, 3.6 Hz, 1H), 2.77 (s, 1H), 2.42 (s, 3H), 2.38 – 2.28 (m, 1H), 2.19 – 2.08 (m, 1H), 1.22 (s, 3H), 0.99 (s, 3H); ^{13}C NMR (101 MHz, $CDCl_3$) δ 171.23, 170.53, 159.80 (d, *J* = 248 Hz), 156.52, 151.81, 150.99, 144.48, 143.93, 143.57, 141.70 (d, *J* = 7.32 Hz), 141.43, 141.38, 132.14 (d, *J* = 2.1 Hz), 129.97, 127.96, 127.26, 127.05, 125.23, 125.09, 124.59, 123.12 (d, *J* = 1.96 Hz), 120.17, 118.19 (d, *J* = 15.3 Hz), 115.05 (d, *J* = 22.7 Hz), 70.22, 68.70, 67.71, 59.07, 58.29, 56.81, 54.44, 47.09, 42.51, 36.90, 26.04, 25.45, 16.13 (d, *J* = 2.35 Hz). LC-MS, ESI$^+$, m/z 931 [M+H]$^+$.

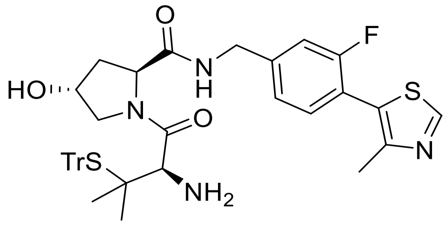

(2*S*,4*R*)-1-((*R*)-2-amino-3-methyl-3-(tritylthio)butanoyl)-N-(3-fluoro-4-(4-methylthiazol-5-yl)benzyl)-4-hydroxypyrrolidine-2-carboxamide (**1-6a**)

86 mg **1-6a** was prepared from 150 mg **1-5a** following general procedure 5 (75% yield). ^{1}H NMR (400 MHz, MeOD-d_4) δ 8.96 (s, 1H), 7.62 – 7.53 (m, 6H), 7.35 – 7.23 (m, 7H), 7.22 – 7.15 (m, 5H), 4.45 (t, *J* = 8.2 Hz, 1H), 4.40 – 4.22 (m, 3H), 3.24 (dd, *J* = 11.2, 4.1 Hz, 1H), 3.06 (d, *J* = 11.2 Hz, 1H), 2.72 (s, 1H), 2.34 (s, 3H), 2.16 – 2.07 (m, 1H), 2.00 – 1.90 (m, 1H), 1.25 (s, 3H), 1.17 (s, 3H); ^{19}F NMR (471 MHz, MeOD-d_4) δ -114.52. ^{13}C NMR (126 MHz, $CDCl_3$) δ 172.43, 171.75, 159.82 (d, *J* = 249.9 Hz), 151.81, 151.01, 144.87, 141.79 (d, *J* = 7.2 Hz), 132.15 (d, *J* = 2.1 Hz), 130.03, 127.98, 126.95, 124.53, 122.95 (d, *J* = 2.9 Hz), 118.17 (d, *J* = 15.5 Hz), 114.86 (d, *J* = 23.1 Hz), 70.06, 68.29, 59.17, 58.32, 58.07, 56.94, 42.42, 37.30, 25.25, 24.74, 16.16 (d, *J* = 2.1 Hz); LC-MS, ESI$^+$, m/z 731.6 [M+Na]$^+$.

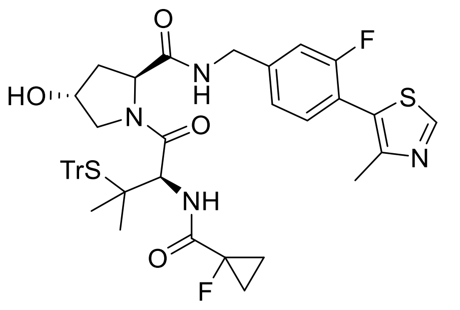

(2*S*,4*R*)-N-(3-fluoro-4-(4-methylthiazol-5-yl)benzyl)-1-((*R*)-2-(1-fluorocyclopropane-1-carboxamido)-3-methyl-3-(tritylthio)butanoyl)-4-hydroxypyrrolidine-2-carboxamide (**1-7a**)

77 mg **1-7a** was prepared from 85 mg **1-6a** following general procedure 6 (81% yield). ^{1}H NMR (500 MHz, $CDCl_3$) δ 8.79 (s, 1H), 7.65 – 7.52 (m, 6H), 7.46 (t, *J* = 6.1 Hz, 1H), 7.34 – 7.18 (m, 11H), 7.03 (d, *J* = 9.2 Hz, 2H), 4.65 (t, *J* = 8.1 Hz, 1H), 4.38 (s, 1H), 4.26 (d, *J* = 6.1 Hz, 2H), 3.66 (d, *J* = 5.2 Hz, 1H), 3.50 (d, *J* = 11.4 Hz, 1H), 3.41 (d, *J* = 5.6 Hz, 1H), 3.31 (dd, *J* = 11.4, 3.8 Hz, 1H), 2.41 (d, *J* = 1.0 Hz, 3H), 2.27 – 2.20 (m, 1H), 2.16 – 2.10 (m, 1H), 1.41 – 1.20 (m, 7H), 1.10 (s, 3H); ^{13}C NMR (126 MHz, $CDCl_3$) δ 171.17, 170.22 (d, *J* = 20.8 Hz), 170.14, 169.88, 159.78 (d, *J* = 250.0 Hz), 151.77, 150.98, 144.49, 141.68 (d, *J* = 7.7 Hz), 132.15, 129.88, 127.96, 127.01, 124.56, 123.16 (d, *J* = 3.1 Hz), 118.20 (d, *J* = 15.5 Hz), 115.08 (d, *J* = 23.1 Hz), 78.24 (d, *J* = 232.5Hz), 70.23, 68.53, 59.01, 57.08, 56.92, 54.09, 42.54, 36.90, 26.03, 25.64, 16.10 (d, *J* = 2.48 Hz), 13.72 (d, *J* = 10.0 Hz), 13.69 (d, *J* = 10.0 Hz). ^{19}F NMR (471 MHz, $CDCl_3$) δ -112.01, -197.22. LC-MS, ESI$^-$, m/z 793 [M-H]$^-$.

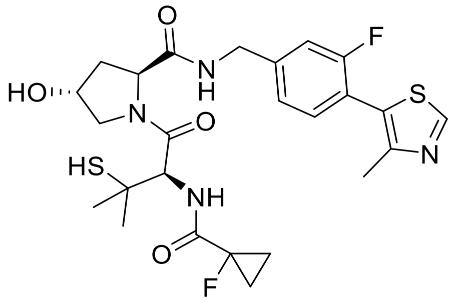

(2*S*,4*R*)-N-(3-fluoro-4-(4-methylthiazol-5-yl)benzyl)-1-((*R*)-2-(1-fluorocyclopropane-1-carboxamido)-3-mercapto-3-methylbutanoyl)-4-hydroxypyrrolidine-2-carboxamide (**1-8a**)

27 mg **1-8a** was prepared from 40 mg **1-7a** following general procedure 7 (97%yield). ^{1}H NMR (500 MHz, $CDCl_3$) δ 9.04 (s, 1H), 7.53 (t, *J* = 5.7 Hz, 1H), 7.43 (dd, *J* = 8.5, 3.0 Hz, 1H), 7.29 – 7.24 (m, 1H), 7.20 – 7.13 (m, 2H), 5.67 (br, s, 1H), 4.76 – 4.66 (m, 2H), 4.60 (dd, *J* = 15.5, 6.8 Hz, 1H), 4.56 – 4.52 (m, 1H), 4.32 (dd, *J* = 15.5, 5.3 Hz, 1H), 4.08 (d, *J* = 11.1 Hz, 1H), 3.81 (dd, *J* = 11.1, 3.7 Hz, 1H), 2.62 (s, 1H), 2.42 (s, 3H), 2.35 (ddd, *J* = 13.0, 8.4, 4.4 Hz, 1H), 2.24 – 2.16 (m, 1H), 1.43 (s, 3H), 1.38 (s, 3H), 1.36 – 1.25 (m, 4H). ^{13}C NMR (126 MHz, $CDCl_3$) δ 171.32, 170.6 (d, *J* = 20.7 Hz), 170.22, 159.8 (d, *J* = 251.0 Hz), 152.76, 149.28, 142.32 (d, *J* = 7.7 Hz), 132.14 (d, *J* = 2.3 Hz), 125.65, 123.55 (d, *J* = 4.4 Hz), 117.44 (d, *J* = 15.2 Hz), 115.38 (d, *J* = 22.9 Hz), 77.27 (d, *J* = 232.7 Hz), 70.26, 59.37, 57.81, 56.88, 46.55, 42.90, 37.12, 30.47, 28.83, 15.40, 14.05 (d, *J* = 10.5 Hz), 13.99 (d, *J* = 9.7 Hz).^{19}F NMR (471 MHz, $CDCl_3$) δ -111.74, -197.61. LC-MS, ESI$^+$, m/z 553.4 [M+H]$^+$.

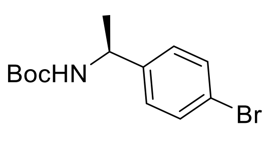

tert-butyl (*S*)-(1-(4-bromophenyl)ethyl)carbamate (**1-2b**)

3.6 g **1-2b** was obtained from 4.17 g **1-1b** following general procedure 1 (58% yield). ^{1}H NMR (400 MHz, $CDCl_3$) δ 7.51 – 7.44 (m, 2H), 7.20 (d, *J* = 8.4 Hz, 2H), 4.76 (s, 2H), 1.48 – 1.38 (m, 12H).

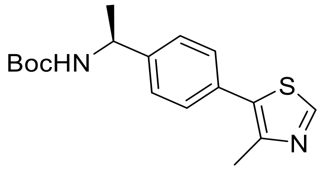

tert-butyl (*S*)-(1-(4-(4-methylthiazol-5-yl)phenyl)ethyl)carbamate (**1-3b**)

600 mg **1-3b** was prepared from 1.03 g **1-2b** following general procedure 2 (55% yield). ^{1}H NMR (400 MHz, $CDCl_3$) δ 8.69 (s, 1H), 7.47 – 7.41 (m, 2H), 7.38 (d, *J* = 8.3 Hz, 2H), 4.83 (s, 2H), 2.56 (s, 3H), 1.50 (d, *J* = 6.6 Hz, 3H), 1.46 (s, 9H).

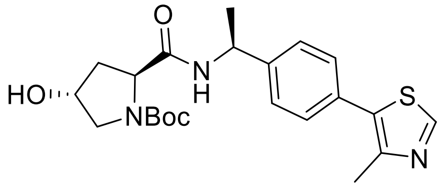

tert-butyl (2*S*,4*R*)-4-hydroxy-2-(((*S*)-1-(4-(4-methylthiazol-5-yl)phenyl)ethyl)carbamoyl)pyrrolidine-1-carboxylate (**1-4b**)

380 mg **1-4b** was prepared from 337 mg **1-3b** following general procedure 3 (83% yield). ^{1}H NMR (400 MHz, $CDCl_3$) δ 8.69 (s, 1H), 7.45 – 7.37 (m, 4H), 5.13 (s, 1H), 4.53 (s, 1H), 4.48 – 4.36 (m, 1H), 3.75 – 3.35 (m, 2H), 2.55 (s, 3H), 1.94 (d, *J* = 3.8 Hz, 1H), 1.62 (s, 1H), 1.56 – 1.33 (m, 12H).

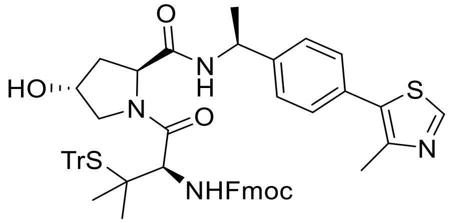

(9H-fluoren-9-yl)methyl ((*R*)-1-((2*S*,4*R*)-4-hydroxy-2-(((*S*)-1-(4-(4-methylthiazol-5-yl)phenyl)ethyl)carbamoyl)pyrrolidin-1-yl)-3-methyl-1-oxo-3-(tritylthio)butan-2-yl)carbamate (**1-5b**)

190 mg **1-5b** was prepared from 118 mg **1-4b** following general procedure 4 (75% yield). ^{1}H NMR (400 MHz, $CDCl_3$) δ 8.67 (s, 1H), 7.78 – 7.72 (m, 2H), 7.62 – 7.52 (m, 8H), 7.41 – 7.17 (m, 18H), 5.73 (d, *J* = 5.3 Hz, 1H), 5.00 (p, *J* = 7.0 Hz, 1H), 4.65 (t, *J* = 8.0 Hz, 1H), 4.42 – 4.15 (m, 4H), 3.56 (d, *J* = 5.3 Hz, 1H), 3.45 (d, *J* = 11.6 Hz, 1H), 3.17 (dd, *J* = 11.6, 3.5 Hz, 1H), 2.83 (br, s, 1H), 2.51 (s, 3H), 2.44 – 2.32 (m, 1H), 2.08 – 1.96 (m, 1H), 1.39 – 1.31 (m, 6H), 1.17 (s, 3H). ^{13}C NMR (101 MHz, $CDCl_3$) δ 170.91, 169.57, 156.61, 150.35, 148.68, 144.60, 144.02, 143.58, 143.26, 141.41, 130.99, 130.04, 129.68, 128.03, 127.94, 127.28, 127.08, 126.58, 125.29, 125.13, 120.18, 70.25, 67.79, 58.61, 58.38, 56.62, 48.92, 47.14, 35.86, 26.12, 26.05, 22.34, 16.23. LC-MS, ESI$^+$, *m/z* 927 $[M+H]^+$.

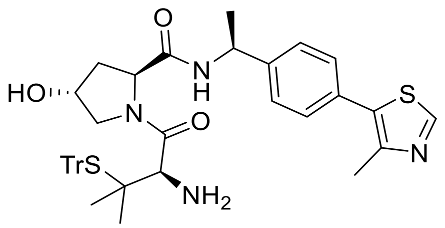

(2*S*,4*R*)-1-((*R*)-2-amino-3-methyl-3-(tritylthio)butanoyl)-4-hydroxy-N-((*S*)-1-(4-(4-methylthiazol-5-yl)phenyl)ethyl)pyrrolidine-2-carboxamide (**1-6b**)

80 mg **1-6b** was prepared from 150 mg **1-5b** following general procedure 5 (70% yield). ^{1}H NMR (400 MHz, MeOD-d_4) δ 8.82 (s, 1H), 7.67 – 7.55 (m, 6H), 7.41 – 7.31 (m, 4H), 7.30 – 7.23 (m, 6H), 7.23 – 7.14 (m, 3H), 4.92 (q, *J* = 7.0 Hz, 1H), 4.44 (t, *J* = 8.2 Hz, 1H), 4.33 – 4.23 (m, 1H), 3.13 (dd, *J* = 11.3, 4.1 Hz, 1H), 2.98 (d, *J* = 11.3 Hz, 1H), 2.64 (s, 1H), 2.43 (s, 3H), 2.13 – 2.04 (m, 1H), 1.86 – 1.78 (m, 1H), 1.40 (d, *J* = 7.0 Hz, 3H), 1.29 (s, 3H), 1.24 (s, 3H). ^{13}C NMR (101 MHz, MeOD-d_4) δ 173.07, 172.65, 152.75, 149.07, 146.36, 145.48, 133.28, 131.47, 131.12, 130.43, 128.89, 127.84, 127.57, 70.55, 69.16, 60.62, 58.98, 58.61, 58.03, 38.68, 26.29, 25.39, 22.26, 15.83. LC-MS, ESI^{+}, m/z 727.2 [M+Na^{+}].

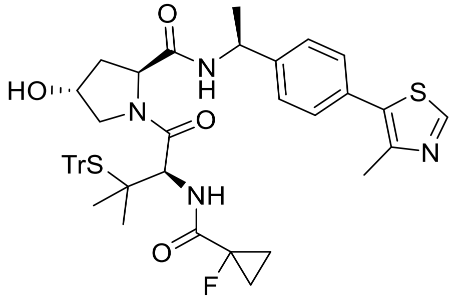

(2*S*,4*R*)-1-((*R*)-2-(1-fluorocyclopropane-1-carboxamido)-3-methyl-3-(tritylthio)butanoyl)-4-hydroxy-N-((S)-1-(4-(4-methylthiazol-5-yl)phenyl)ethyl)pyrrolidine-2-carboxamide (**1-7b**)

80 mg **1-7b** was prepared from 80 mg **1-6b** following general procedure 6 (89% yield). ^{1}H NMR (500 MHz, $CDCl_3$) δ 8.69 (s, 1H), 7.67 – 7.60 (m, 6H), 7.44 – 7.33 (m, 6H), 7.31 – 7.27 (m, 6H), 7.26 – 7.21 (m, 3H), 5.03 (p, *J* = 7.0 Hz, 1H), 4.65 (t, *J* = 8.1 Hz, 1H), 4.35 (s, 1H), 3.59 (d, *J* = 4.8 Hz, 1H), 3.49 (d, *J* = 11.5 Hz, 1H), 3.26 (d, *J* = 5.9 Hz, 1H), 3.19 (dd, *J* = 11.5, 3.7 Hz, 1H), 2.53 (s, 3H), 2.33 (ddd, *J* = 13.0, 8.1, 4.5 Hz, 1H), 2.11 – 1.97 (m, 1H), 1.41 – 1.32 (m, 8H), 1.29 – 1.20 (m, 5H). ^{19}F NMR (471 MHz, $CDCl_3$) δ -197.26. ^{13}C NMR (126 MHz, $CDCl_3$) δ 170.38 (*J* = 16.7 Hz), 170.11, 169.64, 150.34, 148.65, 144.60, 143.30, 131.77, 130.95, 129.94, 129.65, 128.02, 127.04, 126.60, 79.22, 70.25, 68.52, 58.64, 57.30, 56.78, 53.67, 48.90, 36.04, 26.16, 22.39, 16.21, 13.88 (d, *J* = 10.3 Hz), 13.71 (d, *J* = 10.3 Hz). LC-MS, ESI^{-}, m/z 789 [M-H]$^{-}$.

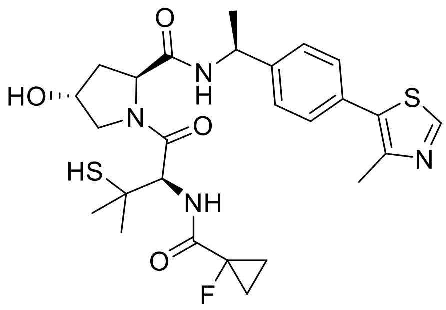

(2*S*,4*R*)-1-((*R*)-2-(1-fluorocyclopropane-1-carboxamido)-3-mercapto-3-methylbutanoyl)-4-hydroxy-N-((*S*)-1-(4-(4-methylthiazol-5-yl)phenyl)ethyl)pyrrolidine-2-carboxamide (**1-8b**)

25 mg **1-8b** was prepared from 40 mg **1-7b** following general procedure 7 (90% yield). ^{1}H NMR (500 MHz, $CDCl_3$) δ 8.73 (s, 1H), 7.49 – 7.43 (m, 1H), 7.42 – 7.36 (m, 4H), 7.28 (d, *J* = 8.4 Hz, 1H), 5.10 (p, *J* = 7.0 Hz, 1H), 4.72 – 4.66 (m, 2H), 4.55 – 4.49 (m, 1H), 4.25 (s, 1H), 4.09 (d, *J* = 11.2 Hz, 1H), 3.76 (dd, *J* = 11.2, 3.9 Hz, 1H), 2.71 (s, 1H), 2.53 (s, 3H), 2.44 – 2.37 (m, 1H), 2.13 – 2.05 (m, 1H), 1.54 – 1.50 (m, 6H), 1.43 (s, 3H), 1.38 – 1.27 (m, 4H). ^{13}C NMR (126 MHz, $CDCl_3$) δ 170.72 (d, *J* = 21.0 Hz), 170.43, 169.73, 150.70, 148.27, 143.35, 132.01, 130.85, 129.75, 126.66, 77.24 (d, *J* = 232.2 Hz), 70.17, 59.14, 57.85, 56.70, 49.10, 46.45, 36.51, 30.78, 28.82, 22.34, 15.97, 14.06 (d, *J* = 10.4 Hz), 14.05 (d, *J* = 10.9 Hz). ^{19}F NMR (471 MHz, $CDCl_3$) δ -197.71. LC-MS, ESI$^+$, m/z 549.5 [M+H]$^+$.

Scheme S2. Synthesis of cis- and trans-1,4-bis(bromomethyl)cyclohexane

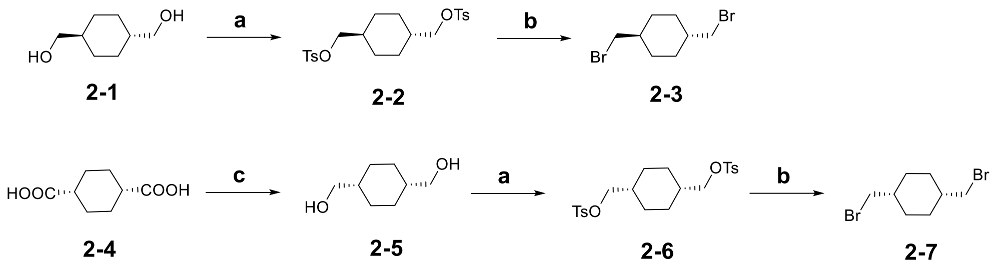

Reagents and conditons: a. TsCl, DMAP, TEA, DCM; b. LiBr, acetone, reflux; c. Me_2S-BH_3, THF, 0 °C

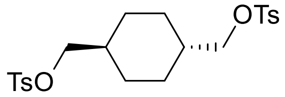

(*Trans*-cyclohexane-1,4-diyl)bis(methylene) bis(4-methylbenzenesulfonate) (**2-2**)

To a solution of *trans*-1,4-cyclohexanedimethanol (500 mg, 3.47 mmol) in DCM (10 ml) was added TEA (1.93 ml, 13.9 mmol), DMAP (42 mg, 0.34 mmol) and TsCl (1.655g, 8.68 mmol) separately at 0 °C. The resulting mixture was then warmed to room temperature and stirred at room temperature overnight. DCM was added, and the mixture was washed with water and brine, dried over sodium sulfate, filtered, condensed under reduced pressure to afford a residue which was purified through flash column chromatography on silica gel (0%-10% Methanol in DCM) to give the title compound (1.29 g, 82%yield) as white solid. ^{1}H NMR (400 MHz, $CDCl_3$) δ 7.76 (d, *J* = 8.3 Hz, 4H), 7.33 (d, *J* = 8.0 Hz, 4H), 3.80 (d, *J* = 6.3 Hz, 4H), 2.45 (s, 6H), 1.83 – 1.63 (m, 4H), 1.65 – 1.50 (m, 2H), 0.99 – 0.78 (m, 4H). ^{13}C NMR (101 MHz, $CDCl_3$) δ 144.84, 133.31, 129.96, 128.01, 74.94, 37.15, 28.22, 21.76. LC-MS, ESI$^+$, m/z 475 [M+Na]$^+$.

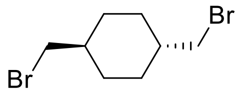

Trans-1,4-bis(bromomethyl)cyclohexane (**2-3**)

To a solution of **2-2** (125 mg, 0.28 mmol) in acetone (3 ml) was added LiBr (95 mg, 1.1 mmol). After stirring at reflux overnight, the mixture was cooled down, added with ethyl acetate, washed with water and brine, dried over sodium sulfate, filtered, and condensed under reduced pressure to give a residue which was purified with flash column chromatography on silica gel (0% to 20% ethyl acetate in heptane) to give the title compound (61 mg, 81% yield) as white solid. ^{1}H NMR (400 MHz, $CDCl_3$) δ 3.28 (d, *J* = 6.3 Hz, 4H), 2.03 – 1.87 (m, 4H), 1.67 – 1.55 (m, 2H), 1.19 – 0.91 (m, 4H). ^{13}C NMR (101 MHz, $CDCl_3$) δ 40.03, 39.91, 31.17.[2]

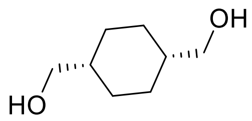

cis-1,4-cyclohexanedimethanol (**2-5**)

To a solution of *cis*-cyclohexane-1,4-dicarboxylic acid (500 mg, 2.9 mmol) in THF (5 ml) was added Me_2S-BH_3 (2 M in THF, 5.8 ml, 11.6 mmol) at 0 °C. After stirring at 0 °C for 2h, the reaction mixture was quenched with methanol, then condensed under reduced pressure to afford a residue which was purified with flash column chromatography on silica gel to give the title compound (418 mg, 100% yield) as a colorless oil. ^{1}H NMR (400 MHz, $CDCl_3$) δ 3.54 (d, *J* = 6.9 Hz, 2H), 1.75 – 1.63 (m, 2H), 1.60 – 1.50 (m, 2H), 1.46 – 1.37 (m, 2H). ^{13}C NMR (101 MHz, $CDCl_3$) δ 66.23, 38.25, 25.44.

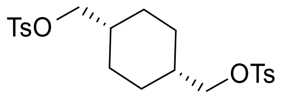

(*Cis*-cyclohexane-1,4-diyl)bis(methylene) bis(4-methylbenzenesulfonate) (**2-6**)

TsCl (437 mg, 2.3 mmol) was added to a mixture of *cis*-1,4-cyclohexanedimethanol (150 mg, 1.04 mmol), TEA (0.58 ml, 4.17 mmol), and DMAP (13 mg, 0.1 mmol) in DCM (20 ml) at 0 °C. The mixture was then warmed to room temperature and stirred for overnight. DCM was added, and the reaction mixture was washed with water and brine, dried over sodium sulfate, filtered, condensed to afford a residue which was purified through flash column chromatography on silica gel (0%-30% ethyl acetate in heptane) to give the title compound (208 mg, 44% yield) as white solid. ^{1}H NMR (400 MHz, $CDCl_3$) δ 7.83 – 7.68 (m, 4H), 7.34 (d, *J* = 8.0 Hz, 4H), 3.85 (d, *J* = 7.1 Hz, 4H), 2.44 (s, 6H), 1.90 – 1.76 (m, 2H), 1.50 – 1.40 (m, 4H), 1.30 – 1.20 (m, 4H). ^{13}C NMR (101 MHz, $CDCl_3$) δ 144.89, 133.19, 129.97, 127.91, 72.84, 34.52, 24.64, 21.71. LC-MS, ESI$^+$, m/z 475 $[M+Na]^+$.

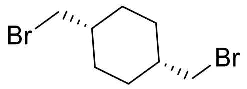

Cis-1,4-bis(bromomethyl)cyclohexane (**2-7**)

To a solution of **2-6** (150 mg, 0.33 mmol) in acetone (5 ml) was added LiBr (358 mg, 1.32 mmol). After refluxing overnight, the reaction mixture was cooled down to room temperature, diluted with ethyl acetate, washed with water and brine separately, dried over sodium sulfate, filtered, condensed to afford a residue which was purified with flash column chromatography on silica gel (0%-30% ethyl acetate in heptane) to give the title compound (75 mg, 83% yield) as white solid. ^{1}H NMR (400 MHz, $CDCl_3$) δ 3.37 (d, *J* = 7.0 Hz, 4H), 1.92 – 1.80 (m, 2H), 1.71 – 1.58 (m, 4H), 1.57 – 1.45 (m, 4H). ^{13}C NMR (101 MHz, $CDCl_3$) δ 38.20, 37.76, 27.17.

Scheme S3. Synthesis of SD74, SD75, SD82, SD100, XL01078B, XL01072, XL01078B, XL01119, XL01118, XL01120, XL0123, XL01122, XL01121, XL01131, XL01111, XL01134

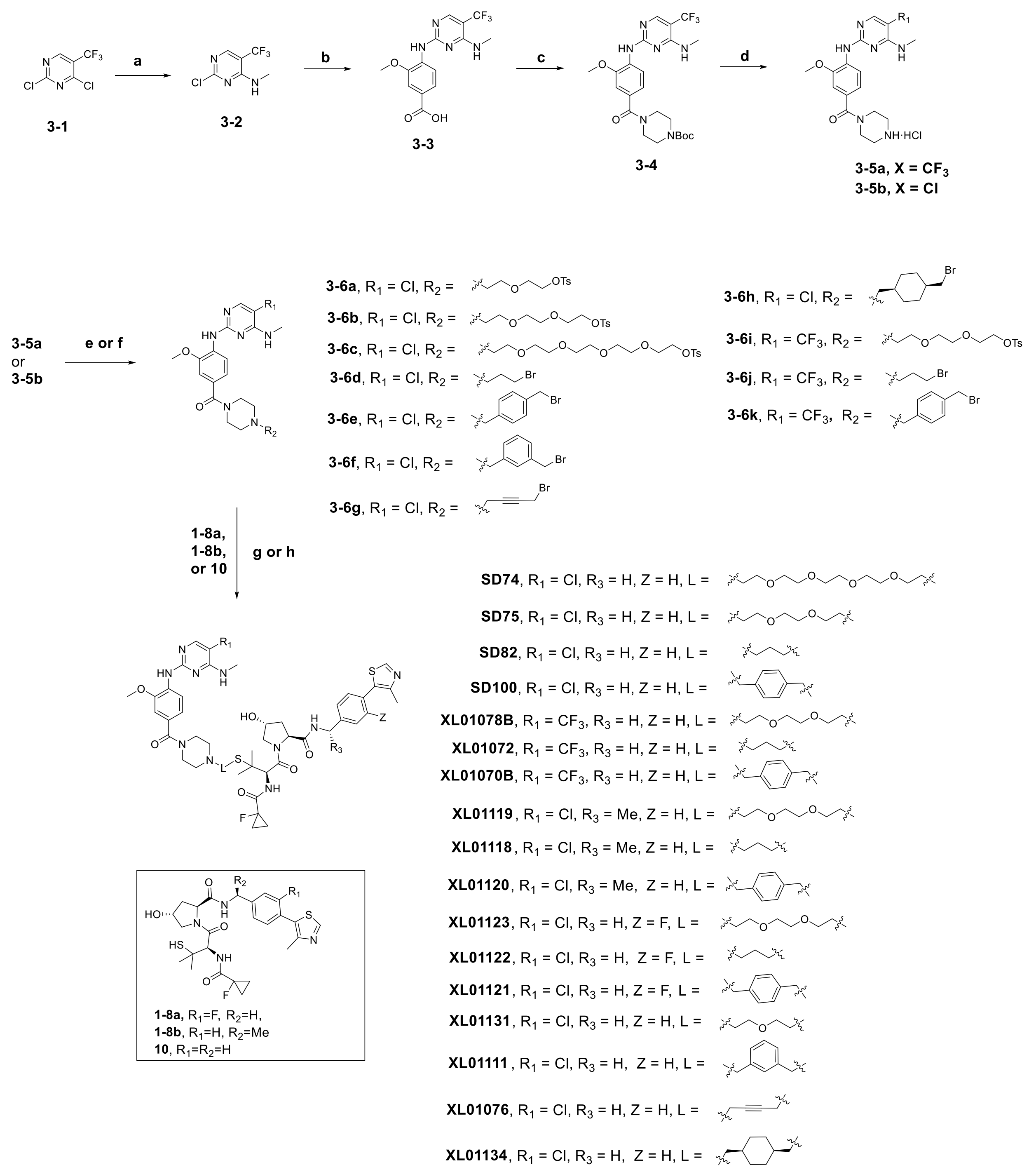

Reagents and conditions: a. $MeNH_2$, DIPEA, THF, 0 °C; b. 4 N HCl in 1,4-Dioxane, water, 4-amino-3-methoxybenzoic acid, 100 °C; c. HOBt, EDCI, DIPEA, 1-Boc-piperizine, DMF; d, 4N HCl in 1,4-dioxane, DCM, methanol; e. K_2CO_3, dibromide or ditosylate linker, acetone, rt or 50 °C; f. 4-(bromomethyl)benzaldehyde or 3-(bromomethyl)benzaldehyde or 2-(2-oxoethoxy)ethyl 4-methylbenzenesulfonate, $Na(OAc)_3BH$, DCM; g. **1-8a**, **1-8b** or **10**, DBU, THF; h. K_2CO_3, KI, DMF, 100 °C.

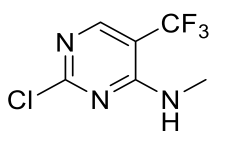

2-chloro-N-methyl-5-(trifluoromethyl)pyrimidin-4-amine (**3-2**)

To a stirred solution of 2,4-dichloro-5-(trifluoromethyl)pyrimidine (2.6 g, 11.99 mmol) in anhydrous THF (50 mL) was added DIPEA (2.3 mL, 13.18 mmol) followed by methylamine (33% solution in ethanol, 1.64 mL, 13.18 mmol) dropwise at 0°C. The reaction mixture was stirred at 0°C for 1 hour, then warmed to room temperature and concentrated in vacuo. The crude residue was purified by flash column chromatography on silica gradient from 0% to 50% of EtOAc in Petroleum spirit to give the title compound (1.36 g, 6.42 mmol, 53% yield). ^{1}H NMR (500 MHz, $CDCl_3$) δ 8.27 (d, *J*= 0.90 Hz, 1H), 5.51 (br s, 1H), 3.14 (d, *J* = 4.80 Hz, 3H); ^{19}F NMR (471 MHz, $CDCl_3$) δ - 63.30; ^{13}C NMR (126 MHz, $CDCl_3$) δ 163.82, 159.75,154.91, 123.75 (q, *J* = 272 Hz), 106.05, 28.41. LC-MS, ESI$^+$, m/z 212.02 [M+H]$^+$.

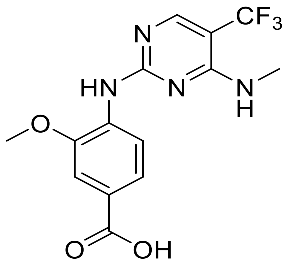

3-methoxy-4-((4-(methylamino)-5-(trifluoromethyl)pyrimidin-2-yl)amino)benzoic acid (**3-3**)

To a solution of 2-chloro-N-methyl-5-(trifluoromethyl)pyrimidin-4-amine (1.0g, 4.72 mmol) in a mixture of dioxane and water (20 ml : 20 ml) was added 4-amino-3-methoxybenzoic acid (0.87g, 5.20 mmol) followed by 4N solution of HCl in dioxane (1.3 ml , 5.20 mmol). After refluxing at 100 °C for 2h, the mixture was cooled to room temperature to precipitate white solids. The solids were filtered, washed with water, dried under vacuum to afford the title compound as white solid (1.25 g, 77% yield). ^{1}H NMR (500 MHz, DMSO-d_6) δ 12.71 (br, 1H), 9.19 (s, 1H), 8.44 (s, 1H), 8.36 (d, *J* = 8.4 Hz, 1H), 8.07 (br, 1H), 7.63 (dd, *J* = 8.4, 1.7 Hz, 1H), 7.57 (d, *J* = 1.7 Hz, 1H), 3.95 (s, 3H), 2.99 (d, *J* = 4.4 Hz, 3H). ^{19}F NMR (471 MHz, DMSO-d_6) δ -61.32. ^{13}C NMR (126 MHz, DMSO-d_6) δ 167.31, 158.41, 156.47, 149.50, 149.21, 131.41, 126.51, 124.00 (q, *J* = 270.37 Hz), 122.85, 120.39, 111.79, 99.33, 56.55, 29.19. LC-MS, ESI$^+$, m/z 343 [M+H]$^+$.

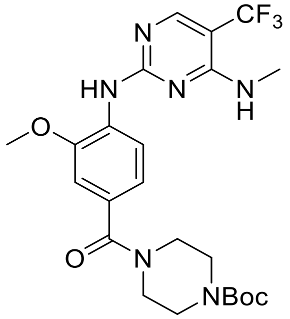

tert-butyl4-(3-methoxy-4-((4-(methylamino)-5-(trifluoromethyl)pyrimidin-2-yl)amino)benzoyl)piperazine-1-carboxylate (**3-4**)

To a solution of **3-3** (0.98 g, 2.58 mmol) in DMF (15 mL) was added HOBt (0.42 g, 3.09 mmol), EDCl (0.59 g, 3.09 mmol), 1-Boc-piperazine (0.51g, 2.73 mmol), and DIPEA (1.8 mL, 10.32 mmol) at room temperature. After stirring at room temperature for 16 h, the mixture was diluted with water (30 mL) and extracted with EtOAc (100 mL). The organic layer was washed with brine (15 mL) and concentrated to give a residue which was purified by flash column chromatography on silica (0% to 100% of EtOAc in DCM) to give the title compound (1.12 g, 2.19 mmol, 85% yield). ^{1}H NMR (500 MHz, $CDCl_3$) δ 8.60 (d, *J*=8.2 Hz, 1H), 8.21 (s, 1H), 7.85 (s, 1H), 7.04 (m, 2H), 5.26 (m, 1H), 3.97 (s, 3H), 3.65 (br s, 4H), 3.49 (br s, 4H), 3.14 (d, *J* = 4.7 Hz, 3H), 1.50 (s, 9H). ^{19}F NMR (471 MHz, $CDCl_3$) δ – 61.52. ^{13}C NMR (101 MHz, $CDCl_3$) δ 170.73, 162.62, 160.80, 159.70, 154.67 (q, *J* = 5.0 Hz), 147.93, 130.93, 128.54, 125.05 (q, *J* = 267 Hz), 120.13, 117.89, 109.73, 99.56, 80.43, 56.03, 43.93, 28.50, 28.45; LC-MS, ESI$^-$, m/z 509.2 [M-H]$^-$.

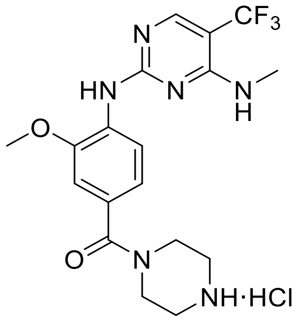

(3-methoxy-4-((4-(methylamino)-5-(trifluoromethyl)pyrimidin-2-yl)amino)phenyl)(piperazin-1-yl)methanone HCl salt (**3-5a**)

To a solution of **3-4** (1.12 g, 2.19 mmol) in a mixture of DCM and MeOH 9:1 (20 ml) was added 4N solution of HCl in dioxane (2.2 ml, 8.76 mmol) at room temperature. After stirring at room temperature overnight, the reaction mixture was diluted with Et_2O (100 ml) to precipitate white solid which was filtered, washed with Et_2O (50 ml) and air dried overnight to give the title compound (0.97 g, 2.17 mmol, 99%) as a white powder. ^{1}H NMR (500 MHz, DMSO-d_6) δ 9.81 (s, 1H), 9.74 (s, 2H), 8.51 (s, 1H), 8.44 (s, 1H), 8.14 (d, *J* = 6.0 Hz, 1H), 7.21 (d, *J* = 1.6 Hz, 1H), 7.11 (dd, *J* =1.6 Hz, 8.20 Hz,1H), 3.91 (s, 3H), 3.76 (br s, 4H), 3.14 (br s, 4H), 2.99 (d, *J* = 4.4 Hz, 3H). ^{19}F NMR (471 MHz, DMSO-d_6) δ - 61.54. ^{13}C NMR (126 MHz, DMSO-d_6) δ 169.5, 158.2, 154.6, 150.4, 132.16, 127.53, 123.3 (q, *J* = 269 Hz), 119.97, 111.09, 99.4, 65.5, 56.7, 42.9, 29.7. LC-MS, ESI$^+$, m/z 411 [M+H]$^+$.

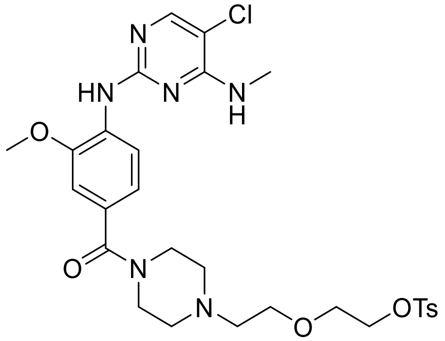

2-(2-(4-(4-((5-chloro-4-(methylamino)pyrimidin-2-yl)amino)-3-methoxybenzoyl)piperazin-1-yl)ethoxy)ethyl 4-methylbenzenesulfonate (**3-6a**)

To a solution of 2-(2-Hydroxyethoxy)ethyl 4-methylbenzenesulfonate (100 mg, 0.38 mmol) in DCM (3 ml) was added Dess-Martin Periodinane (195 mg, 0.46 mmol). After stirring at room temperature for 1h, the reaction mixture was diluted with DCM, washed with water and brine, dried over sodium sulfate, filtered, and condensed to afford a residue (2-(2-oxoethoxy)ethyl 4-methylbenzenesulfonate) which was used to next step without further purification. Half of the residue from the last step was dissolved in DCM (4 ml), followed by addition of **3-5b** (63 mg, 0.15 mmol), Sodium triacetoxyborohydride (72 mg, 0.34 mmol), and HOAc (1 drop). After stirring at room temperature for 2h, the mixture was diluted with DCM, washed with water and brine, dried over sodium sulfate, filtered, condensed to afford a residue which was purified with flash column chromatography on silica gel (0%-10% methanol in DCM) to give the title compound (46 mg, 49% yield). ^{1}H NMR (400 MHz, $CDCl_3$) δ 8.54 (d, *J* = 8.9 Hz, 1H), 7.92 (s, 1H), 7.81 – 7.75 (m, 2H), 7.68 (s, 1H), 7.32 (d, *J* = 8.0 Hz, 2H), 7.03 – 6.95 (m, 2H), 5.34 – 5.29 (m, 1H), 4.17 – 4.13 (m, 2H), 3.92 (s, 3H), 3.74 – 3.58 (m, 8H), 3.14 – 3.07 (m, 3H), 2.64 (t, *J* = 5.4 Hz, 2H), 2.60 – 2.51 (m, 4H), 2.43 (s, 3H). ^{13}C NMR (101 MHz, $CDCl_3$) δ 170.63, 158.74, 157.90, 152.68, 147.64, 145.03, 133.26, 131.59, 129.99, 128.07, 127.79, 120.34, 117.01, 109.76, 105.65, 69.23, 68.95, 68.73, 57.61, 56.04, 53.60, 28.24, 21.76. LC-MS, ESI$^+$, m/z 619.35 [M+H]$^+$.

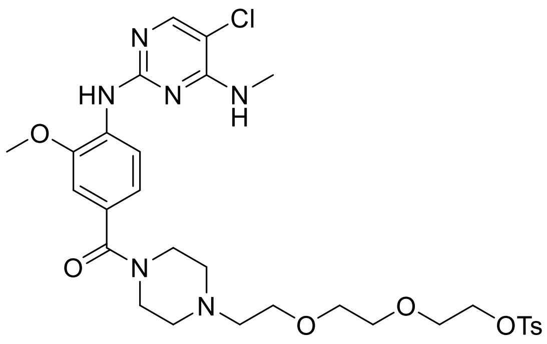

2-(2-(2-(4-(4-((5-chloro-4-(methylamino)pyrimidin-2-yl)amino)-3-methoxybenzoyl)piperazin-1-yl)ethoxy)ethoxy)ethyl 4-methylbenzenesulfonate (**3-6b**)

97 mg **3-6b** is prepared from 91 mg **3-5b** and tri(ethylene glycol) di-p-toluenesulfonate following general procedure 8 (66% yield). ^{1}H NMR (400 MHz, $CDCl_3$) δ 8.51 (d, *J* = 8.7 Hz, 1H), 7.87 (s, 1H), 7.78 – 7.71 (m, 2H), 7.59 (s, 1H), 7.29 (d, *J* = 8.0 Hz, 2H), 7.01 – 6.92 (m, 2H), 5.37 (q, *J* = 4.6 Hz, 1H), 4.14 – 4.09 (m, 2H), 3.88 (s, 3H), 3.74 – 3.48 (m, 12H), 3.05 (d, *J* = 4.9 Hz, 3H), 2.57 (t, *J* = 5.7 Hz, 2H), 2.48 (s, 4H), 2.39 (s, 3H). ^{13}C NMR (101 MHz, $CDCl_3$) δ 170.43, 158.57, 157.81, 152.62, 147.38, 144.82, 133.09, 131.34, 129.84, 127.94, 127.87, 120.19, 116.76, 109.57, 105.51, 70.81, 70.33, 69.24, 68.98, 68.72, 57.74, 55.88, 53.67, 28.08, 21.62. LC-MS, ESI$^+$, m/z 663.3 [M+H]$^+$.

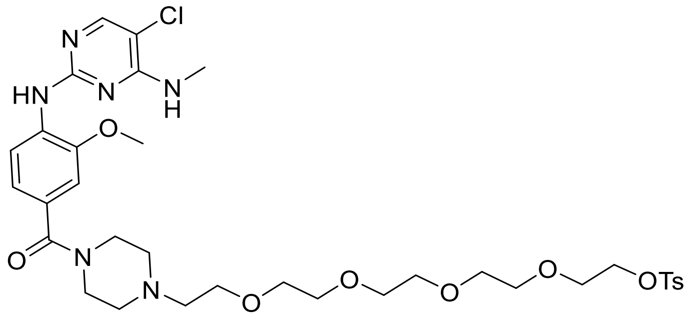

14-(4-(4-((5-chloro-4-(methylamino)pyrimidin-2-yl)amino)-3-methoxybenzoyl)piperazin-1-yl)-3,6,9,12-tetraoxatetradecyl 4-methylbenzenesulfonate (**3-6c**)

30 mg **3-6c** was prepared from 40 mg **3-5b** and 159 mg pentaethylene glycol di(p-toluenesulfonate) following general procedure 8 (41% yield). ^{1}H NMR (400 MHz, $CDCl_3$) δ 8.53 (d, *J* = 8.7 Hz, 1H), 7.90 (s, 1H), 7.78 (d, *J* = 8.2 Hz, 2H), 7.61 (s, 1H), 7.32 (d, *J* = 8.0 Hz, 2H), 7.02 – 6.96 (d, *J* = 7.0 Hz, 2H), 5.32 (q, *J* = 4.3 Hz, 1H), 4.19 – 4.11 (m, 2H), 3.91 (s, 3H), 3.77 – 3.50 (m, 20H), 3.09 (d, *J* = 4.8 Hz, 3H), 2.61 (t, *J* = 5.5 Hz, 2H), 2.52 (s, 4H), 2.43 (s, 3H); ^{13}C NMR (101 MHz, $CDCl_3$) δ 170.53, 158.67, 157.92, 152.75, 147.50, 144.86, 133.24, 131.43, 129.92, 128.07, 127.99, 120.26, 116.85, 109.68, 105.61, 70.87, 70.76, 70.71, 70.65, 70.52, 69.32, 69.03, 68.81, 57.84, 55.98, 53.77, 28.18, 21.72. LC-MS, ESI$^-$, m/z 749 [M-H]$^-$.

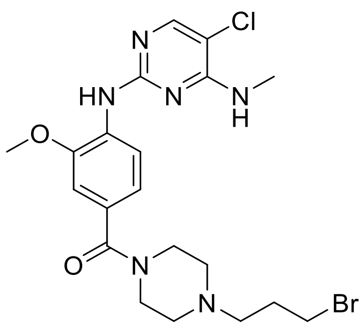

(4-(3-bromopropyl)piperazin-1-yl)(4-((5-chloro-4-(methylamino)pyrimidin-2-yl)amino)-3-methoxyphenyl)methanone (**3-6d**)

51 mg **3-6d** was prepared from 100 mg **3-5b** and 181 mg 1,3-dibromopropane following general procedure 8 (42% yield). ^{1}H NMR (400 MHz, $CDCl_3$) δ 8.59 – 8.47 (m, 1H), 7.91 (s, 1H), 7.62 (s, 1H), 7.03 – 6.97 (m, 2H), 5.36 – 5.26 (m, 1H), 3.91 (s, 3H), 3.77 – 3.53 (m, 4H), 3.47 (t, *J* = 6.6 Hz, 2H), 3.09 (d, *J* = 4.9 Hz, 3H), 2.56 – 2.36 (m, 6H), 2.02 (p, *J* = 6.7 Hz, 2H). ^{13}C NMR (101 MHz, $CDCl_3$) δ 170.56, 158.68, 157.93, 152.77, 147.52, 131.49, 127.94, 120.28, 116.87, 109.69, 105.64, 56.38, 55.98, 53.45, 31.64, 30.00, 28.18. LC-MS, ESI$^+$, m/z 497 [M+H]$^+$, 499 [M+H+2]$^+$.

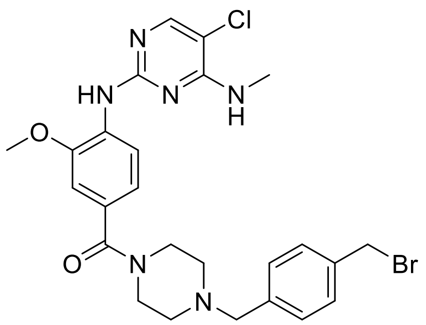

(4-(4-(bromomethyl)benzyl)piperazin-1-yl)(4-((5-chloro-4-(methylamino)pyrimidin-2-yl)amino)-3-methoxyphenyl)methanone (**3-6e**)

To a suspension of **3-5b** (80 mg, 0.19 mmol) and 4-(bromomethyl)benzaldehyde (38.5 mg, 0.19 mmol) in DCM (3 ml) was added $Na(OAc)_3BH$ (123 mg, 0.58 mmol). After stirring at room temperature overnight, the mixture was diluted with DCM and washed with water and brine, dried over sodium sulfate, filtered and condensed to give a residue which was purified through flash column chromatography on silica gel (0% -10% methanol in DCM) to afford the title compound (70 mg, 65% yield) as white solid. ^{1}H NMR (400 MHz, $CDCl_3$) δ 8.55 – 8.47 (m, 1H), 7.88 (s, 1H), 7.62 (s, 1H), 7.35 – 7.25 (m, 4H), 7.0 - 6.95 (m, 2H), 5.46 – 5.30 (m, 1H), 4.46 (s, 2H), 3.89 (s, 3H), 3.64 (s, 4H), 3.50 (s, 2H), 3.06 (d, *J* = 4.9 Hz, 3H), 2.44 (s, 4H). ^{13}C NMR (100 MHz, $CDCl_3$) δ 170.50, 158.61, 157.83, 152.63, 147.45, 138.17, 136.87, 131.40, 129.50, 129.10, 127.88, 120.21, 116.83, 109.63, 105.55, 62.51, 55.92, 53.19, 33.37, 28.12, 14.25. LC-MS, ESI$^+$, m/z 559 [M+H]$^+$, 561 [M+H+2]$^+$.

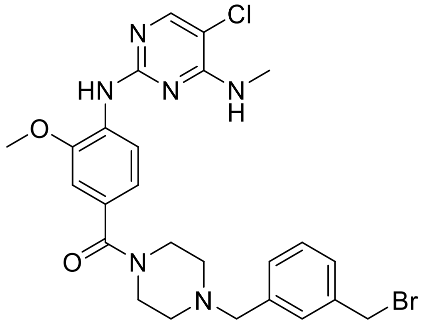

(4-(3-(bromomethyl)benzyl)piperazin-1-yl)(4-((5-chloro-4-(methylamino)pyrimidin-2-yl)amino)-3-methoxyphenyl)methanone (**3-6f**)

To a suspension of **3-5b** (80 mg, 0.19 mmol) and 3-bromomethyl benzylaldehyde (38.5 mg, 0.19 mmol) in DCM (4 mL) was added sodium triacetoxyborohydride (123 mg, 0.58 mmol). After stirring at room temperature overnight, the mixture was diluted with DCM, washed with water and brine, dried over sodium sulfate, filtered, and condensed under reduced pressure to afford a residue which was purified with flash column chromatography on silica gel (0%-10% methanol in DCM) to give the title compound (51 mg, 46% yield). ^{1}H NMR (400 MHz, $CDCl_3$) δ 8.46 (d, *J* = 8.8 Hz, 1H), 7.84 (s, 1H), 7.56 (s, 1H), 7.28 (s, 1H), 7.23 – 7.14 (m, 3H), 6.95 – 6.90 (m, 2H), 5.30 – 5.22 (m, 1H), 4.41 (s, 2H), 3.84 (s, 3H), 3.67 – 3.51 (m, 4H), 3.46 (s, 2H), 3.02 (d, *J* = 4.9 Hz, 3H), 2.39 (s, 4H). ^{13}C NMR (100 MHz, $CDCl_3$) δ 170.59, 158.68, 157.92, 152.74, 147.53, 138.53, 138.06, 131.47, 129.74, 129.27, 128.93, 128.11, 127.97, 120.28, 116.89, 109.70, 105.62, 62.71, 55.99, 53.26, 33.57, 28.19. LC-MS, ESI$^+$, m/z 559.2 [M+H]$^+$, 561.2 [M+H+2]$^+$.

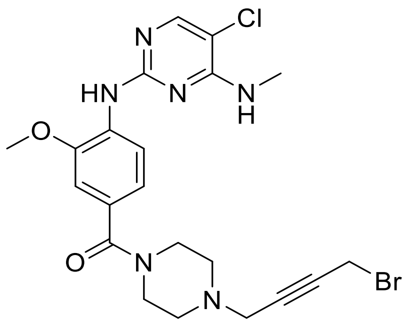

(4-(4-bromobut-2-yn-1-yl)piperazin-1-yl)(4-((5-chloro-4-(methylamino)pyrimidin-2-yl)amino)-3-methoxyphenyl)methanone (**3-6g**)

To a solution of **3-5b** (60 mg, 0.134 mmol) in acetone (2 ml) was added 1,4-dibromo-2-butyne (85.4 mg, 0.403 mmol) and K_2CO_3 (46.4 mg, 0.336 mmol). After stirring at room temperature for 3h, the resulting mixture was diluted with DCM, washed with water and brine separately, dried over sodium sulfate, filtered, and condensed to afford a residue which was purified on flash column chromatography on silica gel (0%-10% methanol in DCM) to give the title compound. The product was unstable and was used to next step as an impure mixture.

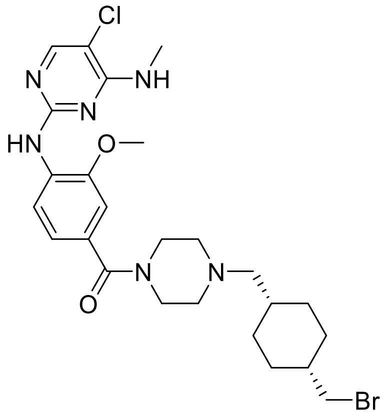

(4-(((1*S*,4*S*)-4-(bromomethyl)cyclohexyl)methyl)piperazin-1-yl)(4-((5-chloro-4-(methylamino)pyrimidin-2-yl)amino)-3-methoxyphenyl)methanone (**3-6h**)

10 mg **3-6h** was obtained from 25 mg **3-5b** and 50 mg *Cis-1,4-bis(bromomethyl)cyclohexane* (**2-7**) (50 mg, 0.18 mmol) following general procedure 8 (29% yield). ^{1}H NMR (400 MHz, $CDCl_3$) δ 8.54 (d, *J* = 8.8 Hz, 1H), 7.92 (s, 1H), 7.62 (s, 1H), 7.00 (dd, *J* = 6.7, 1.7 Hz, 2H), 5.34 – 5.26 (m, 1H), 3.92 (s, 3H), 3.64 (s, 4H), 3.37 (d, *J* = 7.0 Hz, 2H), 3.10 (d, *J* = 4.9 Hz, 3H), 2.41 (s, 4H), 2.24 (d, *J* = 7.5 Hz, 2H), 1.89 – 1.81 (m, 1H), 1.79 – 1.67 (m, 1H), 1.68 – 1.32 (m, 8H). ^{13}C NMR (101 MHz, $CDCl_3$) δ 170.53, 158.72, 157.98, 152.83, 147.55, 131.43, 128.18, 120.30, 116.91, 109.73, 105.66, 62.04, 56.01, 53.88, 38.99, 38.41, 31.92, 28.22, 27.43, 27.02. LC-MS, ESI$^+$, m/z 565, $[M+H]^+$, 567 $[M+H+2]^+$.

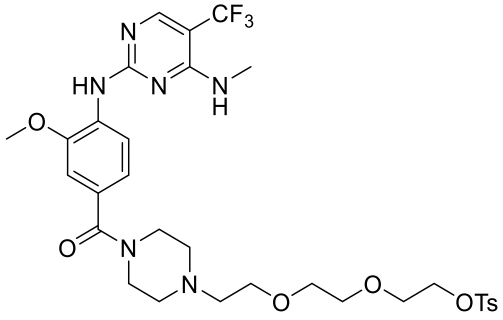

2-(2-(2-(4-(3-methoxy-4-((4-(methylamino)-5-(trifluoromethyl)pyrimidin-2-yl)amino)benzoyl)piperazin-1-yl)ethoxy)ethoxy)ethyl 4-methylbenzenesulfonate (**3-6i**)

36 mg **3-6i** was prepared from 50 mg **3-5a** and 205 mg triethylene glycol ditosylate following general procedure 8 (46% yield). ^{1}H NMR (400 MHz, $CDCl_3$) δ 8.61 – 8.51 (m, 1H), 8.16 (d, *J* = 0.7 Hz, 1H), 7.86 – 7.73 (m, 3H), 7.31 (d, *J* = 8.0 Hz, 2H), 7.03 -6.98 (m, 2H), 5.29 – 5.24 (m, 1H), 4.16 – 4.11 (m, 2H), 3.91 (s, 3H), 3.80 – 3.47 (m, 12H), 3.09 (d, *J* = 4.7 Hz, 3H), 2.59 (t, *J* = 5.7 Hz, 2H), 2.51 (s, 4H), 2.41 (s, 3H); ^{13}C NMR (101 MHz, $CDCl_3$) δ 170.32, 160.79, 159.67, 154.63 (q, *J* = 5.0 Hz), 147.84, 144.89, 133.19, 130.59, 129.91, 129.01, 128.03, 125.05 (d, *J* = 268 Hz), 120.11, 117.83, 109.73, 70.91, 70.42, 69.30, 69.08, 68.82, 57.82, 56.00, 53.74, 28.43, 21.69; LC-MS, ESI$^+$, m/z 697 $[M+H]^+$.

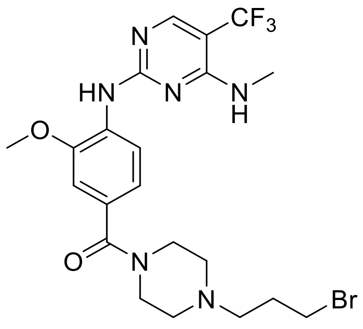

(4-(3-bromopropyl)piperazin-1-yl)(3-methoxy-4-((4-(methylamino)-5-(trifluoromethyl)pyrimidin-2-yl)amino)phenyl)methanone (**3-6j**)

51 mg **3-6j** was prepared from 100 mg **3-5a** and 181 mg 1,3-dibromoproprane following general procedure 8 (42% yield). ^{1}H NMR (400 MHz, $CDCl_3$) δ 8.63 – 8.49 (m, 1H), 8.16 (d, *J* = 0.7 Hz, 1H), 7.81 (s, 1H), 7.09 – 6.90 (m, 2H), 5.31 – 5.23 (m, 1H), 3.92 (s, 3H), 3.80 – 3.50 (m, 4H), 3.46 (t, *J* = 6.6 Hz, 2H), 3.09 (d, *J* = 4.7 Hz, 3H), 2.55 – 2.35 (m, 6H), 2.01 (p, *J* = 6.7 Hz, 2H); ^{13}C NMR (101 MHz, $CDCl_3$) δ 170.33, 160.80, 159.67, 154.65 (q, *J* = 4.9 Hz), 147.86, 130.64, 128.95, 125.05 (q, *J* = 268 Hz), 120.08, 117.84, 109.72, 99.61 (q, *J* = 31.65 Hz), 56.34, 56.00, 53.40, 31.60, 29.97, 28.42; LC-MS, ESI$^+$, m/z 531 $[M+H]^+$, 533 $[M+H+2]^+$.

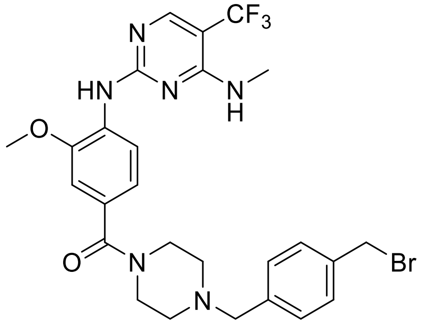

(4-(4-(bromomethyl)benzyl)piperazin-1-yl)(3-methoxy-4-((4-(methylamino)-5-(trifluoromethyl)pyrimidin-2-yl)amino)phenyl)methanone (**3-6k**)

$Na(OAc)_3BH$ (114 mg, 0.54 mmol) was added to a mixture of 4-bromomethylbenzylaldehyde (36 mg, 0.18 mmol) and **3-5a** (80 mg, 0.18 mmol) in DCM (5 ml). After stirring at room temperature for 3h, the resulting mixture was diluted with DCM, washed with water and brine separately, dried over sodium sulfate, filtered, and condensed under reduced pressure to afford a residue which was purified with flash column chromatography on silica gel (0%-10% Methanol in DCM) to give the title compound (70 mg, 66% yield). ^{1}H NMR (400 MHz, $CDCl_3$) δ 8.54 (d, *J* = 8.2 Hz, 1H), 8.17 (d, *J* = 0.8 Hz, 1H), 7.85 (s, 1H), 7.37 – 7.27 (m, 4H), 7.07 – 6.97 (m, 2H), 5.25 (d, *J* = 3.4 Hz, 1H), 4.49 (s, 2H), 3.92 (s, 3H), 3.66 (s, 4H), 3.54 (s, 2H), 3.10 (d, *J* = 4.7 Hz, 3H), 2.47 (s, 4H). ^{13}C NMR (100 MHz, $CDCl_3$) δ 170.41, 160.80, 159.72, 154.63 (d, *J* = 5.2 Hz), 147.95, 137.04, 130.67, 129.64, 129.21, 127.7 (q, *J* = 261 Hz), 120.13, 117.94, 109.81, 99.48, 62.58, 56.05, 33.39, 31.01, 28.48, 20.85. LC-MS, ESI$^+$, m/z 593 $[M+H]^+$, 595 $[M+H+2]^+$.

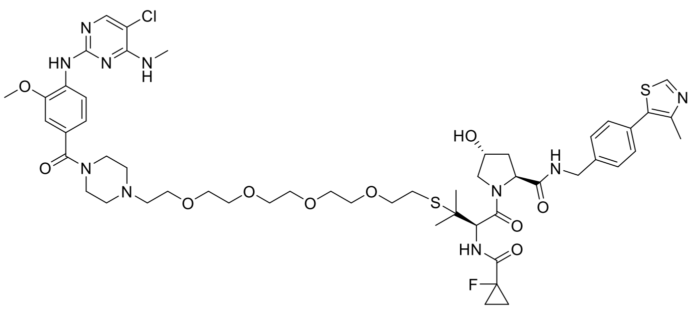

(2*S*,4*R*)-1-((*R*)-1-(4-(4-((5-chloro-4-(methylamino)pyrimidin-2-yl)amino)-3-methoxybenzoyl)piperazin-1-yl)-17-(1-fluorocyclopropane-1-carboxamido)-16,16-dimethyl-3,6,9,12-tetraoxa-15-thiaoctadecan-18-oyl)-4-hydroxy-N-(4-(4-methylthiazol-5-yl)benzyl)pyrrolidine-2-carboxamide (**SD74**)

7.55 mg **SD74** was obtained from 7.0 mg **10** and following general procedure 9 (51% yield). ^{1}H NMR (400 MHz, $CDCl_3$) δ 8.67 (s, 1H), 8.54 (d, *J* = 8.7 Hz, 1H), 7.92 (s, 1H), 7.65 (s, 1H), 7.42 – 7.30 (m, 5H), 7.19 (d, *J* = 7.8 Hz, 1H), 7.03 – 6.96 (m, 2H), 5.39 – 5.27 (m, 1H), 4.85 (d, *J* = 8.2 Hz, 1H), 4.76 (t, *J* = 7.7 Hz, 1H), 4.59 – 4.33 (m, 3H), 3.92 (s, 3H), 3.91 – 3.82 (m, 2H), 3.82 – 3.64 (m, 5H), 3.65 – 3.44 (m, 15H), 3.10 (d, *J* = 4.8 Hz, 3H), 2.85 – 2.05 (m, 8H), 2.51 (s, 3H), 2.46 – 2.39 (m, 1H), 2.25 – 2.15 (m, 1H), 1.39 – 1.20 (m, 10H); ^{13}C NMR (101 MHz, $CDCl_3$) δ 170.98, 170.66, 170.25 (d, *J* = 20.48 Hz), 169.96, 158.74, 157.91, 152.74, 150.39, 148.65, 147.61, 138.31, 131.69, 131.10, 129.62, 129.08, 128.33, 126.03, 120.40, 116.97, 109.76, 105.72, 79.43, 70.72, 70.68, 70.63, 70.55, 70.25, 69.88, 68.36, 59.35, 57.69, 56.51, 56.06, 55.94, 53.61, 48.10, 43.19, 36.91, 28.72, 28.25, 25.97, 25.19, 21.48, 16.22, 14.00, 13.91; HRMS (ESI$^+$) *m/z,* calcd for $C_{52}H_{70}ClFN_{10}O_{10}S_2$: 1113.4463 [M + H]$^+$, found 1113.4220.

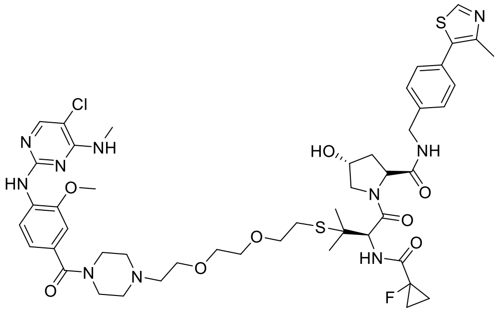

(2*S*,4*R*)-1-((R)-3-((2-(2-(2-(4-(4-((5-chloro-4-(methylamino)pyrimidin-2-yl)amino)-3-methoxybenzoyl)piperazin-1-yl)ethoxy)ethoxy)ethyl)thio)-2-(1-fluorocyclopropane-1-carboxamido)-3-methylbutanoyl)-4-hydroxy-N-(4-(4-methylthiazol-5-yl)benzyl)pyrrolidine-2-carboxamide (**SD75**)

To a solution of **3-6b** (8.29 mg, 0.0125 mmol) and **10** (6.68 mg, 0.0125 mmol) in DMF (0.2 ml) was added K_2CO_3 (5 mg, 0.036 mmol) and KI (0.2 mg, 0.0125 mmol). After stirring at 80 °C overnight, the resulting mixture was cooled down and purified with preparative HPLC under acidic condition (10-95 % CH_3CN in 0.1 % aq. HCO_2H) to give the title compound (2.12 mg, 17% yield). ^{1}H NMR (500 MHz, MeOD-*d*$_4$) δ 8.85 (s, 1H), 8.57 (d, *J* = 8.3 Hz, 1H), 7.84 (s, 1H), 7.45 – 7.36 (m, 4H), 7.06 – 6.99 (m, 2H), 4.91 (s, 1H), 4.58 (t, *J* = 8.4 Hz, 1H), 4.53 – 4.45 (m, 2H), 4.35 (d, *J* = 15.5 Hz, 1H), 3.96 – 3.92 (m, 3H), 3.87 (d, *J* = 2.6 Hz, 2H), 3.62 (t, *J* = 5.4 Hz, 3H), 3.59 – 3.50 (m, 6H), 3.34 (s, 3H), 3.03 (s, 3H), 2.76 (ddd, *J* = 12.7, 8.0, 4.9 Hz, 2H), 2.67 – 2.53 (m, 6H), 2.46 (s, 3H), 2.25 (ddd, *J* = 12.9, 7.7, 1.8 Hz, 1H), 2.08 (ddd, *J* = 13.3, 9.1, 4.5 Hz, 1H), 1.39 (d, *J* = 10.1 Hz, 6H), 1.36 – 1.22 (m, 4H). ^{13}C NMR (101 MHz, $CDCl_3$) δ 170.89, 170.72, 170.30, 169.99, 158.75, 157.90, 152.77, 150.39, 148.70, 147.62, 138.25, 131.18, 129.66, 129.07, 128.33, 126.05, 120.48, 116.96, 109.77, 77.48, 70.47, 70.32, 69.77, 59.26, 57.77, 56.28, 56.07, 55.96, 53.59, 48.12, 43.27, 36.58, 28.82, 28.25, 25.90, 25.43, 21.48, 16.25, 13.99 (d, *J* = 9.97 Hz), 13.95 (d, *J* = 9.93 Hz). HRMS (ESI$^+$) *m/z,* calcd for $C_{48}H_{62}ClFN_{10}O_8S_2$:1025.3944 [M + H]$^+$, found 1025.3915.

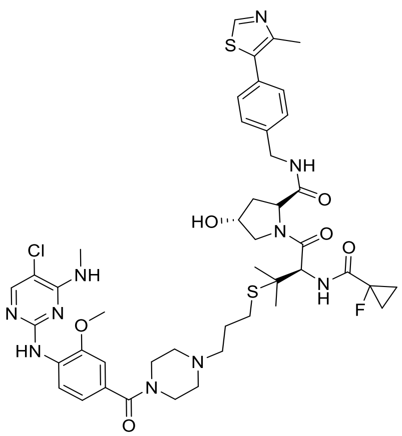

(2*S*,4*R*)-1-((*R*)-3-((3-(4-(4-((5-chloro-4-(methylamino)pyrimidin-2-yl)amino)-3-methoxybenzoyl)piperazin-1-yl)propyl)thio)-2-(1-fluorocyclopropane-1-carboxamido)-3-methylbutanoyl)-4-hydroxy-N-(4-(4-methylthiazol-5-yl)benzyl)pyrrolidine-2-carboxamide (**SD82)**

To a solution of **3-6d** (7.36 mg, 0.0125 mmol) and **10** (6.675 mg, 0.0125 mmol) in DMF (0.2 ml) was added K_2CO_3 (5 mg, 0.036 mmol) and KI (0.2 mg, 0.0125 mmol). After stirring at 80 °C overnight, the resulting mixture was cooled down and purified with preparative HPLC under acidic condition (10-80 % CH_3CN in 0.1 % aq. HCO_2H) to give the title compound (3.01 mg, 25% yield). ^{1}H NMR (500 MHz, MeOD-*d*$_4$) δ 8.87 (s, 1H), 8.60 (d, *J* = 8.3 Hz, 1H), 7.88 (s, 1H), 7.51 – 7.39 (m, 4H), 7.10 – 7.00 (m, 2H), 4.94 (s, 1H), 4.63 (t, *J* = 8.4 Hz, 1H), 4.58 (d, *J* = 15.6 Hz, 1H), 4.53 (s, 1H), 4.37 (d, *J* = 15.7 Hz, 1H), 3.98 (s, 3H), 3.94 – 3.86 (m, 2H), 3.78 – 3.62 (m, 4H), 3.06 (s, 3H), 2.76 – 2.55 (m, 8H), 2.49 (s, 3H), 2.29 (dd, *J* = 12.9, 7.8 Hz, 1H), 2.12 (ddd, *J* = 13.3, 9.2, 4.4 Hz, 1H), 1.81 – 1.72 (m, 2H), 1.46 (d, *J* = 10.8 Hz, 6H), 1.40 – 1.28 (m, 4H). HRMS (ESI$^+$) *m/z,* calcd for $C_{45}H_{56}ClFN_{10}O_6S_2$: 951.3571 [M + H]$^+$, found 951.3565.

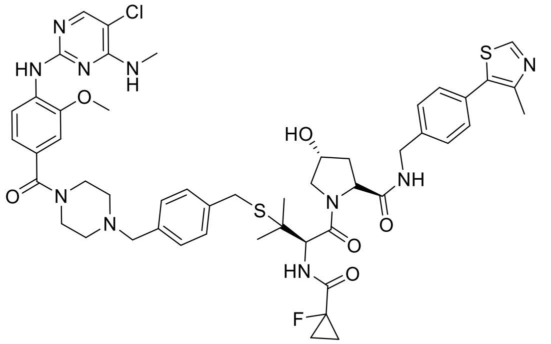

(2*S*,4*R*)-1-((*R*)-3-((4-((4-(4-((5-chloro-4-(methylamino)pyrimidin-2-yl)amino)-3-methoxybenzoyl)piperazin-1-yl)methyl)benzyl)thio)-2-(1-fluorocyclopropane-1-carboxamido)-3-methylbutanoyl)-4-hydroxy-N-(4-(4-methylthiazol-5-yl)benzyl)pyrrolidine-2-carboxamide (**SD100**)

4.2 mg **SD100** was obtained from 6.0 mg **10** and 6 mg **3-6e** following general procedure 9 (39% yield). ^{1}H NMR (400 MHz, $CDCl_3$) δ 8.67 (s, 1H), 8.53 (d, *J* = 8.8 Hz, 1H), 7.92 (s, 1H), 7.64 (s, 1H), 7.33 (dt, *J* = 16.5, 5.2 Hz, 4H), 7.25 – 7.19 (m, 6H), 7.02 – 6.98 (m, 2H), 5.32 – 5.24 (m, 1H), 4.73 (t, *J* = 7.8 Hz, 1H), 4.63 (d, *J* = 7.5 Hz, 1H), 4.53 – 4.31 (m, 3H), 3.92 (s, 3H), 3.87 (d, *J* = 11.2 Hz, 1H), 3.81 – 3.54 (m, 7H), 3.48 (s, 2H), 3.10 (d, *J* = 4.9 Hz, 3H), 2.53 – 2.36 (m, 8H), 2.24 – 2.11 (m, 1H), 1.38 (s, 6H), 1.35 – 1.25 (m, 4H). ^{13}C NMR (101 MHz, $CDCl_3$) δ 170.65, 170.45, 170.16, 158.73, 157.96, 152.79, 150.41, 148.69, 147.58, 138.12, 136.98, 136.47, 131.69, 131.51, 131.21, 129.68, 129.08, 128.25, 128.00, 120.33, 116.94, 109.77, 105.67, 79.44, 77.37, 70.19, 62.70, 58.96, 56.51, 56.46, 56.03, 53.34, 48.77, 43.32, 36.59, 33.43, 28.23, 25.81, 25.49, 16.22, 14.06 (d, *J* = 11.94 Hz), 13.95 (d, *J* = 10.48 Hz). HRMS (ESI$^+$) m/z, calcd for $C_{50}H_{59}ClFN_{10}O_6S_2$ 1013.3727 [M + H]$^+$, found 1013.3849.

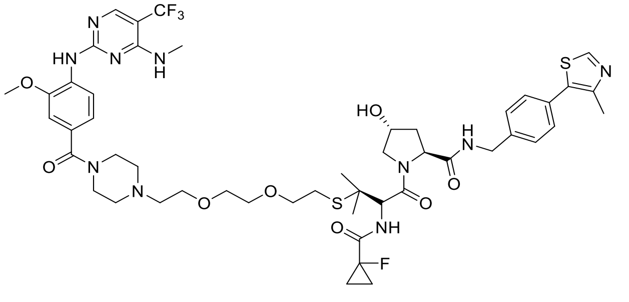

(2*S*,4*R*)-1-((*S*)-2-(1-fluorocyclopropane-1-carboxamido)-3-((2-(2-(2-(4-(3-methoxy-4-((4-(methylamino)-5-(trifluoromethyl)pyrimidin-2-yl)amino)benzoyl)piperazin-1-yl)ethoxy)ethoxy)ethyl)thio)-3-methylbutanoyl)-4-hydroxy-N-(4-(4-methylthiazol-5-yl)benzyl)pyrrolidine-2-carboxamide (**XL01078B**)

3.6 mg **XL01078B** was obtained from 5 mg **10** and 6.5 mg **3-6i** following general procedure 9 (36% yield). 1H NMR (500 MHz, $CDCl_3$) δ 8.68 (s, 1H), 8.55 (d, *J* = 8.8 Hz, 1H), 8.20 – 8.13 (m, 2H), 7.88 (s, 1H), 7.39 – 7.33 (m, 5H), 7.20 (dd, *J* = 8.3, 3.2 Hz, 1H), 7.03 – 6.98 (m, 2H), 5.25 (d, *J* = 3.6 Hz, 1H), 4.85 (d, *J* = 8.2 Hz, 1H), 4.76 (dd, *J* = 8.3, 6.3 Hz, 1H), 4.59 – 4.52 (m, 1H), 4.44 (qt, *J* = 12.8, 6.4 Hz, 2H), 3.93 (s, 3H), 3.91 – 3.87 (m, 2H), 3.72 (s, 4H), 3.66 – 3.57 (m, 4H), 3.56 – 3.52 (m, 4H), 3.50 – 3.46 (m, 2H), 3.11 (d, *J* = 4.7 Hz, 3H), 2.76 (ddd, *J* = 16.4, 12.4, 5.9 Hz, 2H), 2.69 (t, *J* = 5.4 Hz, 2H), 2.63 (s, 2H), 2.52 (s, 3H), 2.50 – 2.43 (m, 1H), 2.21 – 2.14 (m, 1H), 1.37 – 1.28 (m, 10H); ^{13}C NMR (126 MHz, $CDCl_3$) δ 170.92, 170.51, 170.29 (d, *J* = 20.6 Hz), 170.01, 164.24, 160.75, 159.75, 154.57 (q, *J* = 4.8 Hz), 150.49, 148.60, 148.03, 138.24, 131.79, 131.12, 130.86, 129.66, 128.59, 128.33,125.05 (q, *J* = 270.5 Hz), 120.21, 118.03, 109.83, 79.20, 70.50, 70.44, 70.32, 69.77, 68.36, 59.27, 57.59, 56.28, 56.11, 55.97, 53.48, 48.06, 43.27, 36.62, 28.78, 28.52, 25.90, 25.34, 16.17, 14.00 (d, *J* = 9.9 Hz), 13.95 (d, *J* = 9.7 Hz); ^{19}F NMR (471 MHz, $CDCl_3$) δ -61.53, -197.74; HRMS (ESI$^+$) m/z, calcd for $C_{49}H_{62}F_4N_{10}O_8S_2$: 1059.4202 [M + H]+, found 1059.3872.

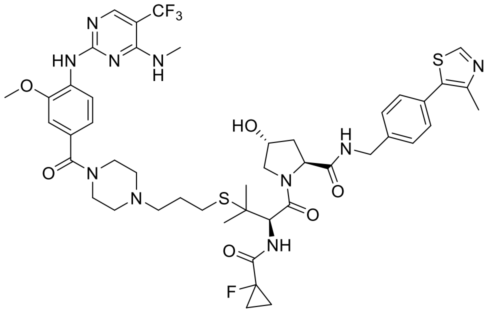

(2*S*,4*R*)-1-((*R*)-2-(1-fluorocyclopropane-1-carboxamido)-3-((3-(4-(3-methoxy-4-((4-(methylamino)-5-(trifluoromethyl)pyrimidin-2-yl)amino)benzoyl)piperazin-1-yl)propyl)thio)-3-methylbutanoyl)-4-hydroxy-N-(4-(4-methylthiazol-5-yl)benzyl)pyrrolidine-2-carboxamide (**XL01072**)

6.1 mg **XL01072** was obtained from 7 mg **10** and 7 mg **3-6j** following general procedure 9 (47% yield). ^{1}H NMR (400 MHz, DMSO-d_6) δ 8.96 (s, 1H), 8.54 (t, *J* = 5.9 Hz, 1H), 8.30 (d, *J* = 8.2 Hz, 1H), 8.19 (s, 1H), 8.05 (s, 1H), 7.47 – 7.34 (m, 5H), 7.22 – 7.16 (m, 1H), 7.03 (d, *J* = 1.7 Hz, 1H), 6.97 (dd, *J* = 8.2, 1.7 Hz, 1H), 5.19 (br, s, 1H), 4.79 (d, *J* = 9.2 Hz, 1H), 4.51 – 4.34 (m, 3H), 4.24 (dd, *J* = 15.7, 5.5 Hz, 1H), 3.89 (s, 3H), 3.74 (dd, *J* = 10.5, 4.0 Hz, 1H), 3.64 (d, *J* = 10.8 Hz, 1H), 3.58 – 3.42 (m, 4H), 2.92 (d, *J* = 4.4 Hz, 3H), 2.56 (t, *J* = 7.2 Hz, 2H), 2.44 (s, 3H), 2.37 – 2.26 (m, 6H), 2.13 – 2.04 (m, 1H), 1.97 – 1.86 (m, 1H), 1.62 – 1.51 (m, 2H), 1.44 – 1.32 (m, 8H), 1.25 – 1.18 (m, 2H); ^{13}C NMR (101 MHz, DMSO-d_6) δ 171.44, 168.62, 168.05 (d, $^2J_{C\text{-}F}$ = 20.7 Hz,), 167.79, 160.56, 158.42, 154.27 (d, *J* = 5.4 Hz), 151.32, 148.30, 147.71, 139.26, 131.04, 129.89, 129.72, 129.43, 128.69, 127.96, 127.46 (q, *J* = 267 Hz), 127.40, 119.34, 116.07, 109.84, 78.02 (d, $^1J_{C\text{-}F}$ = 233.0 Hz,), 68.78, 58.94, 56.59, 56.46, 55.99, 54.82, 52.61, 49.05, 41.61, 37.88, 27.99, 26.50, 26.45, 25.64, 24.19, 15.87, 12.93 (d, $^2J_{C\text{-}F}$ =

10.6 Hz,), 12.78 (d, $^2J_{C-F}$ = 10.8 Hz,); ^{19}F NMR (471 MHz, $CDCl_3$) δ -61.50, -197.76. HRMS (ESI$^+$) *m/z,* calcd for $C_{46}H_{56}F_4N_{10}O_6S_2$: 985.3835 [M + H]$^+$, found 985.3813.

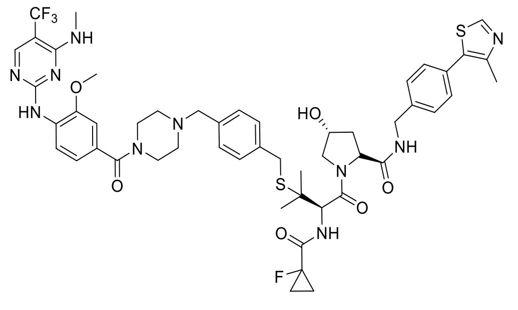

(2*S*,4*R*)-1-((*R*)-2-(1-fluorocyclopropane-1-carboxamido)-3-((4-((4-(3-methoxy-4-((4-(methylamino)-5-(trifluoromethyl)pyrimidin-2-yl)amino)benzoyl)piperazin-1-yl)methyl)benzyl)thio)-3-methylbutanoyl)-4-hydroxy-N-(4-(4-methylthiazol-5-yl)benzyl)pyrrolidine-2-carboxamide (**XL01070B**)

7.8 mg **XL01070B** was obtained from 7 mg **10**and 8.54 mg **3-6k** following general procedure 9 (57% yield). ^{1}H NMR (400 MHz, $CDCl_3$) δ 8.66 (s, 1H), 8.54 (d, *J* = 8.2 Hz, 1H), 8.18 (d, *J* = 0.7 Hz, 1H), 7.83 (s, 1H), 7.36 – 7.28 (m, 4H), 7.26 – 7.18 (m, 6H), 7.03 – 6.97 (m, 2H), 5.26 – 5.19 (m, 1H), 4.73 (t, *J* = 7.8 Hz, 1H), 4.63 (d, *J* = 7.6 Hz, 1H), 4.54 – 4.33 (m, 3H), 3.93 (s, 3H), 3.87 (d, *J* = 11.3 Hz, 1H), 3.82 – 3.54 (m, 7H), 3.48 (s, 2H), 3.11 (d, *J* = 4.7 Hz, 3H), 2.52 – 2.37 (m, 8H), 2.21 – 2.11 (m, 1H), 1.41 – 1.24 (m, 10H); ^{13}C NMR (101 MHz, $CDCl_3$) δ 170.67, 170.64, 170.42, 170.15, 160.84, 159.75, 154.69 (q, *J* = 4.9 Hz), 150.40, 148.68, 147.94, 138.12, 136.95, 136.48, 131.69, 131.20, 130.68, 129.67, 129.63, 129.08, 128.24, 127.73 (q, *J* = 262 Hz), 120.16, 117.92, 109.83, 78.40 (d, *J* = 207.5 Hz), 70.19, 62.69, 58.98, 56.53, 56.46, 56.07, 53.34, 48.81, 43.31, 36.63, 33.42, 28.49, 25.84, 25.47, 16.21, 14.04 (d, *J* = 10.3 Hz), 13.94 (d, 10.3 Hz); ^{19}F NMR (471 MHz, $CDCl_3$) δ -61.49, -197.70; HRMS (ESI$^+$) *m/z,* calcd for $C_{51}H_{58}F_4N_{10}O_6S_2$ 1047.3991 [M + H]$^+$, found 1047.4052.

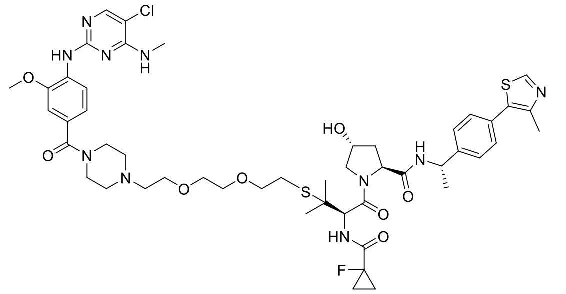

(2*S*,4*R*)-1-((*R*)-3-((2-(2-(2-(4-(4-((5-chloro-4-(methylamino)pyrimidin-2-yl)amino)-3-methoxybenzoyl)piperazin-1-yl)ethoxy)ethoxy)ethyl)thio)-2-(1-fluorocyclopropane-1-carboxamido)-3-methylbutanoyl)-4-hydroxy-N-((S)-1-(4-(4-methylthiazol-5-yl)phenyl)ethyl)pyrrolidine-2-carboxamide (**XL01119**)

6.7 mg **XL01119** was obtained from 8 mg **10** and 10.6 mg **3-6b** material (40% yield). ^{1}H NMR (500 MHz, $CDCl_3$) δ 8.66 (s, 1H), 8.54 (d, *J* = 8.7 Hz, 1H), 7.92 (s, 1H), 7.63 (s, 1H), 7.42 – 7.32 (m, 5H), 7.22 (dd, *J* = 8.2, 3.2 Hz, 1H), 7.03 – 6.98 (m, 2H), 5.30 (q, *J* = 4.5 Hz, 1H), 5.10 (p, *J* = 7.0 Hz, 1H), 4.88 (d, *J* = 8.1 Hz, 1H), 4.74 (dd, *J* = 8.4, 6.0 Hz, 1H), 4.54 (p, *J* = 4.4 Hz, 1H), 3.92 (s, 3H), 3.90 (d, *J* = 4.2 Hz, 2H), 3.72 – 3.55 (m, 12H), 3.10 (d, *J* = 4.9 Hz, 3H), 2.90 – 2.78 (m, 2H), 2.64 – 2.60 (m, 2H), 2.57 – 2.41 (m, 8H), 2.11 (ddd, *J* = 13.2, 8.5, 4.6 Hz, 1H), 1.48 (d, *J* = 7.0 Hz, 3H), 1.42 (s, 3H), 1.39 (s, 3H), 1.37 – 1.28 (m, 4H). ^{13}C NMR (126 MHz, $CDCl_3$) δ 170.62, 170.39, 170.23, 169.83, 158.73, 157.96, 152.82, 150.34, 148.68, 147.58, 143.21, 131.79, 131.56, 131.02, 129.67, 127.88, 126.68, 120.36, 116.93, 109.75, 105.71, 79.22, 70.59, 70.53, 70.39, 69.72, 68.88, 59.21, 57.88, 56.22, 56.04, 53.80, 48.88, 47.98, 36.26, 28.92, 28.23, 25.97, 25.47, 22.26, 16.25, 14.04 (d, *J* = 10.3 Hz), 13.92 (d, *J* = 9.8 Hz). ^{19}F NMR (471 MHz, $CDCl_3$) δ -197.73. HRMS (ESI$^+$) m/z, calcd for $C_{49}H_{64}ClFN_{10}O_8S_2$: 1039.4095 [M + H]$^+$, found 1039.3852.

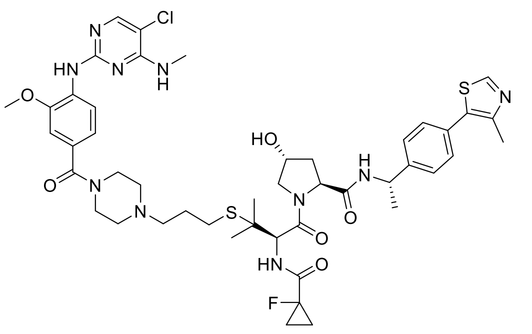

(2*S*,4*R*)-1-((R)-3-((3-(4-(4-((5-chloro-4-(methylamino)pyrimidin-2-yl)amino)-3-methoxybenzoyl)piperazin-1-yl)propyl)thio)-2-(1-fluorocyclopropane-1-carboxamido)-3-methylbutanoyl)-4-hydroxy-N-((S)-1-(4-(4-methylthiazol-5-yl)phenyl)ethyl)pyrrolidine-2-carboxamide (**XL01118**)

3.1 mg **XL01118** was obtained from 8 mg **1-8b** and 8 mg **3-6d** following general procedure 9 (20% yield). ^{1}H NMR (500 MHz, $CDCl_3$) δ 8.69 (s, 1H), 8.57 (d, *J* = 8.2 Hz, 1H), 7.95 (s, 1H), 7.68 (s, 1H), 7.45 – 7.38 (m, 4H), 7.33 (d, *J* = 7.9 Hz, 1H), 7.32 – 7.28 (m, 1H), 7.06 – 6.98 (m, 2H), 5.32 (q, *J* = 4.5 Hz, 1H), 5.13 (p, *J* = 7.0 Hz, 1H), 4.82 – 4.76 (m, 2H), 4.56 (s, 1H), 4.09 (d, *J* = 11.3 Hz, 1H), 3.95 (s, 3H), 3.82 – 3.57 (m, 5H), 3.13 (d, *J* = 4.9 Hz, 3H), 2.71 – 2.59 (m, 2H), 2.55 (s, 3H), 2.54 – 2.40 (m, 8H), 2.21 – 2.13 (m, 1H), 1.85 – 1.72 (m, 2H), 1.52 (d, *J* = 6.9 Hz, 3H), 1.48 (s, 3H), 1.46 (s, 3H), 1.41 – 1.30 (m, 4H). ^{13}C NMR (126 MHz, $CDCl_3$) δ 170.69 (d, *J* = 21.0 Hz), 170.66, 170.30, 169.64, 158.74, 157.94, 152.76, 150.41, 148.69, 147.62, 143.13, 131.74, 131.61, 131.09, 129.72, 127.82, 126.63, 120.35, 116.95, 109.77, 105.73, 79.24, 70.27, 58.97, 57.28, 56.64, 56.46, 56.04, 53.34, 48.94, 48.02, 36.28, 29.78, 28.24, 26.59, 26.26, 25.79, 22.41, 16.24, 14.15 (d, *J* = 10.4 Hz), 13.98 (d, *J* = 10.2 Hz). ^{19}F NMR (471 MHz, $CDCl_3$) δ -197.76. HRMS (ESI$^+$) m/z, calcd for $C_{46}H_{58}ClFN_{10}O_6S_2$: 965.3727 $[M + H]^+$, found 965.4213.

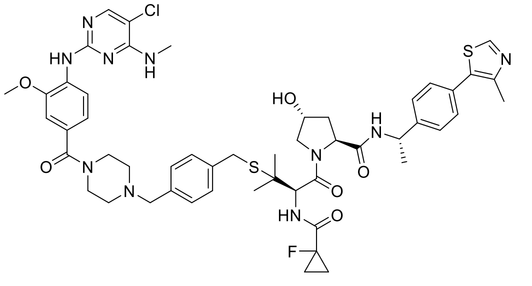

(2*S*,4*R*)-1-((*R*)-3-((4-((4-(4-((5-chloro-4-(methylamino)pyrimidin-2-yl)amino)-3-methoxybenzoyl)piperazin-1-yl)methyl)benzyl)thio)-2-(1-fluorocyclopropane-1-carboxamido)-3-methylbutanoyl)-4-hydroxy-N-((S)-1-(4-(4-methylthiazol-5-yl)phenyl)ethyl)pyrrolidine-2-carboxamide (**XL01120**)

9.5 mg **XL01120** was obtained from 8 mg **1-8b** and 8 mg **3-6e** following general procedure 9 (65% yield). ^{1}H NMR (500 MHz, $CDCl_3$) δ 8.66 (s, 1H), 8.53 (d, *J* = 8.8 Hz, 1H), 7.91 (s, 1H), 7.62 (s, 1H), 7.38 (d, *J* = 8.3 Hz, 2H), 7.34 (d, *J* = 8.3 Hz, 2H), 7.31 – 7.23 (m, 6H), 7.02 – 6.97 (m, 2H), 5.30 (q, *J* = 4.7 Hz, 1H), 5.06 (p, *J* = 7.0 Hz, 1H), 4.71 (t, *J* = 7.8 Hz, 1H), 4.67 (d, *J* = 7.5 Hz, 1H), 4.48 – 4.42 (m, 1H), 3.91 (s, 3H), 3.88 (d, *J* = 11.3 Hz, 1H), 3.82 (q, *J* = 12.4 Hz, 2H), 3.75 – 3.54 (m, 5H), 3.49 (s, 2H), 3.09 (d, *J* = 4.9 Hz, 3H), 2.51 (s, 3H), 2.49 – 2.34 (m, 5H), 2.15 – 2.07 (m, 1H), 1.48 (d, *J* = 6.7 Hz, 6H), 1.40 (d, *J* = 6.9 Hz, 3H), 1.37 – 1.25 (m, 4H); ^{13}C NMR (126 MHz, $CDCl_3$) δ 170.62, 170.33 (d, *J* = 19.0 Hz), 170.22, 169.75, 158.71, 157.95, 152.79, 150.36, 148.66, 147.55, 143.17, 137.08, 136.35, 131.75, 131.49, 131.03, 129.67, 129.60, 129.12, 128.00, 126.63, 120.33, 116.91, 109.75, 105.68, 78.28 (d, *J* = 231.5 Hz), 62.71, 59.06, 56.61, 56.52, 56.02, 53.33, 48.93, 48.85, 36.45, 33.48, 28.21, 25.92, 25.54, 22.30, 16.23, 14.01 (d, *J* = 10.2Hz), 13.94 (d, *J* = 10.2 Hz). ^{19}F NMR (471 MHz, $CDCl_3$) δ -197.63. HRMS (ESI$^+$) *m/z,* calcd for $C_{51}H_{60}ClFN_{10}O_6S_2$: 1027.3884 $[M + H]^+$, found 1027.4164.

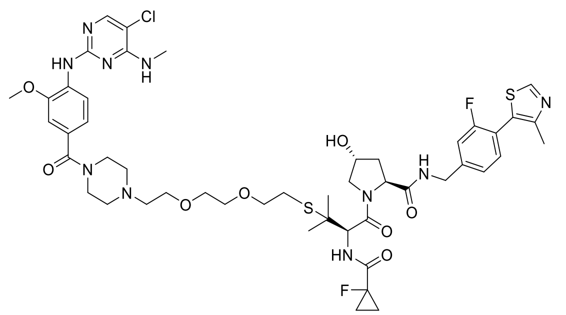

(2*S*,4*R*)-1-((*R*)-3-((2-(2-(2-(4-(4-((5-chloro-4-(methylamino)pyrimidin-2-yl)amino)-3-methoxybenzoyl)piperazin-1-yl)ethoxy)ethoxy)ethyl)thio)-2-(1-fluorocyclopropane-1-carboxamido)-3-methylbutanoyl)-N-(3-fluoro-4-(4-methylthiazol-5-yl)benzyl)-4-hydroxypyrrolidine-2-carboxamide (**XL01123**)

5 mg **XL01123** was obtained from 8 mg **1-8a** and 11.5 mg **3-6b** following general procedure 9 (33% yield). ^{1}H NMR (500 MHz, $CDCl_3$) δ 8.76 (s, 1H), 8.54 (d, *J* = 8.7 Hz, 1H), 7.92 (s, 1H), 7.63 (s, 1H), 7.44 (t, *J* = 5.9 Hz, 1H), 7.32 – 7.27 (m, 1H), 7.18 (dd, *J* = 8.3, 3.1 Hz, 1H), 7.15 – 7.11 (m, 2H), 7.02 – 6.98 (m, 2H), 5.32 – 5.27 (m, 1H), 4.87 (d, *J* = 8.1 Hz, 1H), 4.76 (dd, *J* = 8.4, 6.2 Hz, 1H), 4.58 – 4.52 (m, 1H), 4.50 – 4.40 (m, 2H), 3.92 (s, 3H), 3.91 – 3.88 (m, 2H), 3.80 – 3.45 (m, 13H), 3.10 (d, *J* = 4.9 Hz, 3H), 2.85 – 2.72 (m, 2H), 2.61 (s, 2H), 2.57 – 2.43 (m, 5H), 2.42 (d, *J* = 1.1 Hz, 3H), 2.21 – 2.14 (m, 1H), 1.36 (s, 3H), 1.34 (s, 3H), 1.33 – 1.27 (m, 4H); ^{13}C NMR (126 MHz, $CDCl_3$) δ 171.05, 170.65, 170.21, 170.14, 163.36, 158.75, 157.93, 152.75, 151.85, 151.07, 147.62, 141.50, 132.40 (d, *J = 2.5 Hz*), 131.61, 127.76, 124.51, 123.63 (*J = 3.3 Hz*), 120.37, 118.59 (d, *J = 15.7 Hz*), 116.99, 115.5 (d, *J = 22.9 Hz*), 109.76, 105.71, 79.22, 70.56, 70.45, 70.22, 69.67, 68.65, 59.26, 57.77, 56.19, 56.06, 55.96, 53.63, 47.98, 42.88, 36.60, 28.89, 28.25, 26.00, 25.20, 16.13, 14.04 (d, *J* = 9.85 Hz), 13.96 (d, *J* = 9.84 Hz); HRMS (ESI^{+}) *m/z,* calcd for $C_{48}H_{61}ClF_2N_{10}O_8S_2$: 1043.3845 [M + H]$^{+}$, found 1043.3733.

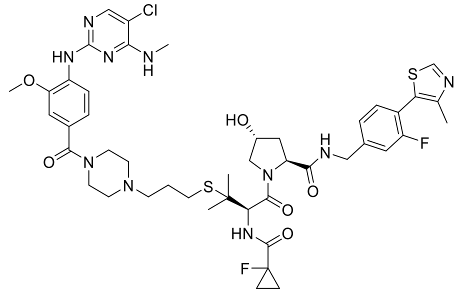

(2*S*,4*R*)-1-((*R*)-3-((3-(4-(4-((5-chloro-4-(methylamino)pyrimidin-2-yl)amino)-3-methoxybenzoyl)piperazin-1-yl)propyl)thio)-2-(1-fluorocyclopropane-1-carboxamido)-3-methylbutanoyl)-N-(3-fluoro-4-(4-methylthiazol-5-yl)benzyl)-4-hydroxypyrrolidine-2-carboxamide (**XL01122**)

1.4 mg **XL01122** was obtained from 8 mg **1-8a** and 7.2 mg **3-6d** following general procedure 9 (10% yield). ^{1}H NMR (500 MHz, $CDCl_3$) δ 8.76 (s, 1H), 8.54 (d, *J* = 8.1 Hz, 1H), 7.93 (s, 1H), 7.65 (s, 1H), 7.36 – 7.28 (m, 2H), 7.23 (dd, *J* = 7.8, 3.2 Hz, 1H), 7.16 – 7.10 (m, 2H), 7.02 – 6.97 (m, 2H), 5.32 – 5.27 (m, 1H), 4.81 – 4.70 (m, 2H), 4.59 – 4.47 (m, 2H), 4.41 (dd, *J* = 15.3, 5.7 Hz, 1H), 4.08 (d, *J* = 11.4 Hz, 1H), 3.93 (s, 3H), 3.76 – 3.52 (m, 5H), 3.11 (d, *J* = 4.9 Hz, 3H), 2.62 – 2.47 (m, 4H), 2.47 – 2.36 (m, 9H), 2.26 – 2.20 (m, 1H), 1.74 – 1.64 (m, 2H), 1.37 (s, 3H), 1.36 (s, 3H), 1.34 – 1.26 (m, 4H); ^{13}C NMR (126 MHz, $CDCl_3$) δ 170.81, 170.64, 170.61, 170.26, 158.75, 157.95, 152.78, 151.88, 151.12, 147.62, 141.46, 141.41, 132.45, 132.43, 131.61, 124.41, 123.55 (d, *J* = 3.0 Hz), 120.35, 116.95, 115.42 (d, *J* = 22.4 Hz), 109.77, 105.73, 79.24, 70.31, 59.00, 57.24, 56.69, 56.45, 56.05, 53.27, 47.98, 42.95, 36.62, 28.24, 26.17, 25.73, 25.53, 16.15, 14.15 (d, *J* = 10.6 Hz), 14.00 (d, *J* = 10.5 Hz); HRMS (ESI^{+}) *m/z,* calcd for $C_{45}H_{55}ClF_2N_{10}O_6S_2$: 969.3477 [M + H]$^{+}$, found 969.3772.

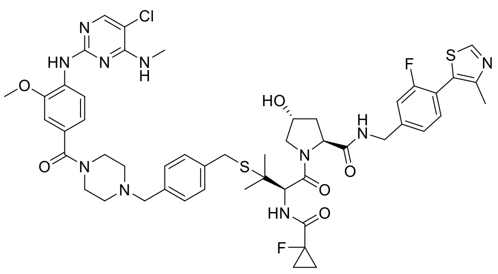

(2*S*,4*R*)-1-((*R*)-3-((4-((4-(4-((5-chloro-4-(methylamino)pyrimidin-2-yl)amino)-3-methoxybenzoyl)piperazin-1-yl)methyl)benzyl)thio)-2-(1-fluorocyclopropane-1-carboxamido)-3-methylbutanoyl)-N-(3-fluoro-4-(4-methylthiazol-5-yl)benzyl)-4-hydroxypyrrolidine-2-carboxamide (**XL01121**)

12.2 mg **XL01121** was obtained from 8 mg **1-8a** and 8 mg **3-6e** following general procedure 9 (83% yield). ^{1}H NMR (500 MHz, $CDCl_3$) δ 8.79 (s, 1H), 8.57 (d, *J* = 8.8 Hz, 1H), 7.95 (s, 1H), 7.66 (s, 1H), 7.41 (t, *J* = 6.0 Hz, 1H), 7.32 – 7.26 (m, 6H), 7.12 (d, *J* = 9.1 Hz, 2H), 7.06 – 7.00 (m, 2H), 5.34 (q, *J* = 4.7 Hz, 1H), 4.76 (t, *J* = 7.9 Hz, 1H), 4.66 (d, *J* = 7.5 Hz, 1H), 4.51 – 4.48 (m, 1H), 4.49 – 4.43 (m, 1H), 4.38 (dd, *J* = 15.4, 5.8 Hz, 1H), 3.95 (s, 3H), 3.90 (d, *J* = 11.2 Hz, 1H), 3.80 (dd, *J* = 12.5 Hz, 2H), 3.75 – 3.57 (m, 5H), 3.51 (s, 2H), 3.14 (d, *J* = 4.9 Hz, 3H), 2.56 – 2.38 (m, 8H), 2.24 – 2.16 (m, 1H), 1.47 (s, 3H), 1.43 (s, 3H), 1.39 – 1.29 (m, 4H); ^{13}C NMR (126 MHz, $CDCl_3$) δ 171.04, 170.62, 170.46 (d, *J* = 20.9 Hz), 170.13, 159.86 (d, *J* = 250.1 Hz), 158.71, 157.95, 152.79, 151.82, 151.05, 147.54, 141.49 (d, *J* = 7.0 Hz), 137.04, 136.41, 132.33 (d, *J* = 2.4 Hz), 131.49, 129.60, 129.03, 127.99, 124.45, 123.39 (d, *J* = 3.5 Hz), 120.32, 118.51 (d, *J* = 15.7 Hz), 116.92, 115.30 (d, *J* = 23.3 Hz), 109.74, 105.67, 78.28 (d, *J* = 229.1 Hz), 70.17, 62.69, 59.15, 56.66, 56.47, 56.02, 53.30, 48.94, 42.84, 36.86, 33.39, 28.21, 25.75, 25.36, 16.11 (d, *J* = 2.8 Hz), 13.96 (d, *J* = 10.8 Hz), 13.94 (d, *J* = 10.9 Hz). HRMS (ESI^{+}) *m/z,* calcd for C50H57ClF2N10O6S2: 1031.3633 [M + H]$^{+}$, found 1031.3403.

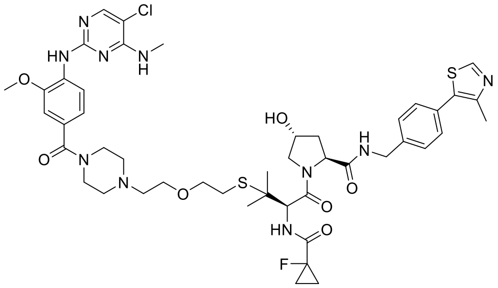

(2*S*,4*R*)-1-((*R*)-3-((2-(2-(4-(4-((5-chloro-4-(methylamino)pyrimidin-2-yl)amino)-3-methoxybenzoyl)piperazin-1-yl)ethoxy)ethyl)thio)-2-(1-fluorocyclopropane-1-carboxamido)-3-methylbutanoyl)-4-hydroxy-N-(4-(4-methylthiazol-5-yl)benzyl)pyrrolidine-2-carboxamide (**XL01131**)

5.9 mg **XL01131** was obtained from 8 mg **10** and 9.3 mg **3-6a** following general procedure 9 (40% yield). ^{1}H NMR (500 MHz, $CDCl_3$) δ 8.70 (s, 1H), 8.55 (d, *J* = 8.5 Hz, 1H), 7.94 (s, 1H), 7.71 (s, 1H), 7.39 (dd, *J* = 20.4, 8.2 Hz, 4H), 7.31 (d, *J* = 5.9 Hz, 1H), 7.25 (dd, *J* = 8.1, 3.2 Hz, 1H), 7.05 – 6.99 (m, 2H), 5.37 – 5.31 (m, 1H), 4.82 (d, *J* = 8.1 Hz, 1H), 4.80 – 4.74 (m, 1H), 4.58 – 4.52 (m, 1H), 4.48 (d, *J* = 5.9 Hz, 2H), 3.99 (d, *J* = 12.1 Hz, 1H), 3.95 (s, 3H), 3.83 (dd, *J* = 10.9, 4.3 Hz, 1H), 3.79 – 3.65 (m, 4H), 3.61 (t, *J* = 5.4 Hz, 2H), 3.59 – 3.55 (m, 1H), 3.53 – 3.48 (m, 1H), 3.36 (s, 2H), 3.13 (d, *J* = 4.9 Hz, 3H), 2.80 – 2.70 (m, 2H), 2.68 (t, *J* = 5.4 Hz, 2H), 2.62 (s, 2H), 2.54 (s, 3H), 2.52 – 2.46 (m, 1H), 2.25 – 2.18 (m, 1H), 1.40 – 1.31 (m, 10H); ^{13}C NMR (126 MHz, $CDCl_3$) δ 170.80, 170.69, 170.46 (d, *J* = 20.8 Hz), 169.99, 158.76, 157.87, 152.62, 150.48, 148.64, 147.69, 138.22, 131.73, 131.64, 131.17, 129.67, 128.27, 127.64, 120.36, 117.05, 109.80, 105.74, 79.22, 70.32, 70.04, 68.59, 59.18, 57.50, 56.49, 56.22, 56.06, 53.54, 48.16, 43.29, 36.66, 28.48, 28.25, 25.87, 25.52, 16.20, 14.06 (d, *J* = 10.6 Hz), 13.97 (d, *J* = 10.3 Hz). HRMS (ESI^{+}) *m/z,* calcd for $C_{46}H_{58}ClFN_{10}O_7S_2$: 981.3676 [M + H]$^{+}$, found 981.4273.

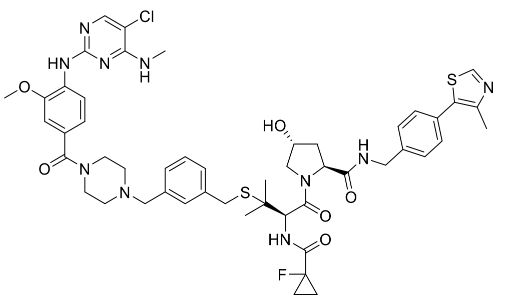

(2*S*,4*R*)-1-((*R*)-3-((3-((4-(4-((5-chloro-4-(methylamino)pyrimidin-2-yl)amino)-3-methoxybenzoyl)piperazin-1-yl)methyl)benzyl)thio)-2-(1-fluorocyclopropane-1-carboxamido)-3-methylbutanoyl)-4-hydroxy-N-(4-(4-methylthiazol-5-yl)benzyl)pyrrolidine-2-carboxamide (**XL01111**)

7.5 mg **XL01111** was obtained from 10 mg **10** and 9.5 mg **3-6f** following general procedure 9 (42%). ^{1}H NMR (400 MHz, $CDCl_3$) δ 8.66 (s, 1H), 8.53 (d, *J* = 8.4 Hz, 1H), 7.91 (s, 1H), 7.62 (s, 1H), 7.34 – 7.26 (m, 5H), 7.26 – 7.15 (m, 5H), 7.02 – 6.96 (m, 2H), 5.37 – 5.23 (m, 1H), 4.84 – 4.68 (m, 2H), 4.50 – 4.32 (m, 3H), 3.96 – 3.88 (m, 4H), 3.82 – 3.71 (m, 3H), 3.64 (s, 4H), 3.51 (d, *J* = 13.0 Hz, 1H), 3.44 (d, *J* = 13.0 Hz, 1H), 3.10 (d, *J* = 4.9 Hz, 3H), 2.55 – 2.36 (m, 8H), 2.23 – 2.11 (m, 1H), 1.41 – 1.25 (m, 10H). ^{13}C NMR (101 MHz, $CDCl_3$) δ 170.70, 170.63, 170.50 (d, *J* = 20.8 Hz), 170.04, 158.72, 157.95, 152.79, 150.38, 148.68, 147.57, 138.21, 138.10, 137.31, 131.67, 131.53, 131.16, 129.81, 129.65, 128.86, 128.41, 128.20, 127.92, 120.33, 116.91, 109.75, 105.70, 78.40 (d, *J* = 209.1 Hz), 69.99, 62.89, 59.09, 56.43, 56.25, 56.03, 48.98, 43.25, 36.67, 33.60, 28.22, 25.73, 25.63, 16.22, 14.07 (d, *J* = 9.3 Hz), 13. 95 (d, *J* = 8.9 Hz). ^{19}F NMR (471 MHz, $CDCl_3$) δ -197.75. HRMS (ESI$^+$) *m/z*, calcd for $C_{50}H_{58}ClFN_{10}O_6S_2$:1013.3727 [M + H]$^+$, found 1013.3893.

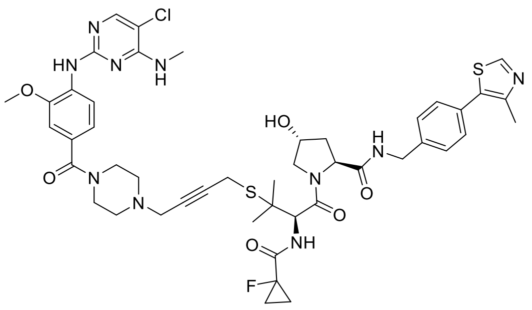

(2*S*,4*R*)-1-((*R*)-3-((4-(4-(4-((5-chloro-4-(methylamino)pyrimidin-2-yl)amino)-3-methoxybenzoyl)piperazin-1-yl)but-2-yn-1-yl)thio)-2-(1-fluorocyclopropane-1-carboxamido)-3-methylbutanoyl)-4-hydroxy-N-(4-(4-methylthiazol-5-yl)benzyl)pyrrolidine-2-carboxamide (**XL01076**)

3.1 mg **XL01076** was obtained starting from 5 mg **10** following general procedure 9 (34% yield). ^{1}H NMR (500 MHz, $CDCl_3$) δ 8.68 (s, 1H), 8.53 (d, *J* = 8.7 Hz, 1H), 7.91 (s, 1H), 7.70 (s, 1H), 7.36 (dd, *J* = 20.1, 8.2 Hz, 4H), 7.25 – 7.17 (m, 2H), 7.03 – 6.97 (m, 2H), 5.36 – 5.27 (m, 1H), 4.91 (d, *J* = 8.0 Hz, 1H), 4.76 – 4.70 (m, 1H), 4.57 – 4.53 (m, 1H), 4.46 (ddd, *J* = 37.5, 15.0, 5.9 Hz, 2H), 4.00 (d, *J* = 11.1 Hz, 1H), 3.92 (s, 3H), 3.85 (dd, *J* = 11.0, 4.3 Hz, 1H), 3.69 (s, 4H), 3.43 – 3.36 (m, 1H), 3.34 – 3.28 (m, 3H), 3.10 (d, *J* = 4.9 Hz, 3H), 2.55 (s, 2H), 2.52 (s, 3H), 2.51 – 2.48 (m, 1H), 2.42 (s, 2H), 2.21 – 2.14 (m, 1H), 1.40 (s, 3H), 1.35 (s, 3H), 1.34 – 1.27 (m, 4H); ^{13}C NMR (126 MHz, $CDCl_3$) δ 170.74, 170.71, 170.52 (d, *J* = 20.7 Hz), 169.92, 162.81, 158.76, 157.85, 152.56, 150.53, 148.63, 147.70, 138.15, 131.75, 131.65, 131.20, 129.72, 128.31, 127.66, 120.38, 117.08, 109.82, 105.73, 81.99, 79.20, 77.99, 70.05, 59.17, 56.62, 56.26, 56.08, 52.20, 48.99, 47.36, 43.38, 36.48, 28.26, 25.64, 25.46, 17.53, 16.17, 14.08 (d, *J* = 11.06 Hz), 13.99 (d, *J* = 12.26 Hz); ^{19}F NMR (471 MHz, $CDCl_3$) δ -197.70; HRMS (ESI$^+$) *m/z*, calcd for $C_{46}H_{54}ClFN_{10}O_6S_2$: 961.3414 [M + H]$^+$, found 961.3672.

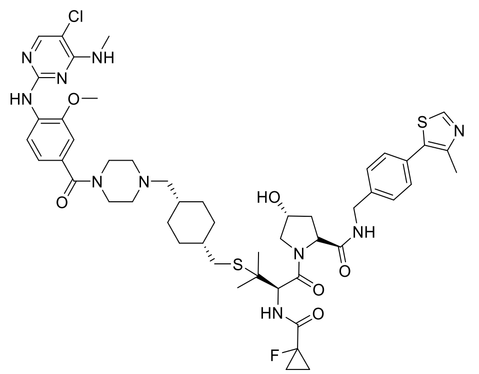

(2*S*,4*R*)-1-((*R*)-3-((((1*S*,4*S*)-4-((4-(4-((5-chloro-4-(methylamino)pyrimidin-2-yl)amino)-3-methoxybenzoyl)piperazin-1-yl)methyl)cyclohexyl)methyl)thio)-2-(1-fluorocyclopropane-1-carboxamido)-3-methylbutanoyl)-4-hydroxy-N-(4-(4-methylthiazol-5-yl)benzyl)pyrrolidine-2-carboxamide (**XL01134**)

9.3 mg **XL01134** was obtained starting from 8.0 mg **10** and 8.5 mg **3-6h** following general procedure 9 (61% yield). ^{1}H NMR (500 MHz, $CDCl_3$) δ 8.67 (s, 1H), 8.54 (d, *J* = 8.6 Hz, 1H), 7.92 (s, 1H), 7.63 (s, 1H), 7.41 – 7.30 (m, 5H), 7.23 (dd, *J* = 7.6, 3.1 Hz, 1H), 7.03 – 6.96 (m, 2H), 5.29 (q, *J* = 4.7 Hz, 1H), 4.79 (t, *J* = 7.9 Hz, 1H), 4.73 (d, *J* = 7.7 Hz, 1H), 4.55 – 4.50 (m, 1H), 4.46 (d, *J* = 5.9 Hz, 2H), 4.06 (d, *J* = 11.1 Hz, 1H), 3.92 (s, 3H), 3.78 – 3.38 (m, 5H), 3.11 (d, *J* = 4.9 Hz, 3H), 2.68 (s, 1H), 2.54 – 2.29 (m, 10H), 2.26 – 2.14 (m, 3H), 1.68 (s, 1H), 1.59 – 1.42 (m, 5H), 1.39 – 1.27 (m, 14H). ^{13}C NMR (126 MHz, $CDCl_3$) δ 170.72, 170.55, 170.20, 158.73, 157.99, 152.82, 150.39, 148.70, 147.57, 138.19, 131.72, 131.46, 131.19, 129.67, 128.18, 120.31, 116.92, 109.75, 105.68, 79.22, 70.30, 62.34, 58.99, 56.64, 56.42, 56.03, 53.91, 47.67, 43.26, 36.76, 35.95, 32.98, 32.12, 28.69, 28.52, 28.22, 27.14, 25.80, 25.49, 16.25, 14.10 (d, *J* = 10.3 Hz), 13.95 (d, *J* = 10.3 Hz). ^{19}F NMR (471 MHz, $CDCl_3$) δ -197.74. HRMS (ESI$^+$) *m/z,* calcd for $C_{50}H_{64}ClFN_{10}O_6S_2$: 1019.4197 [M + H]$^+$, found 1019.4526.

Scheme S4. Synthesis of SD10 and SD11

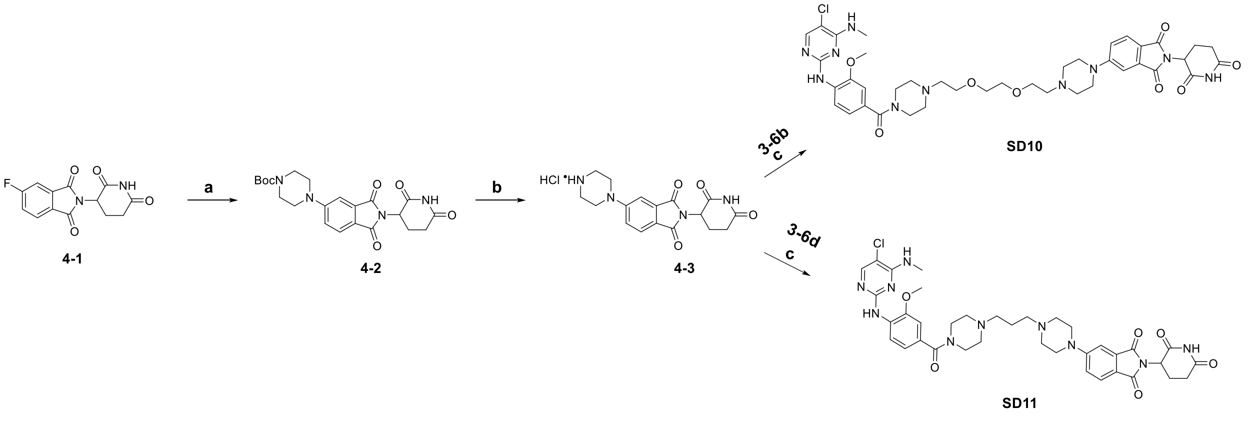

Reagents and conditions: a. Boc-piperidine, DIPEA, NMP, 90 °C; b. 4N HCl in 1,4-dioxane, DCM; c. K_2CO_3, KI, DMF, 100 °C.

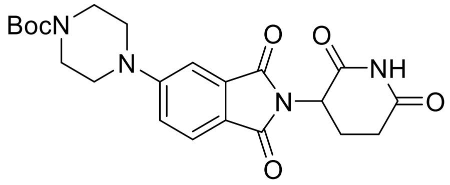

tert-butyl 4-(2-(2,6-dioxopiperidin-3-yl)-1,3-dioxoisoindolin-5-yl)piperazine-1-carboxylate (**4-2**)

To a solution of compound **4-1** (644 mg, 2.33 mmol) and Boc-piperidine (868 mg, 4.66 mmol) in NMP (4.5 ml) was added DIPEA (0.324 ml, 1.86 mmol). After stirring at 90 °C overnight, the mixture was cooled down to room temperature, added with water and extracted with EtOAc (2× 20 ml). The combined organic phase was washed with brine, dried over $MgSO_4$, filtered and condensed under reduced pressure to afford a residue which was purified via flash column chromatography on silica gel (0-50% EtOAc in heptane) to give the title compound (815 mg, 80% yield) as powder. ^{1}H NMR (500 MHz, DMSO-d_6) δ 11.04 (s, 1H), 7.69 (d, *J* = 8.5 Hz, 1H), 7.34 (d, *J* = 2.2 Hz, 1H), 7.24 (dd, *J* = 8.6, 2.3 Hz, 1H), 5.07 (dd, *J* = 12.8, 5.4 Hz, 1H), 3.47 (s, 8H), 2.94 - 2.82 (m, 1H), 2.63 – 2.52 (m, 2H), 2.05 – 1.99 (m, 1H), 1.43 (s, 9H).

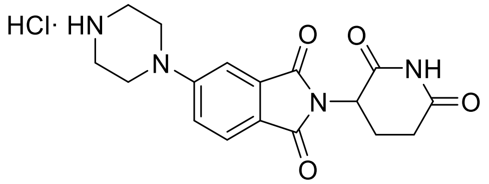

2-(2,6-dioxopiperidin-3-yl)-5-(piperazin-1-yl)isoindoline-1,3-dione (**4-3**)

To a solution of **4-2** (50 mg, 0.11 mmol) in DCM (1 ml) was added with 4N HCl (1 ml). The mixture was stirred at room temperature for 1h and then condensed under reduced pressure to give the title compound as a residue which was used to next step without further purification.

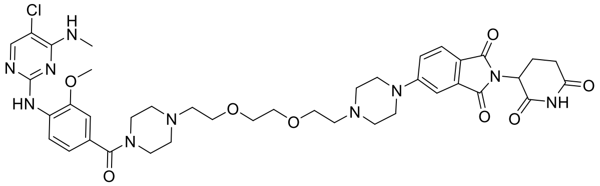

5-(4-(2-(2-(2-(4-(4-((5-chloro-4-(methylamino)pyrimidin-2-yl)amino)-3-methoxybenzoyl)piperazin-1-yl)ethoxy)ethoxy)ethyl)piperazin-1-yl)-2-(2,6-dioxopiperidin-3-yl)isoindoline-1,3-dione (**SD10**)

To a solution of **3-6b** (24.9 mg, 0.0375 mmol) in DMF (0.5 ml) was added **4-3** (14.2 mg , 0.0375 mmol), K_2CO_3 (15 mg, 0.109 mmol), and KI (0.6 mg, 0.00375 mmol). The resulting mixture was heated to 100 °C and stirred at 100 °C overnight. The mixture was filtered through 0.45 μM filter and purified with preparative HPLC under acidic condition (10-85% CH_3CN in 0.1% aq HCO_2H) to give the title compound (2.82 mg, 9% yield). ^{1}H NMR (400 MHz, MeOD-d_4) δ 8.63 – 8.49 (m, 1H), 7.84 (d, *J* = 15.7 Hz, 1H), 7.69 – 7.59 (m, 1H), 7.32 (d, *J* = 31.5 Hz, 1H), 7.19 (dd, *J* = 39.4, 8.5 Hz, 1H), 7.04 (dd, *J* = 18.7, 9.7 Hz, 2H), 5.12 – 5.04 (m, 1H), 3.97 (d, *J* = 4.2 Hz, 3H), 3.75 - 3.65 (m, 5H), 3.64 – 3.56 (m, 6H), 3.55 – 3.49 (m, 2H), 3.42 – 3.35 (m, 5H), 3.21 – 3.10 (m, 2H), 3.04 (d, *J* = 3.4 Hz, 3H), 2.96 – 2.81 (m, 2H), 2.71 – 2.58 (m, 10H). HRMS (ESI$^+$) *m/z*, calcd for $C_{40}H_{49}ClN_{10}O_8$: 833.3496 [M + H]$^+$, found 833.3521.

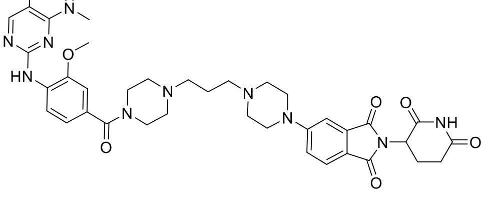

5-(4-(3-(4-(4-((5-chloro-4-(methylamino)pyrimidin-2-yl)amino)-3-methoxybenzoyl)piperazin-1-yl)propyl)piperazin-1-yl)-2-(2,6-dioxopiperidin-3-yl)isoindoline-1,3-dione (**SD11**)

To a solution of **3-6d** (18.7 mg, 0.0375 mmol) and **4-3** (14.2 mg, 0.0375 mmol) in DMF (0.4 ml) was added K_2CO_3 (15 mg, 0.11 mmol) and KI (0.6 mg, 0.00375 mmol). After stirring at 100 °C overnight, the resulting mixture was cooled down and purified with preparative HPLC under acidic condition (10-50 % CH_3CN in 0.1 % aq. HCO_2H) to give the title compound (5.4 mg, 19% yield). ^{1}H NMR (400 MHz, MeOD-d_4) δ 8.60 (d, *J* = 8.2 Hz, 1H), 7.87 (s, 1H), 7.74 (dd, *J* = 14.8, 8.5 Hz, 1H), 7.45 (d, *J* = 21.7 Hz, 1H), 7.32 (dd, *J* = 21.5, 8.5 Hz, 1H), 7.15 – 7.01 (m, 2H), 5.22 – 5.03 (m, 1H), 3.98 (s, 3H), 3.82 – 3.67 (m, 5H),3.63 – 3.57 (m, 3H), 3.42 – 3.37 (m, 1H), 3.36 (s, 3H), 3.06 (s, 3H), 3.01 (s, 2H), 2.95 – 2.82 (m, 3H), 2.79 – 2.69 (m, 6H), 2.17 – 2.08 (m, 1H), 2.00 – 1.77 (m, 2H). HRMS (ESI$^+$) *m/z*, calcd for $C_{37}H_{43}ClN_{10}O_6$: 759.3128 [M + H]$^+$, found 759.3135.

Scheme S5. Synthesis of SD112, SD113, SD114, SD12, SD13, and SD79

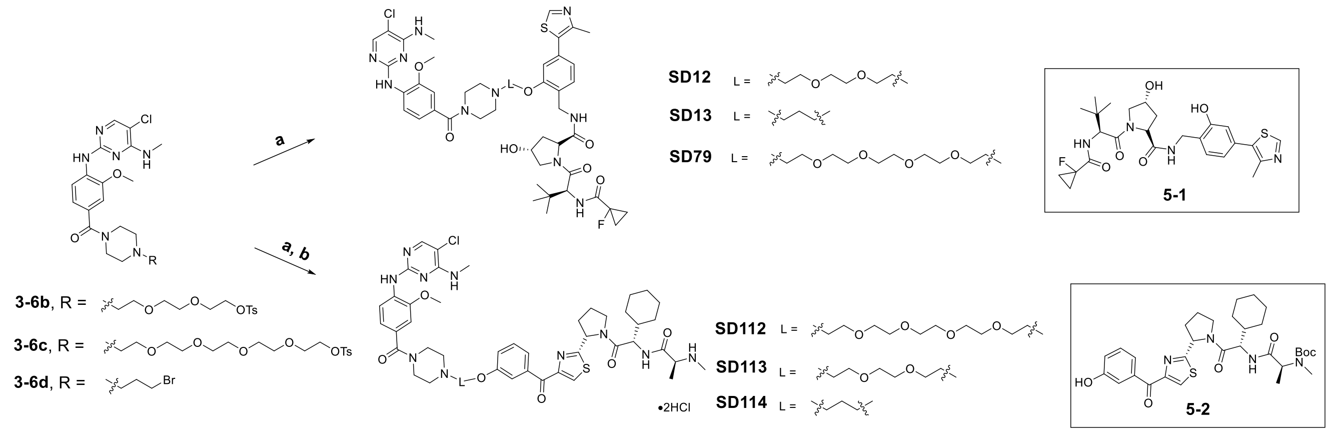

Reagents and conditions: a. **5-1** or **5-2**, K_2CO_3, KI, DMF, 100 °C; b. 4N HCl in 1,4-dioxane, DCM.

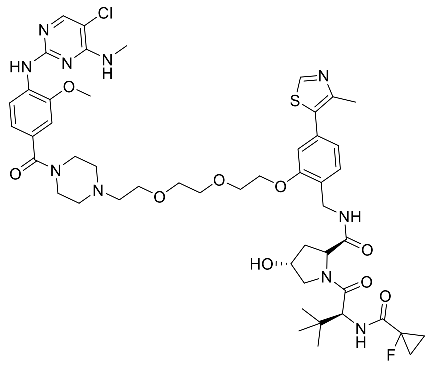

(2*S*,4*R*)-N-(2-(2-(2-(2-(4-(4-((5-chloro-4-(methylamino)pyrimidin-2-yl)amino)-3-methoxybenzoyl)piperazin-1-yl)ethoxy)ethoxy)ethoxy)-4-(4-methylthiazol-5-yl)benzyl)-1-((*S*)-2-(1-fluorocyclopropane-1-carboxamido)-3,3-dimethylbutanoyl)-4-hydroxypyrrolidine-2-carboxamide (**SD12**)

To a solution of **3-6b** (8.29 mg, 0.0125 mmol) and **5-1**[3] (6.65 mg, 0.0125 mmol) in DMF (0.2 ml) was added K_2CO_3 (5 mg, 0.036 mmol) and KI (0.2 mg, 0.0125 mmol). After stirring at 100 °C overnight, the resulting mixture was cooled down, filtered through 0.45 μm PTFE filter, and purified with preparative HPLC under acidic condition (5-95 % CH_3CN in 0.1 % aq. HCO_2H) to give the title compound (3.54 mg, 28% yield). ^{1}H NMR (500 MHz, MeOD-d_4) δ 8.93 (s, 1H), 8.64 (d, *J* = 8.3 Hz, 1H), 7.94 (s, 1H), 7.52 (d, *J* = 7.8 Hz, 1H), 7.12 (d, *J* =

1.4 Hz, 2H), 7.08 (ddd, *J* = 7.7, 4.0, 1.6 Hz, 2H), 4.82 (s, 1H), 4.71 (t, *J* = 8.3 Hz, 1H), 4.57 (dd, *J* = 4.1, 2.1 Hz, 1H), 4.56 – 4.44 (m, 2H), 4.37 – 4.24 (m, 2H), 4.04 (s, 3H), 3.99 (t, *J* = 4.5 Hz, 2H), 3.92 (d, *J* = 11.1 Hz, 1H), 3.88 (d, *J* = 3.7 Hz, 1H), 3.86 – 3.82 (m, 2H), 3.79 – 3.67 (m, 8H), 3.13 (s, 3H), 2.77 (t, *J* = 5.4 Hz, 2H), 2.70 (s, 4H), 2.56 (s, 3H), 2.31 (dd, *J* = 13.1, 7.7 Hz, 1H), 2.17 (ddd, *J* = 13.3, 9.0, 4.5 Hz, 1H), 1.45 – 1.36 (m, 4H), 1.11 (s, 9H). HRMS (ESI$^+$) *m/z*, calcd for $C_{49}H_{64}ClFN_{10}O_9S$:1023.4329 [M + H]$^+$, found 1023.4282.

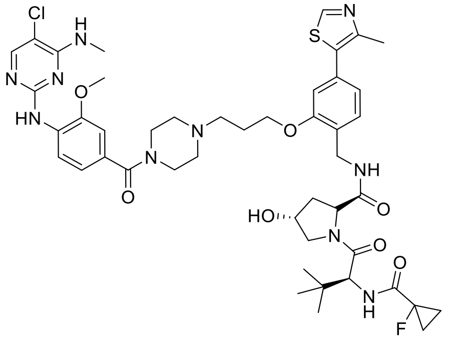

(2*S*,4*R*)-N-(2-(3-(4-(4-((5-chloro-4-(methylamino)pyrimidin-2-yl)amino)-3-methoxybenzoyl)piperazin-1-yl)propoxy)-4-(4-methylthiazol-5-yl)benzyl)-1-((*S*)-2-(1-fluorocyclopropane-1-carboxamido)-3,3-dimethylbutanoyl)-4-hydroxypyrrolidine-2-carboxamide (**SD13**)

To a solution of **3-6d** (7.36 mg, 0.0125 mmol) and **5-1**[3] (6.65 mg, 0.0125 mmol) in DMF (0.2 ml) was added K_2CO_3 (5 mg, 0.036 mmol) and KI (0.2 mg, 0.0125 mmol). After stirring at 100 °C overnight, the resulting mixture was cooled down, filtered through 0.45 μm PTFE filter and purified with preparative HPLC under acidic condition (5-95 % CH_3CN in 0.1 % aq. HCO_2H) to give the title compound (2.95 mg, 25% yield). ^{1}H NMR (500 MHz, MeOD-d_4) δ 8.95 (s, 1H), 8.67 (d, *J* = 8.3 Hz, 1H), 7.95 (s, 1H), 7.55 (d, *J* = 7.7 Hz, 1H), 7.17 – 7.06 (m, 4H), 4.81 (s, 1H), 4.70 (t, *J* = 8.3 Hz, 1H), 4.59 (s, 1H), 4.52 (d, *J* = 2.5 Hz, 2H), 4.25 (t, *J* = 5.9 Hz, 2H), 4.06 (s, 3H), 3.93 (d, *J* = 11.1 Hz, 1H), 3.87 (dd, *J* = 11.0, 3.8 Hz, 1H), 3.83 – 3.66 (m, 4H), 3.14 (s, 3H), 2.79 (t, *J* = 7.3 Hz, 2H), 2.70 (s, 4H), 2.58 (s, 3H), 2.34 – 2.25 (m, 1H), 2.25 – 2.15 (m, 3H), 1.45 – 1.34 (m, 4H), 1.10 (s, 9H). HRMS (ESI$^+$) *m/z*, calcd for $C_{46}H_{58}ClFN_{10}O_7S$:949.3961 [M + H]$^+$, found 949.3944.

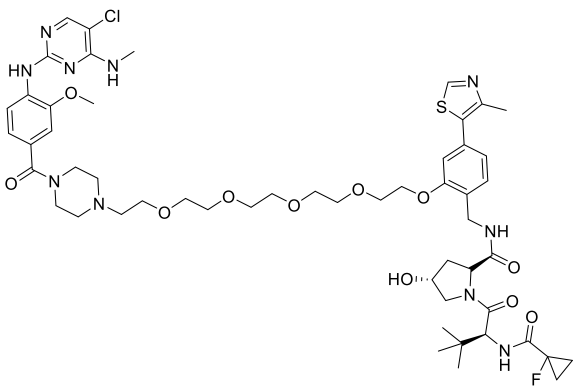

(2*S*,4*R*)-N-(2-((14-(4-(4-((5-chloro-4-(methylamino)pyrimidin-2-yl)amino)-3-methoxybenzoyl)piperazin-1-yl)-3,6,9,12-tetraoxatetradecyl)oxy)-4-(4-methylthiazol-5-yl)benzyl)-1-((*S*)-2-(1-fluorocyclopropane-1-carboxamido)-3,3-dimethylbutanoyl)-4-hydroxypyrrolidine-2-carboxamide (**SD79**)

To a solution of **3-6c** (9.39 mg, 0.0125 mmol) and **5-1** (6.65 mg, 0.0125 mmol) in DMF (0.2 ml) was added K_2CO_3 (5 mg, 0.036 mmol) and KI (0.2 mg, 0.0125 mmol). After stirring at 100 °C overnight, the resulting mixture was cooled down, filtered through 0.45 μm PTFE filter, and purified with preparative HPLC under acidic condition (5-95 % CH_3CN in 0.1 % aq. HCO_2H) to give the title compound (2.24 mg, 16% yield). ^{1}H NMR (500 MHz, MeOD-d_4) δ 8.88 (s, 1H), 8.60 (d, *J* = 8.3 Hz, 1H), 7.86 (s, 1H), 7.48 (d, *J* = 7.6 Hz, 1H), 7.10 – 7.00 (m, 4H), 4.76 (s, 1H), 4.64 (t, *J* = 8.3 Hz, 1H), 4.54 – 4.38 (m, 3H), 4.26 – 4.20 (m, 2H), 3.98 (s, 3H), 3.93 – 3.84 (m, 4H), 3.83 – 3.79 (m, 1H), 3.75

– 3.69 (m, 7H), 3.66 – 3.62 (m, 10H), 3.05 (s, 3H), 2.87 – 2.75 (m, 6H), 2.50 (s, 3H), 2.28 – 2.22 (m, 1H), 2.15 – 2.07 (m, 1H), 1.40 – 1.30 (m, 4H), 1.05 (s, 9H). HRMS (ESI$^+$) *m/z*, calcd for $C_{53}H_{72}ClFN_{10}O_{11}S$: 556.2460 [M + 2H]$^{2+}$, found 556.2469.

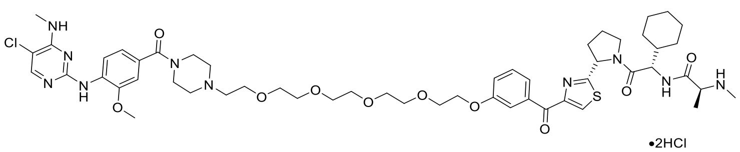

(*S*)-N-((*S*)-2-((*S*)-2-(4-(3-((14-(4-(4-((5-chloro-4-(methylamino)pyrimidin-2-yl)amino)-3-methoxybenzoyl)piperazin-1-yl)-3,6,9,12-tetraoxatetradecyl)oxy)benzoyl)thiazol-2-yl)pyrrolidin-1-yl)-1-cyclohexyl-2-oxoethyl)-2-(methylamino)propenamide HCl salt (**SD112**)

To a solution of **3-6c** (9.4 mg, 0.0125 mmol) in DMF (0.2 ml) was added **5-2**[4] (7.5 mg, 0.0125 mmol), K_2CO_3 (5 mg, 0.036 mmol), and KI (0.6 mg, 0.00375 mmol). The resulting mixture was heated to 100 °C and stirred at 100 °C overnight. The mixture was filtered through 0.45 µM filter and purified with preparative HPLC under acidic condition (10-85% CH_3CN in 0.1% aq HCO_2H) to give the Boc-protected product which was used directly to next step (3.8 mg, 26% yield); HRMS (ESI$^+$) *m/z*, calcd for $C_{58}H_{82}ClN_{10}O_{12}S$ 1177.5517 [M + H]$^+$, found 1177.5747. To a solution of the Boc-protected product (3.8 mg, 0.0028 mmol) in 0.2 ml DCM was added 4N HCl solution in 1,4-dioxane (0.2 ml). The resulting mixture was stirred at room temperature for 2h, then condensed to afford the title compound as HCl salt (3.7 mg, 100% yield). ^{1}H NMR (500 MHz, DMSO- d_6) δ 8.49 (s, 1H), 8.40 (d, *J* = 8.3 Hz, 1H), 8.27 (s, 2H), 8.06 (d, *J* = 8.7 Hz, 1H), 7.96 (s, 1H), 7.70 – 7.60 (m, 3H), 7.46 (t, *J* = 8.0 Hz, 1H), 7.34 (q, *J* = 4.4 Hz, 1H), 7.29 – 7.21 (m, 1H), 7.02 (d, *J* = 1.6 Hz, 1H), 6.97 (dd, *J* = 8.3, 1.6 Hz, 1H), 5.39 (dd, *J* = 8.0, 2.9 Hz, 1H), 4.49 (dd, *J* = 8.5, 7.1 Hz, 1H), 4.18 – 4.13 (m, 2H), 3.89 (s, 3H), 3.82 – 3.78 (m, 2H), 3.77 – 3.74 (m, 2H), 3.60 – 3.58 (m, 2H), 3.55 – 3.52 (m, 2H), 3.52 – 3.46 (m, 15H), 3.12 (q, *J* = 6.8 Hz, 1H), 2.90 (d, *J* = 4.6 Hz, 3H), 2.48 (d, *J* = 5.8 Hz, 1H), 2.42 (s, 4H), 2.30 – 2.24 (m, 1H), 2.22 (s, 3H), 2.20 – 2.15 (m, 1H), 2.07 – 1.99 (m, 2H), 1.74 – 1.61 (m, 4H), 1.55 (d, *J* = 10.2 Hz, 2H), 1.14 (d, *J* = 6.9 Hz, 3H), 1.12 – 0.92 (m, 6H); HRMS (ESI$^+$) *m/z*, calcd for $C_{53}H_{73}ClN_{10}O_{10}S$: 1077.4993 [M + H]$^+$, found 1077.5042.

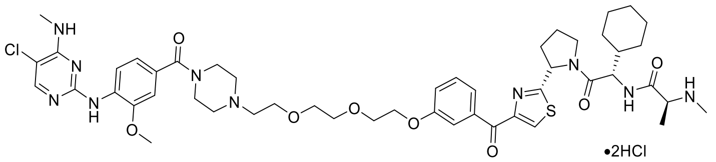

(*S*)-N-((*S*)-2-((*S*)-2-(4-(3-(2-(2-(2-(4-(4-((5-chloro-4-(methylamino)pyrimidin-2-yl)amino)-3-methoxybenzoyl)piperazin-1-yl)ethoxy)ethoxy)ethoxy)benzoyl)thiazol-2-yl)pyrrolidin-1-yl)-1-cyclohexyl-2-oxoethyl)-2-(methylamino)propenamide HCl salt (**SD113**)

To a solution of **3-6b** (8.29 mg, 0.0125 mmol) in DMF (0.2 ml) was added **5-2** (7.5 mg, 0.0125 mmol), K_2CO_3 (5 mg, 0.036 mmol), and KI (0.6 mg, 0.00375 mmol). The resulting mixture was heated to 100 °C and stirred at 100 °C overnight. The mixture was filtered through 0.45 µM filter and purified with preparative HPLC under acidic condition (10-85% CH_3CN in 0.1% aq HCO_2H) to give the Boc-protected compound (3 mg, 22% yield); HRMS (ESI$^+$) *m/z*, calcd for $C_{54}H_{74}ClN_{10}O_{10}S$ 1089.4993 [M + H]$^+$, found 1089.5198. To a solution of the Boc-protected compound (3 mg, 0.0028 mmol) in 0.2 ml DCM was added 4N HCl solution in 1,4-dioxane (0.2 ml). The resulting mixture was stirred at room temperature for 2h, then condensed to afford the title compound as HCl salt (2.9 mg, 100% yield). ^{1}H NMR (500 MHz, DMSO-d_6) δ 8.48 (s, 1H), 8.39 (d, *J* = 8.3 Hz, 1H), 8.25 – 8.19 (m, 3H), 7.96 (s, 1H), 7.69 – 7.60 (m, 3H), 7.45 (t, *J* = 8.0 Hz, 1H), 7.34 (q, *J* = 4.3 Hz, 1H), 7.27 – 7.21 (m, 1H), 7.01 (d, *J* = 1.6 Hz, 1H), 6.96 (dd, *J* = 8.3, 1.6 Hz, 1H), 5.39 (dd, *J* = 8.0, 3.0 Hz, 1H), 4.55 – 4.44 (m, 1H), 4.18 – 4.15 (m, 2H), 3.89 (s, 3H), 3.82 – 3.76 (m, 4H), 3.62 – 3.58 (m, 4H), 3.55 – 3.51 (m, *J* = 7.6, 4.5 Hz, 6H), 3.30 (dd, *J* = 13.7, 6.8 Hz, 1H), 2.90 (d, *J* = 4.6 Hz, 3H), 2.49 (d, *J* = 5.9 Hz, 1H), 2.42 (s, 4H), 2.29 (s, 3H), 2.27 – 2.16 (m, 2H), 2.08 – 1.99 (m, 2H), 1.72 – 1.59

(m, 4H), 1.55 (d, *J* = 9.7 Hz, 2H), 1.19 (d, *J* = 6.9 Hz, 3H), 1.15 – 0.93 (m, 6H). HRMS (ESI$^+$) *m/z,* calcd for $C_{49}H_{65}ClN_{10}O_8S$: 989.4468 [M + H]$^+$, found 989.4537.

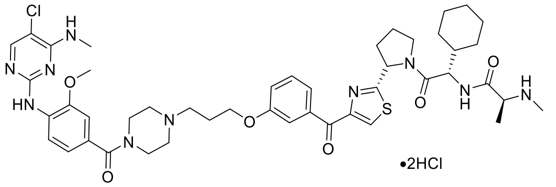

(*S*)-N-((*S*)-2-((*S*)-2-(4-(3-(3-(4-(4-((5-chloro-4-(methylamino)pyrimidin-2-yl)amino)-3-methoxybenzoyl)piperazin-1-yl)propoxy)benzoyl)thiazol-2-yl)pyrrolidin-1-yl)-1-cyclohexyl-2-oxoethyl)-2-(methylamino)propenamide HCl salt (**SD114**)

To a solution of **3-6d** (7.36 mg, 0.0125 mmol) in DMF (0.2 ml) was added **5-2** (7.5 mg, 0.0125 mmol), K_2CO_3 (5 mg, 0.036 mmol), and KI (0.6 mg, 0.00375 mmol). The resulting mixture was heated to 100 °C and stirred at 100 °C overnight. The mixture was filtered through 0.45 µM filter and purified with preparative HPLC under acidic condition (10-85% CH_3CN in 0.1% aq HCO_2H) to give Boc protected **SD114** (4 mg, 32% yield); HRMS (ESI$^+$) *m/z,* calcd for $C_{51}H_{67}ClN_{10}O_8S$ 1015.462534 [M + H]$^+$, found 1015.4770. To a solution of Boc protected SD114 (4 mg, 0.0039 mmol) in 0.2 ml DCM was added 4N HCl solution in 1,4-dioxane (0.2 ml). The resulting mixture was stirred at room temperature for 2h, then condensed to afford the title compound as HCl salt (3.9 mg, 100% yield). ^{1}H NMR (500 MHz, DMSO-d_6) δ 8.50 (s, 1H), 8.41 (d, *J* = 8.3 Hz, 1H), 8.06 (br, s, 1H), 7.97 (s, 1H), 7.73 – 7.63 (m, 3H), 7.51 – 7.41 (m, 1H), 7.35 (q, *J* = 4.3 Hz, 1H), 7.27 – 7.20 (m, 1H), 7.03 (d, *J* = 1.5 Hz, 1H), 6.98 (dd, *J* = 8.3, 1.6 Hz, 1H), 5.40 (dd, *J* = 7.9, 2.7 Hz, 1H), 4.52 – 4.44 (m, 1H), 4.10 (t, *J* = 6.2 Hz, 2H), 3.90 (s, 3H), 3.85 – 3.77 (m, 4H), 3.52 – 3.47 (m, 4H), 2.91 (d, *J* = 4.6 Hz, 3H), 2.48 (d, *J* = 7.2 Hz, 1H), 2.46 – 2.35 (m, 4H), 2.31 – 2.15 (m, 4H), 2.14 – 2.08 (m, 1H), 2.08 – 2.00 (m, 2H), 1.98 – 1.88 (m, 2H), 1.72 – 1.56 (m, 4H), 1.56 – 1.49 (m, 2H), 1.19 – 0.86 (m, 9H). HRMS (ESI$^+$) *m/z,* calcd for $C_{46}H_{59}ClN_{10}O_6S$: 915.4101 [M + H]$^+$, found 915.4190.

Scheme S6. Synthesis of XL01140

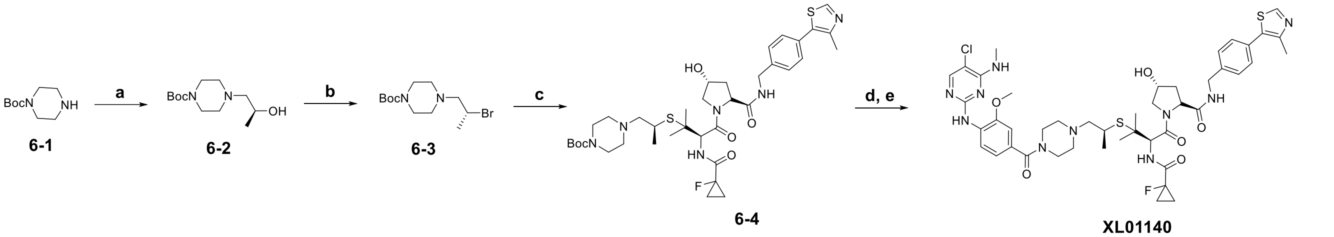

Reagents and Conditions: a.(*S*)-Propylene oxide, ethanol; b. CBr_4, PPh_3, DCM; c. **10**, DBU, THF; d. 4N HCl in 1,4-dioaxne, DCM; e. **7**, PyOxim, DIPEA, DMF.

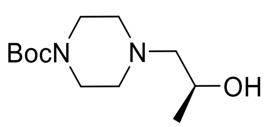

tert-butyl (*S*)-4-(2-hydroxypropyl)piperazine-1-carboxylate (**6-2**)

A mixture of tert-butyl piperazine-1-carboxylate (500 mg, 2.69 mmol), (*S*)-propylene oxide (234 mg, 4.0 mmol), and ethanol (3 ml) was stirred in a sealed microwave tube at room temperature overnight. The mixture was condensed under reduced pressure to afford a residue which was purified with flash column chromatography on silica gel (0%-10% methanol in DCM) to give the title compound (390 mg, 60% yield). ^{1}H NMR (400 MHz, $CDCl_3$) δ 3.93 – 3.73 (m, 1H), 3.48 – 3.34 (m, 4H), 3.30 (s, 1H), 2.66 – 2.53 (m, 2H), 2.36 – 2.17 (m, 4H), 1.43

(s, 9H), 1.11 (d, *J* = 6.1 Hz, 3H). ^{13}C NMR (101 MHz, $CDCl_3$) δ 154.80, 79.80, 65.91, 62.45, 53.17, 43.81, 28.53, 20.09. LC-MS, ESI$^+$, m/z 245.1 [M+H]$^+$.

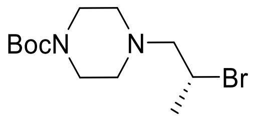

tert-butyl (R)-4-(2-bromopropyl)piperazine-1-carboxylate (**6-3**)

To a solution of **6-2** (150 mg, 0.61 mmol) and CBr_4 (367 mg, 1.1 mmol) in DCM (6 ml) was added triphenyl phosphine (298 mg, 1.1 mmol) at 0 °C. After stirring at 0 °C for 1h, NH_4Cl aqueous was added and the mixture was extracted with DCM (3×). The combined organic phase was washed with water and brine separately, dried over sodium sulfate, filtered, and condensed to afford a residue which was purified through flash column chromatography on silica gel (0%-50% ethyl acetate in heptane) to give the title compound (64 mg, 34% yield). ^1H NMR (500 MHz, $CDCl_3$) δ 4.21 – 4.04 (m, 1H), 3.42 (t, *J* = 5.0 Hz, 4H), 2.75 (dd, *J* = 13.2, 6.6 Hz, 1H), 2.54 (dd, *J* = 13.2, 7.5 Hz, 1H), 2.48 – 2.37 (m, 4H), 1.71 (d, *J* = 6.6 Hz, 3H), 1.45 (s, 9H). ^{13}C NMR (126 MHz, $CDCl_3$) δ 154.88, 79.78, 67.00, 53.38, 46.62, 43.89, 28.58, 24.24. LC-MS, ESI$^+$, m/z 306.9 [M+H]$^+$, 308.8 [M+H+2]$^+$.

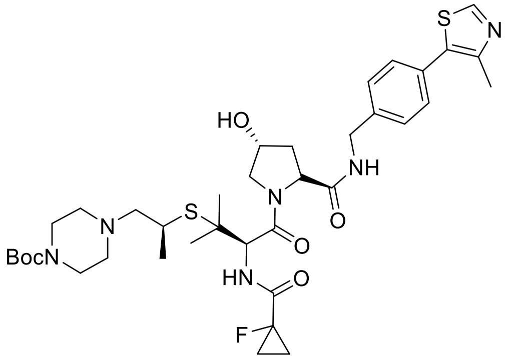

tert-butyl 4-((*S*)-2-(((*R*)-3-(1-fluorocyclopropane-1-carboxamido)-4-((2*S*,4*R*)-4-hydroxy-2-((4-(4-methylthiazol-5-yl)benzyl)carbamoyl)pyrrolidin-1-yl)-2-methyl-4-oxobutan-2-yl)thio)propyl)piperazine-1-carboxylate (**6-4**)

16 mg **6-4** was obtained from 20 mg **10** following general procedure 9 (57% yield). ^1H NMR (500 MHz, $CDCl_3$) δ 8.67 (s, 1H), 7.40 – 7.32 (m, 4H), 7.30 – 7.26 (m, 1H), 4.75 (t, *J* = 7.9 Hz, 1H), 4.70 (d, *J* = 7.5 Hz, 1H), 4.53 (s, 1H), 4.45 (qd, *J* = 15.1, 6.0 Hz, 2H), 4.05 (d, *J* = 11.1 Hz, 1H), 3.70 (dd, *J* = 11.0, 3.6 Hz, 1H), 3.44 – 3.41 (m, 1H), 3.37 (s, 4H), 2.91 (s, 1H), 2.76 – 2.65 (m, 2H), 2.51 (s, 3H), 2.49 – 2.46 (m, 1H), 2.46 – 2.41 (m, 3H), 2.41 – 2.35 (m, 2H), 2.23 – 2.16 (m, 1H), 1.44 (s, 9H), 1.36 – 1.28 (m, 10H), 1.01 (d, *J* = 5.8 Hz, 3H); ^{13}C NMR (126 MHz, $CDCl_3$) δ 170.76, 170.55 (d, *J* = 20.1 Hz), 170.10, 154.86, 150.40, 148.70, 138.16, 131.68, 131.21, 129.68, 128.14, 79.73, 79.20, 70.33, 67.00, 59.45, 59.09, 56.73, 56.26, 53.38, 48.26, 43.27, 36.77, 32.17, 28.59, 25.98, 25.24, 16.23, 14.53, 14.03 (d, *J* = 10.4 Hz), 13.91 (d, *J* = 10.4 Hz). LC-MS, ESI$^+$, m/z 761.9 [M+H]$^+$.

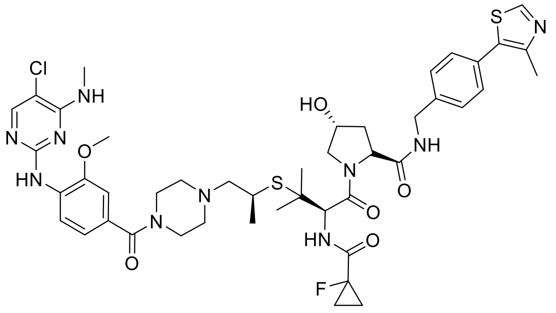

(2*S*,4*R*)-1-((*R*)-3-(((*S*)-1-(4-(4-((5-chloro-4-(methylamino)pyrimidin-2-yl)amino)-3-methoxybenzoyl)piperazin-1-yl)propan-2-yl)thio)-2-(1-fluorocyclopropane-1-carboxamido)-3-methylbutanoyl)-4-hydroxy-N-(4-(4-methylthiazol-5-yl)benzyl)pyrrolidine-2-carboxamide (**XL01140**)

To a stirring solution of **6-4** (16 mg, 0.021 mmol) in DCM (1 mL) was added HCl (4N in 1,4-dioxane, 1 mL). After stirring at room temperature overnight, the mixture was concentrated to afford a solid which was washed with ethyl ether and used to next step. The solid was dissolved in DMF (1ml), 4-((5-chloro-4-(methylamino)pyrimidin-2-yl)amino)-3-methoxybenzoic acid (**7**, 7 mg, 0.023 mmol), DIPEA (16 μL, 0.084 mmol), and PyOxim (12 mg, 0.023 mmol) was added separately. After stirring at room temperature for 2h, the reaction mixture was diluted with DCM (5ml), washed with water and brine, dried over sodium sulfate, filtered, and condensed to afford a residue which was purified via preparative HPLC under acidic condition (5-95 % CH_3CN in 0.1 % aq. HCO_2H) to give the title compound (3.6 mg, 17% yield). ^{1}H NMR (500 MHz, $CDCl_3$) δ 8.67 (s, 1H), 8.54 (d, *J* = 8.2 Hz, 1H), 7.92 (s, 1H), 7.63 (s, 1H), 7.40 – 7.32 (m, 4H), 7.29 – 7.26 (m, 1H), 7.21 (t, *J* = 5.9 Hz, 1H), 7.02 – 6.96 (m, 2H), 5.33 – 5.25 (m, 1H), 4.75 (t, *J* = 8.0 Hz, 1H), 4.70 (d, *J* = 7.6 Hz, 1H), 4.55 (s, 1H), 4.46 (ddd, *J* = 38.2, 15.1, 5.9 Hz, 2H), 4.07 (d, *J* = 11.3 Hz, 1H), 3.92 (s, 3H), 3.73 – 3.54 (m, 5H), 3.11 (d, *J* = 4.9 Hz, 3H), 2.81 – 2.73 (m, 1H), 2.73 – 2.67 (m, 1H), 2.64 (s, 1H), 2.54 – 2.52 (m, 1H), 2.51 (s, 3H), 2.51 – 2.44 (m, 4H), 2.25 – 2.16 (m, 1H), 1.34 (d, *J* = 1.3 Hz, 6H), 1.33 – 1.27 (m, 4H), 1.03 (d, *J* = 6.4 Hz, 3H); ^{13}C NMR (126 MHz, $CDCl_3$) δ 170.56, 170.50, 170.40, 170.00, 158.57, 157.81, 152.66, 150.26, 148.57, 147.42, 137.95, 131.48, 131.38, 131.12, 129.55, 128.01, 120.22, 116.73, 109.66, 105.56, 79.08, 70.21, 59.27, 58.83, 56.54, 56.15, 55.88, 48.28, 47.37, 43.17, 36.44, 32.06, 28.07, 25.91, 25.10, 16.09, 14.34, 13.96 (d, *J* = 10.4 Hz), 13.76 (d, *J* = 10.2 Hz). LC-MS, ESI$^-$, m/z 949 [M-H]$^-$. HRMS (ESI$^+$) *m/z,* calcd for $C_{45}H_{56}ClFN_{10}O_6S_2$: 951.3571 [M + H]$^+$, found 951.3586.

Scheme S7. Synthesis of XL01145

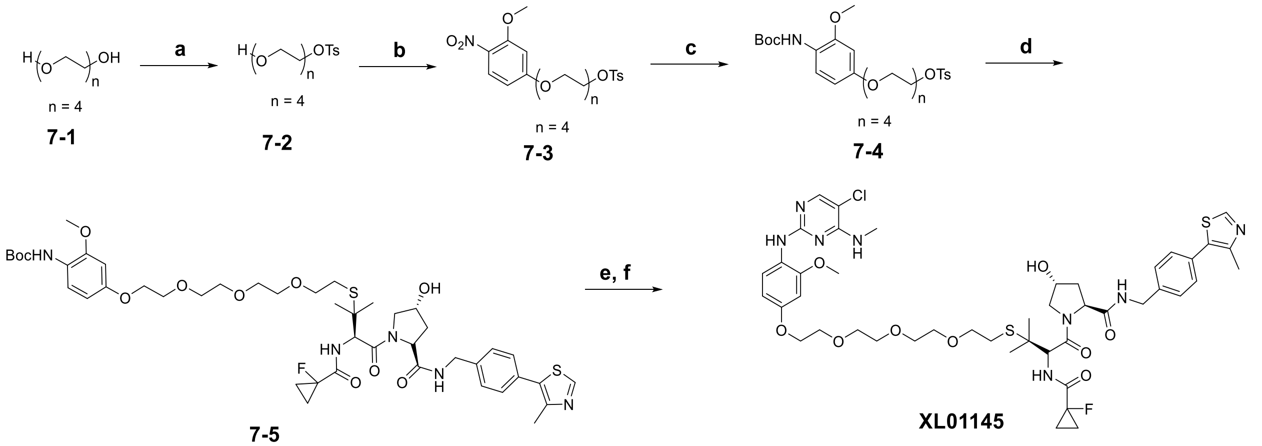

Reagents and conditions: a. TsCl, DMAP, TEA, DCM; b. 3-methoxy-4-nitrophenol, DIAD, PPh_3, THF; c. i) Pd/C, H_2, Ethyl acetate; ii) Boc_2O, TEA, DCM; d. **10**, DBU, THF; e. 4 N HCl in 1,4-dioxane, DCM; f. 2,5-dichloro-N-methylpyrimidin-4-amine, HCl, i-PrOH, MW, 100 °C.

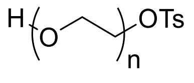

n = 4

2-(2-(2-(2-hydroxyethoxy)ethoxy)ethoxy)ethyl 4-methylbenzenesulfonate (**7-2**)

To a solution of tetraethylene glycol (6.1 g, 31.5 mmol) in DCM (100 mL) was added TEA (2.92 mL, 20.98 mmol), DMAP (64 mg, 0.52 mmol), and TsCl (2g, 10.5 mmol) at 0 °C. After stirring at room temperature overnight, the mixture was diluted with DCM, washed with water and brine, dried over sodium sulfate, filtered, condensed to afford a residue which was purified through flash column chromatography on silica gel (0%-100% ethyl acetate in heptane) to give the title compound (2.4 g, 66% yield) as white solid. ^{1}H NMR (400 MHz, $CDCl_3$) δ 7.79 (d, *J* = 8.0 Hz, 2H), 7.33 (d, *J* = 8.0 Hz, 2H), 4.18 -4.14 (m, 2H), 3.74 – 3.53 (m, 14H), 2.44 (s, 3H).

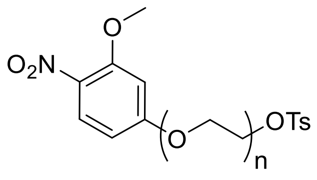

n = 4

2-(2-(2-(2-(3-methoxy-4-nitrophenoxy)ethoxy)ethoxy)ethoxy)ethyl 4-methylbenzenesulfonate (**7-3**)

Triphenyl phosphine (275 mg, 1.05 mmol) was added to a solution of 3-methoxy-4-nitrophenol (111 mg, 0.658 mmol), **7-2** (229 mg, 0.658 mmol), and Diisopropyl azodicarboxylate (199 mg, 0.987 mmol) in THF (5 ml) under nitrogen protection. After stirring at room temperature overnight, the mixture was diluted with ethyl acetate, washed with $NaOH_{aq}$, 1N HCl_{aq} and brine separately, dried over sodium sulfate, filtered, and condensed under reduced pressure to give a residue which was purified through flash column chromatography on silica gel (0%-100% ethyl acetate in heptane) to afford the title compound (85 mg, 24% yield). ^{1}H NMR (400 MHz, $CDCl_3$) δ 7.97 (d, *J* = 9.1 Hz, 1H), 7.83 – 7.73 (m, 2H), 7.33 (d, *J* = 8.0 Hz, 2H), 6.59 (d, *J* = 2.5 Hz, 1H), 6.51 (dd, *J* = 9.1, 2.5 Hz, 1H), 4.21 (dd, *J* = 5.3, 4.1 Hz, 2H), 4.15 (dd, *J* = 5.4, 4.3 Hz, 2H), 3.93 (s, 3H), 3.88 (dd, *J* = 5.3, 4.1 Hz, 2H), 3.74 – 3.64 (m, 6H), 3.63 – 3.56 (m, 4H), 2.44 (s, 3H). ^{13}C NMR (101 MHz, $CDCl_3$) δ 164.21, 155.80, 144.97, 133.25, 129.98, 128.51, 128.10, 105.49, 100.57, 71.03, 70.95, 70.88, 70.73, 69.61, 69.37, 68.89, 68.35, 56.66, 21.76. LC-MS, ESI$^+$, m/z 500 $[M+H]^+$.

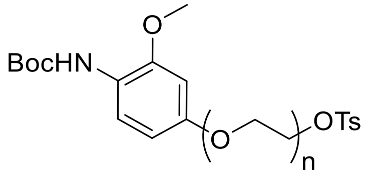

n = 4

2-(2-(2-(2-(4-((tert-butoxycarbonyl)amino)-3-methoxyphenoxy)ethoxy)ethoxy)ethoxy)ethyl 4-methylbenzenesulfonate (**7-4**)

To a solution of **7-3** (85 mg, 0.17 mmol) in ethyl acetate (3 ml) was added 10% palladium charcoal (10 mg) under nitrogen protection. The nitrogen was then replaced with hydrogen and the resulting mixture was stirred under hydrogen pressure (hydrogen balloon) overnight. The mixture was filtered through a pad of celite and concentrated to afford a residue which was dissolved in DCM, followed by addition of TEA (0.047 ml, 0.46 mmol) and Di-tert-butyl decarbonate (37 mg, 0.17 mmol). After stirring at room temperature for 2h, the resulting mixture was concentrated and purified via flash column chromatography on silica gel (0%-100% ethyl acetate in heptane) to give the title compound (58 mg, 60% yield for two steps). ^{1}H NMR (400 MHz, $CDCl_3$) δ 7.88 (d, *J* = 7.8 Hz, 1H), 7.83 – 7.71 (m, 2H), 7.32 (d, *J* = 8.0 Hz, 2H), 6.81 (s, 1H), 6.50 (d, *J* = 2.6 Hz, 1H), 6.44 (dd, *J* = 8.8, 2.6 Hz, 1H), 4.17 – 4.12 (m, 2H), 4.11 – 4.02 (m, 2H), 3.86 – 3.77 (m, 5H), 3.71 – 3.66 (m, 4H), 3.65 – 3.61 (m, 2H), 3.60 – 3.55 (m, 4H), 2.43 (s, 3H), 1.51 (s, 9H). ^{13}C NMR (101 MHz, $CDCl_3$) δ 154.81, 153.12, 148.97,

144.87, 133.26, 129.93, 128.08, 121.94, 119.07, 104.95, 99.64, 80.13, 70.89, 70.81, 70.70, 69.94, 69.35, 68.83, 67.94, 55.79, 28.51, 21.72. LC-MS, ESI$^+$, 470.15. [M-100+H]$^+$.

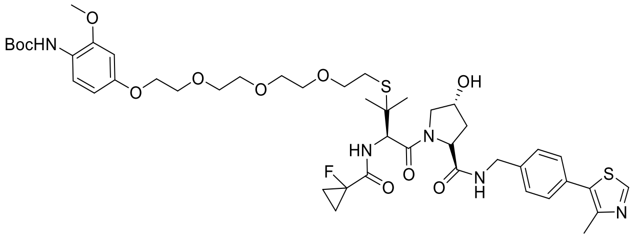

tert-butyl (4-(((*R*)-1-(1-fluorocyclopropyl)-3-((2*S*,4*R*)-4-hydroxy-2-((4-(4-methylthiazol-5-yl)benzyl)carbamoyl)pyrrolidine-1-carbonyl)-4,4-dimethyl-1-oxo-8,11,14-trioxa-5-thia-2-azahexadecan-16-yl)oxy)-2-methoxyphenyl)carbamate (**7-5**)

To a solution of **7-4** (29 mg, 0.05 mmol) and **10** (27 mg, 0.05 mmol) in THF (2 ml) was added 1,8-Diazabicyclo[5.4.0]undec-7-ene (DBU) (0.072 mL, 0.5 mmol). After stirring at room temperature overnight, the mixture was concentrated and purified through flash column chromatography on silica gel (0%-10% methanol in DCM) to yield the title compound (36 mg, 77% yield). ^{1}H NMR (500 MHz, $CDCl_3$) δ 8.59 (s, 1H), 7.81 (s, 1H), 7.34 – 7.23 (m, 5H), 7.14 (dd, *J* = 8.1, 3.2 Hz, 1H), 6.75 (s, 1H), 6.42 (d, *J* = 2.6 Hz, 1H), 6.38 (dd, *J* = 8.8, 2.6 Hz, 1H), 4.74 (d, *J* = 8.0 Hz, 1H), 4.68 (t, *J* = 7.8 Hz, 1H), 4.47 – 4.30 (m, 3H), 4.06 – 3.98 (m, 2H), 3.87 (d, *J* = 11.2 Hz, 1H), 3.80 – 3.71 (m, 6H), 3.65 – 3.59 (m, 2H), 3.57 – 3.55 (m, 2H), 3.54 – 3.41 (m, 6H), 3.14 (s, 1H), 2.80 – 2.56 (m, 2H), 2.44 (s, 3H), 2.33 (ddd, *J* = 7.2, 6.0, 3.3 Hz, 1H), 2.19 – 2.05 (m, 1H), 1.43 (s, 9H), 1.29 – 1.19 (m, 10H); ^{13}C NMR (126 MHz, $CDCl_3$) δ 170.81, 170.22 (d, *J* = 20.68 Hz), 169.81, 154.67, 153.04, 150.24, 148.91, 148.49, 138.13, 131.60, 130.95, 129.46, 128.13, 121.82, 119.06, 104.83, 99.53, 80.05, 78.12 (d, *J* = 232.46 Hz), 70.78, 70.59, 70.53, 70.40, 70.24, 69.89, 69.82, 67.80, 59.15, 56.43, 56.00, 55.68, 47.96, 43.03, 36.81, 28.45, 28.39, 25.75, 25.20, 16.07, 13.83 (d, *J* = 10.6 Hz), 13.81 (d, *J* = 10.5 Hz). LC-MS, ESI$^+$, m/z 933 [M+H]$^+$.

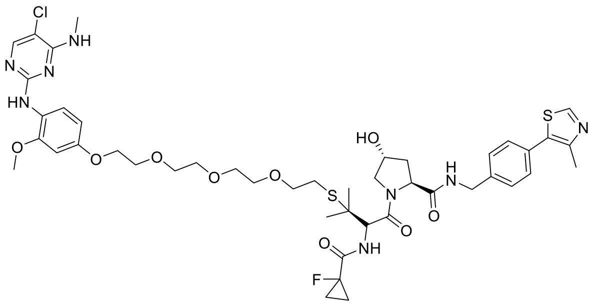

(2*S*,4*R*)-1-((*S*)-1-(4-((5-chloro-4-(methylamino)pyrimidin-2-yl)amino)-3-methoxyphenoxy)-14-(1-fluorocyclopropane-1-carboxamido)-13,13-dimethyl-3,6,9-trioxa-12-thiapentadecan-15-oyl)-4-hydroxy-N-(4-(4-methylthiazol-5-yl)benzyl)pyrrolidine-2-carboxamide (**XL01145**)

To a solution of **7-5** (36 mg, 0.039 mmol) in DCM (1 mL) was added HCl (4N in 1,4-dioxane, 1 mL). After stirring at room temperature overnight, the mixture was condensed and dissolved in isopropanol (1.5 ml). 2,5-dichloro-N-methylpyrimidin-4-amine (8 mg, 0.045 mmol) and HCl (4N in 1,4-dioxane, one drop) were added and the resulting mixture was heated in microwave at 100 °C for 8h. Removal of the solvent under reduced pressure give a residue which was purified through preparation HPLC under acidic condition (5-95 % CH_3CN in 0.1 % aq. HCO_2H) to give the title compound (8.3 mg, 22% yield for two steps). ^{1}H NMR (400 MHz, $CDCl_3$) δ 8.67 (s, 1H), 8.25 (d, *J* = 8.8 Hz, 1H), 7.85 (s, 1H), 7.41 – 7.29 (m, 6H), 7.19 (dd, *J* = 8.0, 3.2 Hz, 1H), 6.54 (d, *J* = 2.6 Hz, 1H), 6.49 (dd, *J* = 8.9, 2.7 Hz, 1H), 5.27 – 5.20 (m, 1H), 4.81 (d, *J* = 7.8 Hz, 1H), 4.78 – 4.73 (m, 1H), 4.55 – 4.50 (m, 1H), 4.50 – 4.38 (m, 2H), 4.14 – 4.08 (m, 2H), 3.95 (d, *J* = 11.2 Hz, 1H), 3.87 – 3.78 (m, 6H), 3.74 – 3.67 (m, 2H), 3.67 – 3.62 (m, 2H), 3.62 – 3.46 (m, 6H), 3.07 (d, *J* = 4.9 Hz, 3H), 2.83 – 2.67 (m, 2H), 2.51 (s,

3H), 2.48 – 2.40 (m, 1H), 2.26 – 2.14 (m, 1H), 1.38 – 1.23 (m, 10H); ^{13}C NMR (101 MHz, $CDCl_3$) δ 170.83, 170.43 (d, *J* = 20.6 Hz), 170.06, 158.69, 158.37, 154.30, 152.65, 150.41, 149.52, 148.65, 138.26, 131.76, 131.14, 129.64, 128.33, 123.53, 119.70, 104.83, 104.62, 99.76, 78.40 (d, *J* = 209.1 Hz), 70.97, 70.77, 70.70, 70.58, 70.35, 70.03, 68.03, 59.19, 56.46, 56.17, 55.92, 47.94, 43.23, 36.80, 28.64, 28.13, 25.96, 25.27, 16.22, 14.05 (d, *J* = 9.6 Hz), 13.95 (d, *J* = 9.5 Hz); HRMS (ESI$^+$) *m/z,* calcd for $C_{45}H_{58}ClFN_8O_9S_2$:973.3513 [M + H]$^+$, found 973.3903.

Scheme S8. Synthesis of XL01149

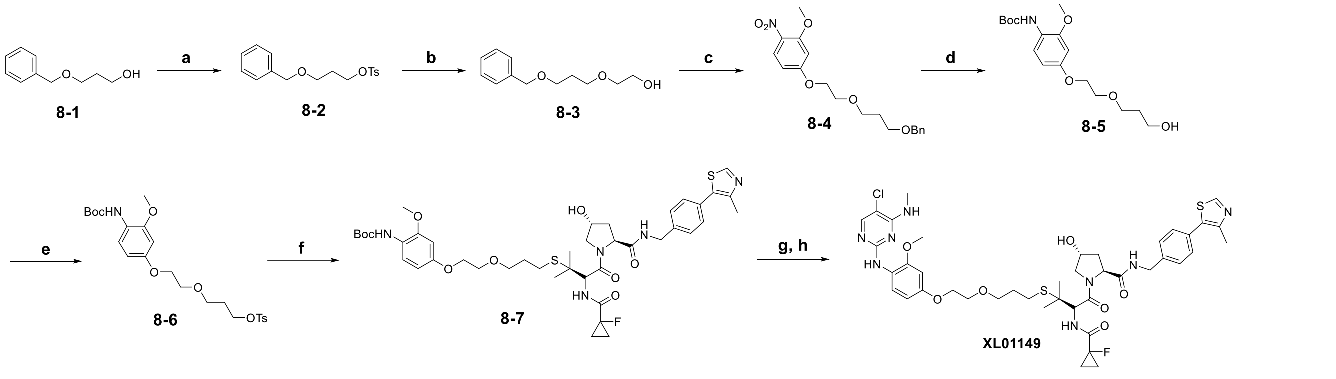

Reagents and conditions: a. TsCl, DMAP, TEA, DCM; b. ethylene glycol, NaH, 60 °C, overnight; c. 3-methoxy-4-nitrophenol, DIAD, PPh_3, THF; d. i) Pd/C, H_2, Methanol; ii) Boc_2O, TEA, DCM; e. TsCl, DMAP, TEA; f. **10**, DBU, THF; g. 4N HCl in 1,4-dioane; h. 2,5-dichloro-N-methylpyrimidin-4-amine, 4N HCl, i-PrOH, MW, 100 °C.

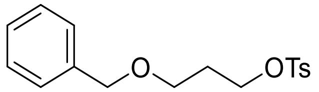

3-(benzyloxy)propyl 4-methylbenzenesulfonate (**8-2**)

TsCl (867 mg, 4.54 mmol) was added to a solution of 3-(benzyloxy)propan-1-ol (720 mg, 4.33 mmol), TEA (0.904 mL, 6.5 mmol), and DMAP (26 mg, 0.21 mmol) in DCM (10 mL) at 0 °C. After stirring at room temperature overnight, the mixture was diluted with DCM (20 mL), washed with water and brine, dried over sodium sulfate, filtered, and condensed under reduced pressure to give a residue which was purified through flash column chromatography on silica gel (0%-50% ethyl acetate in heptane) to yield the title compound (750 mg, 54% yield). ^{1}H NMR (400 MHz, $CDCl_3$) δ 7.85 – 7.77 (m, 2H), 7.39 – 7.24 (m, 7H), 4.43 (s, 2H), 4.20 (t, *J* = 6.1 Hz, 2H), 3.53 (t, *J* = 6.1 Hz, 2H), 2.45 (s, 3H), 1.97 (p, *J* = 6.1 Hz, 2H).

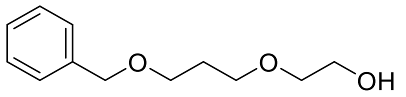

2-(3-(benzyloxy)propoxy)ethan-1-ol (**8-3**)

NaH (60% in mineral oil, 206 mg, 5.16 mmol) was added to a solution of ethylene glycol (1.453 g, 23.4 mmol) and **8-2** (1.5 g, 4.69 mmol) in THF (20 mL) at 0 °C. The resulting mixture was heated to reflux and stirred at reflux overnight. The reaction mixture was then cooled down in ice bath and NH_4Cl_{aq} was added dropwise to quench the reaction. The resulting mixture was extracted with ethyl acetate (3× 30 mL), the combined organic phase was washed with water and brine, dried over sodium sulfate, filtered, and condensed to afford a residue which was purified through flash column chromatography on silica gel (0%-10% methanol in DCM) to give the title compound (534 mg, 54% yield). ^{1}H NMR (400 MHz, $CDCl_3$) δ 7.43 – 7.18 (m, 5H), 4.53 (s, 2H), 3.75 – 3.69 (m, 2H), 3.68 – 3.49 (m, 6H), 2.26 (t, *J* = 5.8 Hz, 1H), 1.93 (p, *J* =

6.3 Hz, 2H). ^{13}C NMR (101 MHz, $CDCl_3$) δ 138.57, 128.46, 127.74, 127.67, 73.08, 71.99, 68.34, 67.32, 61.90, 30.12. LC-MS, ESI$^+$, m/z 233 $[M+Na]^+$.

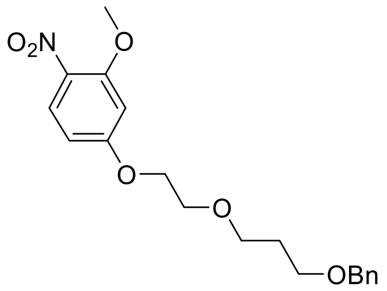

4-(2-(3-(benzyloxy)propoxy)ethoxy)-2-methoxy-1-nitrobenzene (**8-4**)

To a solution of 3-methoxy-4-nitrophenol (81 mg, 0.48 mmol), 2-(3-(benzyloxy)propoxy)ethan-1-ol (100 mg, 0.48 mmol), and triphenyl phosphine (312 mg, 1.19 mmol) in THF was added Diisopropyl azodicarboxylate (192 mg, 0.95 mmol) at 0 °C. The resulting reaction mixture was stirred at room temperature with nitrogen protection overnight. Ethyl acetate was added. The resulting mixture was washed with 1 N $NaOH_{aq}$, water, and brine separately, then dried over sodium sulfate, filtered, and condensed to give a residue which was purified through flash column chromatography on silica gel (0%-100% ethyl acetate in heptane) to afford the title compound (80 mg, 46% yield). ^{1}H NMR (400 MHz, $CDCl_3$) δ 7.97 (d, *J* = 9.1 Hz, 1H), 7.40 – 7.23 (m, 5H), 6.58 (d, *J* = 2.5 Hz, 1H), 6.50 (dd, *J* = 9.1, 2.5 Hz, 1H), 4.49 (s, 2H), 4.17 (dd, *J* = 5.3, 4.1 Hz, 2H), 3.91 (s, 3H), 3.80 (dd, *J* = 5.3, 4.1 Hz, 2H), 3.65 (t, *J* = 6.3 Hz, 2H), 3.57 (t, *J* = 6.3 Hz, 2H), 1.92 (p, *J* = 6.3 Hz, 2H). ^{13}C NMR (101 MHz, $CDCl_3$) δ 164.21, 155.74, 138.64, 128.48, 127.68, 105.37, 100.60, 73.11, 69.12, 68.71, 68.28, 67.19, 56.59, 30.18. LC-MS, ESI$^+$, m/z 361.9 $[M+H]^+$.

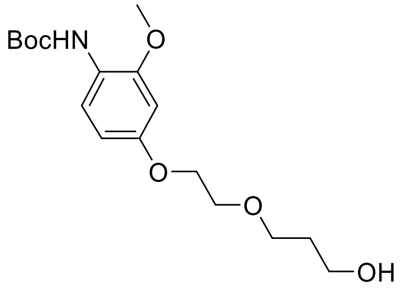

tert-butyl (4-(2-(3-hydroxypropoxy)ethoxy)-2-methoxyphenyl)carbamate (**8-5**)

To a solution of **8-4** (80 mg, 0.22 mmol) in methanol (3 mL) was added 10% Palladium charcoal (8 mg) under nitrogen protection. The nitrogen was evacuated and backfilled with hydrogen and the resulting mixture was stirred under positive hydrogen pressure (hydrogen balloon) overnight. The mixture was then filtered through a pad of celite, and the filtrate was condensed to afford a residue which was dissolved in DCM (3 ml), followed by addition of TEA (0.092 mL, 0.66 mmol) and Di-tert-butyl decarbonate (48 mg, 0.22 mmol). After stirring at room temperature overnight, the mixture was diluted with DCM, washed with 1 M HCl_{aq}, water and brine separately, dried over sodium sulfate, filtered, and condensed under reduced pressure to give a residue which was purified through flash column chromatography on silica gel (0%-10% methanol in DCM) to yield the title compound (14.3 mg, 19% yield for two steps). ^{1}H NMR (400 MHz, $CDCl_3$) δ 7.89 (d, *J* = 7.9 Hz, 1H), 6.82 (s, 1H), 6.50 (d, *J* = 2.6 Hz, 1H), 6.45 (dd, *J* = 8.8, 2.6 Hz, 1H), 4.11 – 4.06 (m, 2H), 3.82 (s, 3H), 3.81 – 3.76 (m, 4H), 3.72 (t, *J* = 5.8 Hz, 2H), 2.29 (t, *J* = 5.5 Hz, 1H), 1.93 – 1.81 (m, 2H), 1.51 (s, 9H). ^{13}C NMR (101 MHz, $CDCl_3$) δ 154.74, 153.15, 149.04, 122.09, 119.11, 104.90, 99.76, 80.18, 70.65, 69.68, 67.82, 61.97, 55.83, 32.15, 28.54. LC-MS, ESI$^+$, m/z 364.1 $[M+Na]^+$.

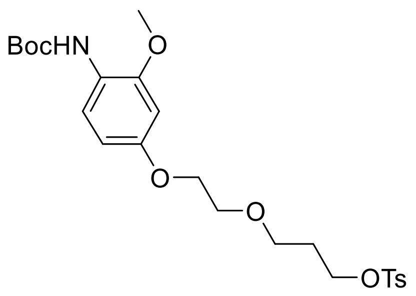

3-(2-(4-((*tert*-butoxycarbonyl)amino)-3-methoxyphenoxy)ethoxy)propyl 4-methylbenzenesulfonate (**8-6**)

To a solution of **8-5** (33 mg, 0.10 mmol), TEA (0.027 mL, 0.19 mmol) and DMAP (1 mg, 0.008 mmol) in DCM (2 mL) was added TsCl (20 mg, 0.1 mmol). After stirring at room temperature overnight, the mixture was diluted with DCM, washed with water and brine, dried over sodium sulfate, filtered, and condensed under reduced pressure to afford a residue which was purified through flash column chromatography on silica gel (0%-10% methanol in DCM) to give the title compound (30 mg, 61% yield). ^{1}H NMR (400 MHz, $CDCl_3$) δ 7.89 (s, 1H), 7.82 – 7.70 (m, 2H), 7.31 (d, *J* = 8.0 Hz, 2H), 6.83 (s, 1H), 6.50 (d, *J* = 2.6 Hz, 1H), 6.42 (dd, *J* = 8.9, 2.6 Hz, 1H), 4.15 (t, *J* = 6.1 Hz, 2H), 4.00 (dd, *J* = 5.4, 4.2 Hz, 2H), 3.82 (s, 3H), 3.68 (dd, *J* = 5.4, 4.2 Hz, 2H), 3.55 (t, *J* = 6.1 Hz, 2H), 2.42 (s, 3H), 1.93 (p, *J* = 6.1 Hz, 2H), 1.51 (s, 9H). ^{13}C NMR (101 MHz, $CDCl_3$) δ 154.75, 153.12, 148.99, 144.82, 133.33, 129.94, 128.02, 122.03, 119.04, 104.82, 99.69, 80.15, 69.62, 67.81, 67.76, 66.89, 55.81, 29.45, 28.52, 21.71. LC-MS, ESI$^+$, m/z 496.1 [M+H]$^+$.

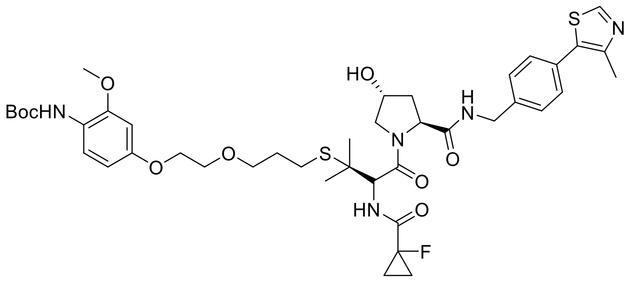

tert-butyl (4-(2-(3-(((*S*)-3-(1-fluorocyclopropane-1-carboxamido)-4-((2*S*,4*R*)-4-hydroxy-2-((4-(4-methylthiazol-5-yl)benzyl)carbamoyl)pyrrolidin-1-yl)-2-methyl-4-oxobutan-2-yl)thio)propoxy)ethoxy)-2-methoxyphenyl)carbamate (**8-7**)

30 mg **8-7** was obtained from 27 mg **10** following general procedure 9 (70% yield). ^{1}H NMR (500 MHz, $CDCl_3$) δ 8.65 (s, 1H), 7.87 (d, *J* = 6.0 Hz, 1H), 7.38 – 7.29 (m, 5H), 7.20 (dd, *J* = 8.0, 3.2 Hz, 1H), 6.81 (s, 1H), 6.48 (d, *J* = 2.6 Hz, 1H), 6.44 (dd, *J* = 8.8, 2.6 Hz, 1H), 4.81 – 4.73 (m, 2H), 4.44 (d, *J* = 5.8 Hz, 3H), 4.07 – 4.02 (m, 2H), 3.99 – 3.94 (m, 1H), 3.80 (s, 3H), 3.74 – 3.69 (m, 3H), 3.57 – 3.47 (m, 2H), 3.03 (s, 1H), 2.68 – 2.60 (m, 1H), 2.60 – 2.53 (m, 1H), 2.50 (s, 3H), 2.42 – 2.35 (m, 1H), 2.24 – 2.16 (m, 1H), 1.79 – 1.68 (m, 2H), 1.50 (s, 9H), 1.36 – 1.24 (m, 10H). ^{19}F NMR (471 MHz, $CDCl_3$) δ -197.69. ^{13}C NMR (126 MHz, $CDCl_3$) δ 170.88, 170.42 (d, *J* = 20.9 Hz), 170.04, 154.83, 153.21, 150.37, 149.15, 148.63, 138.21, 131.72, 131.11, 129.61, 128.23, 122.00, 119.32, 104.91, 99.67, 78.27 (d, *J* = 231.44 Hz), 70.11, 69.81, 69.55, 67.95, 59.18, 56.64, 56.14, 55.82, 48.15, 43.18, 36.95, 29.62, 28.53, 25.88, 25.16, 25.11, 16.21, 14.00 (d, *J* = 9.08 Hz), 13.93 (d, *J* = 9.17 Hz). LC-MS, ESI$^+$, m/z 858.95 [M+H]$^+$.

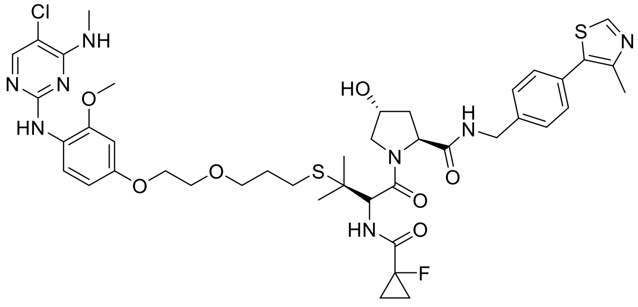

(2*S*,4*R*)-1-((*S*)-3-((3-(2-(4-((5-chloro-4-(methylamino)pyrimidin-2-yl)amino)-3-methoxyphenoxy)ethoxy)propyl)thio)-2-(1-fluorocyclopropane-1-carboxamido)-3-methylbutanoyl)-4-hydroxy-N-(4-(4-methylthiazol-5-yl)benzyl)pyrrolidine-2-carboxamide (**XL01149**)

To a solution of **8-7** (30 mg, 0.035 mmol) in DCM (1 mL) was added HCl (4N in 1,4-dioxane, 1 mL). After stirring at room temperature overnight, the mixture was condensed under reduced pressure to give a solid which was dissolved in iso-propanol, followed by addition of 2,5-dichloro-N-methylpyrimidin-4-amine (7 mg, 3.9 mmol). After heating in a microwave reactor at 100 °C for 8h, the mixture was diluted with DCM, washed with water and brine, dried over sodium sulfate, filtered, and condensed to afford a residue which was purified with preparative HPLC under acidic condition (5-95 % CH_3CN in 0.1 % aq. HCO_2H) to yield the title compound (7.5 mg, 24% for two steps). ^{1}H NMR (500 MHz, $CDCl_3$) δ 8.66 (s, 1H), 8.22 (d, *J* = 8.8 Hz, 1H), 7.85 (s, 1H), 7.40 – 7.30 (m, 5H), 7.28 (s, 1H), 7.19 (dd, *J* = 7.9, 3.1 Hz, 1H), 6.54 (d, *J* = 2.6 Hz, 1H), 6.49 (dd, *J* = 8.8, 2.6 Hz, 1H), 5.24 (d, *J* = 4.6 Hz, 1H), 4.81 – 4.74 (m, 2H), 4.46 (d, *J* = 5.9 Hz, 2H), 4.39 (s, 1H), 4.12 – 4.04 (m, 2H), 3.97 (d, *J* = 11.2 Hz, 1H), 3.84 (s, 3H), 3.78 – 3.66 (m, 3H), 3.60 – 3.44 (m, 2H), 3.07 (d, *J* = 4.9 Hz, 3H), 2.71 – 2.63 (m, 1H), 2.61 – 2.54 (m, 1H), 2.51 (s, 3H), 2.44 – 2.39 (m, 1H), 2.25 – 2.15 (m, 1H), 1.82 – 1.68 (m, 2H), 1.38 – 1.27 (m, 10H); ^{13}C NMR (126 MHz, $CDCl_3$) δ 170.79, 170.51 (d, *J* = 20.9 Hz), 170.15, 163.12, 158.70, 158.42, 154.41, 152.62, 150.41, 149.76, 148.65, 138.22, 131.75, 131.16, 129.66, 128.30, 123.51, 120.03, 104.77, 99.73, 78.26 (d, *J* = 242.0 Hz), 70.09, 69.82, 69.63, 68.06, 59.09, 56.57, 56.12, 55.92, 48.01, 43.24, 36.85, 29.65, 28.11, 25.96, 25.16, 25.07, 16.21, 14.07 (d, *J* = 10.67 Hz), 13.95 (d, *J* = 10.67 Hz); ^{19}F NMR (471 MHz, $CDCl_3$) δ -197.75; HRMS (ESI$^+$) *m/z*, calcd for $C_{42}H_{52}ClFN_8O_7S_2$: 899.3146 [M + H]$^+$, found 899.2639.

Scheme S9. Synthesis of XL01168

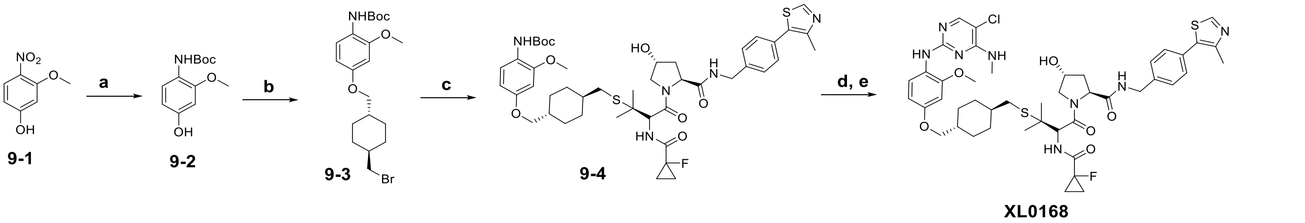

Reagents and conditions: a. i) Pd/C, H_2, Ethyl acetate; ii) Boc_2O, TEA, DCM; b. Trans-1,4-bis(bromomethyl)cyclohexane, Cs_2CO_3, MW, 60 °C, DMF; c. **10**, DBU, THF, d, 4 N HCl in 1,4-dioxane, DCM; e. 2,5-dichloro-N-methylpyrimidin-4-amine, HCl, i-PrOH, MW, 100 °C.

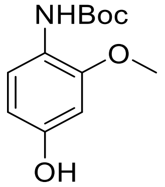

tert-butyl (4-hydroxy-2-methoxyphenyl)carbamate (**9-2**)

To a solution of 3-methoxyl-4-nitrophenol (1 g, 5.9 mmol) in methanol (10 ml) was added 10% Palladium charcoal (100 mg) under nitrogen protection. The resulting mixture was evacuated and backfilled with hydrogen by attaching a hydrogen balloon. After stirring at room temperature overnight, the mixture was filtered through a pad of celite, and the filtrate was condensed to afford a residue which was dissolved in DCM (20 mL). Followed by addition of TEA (1.65 mL, 11.8 mmol) and BoC_2O (1.29 g, 5.9 mmol), the mixture was stirred at room temperature overnight. DCM (30 mL) was added and the resulting mixture was washed with 1N HCl_{aq}, water, and brine separately, then dried over sodium sulfate, filtered, and condensed to give a residue which was purified through flash column chromatography on silica gel (0%-10% methanol in DCM) to give the title compound (200 mg, 14% yield for two steps). ^{1}H NMR (500 MHz, $CDCl_3$) δ 7.70 (s, 1H), 6.76 (s, 1H),

6.40 (d, *J* = 2.6 Hz, 1H), 6.35 (dd, *J* = 8.7, 2.6 Hz, 1H), 6.17 (s, 1H), 3.76 (s, 3H), 1.51 (s, 9H). ^{13}C NMR (126 MHz, $CDCl_3$) δ 153.68, 152.43, 149.63, 120.66, 120.37, 107.04, 99.27, 80.44, 55.70, 28.52. LC-MS, ESI$^-$, m/z 238 [M-H]$^-$.

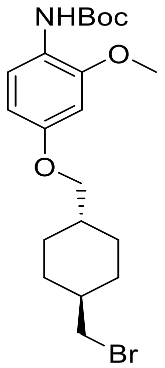

tert-butyl (4-(((1*R*,4*R*)-4-(bromomethyl)cyclohexyl)methoxy)-2-methoxyphenyl)carbamate (**9-3**)

In a microwave tube was added **9-2** (20 mg, 0.084 mmol), *trans*-1,4-bis(bromomethyl)cyclohexane (68 mg, 0.25 mmol), DMF (1 ml), and Cs_2CO_3 (82 mg, 0.25 mmol). After heating in microwave at 60 °C for 30 min, the mixture was cooled down and added with water. The resulting mixture was then extracted with ethyl acetate (3×), the combined organic phase was washed with water and brine, dried over sodium sulfate, filtered, and condensed to afford a residue which was purified through flash column chromatography on silica gel to give the title compound (15 mg, 42% yield). ^{1}H NMR (400 MHz, $CDCl_3$) δ 7.81 (d, *J* = 7.5 Hz, 1H), 6.73 (s, 1H), 6.39 – 6.32 (m, 2H), 3.76 (s, 3H), 3.67 (d, *J* = 6.3 Hz, 2H), 3.23 (d, *J* = 6.3 Hz, 2H), 1.95 – 1.83 (m, 4H), 1.72 – 1.62 (m, 1H), 1.62 – 1.52 (m, 1H), 1.44 (s, 9H), 1.10 – 0.92 (m, 4H); ^{13}C NMR (101 MHz, $CDCl_3$) δ 155.30, 153.18, 149.05, 121.68, 119.24, 104.84, 99.37, 73.60, 55.80, 40.41, 40.30, 37.78, 31.22, 29.40, 28.56. LC-MS, ESI$^+$, m/z 450 [M+Na]$^+$, 452 [M+Na+2]$^+$.

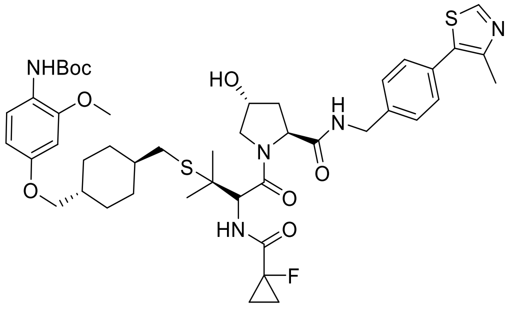

tert-butyl (4-(((1*S*,4*R*)-4-((((*S*)-3-(1-fluorocyclopropane-1-carboxamido)-4-((2*S*,4*R*)-4-hydroxy-2-((4-(4-methylthiazol-5-yl)benzyl)carbamoyl)pyrrolidin-1-yl)-2-methyl-4-oxobutan-2-yl)thio)methyl)cyclohexyl)methoxy)-2-methoxyphenyl)carbamate (**9-4**)

25 mg **9-4** was obtained from 18 mg **10** following general procedure 9 (84% yield). ^{1}H NMR (500 MHz, $CDCl_3$) δ 8.65 (s, 1H), 7.87 (s, 1H), 7.40 – 7.32 (m, 5H), 7.24 (dd, *J* = 8.0, 3.4 Hz, 1H), 6.80 (s, 1H), 6.46 – 6.39 (m, 2H), 4.79 (t, *J* = 7.9 Hz, 1H), 4.74 (d, *J* = 7.8 Hz, 1H), 4.53 (s, 1H), 4.46 (d, *J* = 5.9 Hz, 2H), 4.05 (d, *J* = 11.3 Hz, 1H), 3.82 (s, 3H), 3.73 (dd, *J* = 11.2, 3.9 Hz, 1H), 3.69 (d, *J* = 6.3 Hz, 2H), 2.90 (d, *J* = 4.4 Hz, 1H), 2.51 (s, 3H), 2.50 – 2.34 (m, 3H), 2.28 – 2.19 (m, 1H), 1.87 (t, *J* = 10.3 Hz, 4H), 1.71 – 1.65 (m, 1H), 1.51 (s, 9H), 1.41 – 1.28 (m, 11H), 1.08 – 0.90 (m, 4H). ^{19}F NMR (471 MHz, $CDCl_3$) δ -197.70. ^{13}C NMR (126 MHz, $CDCl_3$) δ 170.83, 170.60 (d, *J* = 20.7 Hz), 170.14, 155.28, 153.17, 150.39, 149.07, 148.67, 138.18, 131.71, 131.17, 129.65, 128.15, 121.67, 119.25, 104.83, 99.35, 78.28 (d, *J* = 232.0 Hz), 73.64, 70.27, 59.10, 56.73, 56.36, 55.80, 47.80, 43.24, 38.33, 37.80, 36.88, 35.37, 32.51, 32.46, 29.62, 28.55, 25.78, 25.47, 16.24, 14.05 (d, *J* = 11.0 Hz), 13.96 (d, *J* = 11.0 Hz). LC-MS, ESI$^+$, m/z 882 [M+H]$^+$.

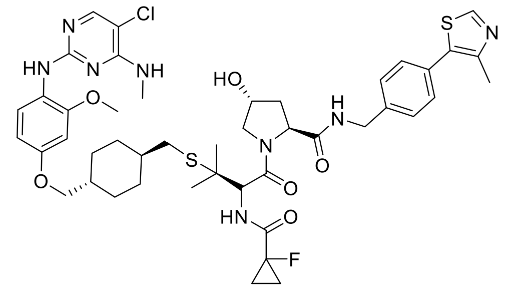

(2*S*,4*R*)-1-((*S*)-3-((((1*R*,4*S*)-4-((4-((5-chloro-4-(methylamino)pyrimidin-2-yl)amino)-3-methoxyphenoxy)methyl)cyclohexyl)methyl)thio)-2-(1-fluorocyclopropane-1-carboxamido)-3-methylbutanoyl)-4-hydroxy-N-(4-(4-methylthiazol-5-yl)benzyl)pyrrolidine-2-carboxamide (**XL01168**)

Compound **9-4** (25 mg, 0.028 mmol) was dissolved in DCM (1 ml), followed by addition of HCl (4N HCl in 1,4-dioxane, 1 mL). After stirring at room temperature overnight, the mixture was condensed under reduced pressure to give a solid which was used to next step directly without further purification. The solid from previous step was dissolved in iso-propanol (1 mL), followed by addition of 2,5-dichloro-N-methylpyrimidin-4-amine (4.8 mg, 0.027 mmol) and HCl (4N in 1,4-dioxane, 0.006 ml). The resulting mixture was heated in microwave at 100 °C for 9h, cooled down, diluted with water and extracted with DCM (3×). The combined organic phase was washed with water and brine, dried over sodium sulfate, filtered, condensed to give a residue which was purified through preparation HPLC under acidic condition (5-95 % CH_3CN in 0.1 % aq. HCO_2H) to yield the title compound (10 mg, 38% yield for two steps). ^1H NMR (500 MHz, $CDCl_3$) δ 8.65 (s, 1H), 8.26 (d, *J* = 8.8 Hz, 1H), 7.87 (s, 1H), 7.41 – 7.31 (m, 5H), 7.25 – 7.21 (m, 2H), 6.56 – 6.35 (m, 2H), 5.24 – 5.18 (m, 1H), 4.80 (t, *J* = 7.9 Hz, 1H), 4.74 (d, *J* = 7.8 Hz, 1H), 4.54 (s, 1H), 4.46 (d, *J* = 5.9 Hz, 2H), 4.07 (d, *J* = 11.3 Hz, 1H), 3.86 (s, 3H), 3.75 – 3.70 (m, 3H), 3.08 (d, *J* = 4.9 Hz, 3H), 2.52 (s, 3H), 2.51 – 2.45 (m, 1H), 2.45-2.34 (m, 2H), 2.27 – 2.20 (m, 1H), 1.88 (t, *J* = 12.6 Hz, 4H), 1.76 – 1.64 (m, 1H), 1.40 – 1.25 (m, 11H), 1.08 – 0.91 (m, 4H); ^{13}C NMR (126 MHz, $CDCl_3$) δ 170.75, 170.66 (d, *J* = 23.3 Hz), 170.21, 158.66, 158.49, 154.72, 152.89, 150.40, 149.46, 148.69, 138.17, 131.71, 131.20, 129.68, 128.18, 123.27, 119.69, 104.64, 104.56, 99.41, 78.3 (d, *J* = 234.4 Hz), 73.71, 70.30, 59.02, 56.69, 56.39, 55.88, 47.71, 43.27, 38.34, 37.82, 36.79, 35.39, 32.54, 32.48, 29.65, 28.11, 25.81, 25.45, 16.25, 14.09 (d, *J* = 10.3 Hz), 13.96 (d, *J* = 10.3 Hz); LC-MS, ESI^-, m/z 921 $[M-H]^-$. HRMS (ESI^+) *m/z*, calcd for $C_{45}H_{56}ClFN_8O_6S_2$:923.3509 $[M + H]^+$, found 923.3479.

Scheme S10. Synthesis of VHL NanoBRET tracer

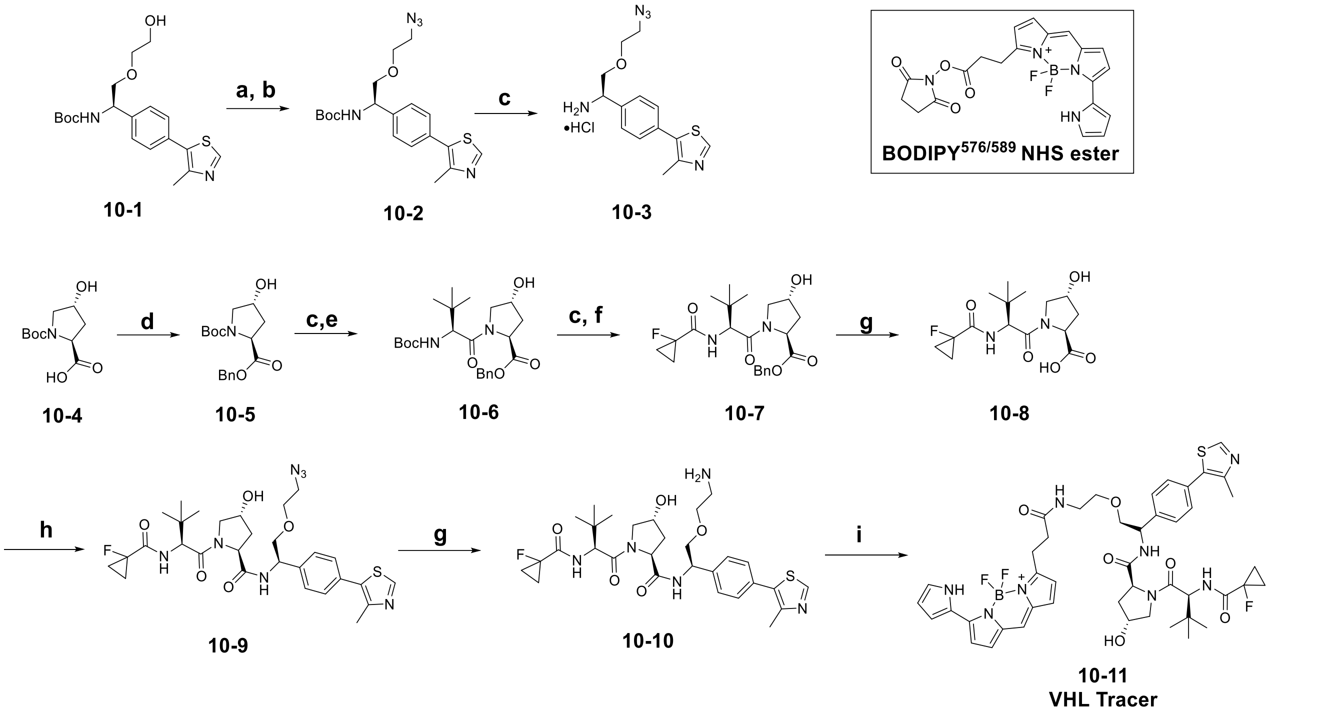

Reagents and conditions: a. DIPEA, MsCl, DCM; b. NaN_3, DMF, 60 °C; c. 4N HCl, DCM; d. BnBr, K_2CO_3, DMF; e. Boc-tert-Leucine, HATU, TEA, DCM; f. 1-fluorocyclopropanecarboxylic acid, HATU, TEA, DCM; g. H_2, 10% Pd/C, ethanol, ethyl acetate; h. **10-3**, HATU, TEA, DCM; i. BODIPY$^{576/589}$ NHS ester, DIPEA, DMF.

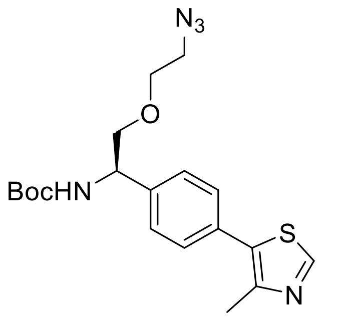

tert-butyl (*R*)-(2-(2-azidoethoxy)-1-(4-(4-methylthiazol-5-yl)phenyl)ethyl)carbamate (**10-2**)

To a solution of alcohol compound **10-1** (60 mg, 0.16 mmol) in DCM at 0 °C was added TEA (55 μL, 0.40 mmol) and MsCl (18 μL, 0.24 mmol). The resulting mixture was stirred at 0 °C for 15 min then diluted with DCM and washed with water and brine. The organic phase was then dried over sodium sulfate, filtered, and condensed to afford a residue which was used to the next without further purification. The residue from last step was dissolved in DMF and added with NaN_3 (11 mg, 0.17 mmol). The mixture was stirred at 55 °C overnight, then cooled down to room temperature and diluted with water. The resulting mixture was then extracted with ethyl acetate (3×). The combined organic phase was washed with water and brine, dried over sodium sulfate, filtered, and condensed to afford a residue which was purified with flash column chromatography on silica gel to give the title compound (60 mg, 94% yield for two steps). ^{1}H NMR (500 MHz, $CDCl_3$) δ 8.65 (s, 1H), 7.44 – 7.33 (m, 4H), 5.36 (s, 1H), 4.87 (s, 1H), 3.77 (dd, *J* = 9.7, 4.5 Hz, 1H), 3.68 (ddd, *J* = 15.9, 9.3, 5.2 Hz, 2H), 3.63 – 3.56 (m, 1H), 3.35 (t, *J* = 4.9 Hz, 2H), 2.52 (s, 3H), 1.42 (s, 9H). ^{13}C NMR (126 MHz, $CDCl_3$) δ 155.51, 150.29, 148.66, 140.23, 131.72, 131.18, 129.48, 127.14, 79.89, 74.14, 70.32, 54.07, 50.83, 28.45, 16.19. LC-MS, ESI$^+$, m/z 404.2 $[M+H]^+$.

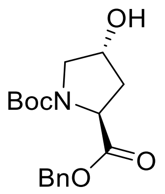

2-benzyl 1-(*tert*-butyl) (2*S*,4*R*)-4-hydroxypyrrolidine-1,2-dicarboxylate (**10-5**)

To a solution of Boc-protected hydroxyl proline (**10-4**, 1.5 g, 6.5 mmol) in DMF was added benzylbromide (1.22 g, 7.1 mmol) and K_2CO_3 (2.24 g, 16.2 mmol). After stirring at room temperature for 24h, the mixture was diluted with water and extracted with ethyl acetate (3×). The combined organic phase was washed with water and brine, dried over sodium sulfate, filtered and condensed to afford a residue which was purified with flash column chromatography on silica gel to give the title compound (1.63 g, 78% yield). ^{1}H NMR (400 MHz, $CDCl_3$) δ 7.39 – 7.27 (m, 5H), 5.27 – 5.03 (m, 2H), 4.56 – 4.36 (m, 2H), 3.65 – 3.59 (m, 1H), 3.58 – 3.39 (m, 1H), 2.39 – 2.15 (m, 2H), 2.11 – 2.02 (m, 1H), 1.45 (s, 3H), 1.34 (s, 6H). LC-MS, 344 $[M+Na]^+$.

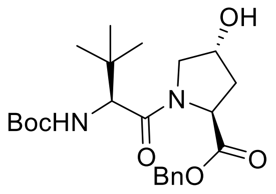

benzyl (2*S*,4*R*)-1-((*S*)-2-((tert-butoxycarbonyl)amino)-3,3-dimethylbutanoyl)-4-hydroxypyrrolidine-2-carboxylate (**10-6**)

To a solution of **10-5** (1.63 g, 5 mmol) in DCM (6.3 mL) was added 4N HCl (6.3 mL). The resulting mixture was stirred at room temperature overnight and condensed to afford a solid which was washed twice with ethyl ether and dried to give a solid (1.16 g, 89% yield). The obtained solid (333 mg, 1.3 mmol) was suspended in DCM (6 mL) and added with TEA (451 μL, 3.2 mmol), Boc-Tle-OH (300 mg, 1.3 mmol), and HATU (518 mg, 1.36 mmol). The resulting mixture was stirred at room temperature for 2h, then diluted with DCM, washed with water and brine, dried over sodium sulfate, filtered, and condensed to afford a residue which was purified with flash column chromatography on silica gel to yield the title compound (555 mg, 98% yield). ^{1}H NMR (500 MHz, $CDCl_3$) δ 7.38 – 7.27 (m, 5H), 5.24 – 5.12 (m, 3H), 4.75 (t, *J* = 8.5 Hz, 1H), 4.49 (s, 1H), 4.17 (d, *J* = 9.4 Hz, 1H), 4.04 (d, *J* = 11.1 Hz, 1H), 3.69 (dd, *J* = 11.1, 3.3 Hz, 1H), 2.63 (d, *J* = 2.8 Hz, 1H), 2.35 (dd, *J* = 13.1, 8.2 Hz, 1H), 1.99 (ddd, *J* = 13.1, 9.0, 4.2 Hz, 1H), 1.41 (s, 9H), 0.99 (s, 9H). ^{13}C NMR (126 MHz, $CDCl_3$) δ 172.09, 171.48, 135.77, 128.68, 128.40, 128.35, 80.13, 70.43, 67.00, 58.85, 57.74, 56.42, 38.76, 37.85, 28.48, 26.44. LC-MS, ESI$^+$, 457.1 $[M+Na]^+$.

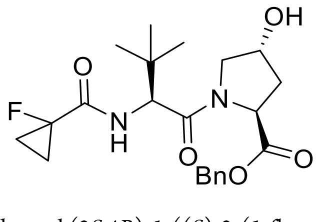

benzyl (2*S*,4*R*)-1-((*S*)-2-(1-fluorocyclopropane-1-carboxamido)-3,3-dimethylbutanoyl)-4-hydroxypyrrolidine-2-carboxylate (**10-7**)

To a solution of **10-6** (555 mg, 1.28 mmol) in DCM (2 mL) was added 4N HCl (2 mL, 8 mmol). The resulting mixture was stirred at room temperature overnight and condensed to afford a solid which was washed with diethyl ether and dried to give 427mg solid (93% yield) which was used to next step. To a suspension of the obtained solid (234 mg, 0.63 mmol) in DCM (6 mL) was added with TEA (208 μL, 1.50 mmol), HATU (239 mg, 0.63 mmol) and 1-fluorocyclopropane-1-carboxylic acid (62 mg, 0.60 mmol). The resulting mixture was stirred at room temperature overnight, then diluted with DCM, washed with water and brine, dried over sodium sulfate, filtered, and condensed to afford a residue which was purified via flash column chromatography on silica gel to yield the title compound (200 mg, 79% yield). ^{1}H NMR

(500 MHz, $CDCl_3$) δ 7.36 – 7.28 (m, 5H), 7.11 (dd, *J* = 9.0, 3.0 Hz, 1H), 5.19 (d, *J* = 12.3 Hz, 1H), 5.12 (d, *J* = 12.3 Hz, 1H), 4.71 (t, *J* = 8.5 Hz, 1H), 4.57 (d, *J* = 9.1 Hz, 1H), 4.46 (s, 1H), 3.94 (d, *J* = 11.1 Hz, 1H), 3.71 (dd, *J* = 11.1, 3.9 Hz, 1H), 3.65 (d, *J* = 4.9 Hz, 1H), 2.33 (dd, *J* = 13.3, 8.0 Hz, 1H), 1.99 (ddd, *J* = 13.5, 9.2, 4.5 Hz, 1H), 1.33 – 1.21 (m, 4H), 1.00 (s, 9H). ^{13}C NMR (126 MHz, $CDCl_3$) δ 172.02, 170.10, 169.99 (d, *J* = 20.8 Hz), 135.68, 128.65, 128.40, 128.38, 78.22 (d, *J* = 232.4 Hz), 70.31, 67.02, 58.12, 57.33, 56.61, 37.80, 36.13, 26.40, 13.73 (t, *J* = 10.1 Hz), 13.65 (t, *J* = 9.8 Hz). LC-MS, ESI$^+$, 443.1 [M+Na]$^+$.

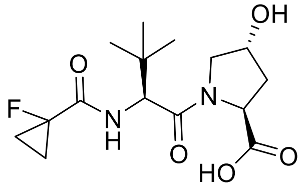

(2*S*,4*R*)-1-((*S*)-2-(1-fluorocyclopropane-1-carboxamido)-3,3-dimethylbutanoyl)-4-hydroxypyrrolidine-2-carboxylic acid (**10-8**)

Under N_2 protection, 10% Pd/C (20 mg) was added to a solution of **10-7** (200 mg, 0.48 mmol) in ethanol and ethyl acetate (2 ml/2 ml). The N_2 was then replaced with H_2 and the mixture was stirred at room temperature overnight. Filter the mixture through 0.45 μM filter and the filtrate was condensed and dried to give the title compound (120 mg, 76% yield). ^1H NMR (500 MHz, DMSO-d_6) δ 7.04 (dd, *J* = 9.2, 2.4 Hz, 1H), 4.97 (s, 1H), 4.41 (d, *J* = 9.2 Hz, 1H), 4.22 – 4.01 (m, 2H), 3.55 – 3.37 (m, 2H), 1.94 (dd, *J* = 12.8, 8.0 Hz, 1H), 1.71 (ddd, *J* = 13.3, 9.2, 4.5 Hz, 1H), 1.21 – 1.11 (m, 2H), 1.06 – 0.97 (m, 2H), 0.81 (d, *J* = 15.0 Hz, 9H). ^{13}C NMR (126 MHz, $CDCl_3$) δ 173.01, 169.08, 168.02 (d, *J* = 20.5 Hz), 78.04 (d, *J* = 232.8 Hz), 68.77, 57.75, 56.38, 56.25, 37.20, 35.95, 26.07, 12.91 (d, *J* = 10.5 Hz), 12.67 (d, *J* = 10.3 Hz). ^{19}F NMR (471 MHz, DMSO-d_6) δ -191.54. LC-MS, ESI$^-$, m/z 329.0 [M-H]$^-$.

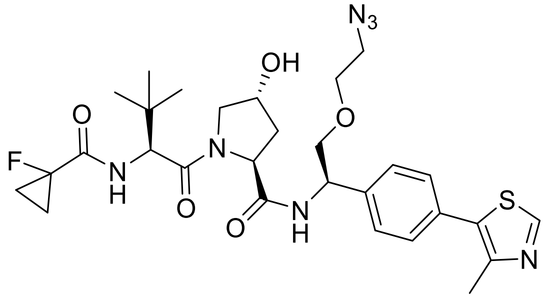

(2*S*,4*R*)-N-((*R*)-2-(2-azidoethoxy)-1-(4-(4-methylthiazol-5-yl)phenyl)ethyl)-1-((S)-2-(1-fluorocyclopropane-1-carboxamido)-3,3-dimethylbutanoyl)-4-hydroxypyrrolidine-2-carboxamide (**10-9**)

To a solution of **10-2** (60 mg, 0.15 mmol) in DCM (371 μL) was added 4N HCl (371 μL). The mixture was stirred at room temperature for 2h and condensed to afford a solid (44 mg, 86% yield) which was used to next step directly. To a suspension of the obtained solid (11 mg, 0.033 mmol) in DCM (1 ml) was added **10-8** (11 mg, 0.032 mmol), TEA (14.8 μL, 0.1 mmol), and HATU (12.4 mg, 0.033 mmol). The resulting mixture was stirred at room temperature for 1h, then diluted with DCM, washed with water and brine, dried over sodium sulfate and condensed to afford a residue which was purified via flash column chromatography on silica gel to give the title compound (20 mg, 97% yield). ^1H NMR (500 MHz, $CDCl_3$) δ 8.66 (s, 1H), 7.47 (t, *J* = 7.9 Hz, 1H), 7.45 – 7.34 (m, 4H), 7.10 (dd, *J* = 8.8, 3.3 Hz, 1H), 5.15 (dt, *J* = 7.8, 4.9 Hz, 1H), 4.72 (t, *J* = 7.9 Hz, 1H), 4.61 (d, *J* = 8.9 Hz, 1H), 4.48 (s, 1H), 3.98 (d, *J* = 11.3 Hz, 1H), 3.77 (d, *J* = 4.9 Hz, 2H), 3.70 – 3.63 (m, 2H), 3.62 – 3.56 (m, 1H), 3.34 (t, *J* = 5.0 Hz, 2H), 2.51 (s, 3H), 2.36 (ddd, *J* = 12.9, 7.8, 4.8 Hz, 1H), 2.17 – 2.06 (m, 1H), 1.40 – 1.21 (m, 4H), 1.07 (s, 9H). ^{13}C NMR (126 MHz, $CDCl_3$) δ 171.17, 170.63, 170.32 (d, *J* = 20.6 Hz), 150.44, 148.66, 139.49, 131.69, 131.32, 129.57, 127.35, 78.31 (d, *J* = 237.5 Hz), 73.70, 70.24, 70.16, 59.00, 57.60, 56.80, 52.90, 50.78, 36.47, 35.76, 26.58, 16.18, 13.84 (d, *J* = 10.2 Hz), 13.79 (d, *J* = 10.1 Hz). ^{19}F NMR (471 MHz, $CDCl_3$) δ -197.36. LC-MS, ESI$^+$, m/z 616.4 [M+H]$^+$.

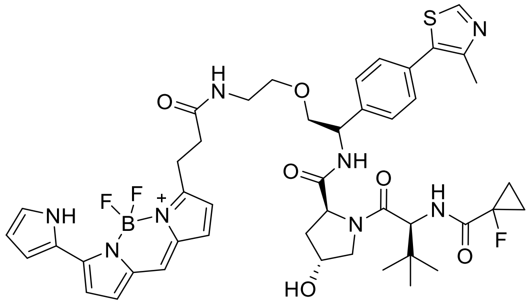

5,5-difluoro-3-(3-((2-((*S*)-2-((2*S*,4*R*)-1-((*S*)-2-(1-fluorocyclopropane-1-carboxamido)-3,3-dimethylbutanoyl)-4-hydroxypyrrolidine-2-carboxamido)-2-(4-(4-methylthiazol-5-yl)phenyl)ethoxy)ethyl)amino)-3-oxopropyl)-7-(1H-pyrrol-2-yl)-5H-5l4-dipyrrolo[1,2-c:2',1'-f][1,3,2]diazaborinin-4-ium (**10-11**, VHL Tracer)

To a solution of **10-9** (80 mg, 0.13 mmol) in methanol and ethyl acetate mixed solvent (1.5 mL/1.5 mL) was added 10% Pd/C (8 mg) under N_2 protection, the N_2 was then replaced with H_2 and the mixture was stirred at room temperature for 2h, then filtered through 0.45 μM filter, condensed, and dried to give a residue which was used to the next step directly. The residue was dissolved in DMF (1 mL), followed by addition of DIPEA (4 μL, 0.023 mmol) and BODIPY$^{576/589}$ NHS ester (5 mg, 0.012 mmol). The resulting mixture was stirred at room temperature overnight then purified via reverse phase prep-HPLC under acidic condition (5-95 % CH_3CN in 0.1 % aq. HCO_2H) to give the title compound (2 mg, 19% yield). ^{1}H NMR (500 MHz, $CDCl_3$) δ 10.37 (s, 1H), 8.65 (s, 1H), 7.35 (t, *J* = 7.1 Hz, 3H), 7.28 (s, 2H), 7.15 – 7.09 (m, 2H), 7.06 – 6.95 (m, 4H), 6.87 (d, *J* = 4.5 Hz, 1H), 6.82 (d, *J* = 3.9 Hz, 1H), 6.38 – 6.30 (m, 2H), 5.09 (dd, *J* = 11.1, 7.0 Hz, 1H), 4.59 (t, *J* = 7.9 Hz, 1H), 4.54 (d, *J* = 8.6 Hz, 1H), 4.41 (s, 1H), 3.91 (d, *J* = 11.0 Hz, 1H), 3.72 – 3.63 (m, 2H), 3.61 (dd, *J* = 11.2, 3.9 Hz, 1H), 3.57 – 3.49 (m, 2H), 3.48 – 3.42 (m, 1H), 3.41 – 3.31 (m, 2H), 3.25 – 3.14 (m, 1H), 2.75 – 2.66 (m, 1H), 2.65 – 2.57 (m, 1H), 2.50 (s, 3H), 2.33 – 2.23 (m, 1H), 2.16 (s, 1H), 1.89 – 1.80 (m, 1H), 1.38 – 1.24 (m, 4H), 1.06 (s, 9H). ^{13}C NMR (126 MHz, $CDCl_3$) δ 172.46, 171.17, 170.75, 170.38 (d, *J* = 19.9 Hz), 156.34, 150.43, 148.75, 139.23, 137.36, 133.62, 131.61, 131.47, 129.62, 127.23, 126.57, 126.17, 123.82, 123.36, 120.50, 117.95, 117.29, 111.68, 79.42, 73.41, 70.42, 70.15, 58.81, 57.85, 56.73, 53.25, 39.16, 36.35, 35.52, 35.37, 26.65, 25.02, 16.26, 13.89 (d, *J* = 10.4 Hz), 13.78 (d, *J* = 10.3 Hz). LC-MS, ESI$^+$, m/z 901.5 [M+H]$^+$. HRMS (ESI$^+$) *m/z,* calcd for $C_{45}H_{52}F3BN8O_6S$: 901.3848 [M + H]$^+$, found 901.3761.

Scheme S11. Synthesis of LRRK2 NanoBRET tracer

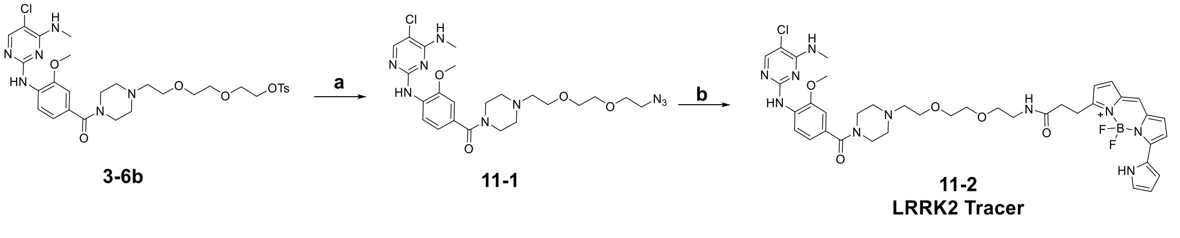

Reagents and conditions: a. NaN_3, DMF, 60 °C; b. i). H_2, 10% Pd/C, methanol; ii). BODIPY$^{576/589}$ NHS ester, DIPEA, DMF.

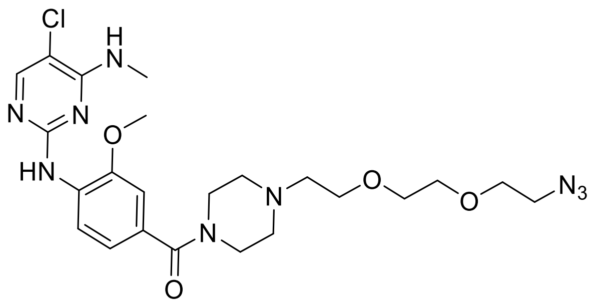

(4-(2-(2-(2-azidoethoxy)ethoxy)ethyl)piperazin-1-yl)(4-((5-chloro-4-(methylamino)pyrimidin-2-yl)amino)-3-methoxyphenyl)methanone (**11-1**)

To a solution of **3-6b** (45 mg, 0.068 mmol) in DMF (1 mL) was added NaN_3 (4.5 mg, 0.069 mmol). After stirring at 60 °C overnight, the mixture was cooled down and diluted with water, then extracted with DCM (3×). The combined organic phase was washed with water and brine, dried over sodium sulfate, filtered and condensed to afford a residue which was purified via flash column chromatography on silica gel to give the title compound (24 mg, 67% yield). ^{1}H NMR (500 MHz, $CDCl_3$) δ 8.54 (d, *J* = 8.3 Hz, 1H), 7.91 (s, 1H), 7.63 (s, 1H), 7.05 – 6.94 (m, 2H), 5.36 – 5.28 (m, 1H), 3.92 (s, 3H), 3.81 – 3.44 (m, 12H), 3.37 (t, *J* = 5.0 Hz, 2H), 3.10 (d, *J* = 4.9 Hz, 3H), 2.63 (t, *J* = 5.7 Hz, 2H), 2.52 (s, 4H). ^{13}C NMR (126 MHz, $CDCl_3$) δ 170.56, 158.65, 157.90, 152.76, 147.50, 131.41, 127.93, 120.26, 116.78, 109.65, 105.59, 70.87, 70.58, 70.21, 69.13, 57.86, 55.96, 53.78, 50.82, 28.22. LC-MS, ESI$^+$, m/z 534 [M+H]$^+$.

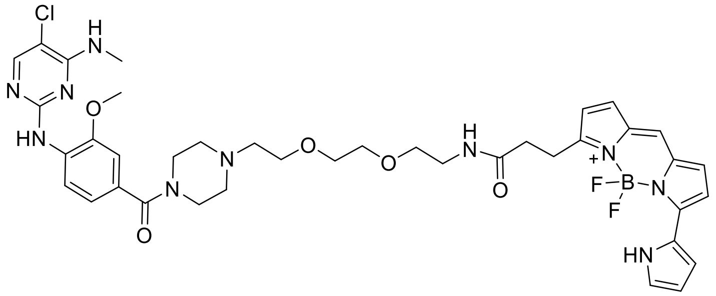

3-(3-((2-(2-(2-(4-(4-((5-chloro-4-(methylamino)pyrimidin-2-yl)amino)-3-methoxybenzoyl)piperazin-1-yl)ethoxy)ethoxy)ethyl)amino)-3-oxopropyl)-5,5-difluoro-7-(1H-pyrrol-2-yl)-5H-5l4-dipyrrolo[1,2-c:2',1'-f][1,3,2]diazaborinin-4-ium (**11-2**, LRRK2 Tracer)

To a solution of **11-1** (24 mg, 0.045 mmol) in methanol was added 10% Pd/C under N_2 protection, the N_2 was then replaced with H_2 and the mixture was stirred at room temperature for 2h. The reaction mixture was then filtered through a 0.45 μM filter, condensed to afford a residue (19 mg, 83% yield) which was used directly to the next step. To the residue from last step (3 mg, 0.006 mmol) in DMF (0.5 mL) was added DIPEA (2 μL, 0.012 mmol) and BODIYP$^{576/589}$ NHS ester (2.5 mg, 0.006 mmol). The mixture was stirred at room temperature for 2h and injected into prep-HPLC for purification under acidic condition (5-95 % CH_3CN in 0.1 % aq. HCO_2H) to give the title compound (4.2 mg, 85% yield). ^{1}H NMR (500 MHz, $CDCl_3$) δ 10.39 (s, 1H), 8.54 (d, *J* = 8.2 Hz, 1H), 7.92 (s, 1H), 7.65 (s, 1H), 7.16 (s, 1H), 7.02 (d, *J* = 4.5 Hz, 1H), 7.01 – 6.92 (m, 4H), 6.83 (dd, *J* = 13.9, 4.2 Hz, 2H), 6.38 – 6.34 (m, 1H), 6.29 (d, *J* = 3.9 Hz, 1H), 6.24 (s, 1H), 5.33 – 5.27 (m, 1H), 3.91 (s, 3H), 3.76 – 3.48 (m, 12H), 3.47 – 3.42 (m, 2H), 3.33 (t, *J* = 7.7 Hz, 2H), 3.10 (d, *J* = 4.9 Hz, 3H), 2.70 – 2.30 (m, 8H). ^{13}C NMR (126 MHz, $CDCl_3$) δ 173.38, 171.87, 170.58, 158.69, 157.88, 155.69, 152.73, 150.54, 147.54, 137.40, 133.63, 131.68, 126.53, 125.90, 123.79, 123.48, 120.40, 117.88, 117.04, 116.87, 111.64, 109.70, 105.71, 70.40, 70.35, 70.00, 57.64, 56.00, 53.48, 39.41, 36.03, 28.25, 24.84. ^{19}F NMR (471 MHz, $CDCl_3$) δ -75.60, -139.84. HRMS (ESI$^+$) *m/z,* calcd for $C_{39}H_{46}BClF_2N_{10}O_5^+$: 819.3480 [M+H]$^+$, found 819.4657.

REFERENCES

(1) Nichols, R. J.; Dzamko, N.; Hutti, J. E.; Cantley, L. C.; Deak, M.; Moran, J.; Bamborough, P.; Reith, A. D.; Alessi, D. R. Substrate Specificity and Inhibitors of LRRK2, a Protein Kinase Mutated in Parkinson's Disease. *Biochem. J.* **2009**, *424* (1), 47–60. DOI: 10.1042/BJ20091035

(2) Kuck, D.; Thölmann, D.; Grützmacher, H.-F. Fast Interannular Proton Transfer in Gaseous Protonated α,ω-Diphenylalkanes: Stereocontrol by the Cyclohexane-1,4-Diyl Unit. *J. Chem. Soc. Perkin Trans. 2* **1990**, No. 2, 251–256. DOI: 10.1039/P29900000251

(3) Farnaby, W.; Koegl, M.; Roy, M. J.; Whitworth, C.; Diers, E.; Trainor, N.; Zollman, D.; Steurer, S.; Karolyi-Oezguer, J.; Riedmueller, C.; Gmaschitz, T.; Wachter, J.; Dank, C.; Galant, M.; Sharps, B.; Rumpel, K.; Traxler, E.; Gerstberger, T.; Schnitzer, R.; Petermann, O.; Greb, P.; Weinstabl, H.; Bader, G.; Zoephel, A.; Weiss-Puxbaum, A.; Ehrenhöfer-Wölfer, K.; Wöhrle, S.; Boehmelt, G.; Rinnenthal, J.; Arnhof, H.; Wiechens, N.; Wu, M.-Y.; Owen-Hughes, T.; Ettmayer, P.; Pearson, M.; McConnell, D. B.; Ciulli, A. BAF Complex Vulnerabilities in Cancer Demonstrated via Structure-Based PROTAC Design. *Nat. Chem. Biol.* **2019**, *15* (7), 672. DOI: 10.1038/s41589-019-0294-6

(4) Shibata, N.; Nagai, K.; Morita, Y.; Ujikawa, O.; Ohoka, N.; Hattori, T.; Koyama, R.; Sano, O.; Imaeda, Y.; Nara, H.; Cho, N.; Naito, M. Development of Protein Degradation Inducers of Androgen Receptor by Conjugation of Androgen Receptor Ligands and Inhibitor of Apoptosis Protein Ligands. *J. Med. Chem.* **2018**, *61* (2), 543–575. DOI: 10.1021/acs.jmedchem.7b00168
